# Unlocking saponin biosynthesis in soapwort

**DOI:** 10.1038/s41589-024-01681-7

**Published:** 2024-07-23

**Authors:** Seohyun Jo, Amr El-Demerdash, Charlotte Owen, Vikas Srivastava, Dewei Wu, Shingo Kikuchi, James Reed, Hannah Hodgson, Alex Harkess, Shengqiang Shu, Chris Plott, Jerry Jenkins, Melissa Williams, Lori-Beth Boston, Elia Lacchini, Tongtong Qu, Alain Goossens, Jane Grimwood, Jeremy Schmutz, Jim Leebens-Mack, Anne Osbourn

**Affiliations:** 1 Department of Biochemistry and Metabolism, John Innes Centre, Norwich Research Park, Norwich, UK; 2 Department of Chemistry, Faculty of Sciences, Mansoura University, Mansoura, Egypt; 3 Department of Botany, School of Life Sciences, Central University of Jammu, Jammu, India; 4 HudsonAlpha Institute for Biotechnology, Huntsville, AL USA; 5 US Department of Energy Joint Genome Institute, Lawrence Berkeley National Laboratory, Berkeley, CA USA; 6 Department of Plant Biotechnology and Bioinformatics, Ghent University, Ghent, Belgium; 7 VIB Centre for Plant Systems Biology, Ghent, Belgium; 8 Department of Plant Biology, Miller Plant Sciences, University of Georgia, Athens, GA USA

**Keywords:** Plant sciences, Natural products, Enzymes, Biosynthesis

## Abstract

Soapwort (*Saponaria officinalis*) is a flowering plant from the Caryophyllaceae family with a long history of human use as a traditional source of soap. Its detergent properties are because of the production of polar compounds (saponins), of which the oleanane-based triterpenoid saponins, saponariosides A and B, are the major components. Soapwort saponins have anticancer properties and are also of interest as endosomal escape enhancers for targeted tumor therapies. Intriguingly, these saponins share common structural features with the vaccine adjuvant QS-21 and, thus, represent a potential alternative supply of saponin adjuvant precursors. Here, we sequence the *S*. *officinalis* genome and, through genome mining and combinatorial expression, identify 14 enzymes that complete the biosynthetic pathway to saponarioside B. These enzymes include a noncanonical cytosolic GH1 (glycoside hydrolase family 1) transglycosidase required for the addition of d-quinovose. Our results open avenues for accessing and engineering natural and new-to-nature pharmaceuticals, drug delivery agents and potential immunostimulants.

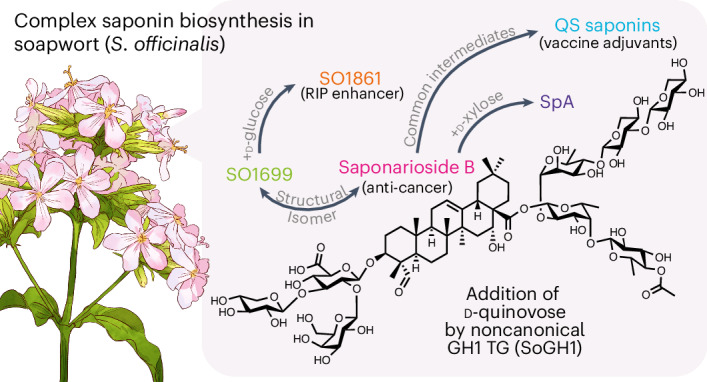

## Abstract (web summary)

Soapwort (*Saponaria* *officinalis*) is a rich reservoir of triterpenoid glycosides that often have important pharmaceutical, nutraceutical and agronomical potential. Here, the authors elucidate the complete biosynthetic pathway of saponarioside B, a major saponin constituent in soapwort.

## Main

Saponins are plant glycosides that are characterized by their ability to form stable foams in water. Saponin-producing plants such as soapwort (*Saponaria officinalis*) have been used as sources of natural soaps for hundreds of years. Indeed, soapwort extract is believed to have been used as a gentle soap to treat the Shroud of Turin^1^. The genus name *Saponaria* is derived from the Latin for soap (*sapo*), while the species name *officinalis* relates to its medical uses. In folk medicine, soapwort extracts are used to treat symptoms of syphilis, rheumatism and bronchitis^2^.

**Fig. 1:**
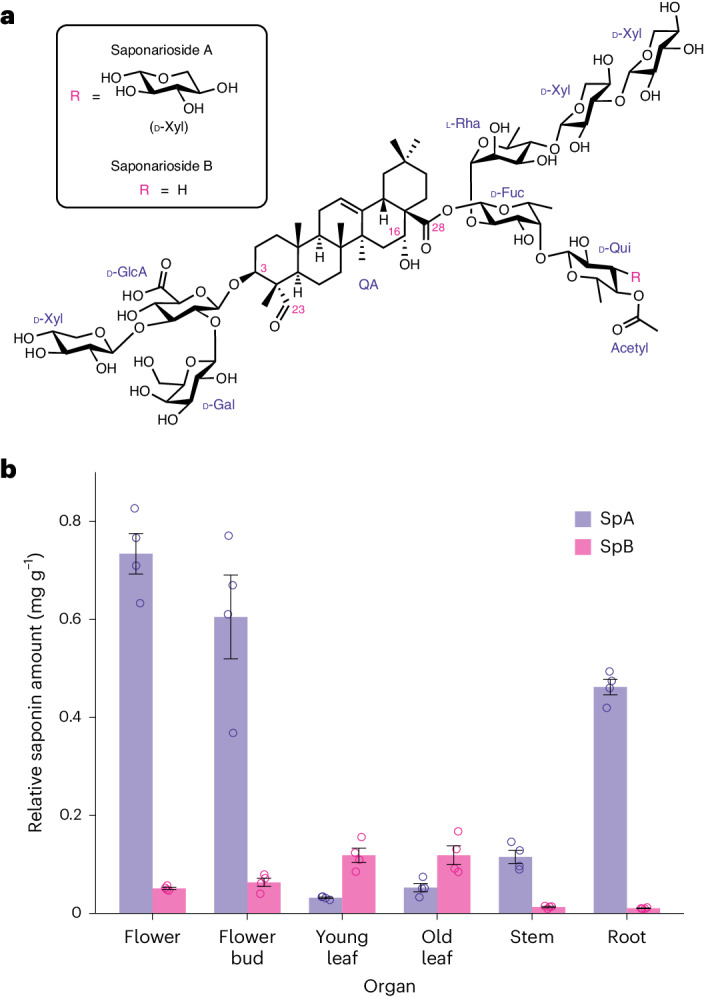
Major saponins found in *S*. *officinalis*: SpA and SpB. **a**, Structures of SpA and SpB, both consisting of a QA aglycone with a branched trisaccharide at C-3 (composed of d-glucuronic acid, d-galactose and d-xylose) and a linear tetrasaccharide at C-28 (composed of d-fucose, l-rhamnose, d-xylose and d-xylose) with an acetylquinovose moiety attached to d-fucose. In SpA, an additional d-xylose is attached to d-quinovose. **b**, Relative abundance of SpA (purple) and SpB (pink). Compounds were identified using authentic standards. Relative abundance was calculated using the internal standard digitoxin, based on dry weight. Each bar represents the mean of four biological replicates and error bars indicate the s.e.m. Source data

Over 40 different saponins have been isolated from soapwort so far^3–9^, some with important pharmaceutical properties including potent anticancer activity^9^. The major saponins found in soapwort are saponariosides A and B (SpA and SpB)^3^ (Fig. 1a). SpA differs from SpB in having an additional sugar (d-xylose) attached to the d-quinovose group. Soapwort saponins have been reported to augment the cytotoxicity of saporin, a type I ribosome-inactivating protein (RIP) found in soapwort^10^. Saporin by itself has low cytotoxicity because, like other type I RIPs, it lacks the natural cell-binding B domain required for entry into the cell^11^. Interestingly, soapwort saponins markedly enhance the cytotoxicity of saporin by initiating endosomal escape of internalized saporins into the cytosol where they exert their toxicity, leading to interest in these compounds as endosomal escape enhancers for targeted tumor therapies^12^. Saporin and its conjugates have also been studied extensively for applications in other prevailing illnesses such as Alzheimer disease, Parkinson disease, insomnia, chronic pain, epilepsy^13,14^ and, more recently, severe acute respiratory syndrome coronavirus 2 (responsible for coronavirus disease 2019, COVID-19)^15^. Most of these studies relied on mixtures of saponins sourced either commercially or as crude plant extracts. However, in one study, a saponin known as SO1861 (also called sapofectosid) was isolated and purified from the roots of soapwort on the basis of its ability to augment saporin toxicity^16^ (Supplementary Fig. 1a).

SpA, SpB, SO1861 and related soapwort saponins are structurally complex molecules with a quillaic acid (QA) scaffold, a branched trisaccharide chain at the C-3 position and a linear tetrasaccharide at the C-28 position (Fig. 1a and Supplementary Fig. 1b). Interestingly, the only other plant genus known to make structurally similar molecules is *Quillaja* (order Fabales), most notably the soapbark tree *Quillaja saponaria* (QS). QS produces saponins that share close structural similarity with saponariosides and are highly valued as vaccine adjuvants, particularly the potent immunostimulant QS-21 (Supplementary Fig. 2), for which the full biosynthetic pathway was recently elucidated^17^. QS-21 is a critical component of human vaccines for shingles, malaria, COVID-19 and the recently approved respiratory syncytial virus (RSV) vaccine Arexvy^18,19^. Mixtures of soapwort saponins have also been observed to form immunostimulating complexes, although no individual soapwort saponins have yet been tested for this property^20^. Despite the considerable interest in the pharmaceutical potential of saponariosides, their biosynthetic pathway is unknown. Recently, preliminary work attempting to elucidate the biosynthetic pathway of saponins produced by a related species (*S*. *vaccaria*) was reported^21^.

Here, we sequence the *S*. *officinalis* genome. Through genome mining, gene coexpression and functional analysis, we elucidate the complete biosynthetic pathway to SpB and reconstitute it in tobacco. We report a total of 14 *S*. *officinalis* genes that together enable saponarioside biosynthesis, including the noncanonical transglycosidase (TG) SoGH1 (glycoside hydrolase family 1), which facilitates the addition of d-quinovose to the C-28 d-fucose moiety of SpB. Although d-quinovose is commonly found in specialized metabolites produced by sea creatures such as starfish and sea cucumbers, it is unusual in plants and its biosynthesis is not understood. Although SpB and the QS saponins are strikingly similar in chemical structure, the enzymes of the saponarioside pathway do not show close amino acid similarity with their counterparts in the QS pathway, with the exception of the first two early pathway steps. Our work opens up broad opportunities for accessing and engineering natural and new-to-nature pharmaceuticals, drug delivery agents and potential immunostimulants with optimized therapeutic properties, inspired by the chemical engineering capabilities of the plant kingdom.

## Results

### Generation of sequence resources for *S*. *officinalis*

**Extended Data Fig. 1:**
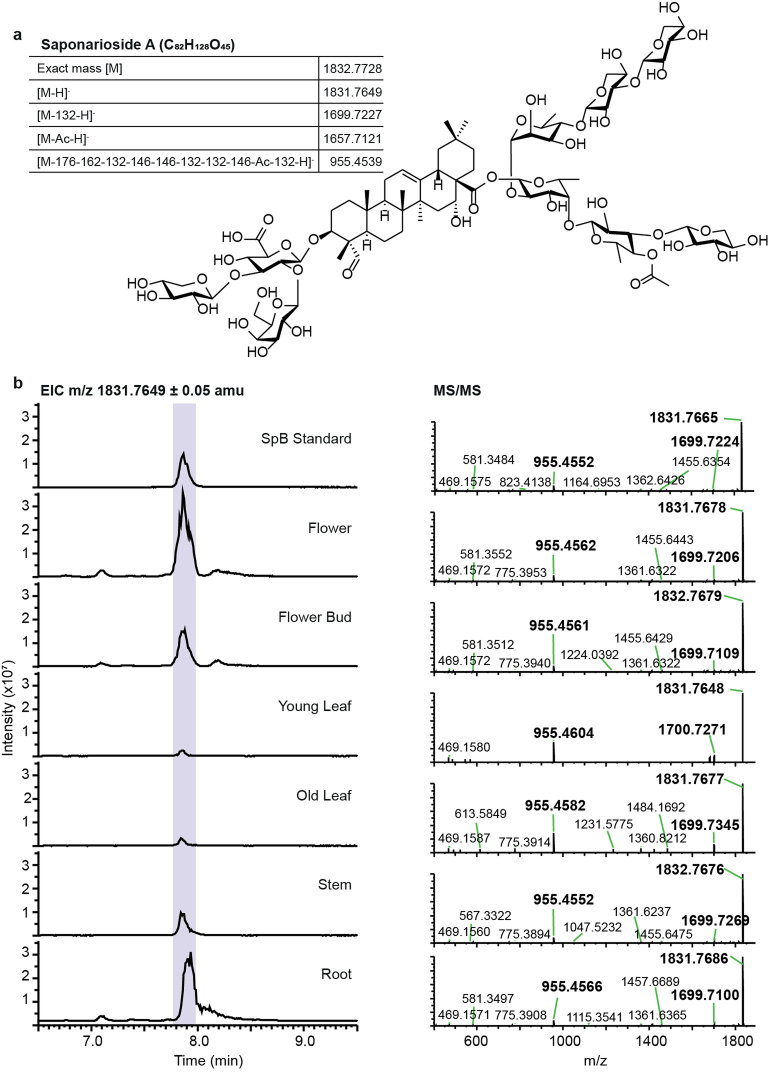
Detection of saponarioside A in extracts of different soapwort organs. The plant extracts were analyzed using HPLC-MS in negative ionization mode. **a**. Structure of saponarioside A with a table showing relevant calculated adducts and fragments. **b**. EIC displayed for *m/z* 1831.7649 (calculated [M-H]^-^ of SpA) and MS/MS spectra of the highlighted peak in corresponding plant samples are shown.

**Extended Data Fig. 2:**
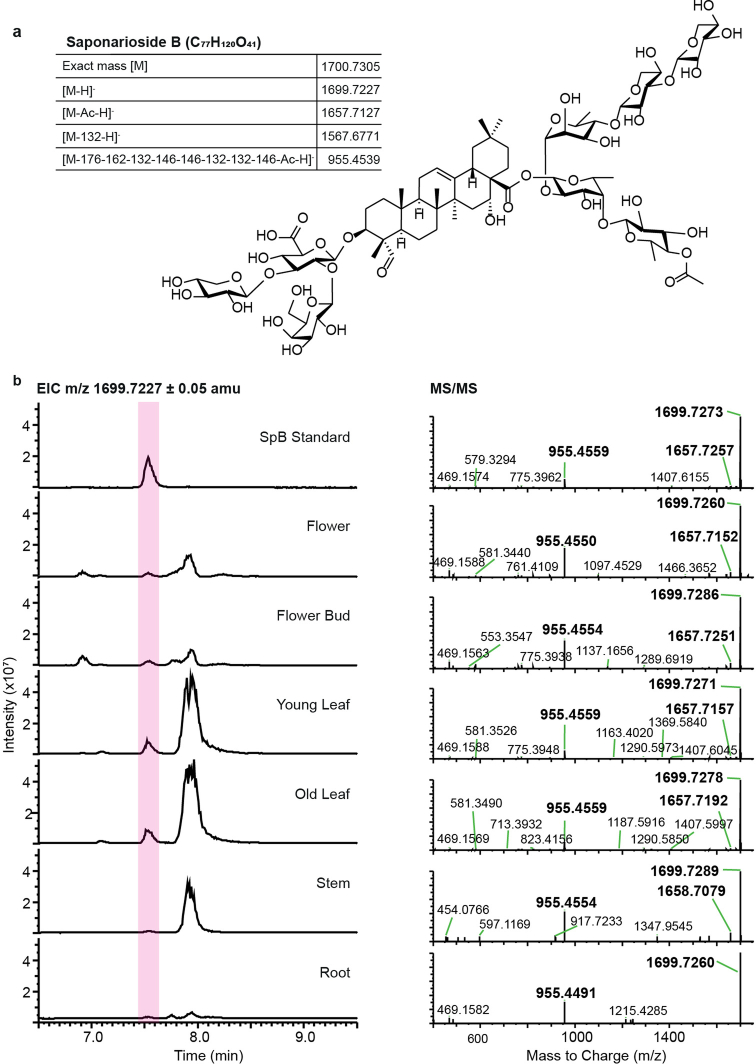
Detection of saponarioside B in extracts of different soapwort organ. The plant extracts were analyzed using HPLC-MS in negative ionization mode. **a**. Structure of saponarioside B with a table showing relevant calculated adducts and fragments. **b**. EIC displayed for *m/z* 1699.7227 (calculated [M-H]^-^ of SpB) and MS/MS spectra of the highlighted peak in corresponding plant samples are shown.

At the start of this work, the only publicly available sequence resource for *S*. *officinalis* was a transcriptome from the 1,000 Plants (1KP) project^22^. This resource is a single dataset derived from pooled plant organs and, thus, was not optimal for the discovery of saponarioside biosynthetic genes. We, therefore, elected to generate our own transcriptome data for *S*. *officinalis*. We first determined the content of SpA and SpB in different *S*. *officinalis* organs. Because commercial standards of these two saponins are not available, we purified SpA and SpB from dried *S*. *officinalis* leaf material and confirmed the structures of the isolated molecules by extensive one-dimensional (1D) and two-dimensional (2D) nuclear magnetic resonance (NMR) (Supplementary Figs. 3–21 and Supplementary Tables 1 and 2). We then carried out targeted high-resolution liquid chromatography–mass spectrometry (HR LC–MS) analysis of extracts from six different *S*. *officinalis* organs (flowers, flower buds, young leaves, old leaves, stem and root; Supplementary Fig. 22). SpA and SpB were identified by comparing the retention times (RTs) and tandem MS (MS/MS) fragmentation patterns with purified standards. Because of the limited availability of purified saponariosides standards, amounts of SpA and SpB in soapwort plants were quantified relative to an internal standard (digitoxin) (Extended Data Figs. 1 and 2). The accumulation patterns of the two saponariosides differed, with SpA being most abundant in the flowers and flower buds and SpB being most abundant in the young and old leaves. The combined levels of both saponins were low in the stems and leaves and highest in the flowers and flower buds (Fig. 1b).

**Extended Data Fig. 3:**
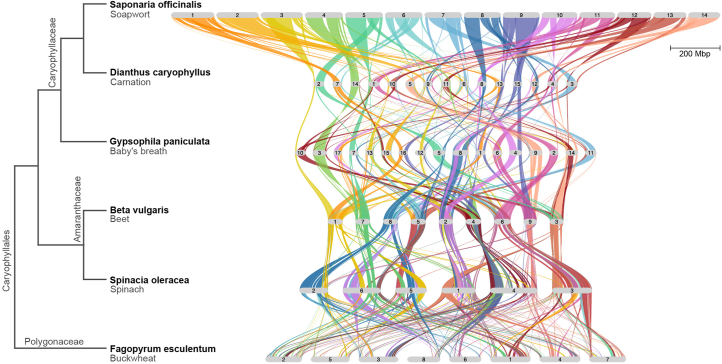
Riparian plot of the newly generated *S. officinalis* genome with genomes of other Caryophyllales species. The chromosomes are drawn to scale (scale bar represents 200 Mbp).

We next performed Illumina paired-end RNA sequencing (RNA-Seq) on RNA from the six different organs (four biological replicates per organ). We also generated a pseudochromosome-level genome assembly of *S*. *officinalis* using PacBio single-molecule real-time circular consensus sequencing (CCS) and high-throughput chromosome conformation capture (Hi-C) sequencing technologies. PacBio long reads were assembled using HiFiasm^23^ and Hi-C data, resulting in 129 scaffolds with an N50 of 148.8 Mb. The largest 14 scaffolds contained 99.46% of the assembled sequences, forming 14 pseudochromosomes (Supplementary Tables 3 and 4). Both the genome size and the predicted chromosome number of *S*. *officinalis* reported here (2.0895 Gb; 1*n* = 14) correspond to values reported using flow cytometry^24,25^. The genome assembly was annotated using the RNA-Seq read alignments generated above and we additionally performed PacBio Iso-Seq CCS to aid in the annotation. Gene models were predicted using homology-based predictors and subjected to Pfam analysis to identify protein families, yielding 37,604 high-confidence protein-coding genes. Genome completeness was assessed using the Benchmarking Universal Single-Copy Orthologs (BUSCO) tool, which determines the presence or absence of highly conserved single-copy genes^26^. The BUSCO analysis revealed that the genome contained 95.2% of expected orthologs as complete single-copy genes, confirming our genome assembly and annotation to be of high quality. Syntenic analysis of the assembled genome was carried out versus other Caryophyllales species and the results showed clear macrosynteny with other species in Caryophyllaceae, as well as in Amaranthaceae (Extended Data Fig. 3).

### Discovery of the biosynthetic genes for QA

**Fig. 2:**
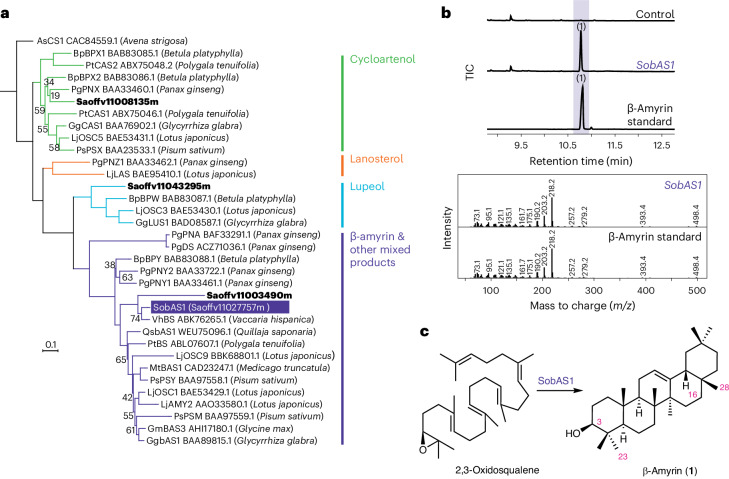
Characterization of SobAS1. **a**, Phylogenetic analysis of candidate *S*. *officinalis* OSCs. The maximum-likelihood tree was generated using an amino acid alignment of putative OSCs in *S*. *officinalis* and previously characterized OSCs from other plant species (listed in Supplementary Table 6). Bootstrap values less than 80% are shown beside each node. The scale bar indicates the number of amino acid substitutions per site. Common enzyme products produced by each clade are labeled on the right. SobAS1, characterized in this work as a β-amyrin (**1**) synthase is highlighted in purple. The three other *S*. *officinalis* OSCs identified in this study are shown in bold. **b**, Transient expression of *SobAS1* in *N*. *benthamiana* leaves. GC–MS total ion chromatograms (TICs) of leaf extracts coexpressing *AstHMGR* and *SobAS1*, along with a control (leaf expressing only *AstHMGR*) and a commercial standard of β-amyrin (**1**), are shown. Mass spectra for leaf extracts expressing *SobAS1* and commercial β-amyrin standard are also given. **c**, Activity of SobAS1 in converting 2,3-oxidosqualene to β-amyrin (**1**).

The first step in triterpene biosynthesis involves the cyclization of the linear precursor 2,3-oxidosqualene to a range of diverse scaffolds by a family of enzymes known as oxidosqualene cyclases (OSCs)^27^. The aglycone core of SpA and SpB is QA, which is derived from one of the most common plant triterpenoid scaffolds, β-amyrin. We, therefore, initiated our search for saponarioside biosynthetic pathway genes by mining the translated *S*. *officinalis* genome for candidate OSCs. This revealed a total of four candidate OSC genes, including one predicted cycloartenol synthase (*Saoffv11008135m*), one predicted lupeol synthase (*Saoffv11043295m*) and two potential β-amyrin synthases (*Saoffv11003490m* and *Saoffv11027757m*) according to phylogenetic analysis (Fig. 2a). *Saoffv11003490m* showed overall low expression in all soapwort tissues compared to *Saoffv11027757m* and the relatively high phylogenetic branch length suggested that this may be a pseudogene or a diverged sequence from one carrying out β-amyrin synthesis (which is found in most higher plants); hence, it was not considered a likely candidate (Supplementary Table 5 and Fig. 2a). Functional analysis of *Saoffv11027757m* by *Agrobacterium*-mediated transient expression in the leaves of *Nicotiana benthamiana* revealed a product with the same gas chromatography (GC)–MS RT and mass spectrum as an authentic β-amyrin standard (**1**), confirming that this enzyme (hereafter named SobAS1) is indeed a β-amyrin synthase (Fig. 2b,c).

**Extended Data Fig. 4:**
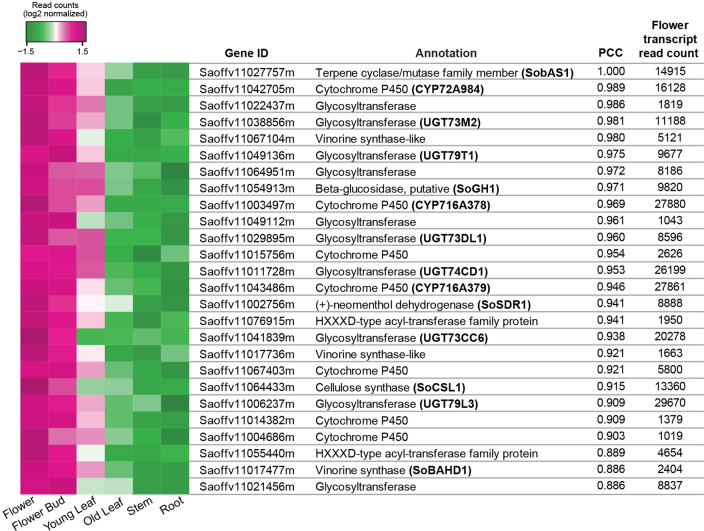
Expression profiles of shortlisted candidate genes. Candidates were filtered by PCC (>0.885) to *SobAS1*, annotation with one of InterPro domains of biosynthetic interest (IPR001128 cytochrome P450; IPR002213 UDP-dependent glycosyltransferase; IPR003480 and IPR001563 acyltransferases) and absolute read count (>1000) in the flower. *SoSDR1* and *SoGH1* has also been included. The heatmap shows library normalized log_2_ read counts scaled by row (gene) and was constructed using Heatmap3^79^. Gene ID, annotation, PCC to *SobAS1* and absolute read count in flower organ (mean, n = 4) are also listed for each candidate. Genes shown in bold are functional saponarioside biosynthetic genes identified and characterized in this study. Full expression data are available as Source Data. Source data

We next performed coexpression analysis across different soapwort organs using *SobAS1* as bait to identify candidate downstream pathway genes. The strength of coexpression was ranked using Pearson’s correlation coefficient (PCC)^28^. Although *SobAS1* showed high expression in all soapwort organs, the highest absolute expression was in the flower, in accordance with our metabolite analysis (Supplementary Table 5 and Fig. 1b). Therefore, we only considered full-length candidates showing high coexpression with *SobAS1* with highest expression in the flower. The resulting list was further filtered by prioritizing candidates annotated with InterPro domains for families of enzymes known to be involved in triterpene biosynthesis, including cytochrome P450s (CYPs; IPR001128), uridine diphosphate (UDP)-dependent glycosyltransferases (UGTs; IPR002213) and acyltransferases (ATs; IPR003480 and IPR001563)^27^ to give the shortlisted candidates shown in Extended Data Fig. 4.

**Fig. 3:**
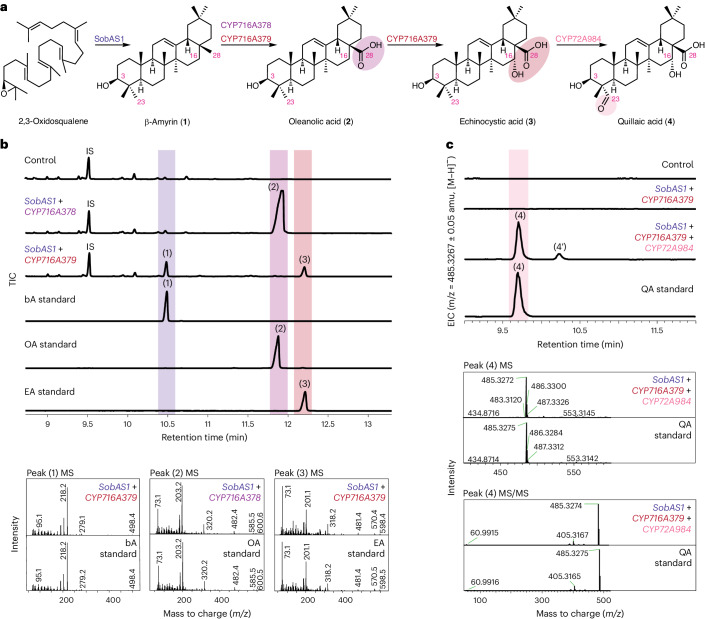
Biosynthesis of QA. **a**, Four *S*. *officinalis* enzymes enable biosynthesis of QA (**4**) in *N*. *benthamiana*. **b**, Products generated by transient expression of *CYP716A378* (C-28 oxidase) and *CYP716A379* (C-28,16α oxidase) in *N*. *benthamiana*. GC–MS TICs of leaf extracts coexpressing *SobAS1* with either *CYP716A378* or *CYP716A379* are shown, along with a control (leaf expressing only *AstHMGR*) and the following commercial standards: bA (**1**, β-amyrin), OA (**2**, oleanolic acid) and EA (**3**, echinocystic acid). Mass spectra of bA (**1**), OA (**2**) and EA (**3**) for leaf extracts expressing *SobAS1* with either *CYP716A378* or *CYP716A379* and for relevant commercial standards are also shown. **c**, Transient expression of *CYP72A984* (C-23 oxidase) in *N*. *benthamiana*. LC–MS extracted ion chromatograms (EICs) of leaf extracts coexpressing *CYP72A984* with the minimal gene set for **3** (*SobAS1* and *CYP716A379*), along with a control (leaf expressing only *AstHMGR*) and a QA (**4**) commercial standard. EICs displayed are at *m*/*z* 485.3267 (calculated [M − H]^−^ of QA (**4**)). MS and MS/MS spectra of QA (**4**) from the commercial standard and leaf extracts coexpressing *SobAS1*, *CYP716A379* and *CYP72A984* are also shown. Formation of another peak (**4′**) putatively identified as gypsogenic acid is also observed when *CYP72A984* is coexpressed with *SobAS1* and *CYP716A379* (MS/MS shown in Supplementary Fig. 25).

The saponarioside scaffold QA (**4**) is a β-amyrin-derived triterpene oxidized at positions C-28, C-16α and C-23 (Fig. 3a). As triterpene scaffolds are commonly oxidized by members of the CYP family^29^, we investigated the functions of the seven candidate CYPs in our shortlist (Extended Data Fig. 4). Each of these CYP candidates was coexpressed with *SobAS1* in *N*. *benthamiana* by transient plant expression and leaf extracts were analyzed by GC–MS and LC–MS to monitor for new product peaks. Our screening implicated three candidate CYPs (encoded by *Saoffv11003497m*, *Saoffv11043486m* and *Saoffv11042705m*) in QA biosynthesis. These were renamed *CYP716A378*, *CYP716A379* and *CYP72A984*, respectively. Transient expression of *CYP716A378* together with *SobAS1* resulted in near-complete conversion of β-amyrin (**1**) to oleanolic acid (**2**) (identified on the basis of a comparison with a commercial standard) (Fig. 3b). When a second candidate, *CYP716A379*, was transiently expressed together with *SobAS1*, we observed the formation of a new peak that we identified as echinocystic acid (**3**) on the basis of a comparison with commercial standards (Fig. 3b and Supplementary Figs. 23 and 24). Coexpression of *CYP72A984* with *SobAS1* and *CYP716A379* resulted in the formation of a new product with an RT, mass and MS/MS fragmentation pattern that matched those of QA (**4**) standard (Fig. 3c). We also observed the production of another peak (**4′**) with a different RT to QA (Fig. 3c, Supplementary Fig. 25). This may be the product of CYP72A984 performing two consecutive C-23 oxidations on residual oleanolic acid resulting in gypsogenic acid, which has the same [M − H]^−^ as QA (Supplementary Figs. 23 and 25). Interestingly, the activity of CYP72A984 also led to accumulation of a product with *m*/*z* 501.3219 ([M − H]^−^ of hydroxylated QA) (Supplementary Fig. 26). This compound may be 16α-hydroxygypsogenic acid (GA_OH_), which is also present in soapwort plants as a saponin backbone^4–6^. Hence, CYP72A984 may also be able to perform further C-23 oxidation on QA to form GA_OH_ (Supplementary Fig. 23). In summary, CYP716A378 is able to introduce a carboxylic acid residue at the C-28 position of β-amyrin (**1**), CYP716A379 is a dual-functioning enzyme that is also able to carry out this modification and, in addition, has C-16α oxidation activity and CYP72A984 performs C-23 oxidation to yield QA (**4**) (Fig. 3a). The phylogenetic relationships of these CYPs with other previously characterized plant CYPs are shown in Supplementary Fig. 27.

### Biosynthesis of the C-3 sugar chain

Having elucidated the steps required for the biosynthesis of QA (**4**), we next focused on the identification of candidate genes for the downstream pathway steps. SpA and SpB both have oligosaccharide chains attached at the C-3 and C-28 positions (Fig. 1a). The presence of a C-3 sugar chain is a common feature of triterpenoid saponins^30^. Additionally, the majority of saponins with a single sugar chain (monodesmosidic saponins) are decorated at the C-3 position of the aglycone rather than the C-28 position^31^. We, therefore, anticipated that the addition of the C-3 sugar chain was likely to occur first, followed by addition of the C-28 sugar chain.

**Fig. 4:**
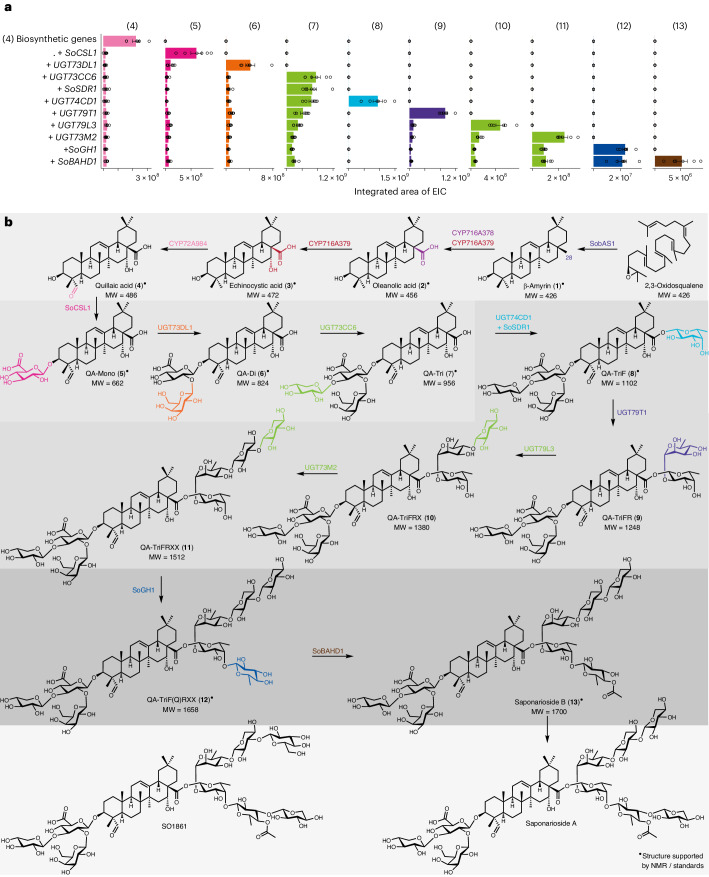
Complete biosynthetic pathway to SpB (13). **a**, Integrated peak areas of EICs for each intermediate accumulating after sequential coexpression of pathway genes in *N*. *benthamiana*, starting with QA (**4**). Each bar represents the mean of six biological replicates and error bars indicate the s.e.m. QA (**4**) biosynthetic genes include *SobAS1*, *CYP716A379* and *CYP72A984*. Data for full characterization of each enzyme are available in the Supplementary Information. **b**, Schematic showing the complete elucidated pathway from 2,3-oxidosqualene to SpB (**13**). The arrows represent the accumulation of metabolite products after each addition of associated enzyme rather than specifying a biosynthetic order in planta. Superscript circles (^●^) indicate structures that are supported by NMR analysis of the purified compound (reported here or in a previous study^35^) or by comparison with an authentic standard. MW, molecular weight. Source data

The C-3 trisaccharide chain of SpA and SpB consists of d-glucuronic acid, d-galactose and d-xylose (Fig. 1a). The sugar that is directly attached to the C-3 position of QA is d-glucuronic acid. UDP-dependent sugar transferases belonging to glycosyltransferase family 1 (GT1) are typically responsible for the glycosylation of plant natural products^32^. However, several cellulose synthase-like (CSL) enzymes have also recently been reported to be involved in the 3-*O*-glucuronidation of triterpene aglycones^33–35^. We observed a predicted CSL hit (*Saoffv11064433m*) that showed high coexpression with *SobAS1* (Extended Data Fig. 4). Phylogenetic analysis of this candidate revealed that *Saoffv11064433m* is a member of the CsyGT/CSLM family, which appears to be a well-conserved subgroup containing 3-*O*-glucuronic acid transferases (Supplementary Fig. 28). This was, therefore, prioritized for functional analysis. This gene was transiently expressed in *N*. *benthamiana* leaves along with the minimal gene set required to produce QA (**4**) (*SobAS1*, *CYP716A379* and *CYP72A984*). LC–MS analysis of leaf extracts revealed a new peak (**5**) with a mass and MS/MS fragmentation pattern corresponding to the authentic 3-*O*-{β-d-glucopyranosiduronic acid}-QA standard (**5**, hereafter abbreviated as QA-Mono) (Supplementary Fig. 29). On the basis of these results, we named this enzyme SoCSL1 (Fig. 4a). We also observed the accumulation of a minor product with *m*/*z* 677.3537 (Supplementary Figs. 23 and 30a). MS/MS analysis of this peak resulted in a loss of 176 (glucuronic acid moiety) from the parent ion with *m*/*z* 501.3231 (calculated [M − H]^−^ of GA_OH_) (Supplementary Fig. 30b). Therefore, in addition to QA (**4**), SoCSL1 may act on GA_OH_ putatively produced by the C-23 oxidation activity of CYP72A984 on **4**. However, compared to the *m*/*z* 677.3537 product peak, *m*/*z* 661.3588 (QA-Mono, **5**) is the major product formed when *SoCSL1* is coexpressed with the QA (**4**) biosynthetic genes (Supplementary Fig. 23). This suggests that SoCSL1 may efficiently convert **4** to **5**, thus pushing the equilibrium toward the production of saponins containing **4** as an aglycone, rather than GA_OH_.

We next screened the ten candidate UGTs in our shortlist of genes that were coexpressed with *SobAS1* (Extended Data Fig. 4) for the ability to elongate the C-3 sugar chain. Each candidate was coexpressed one by one with the gene set needed for biosynthesis of QA-Mono (**5**) (*SobAS1*, *CYP716A379*, *CYP72A984* and *SoCSL1*) and leaf extracts were analyzed by LC–MS. Coexpression of *UGT73DL1* with the QA-Mono biosynthetic genes revealed a new peak (**6**) with a mass ([M − H]^−^ = *m*/*z* 823.4116) consistent with the addition of a hexose to QA-Mono. The RT, mass and fragmentation pattern of this product matched those of an authentic standard of 3-*O*-{β-d-galactopyranosyl-(1 → 2)-β-d-glucopyranosiduronic acid}-QA (**6**, hereafter abbreviated as QA-Di) (Fig. 4, Supplementary Fig. 31). The subsequent coexpression of *UGT73CC6* with *UGT73DL1* and QA-Mono (**5**) biosynthetic genes led to another new product peak (**7**) with a mass ([M − H]^−^ = *m*/*z* 955.4539) corresponding to **6** plus a pentose and an MS/MS fragmentation pattern that matched with a 3-*O*-{β-d-xylopyranosyl-(1 → 3)-[β-d-galactopyranosyl-(1 → 2)]-β-d-glucopyranosiduronic acid}-QA authentic standard (**7**, hereafter abbreviated as QA-Tri) (Fig. 4 and Supplementary Fig. 32). Thus, UGT73DL1 and UGT73CC6 are able to extend the C-3 sugar chain through the addition of a d-galactose and a d-xylose, respectively. These two phylogenetically related UGTs are both located within group D of the GT1 superfamily (Supplementary Fig. 33).

### Biosynthesis of the C-28 sugar chain

We next focused our efforts on elucidation of the steps required for the addition of the main linear C-28 sugar chain of SpB, which is composed of d-fucose linked to a trisaccharide chain consisting of l-rhamnose and two d-xyloses (Fig. 1a). We revisited the remaining eight UGT candidates in our shortlist (Extended Data Fig. 4) and coexpressed each of these in *N*. *benthamiana* leaves with the gene set required for the biosynthesis of QA-Tri (**7**). The first sugar at the C-28 position is d-fucose. Transient coexpression of *UGT74CD1* with the saponarioside biosynthetic genes identified so far resulted in the formation of a product (**8**) with the same RT, mass and MS/MS fragmentation pattern as the authentic standard of 3-*O*-{β-d-xylopyranosyl-(1 → 3)-[β-d-galactopyranosyl-(1 → 2)]-β-d-glucopyranosiduronic acid}-28-*O*-{β-d-fucopyranosyl ester}-QA (**8**, hereafter abbreviated as QA-TriF) and was identified as such (Fig. 4 and Supplementary Fig. 34). However QA-TriF (**8**) accumulated at very low levels and was expected to impede the elucidation of further downstream genes. Poor accumulation of d-fucosylated saponins in *N*. *benthamiana* was also previously observed and suggested to indicate that UDP-α-d-fucose might be limiting^33,35^. We recently showed that this sugar nucleotide is not likely to be relevant for production of the d-fucose moiety found in the structurally related triterpene glycosides from the Chilean soapbark tree^35^. Instead, UDP-4-keto-6-deoxy-glucose, (an intermediate in UDP-l-rhamnose biosynthesis) acts as the sugar donor for transfer of 4-keto-6-deoxy-glucose to the backbone before being reduced in situ to d-fucose by the short-chain dehydrogenase–reductase (SDR) QsFucSyn, which functions as a 4-ketoreductase^35^. During our coexpression analysis we found an SDR candidate (*Saoffv11002756m*) that showed strong coexpression with *SobAS1* (PCC = 0.941) and a high level of absolute expression in the flower organ (Extended Data Fig. 4). The predicted SDR shared 57.2% amino acid sequence identity with QsFucSyn. The transient coexpression of this SDR (renamed *SoSDR1*) with *UGT74CD1* and QA-Tri (**7**) biosynthetic genes led to a significant increase in the production of **8** (Supplementary Fig. 34). Our results suggest that fucosylation of QA-Tri (**7**) may follow the same mechanism as found in soapbark. UGT74CD1 may transfer 4-keto-6-deoxy-glucose to **7**, which is subsequently reduced to d-fucose by the activity of SoSDR1, resulting in the production of QA-TriF (**8**). Next, the additional coexpression of *UGT79T1* with gene set required to produce **8** led to near conversion of **8** to a new product (**9**) with the expected mass of **8** plus a deoxyhexose ([M − H]^−^ = *m*/*z* 1,247.5679) (Fig. 4 and Supplementary Fig. 35). MS/MS analysis of this new product revealed a major fragment ion with mass corresponding to QA-Tri (**7**). This suggested that the addition of deoxyhexose is on the d-fucose moiety of **7**, forming a disaccharide chain that fragments off together ([M − 146 − 146 −H]^−^ = *m*/*z* 955.4539) (Supplementary Fig. 35b). On the basis of our results, we putatively identified this new product as 3-*O*-{β-d-xylopyranosyl-(1 → 3)-[β-d-galactopyranosyl-(1 → 2)]-β-d-glucopyranosiduronic acid}-28-*O*-{α-l-rhamnopyranosyl-(1 → 2)-{β-d-fucopyranosyl ester}-QA (**9**, hereafter abbreviated as QA-TriFR).

Additional rounds of screening led to the discovery of two UGTs with activity toward **9** and the downstream product. The coexpression of *UGT79L3* with the saponarioside biosynthetic genes identified so far resulted in a noticeable depletion of **9** and accumulation of a new product (**10**) with the anticipated mass of **9** plus a pentose ([M − H]^−^ = *m*/*z* 1,379.6119), suggesting the addition of d-xylose and formation of 3-*O*-{β-d-xylopyranosyl-(1 → 3)-[β-d-galactopyranosyl-(1 → 2)]-β-d-glucopyranosiduronic acid}-28-*O*-{β-d-xylopyranosyl-(1 → 4)-α-l-rhamnopyranosyl-(1 → 2)-{β-d-fucopyranosyl ester}-QA (**10**, hereafter abbreviated as QA-TriFRX) (Fig. 4 and Supplementary Fig. 36). The subsequent coexpression of *UGT73M2* together with *UGT79L3* and the set of genes predicted to be required for the biosynthesis of **9** led to the formation of a product (**11**) with a mass ([M − H]^−^ = *m*/*z* 1,511.642) consistent with the addition of a pentose to **10** (Fig. 4 and Supplementary Fig. 37). We anticipated this product to be 3-*O*-{β-d-xylopyranosyl-(1 → 3)-[β-d-galactopyranosyl-(1 → 2)]-β-d-glucopyranosiduronic acid}-28-*O*-{β-d-xylopyranosyl-(1 → 3)-β-d-xylopyranosyl-(1 → 4)-α-l-rhamnopyranosyl-(1 → 2)-{β-d-fucopyranosyl ester}-QA (**11**, hereafter abbreviated as QA-TriFRXX). MS/MS analyses of both **10** and **11** revealed a major fragment ion with mass corresponding to QA-Tri (**7**), suggesting that UGT79L3 and UGT73M2 are both involved in the elongation of the C-28 sugar chain rather than acting upon the aglycone itself (Supplementary Figs. 36b and 37b). On the basis of our results, we putatively identified UGT79L3 as a xylosyltransferase that acts on QA-TriFR (**9**) to produce QA-TriFRX (**10**) and UGT73M2 to be another xylosyltransferase that adds the terminal d-xylose to the main C-28 sugar chain.

The discovery of UGT74CD1, SoSDR1, UGT79T1, UGT79L3 and UGT73M2 completes the set of genes required to produce the main linear part of the C-28 sugar chain present in SpA and SpB. Phylogenetic analysis of these UGTs revealed UGT74CD1 to be a member of GT1 group L, which contains ester-forming GTs, and UGT79T1 and UGT79L3 to be members of GT1 group A, a group known to contain GTs that elongate glycosidic branches^32^ (Supplementary Fig. 33). Together with UGT73DL1 and UGT73CC6, which are involved in the building of the C-3 sugar chain, UGT73M2 grouped within the GT1 group D subfamily UGT73 (Supplementary Fig. 33).

### Addition of d-quinovose by a noncanonical TG

Thus far, we have identified the genes and enzymes that are anticipated to produce QA-TriFRXX (**11**). The missing steps needed to complete the biosynthetic pathway to SpB are those required for the addition of 4-*O*-acetylquinovose to **11**. Although d-quinovose is a common feature of specialized metabolites produced by marine animals such as starfish and sea cucumbers^36^, it is considered unusual as a component of plant metabolites^37^. Consequently, little to none is known about the mechanisms of addition of d-quinovose to plant natural product scaffolds^38^. Although GTs associated with plant natural product biosynthesis typically belong to family 1 of the GT superfamily, none of the UGTs in our candidate shortlist showed quinovosyltransferase activity toward **11**. We noted, however, that a gene predicted to encode a member of a different class of carbohydrate-active enzymes, GH1 transglycosidase (TG), was highly coexpressed (PCC = 0.971) with *SobAS1* (Extended Data Fig. 4). When we expressed this gene (*Saoffv11054913m*) with the other identified saponarioside pathway genes in *N*. *benthamiana*, two new products (**12** and **12′**) with different RTs but the same mass ([M − H]^−^ = *m*/*z* 1,657.7121), corresponding to the expected mass of **11** plus deoxyhexose, were observed (Supplementary Fig. 38). These two products both had the same fragmentation pattern when analyzed by MS/MS. The main fragment ions were *m*/*z* 1,525.6699 and *m*/*z* 955.4539 ([M − H]^−^ of **7**), which suggested a loss of pentose, followed by the loss of the remaining C-28 sugar chain, resulting in **7** (Supplementary Fig. 38b). As the anticipated product, 3-*O*-{β-d-xylopyranosyl-(1 → 3)-[β-d-galactopyranosyl-(1 → 2)]-β-d-glucopyranosiduronic acid}-28-*O*-{β-d-xylopyranosyl-(1 → 3)-β-d-xylopyranosyl-(1 → 4)-α-l-rhamnopyranosyl-(1 → 2)-[β-d-quinovopyranosyl-(1 → 4)]-β-d-fucopyranosyl ester}-QA (hereafter abbreviated as QA-TriF(Q)RXX), is not commercially available, we generated an authentic QA-TriF(Q)RXX standard by purifying the target saponin from extracts of *S*. *officinalis* flowers, followed by extensive 1D and 2D NMR analysis for structural confirmation (Supplementary Figs. 39–49 and Supplementary Table 7). When we compared **12** and **12′** with the authentic QA-TriF(Q)RXX standard, we observed that, although the MS/MS fragmentation of both products matched the QA-TriF(Q)RXX standard, only **12** had the same RT (Supplementary Figs. 38 and 50).

We then carried out large-scale transient expression using 110 *N*. *benthamiana* plants and attempted to purify **12**. Because of its low accumulation, only a crude sample of **12** was obtained even after extensive purification steps. However, 1D and 2D NMR analysis on this rudimentary sample supported the identity of **12** as QA-TriF(Q)RXX (Supplementary Figs. 51–61 and Supplementary Table 8). Taken together, our data suggest that this GH1 TG (which we call SoGH1), is involved in the addition of d-quinovose to d-fucose moiety of QA-TriFRXX (**11**), resulting in the production of QA-TriF(Q)RXX (**12**). Additionally, the matching fragmentation pattern of **12** and **12′** may suggest that these are positional isomers of the terminal d-xylose in the C-28 sugar chain of **12** (Supplementary Fig. 38c). The order of enzyme activity in planta may occur in a complex network and UGT73M2 may transfer d-xylose to d-quinovose after the activity of SoGH1.

**Fig. 5:**
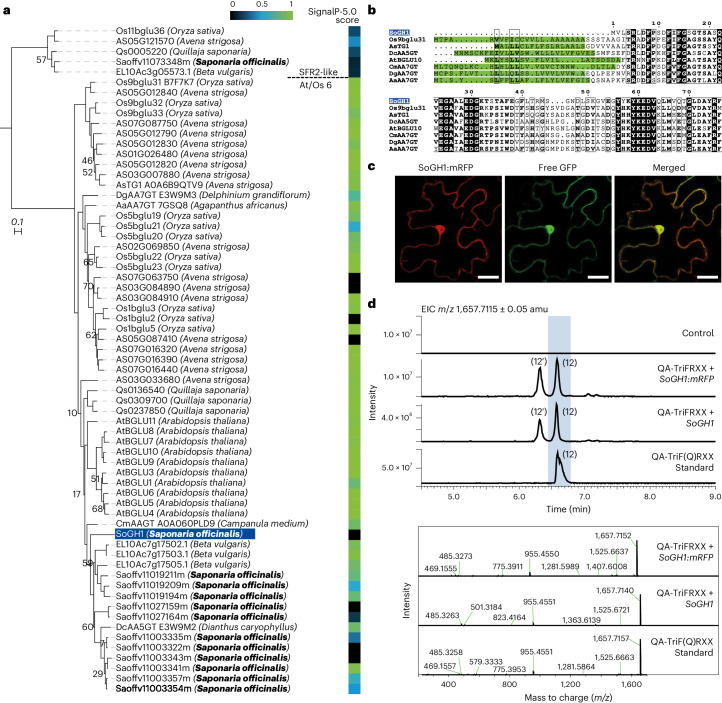
Localization of SoGH1 to the cytosol and nucleus. **a**, Phylogenetic analysis of GH1 enzymes from *S*. *officinalis* and other plant species belonging to the At/Os6 group of the GH1 family. The maximum-likelihood tree (Methods) was generated using an amino acid alignment of putative and characterized (bold) plant GH1 TGs. Bootstrap values less than 80% are shown beside each node. The scale bar indicates the number of amino acid substitutions per site. SFR2 (sensitive to freezing 2)-like enzymes, another subgroup of GH1 family, are used as an outgroup. The side bar to the right shows the SignalP^50^ score for each sequence. **b**, Amino acid sequence alignment (generated using ESPript 3.0)^65^ of the N-terminal regions of all characterized plant GH1 enzymes. Predicted signal peptides are highlighted in green. **c**, Confocal microscopy images of *N*. *benthamiana* leaves transiently coexpressing *SoGH1* tagged with C-terminal mRFP (*SoGH1:mRFP*) and free GFP, both individually and merged. Images were taken 2 days after infiltration. Scale bar, 20 μm. This experiment was performed independently three times with similar results. **d**, Transient expression of *SoGH1:mRFP* in *N*. *benthamiana*. LC–MS EICs of leaf extracts coexpressing the minimal gene set for **11** with either untagged or mRFP-tagged *SoGH1*, along with a control leaf expressing only *AstHMGR* and an authentic QA-TriF(Q)RXX (**12**) standard, are shown. EICs displayed are *m*/*z* 1,657.7115 (calculated [M − H]^−^ of **12**). MS/MS spectra for the leaf extracts and the authentic (**12**) standard are shown at the bottom. The additional peak (**12′**) is putatively identified as a positional isomer of **12** (Supplementary Fig. 38c). Source data

GH1 TGs are an emerging class of sugar transferases with roles in plant specialized metabolism. These enzymes use acyl sugars rather than nucleotide sugars as the sugar donors^39^. The limited number of GH1 TGs characterized so far all transfer glucose^40–46^, with the exception of one galactosyltransferase^47^. Our phylogenetic analysis clustered SoGH1 with the At/Os6 subfamily as designated by Opassiri et al.^48^, which contains most of the previously characterized GH1 TG natural product sugar transferases (Fig. 5a). GH1 enzymes typically have N-terminal signal peptides^48,49^ and all reported GH1 TGs in the At/Os6 subfamily contain signal peptides predicted to target the vacuole^40–47^. Intriguingly, signal sequence analysis by SignalP 5.0 (ref. ^50^) (Fig. 5a) and amino acid alignment of SoGH1 with other characterized members of At/Os6 (Fig. 5b) indicated that SoGH1 lacks an N-terminal leader sequence (Fig. 5b). We next investigated SoGH1 localization by generating C-terminal mRFP (monomeric red fluorescent protein)-tagged SoGH1 recombinant protein (SoGH1:mRFP). Confocal microscopy of *N*. *benthamiana* leaves coinfiltrated with expression constructs for SoGH1:mRFP and free GFP (green fluorescent protein) revealed that SoGH1:mRFP colocalizes with free GFP in the cytosol and nucleus (Fig. 5c), indicating that SoGH1 is a cytosolic protein (the observed nuclear localization is likely because of passive diffusion through the nuclear pores, which is unsurprising for proteins of this size—80 kDa)^51,52^. Metabolite analysis confirmed that the SoGH1:mRFP fusion protein is catalytically active (Fig. 5d). Thus, SoGH1 is a noncanonical GH1 TG that is required for addition of the highly unusual sugar d-quinovose and, unlike the other previously characterized enzymes belonging to the At/Os6 subfamily, does not localize to the vacuole.

**Extended Data Fig. 5:**
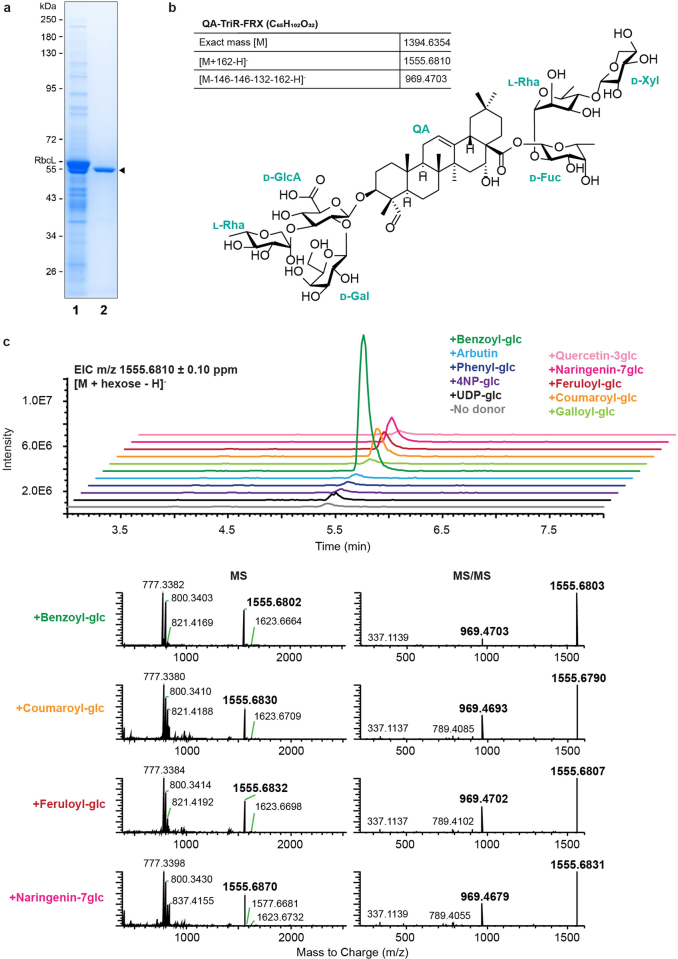
Sugar-donor specificity of SoGH1. **a**. Purified recombinant SoGH1. SoGH1 was expressed with C-terminal His-tag in *N. benthamiana* by *Agrobacterium*-mediated transient expression. Lane 1, cleared lysate before purification. Lane 2, purified fraction by TALON metal affinity purification. RbcL, Rubisco large subunit which is highly abundant in plant leaf soluble extracts. The unprocessed gel image is available as Source Data. **b**. Structure of surrogate saponin acceptor, 3-*O*-{α-l-rhamnopyranosyl-(1 → 2)-[β-d-galactopyranosyl-(1 → 2)]-β-d-glucopyranosiduronic acid}-28-*O*-{β-d-xylopyranosyl-(1 → 4)-α-l-rhamnopyranosyl-(1 → 2)-β-d-fucopyranosyl ester}-quillaic acid (QA-TriR-FRX) used. A table with relevant calculated adducts and modifications is also shown. **c**. Enzyme assay of purified SoGH1 incubated with QA-TriR-FRX and various sugar donors analyzed using HPLC-MS. Extracted ion chromatograms (EIC) and MS/MS spectrum are shown. EIC displayed are for *m/z* 1555.6810, the calculated mass of [M-H]^-^ adduct of QA-TriR-FRX plus hexose. SoGH1 incubated only with QA-TriR-FRX without any sugar donors is used as a negative control (-no donor). A noticeable product peak is observed when benzoyl-glc is given as the sugar donor, but less prominent product peaks are also observed with coumaroyl-glc, feruloyl-glc and naringenin-7glc as sugar donors. MS/MS fragmentation pattern of the product peaks suggests an addition of hexose (d-glucose) to the C-28 sugar chain, which then fragments to *m/z* 969.4703, corresponding to the expected [M-H]^-^ of QA-TriR-FRX without the C-28 sugar chain. This experiment was repeated independently three times with similar results. UDP-glc, UDP-β-d-glucose; 4NP-glc, 4-nitrophenyl-β-d-glucoside; Phenyl-glc, phenyl-β-d-glucoside; arbutin, hydroquinone-β-d-glucoside; benzoyl-glc, benzoyl-β-d-glucoside; galloyl-glc, 1-*O*-galloyl-β-d-glucoside; coumaroyl-glc, 1-*O*-coumaroyl-β-d-glucoside; feruloyl-glc, 1-*O*-feruloyl-β-d-glucoside; naringenin-7glc, naringenin-7-*O*-β-d-glucoside; quercetin-3glc, quercetin-3-*O*-β-d-glucoside. Source data

With the unexpected cytosolic localization of SoGH1, we next set out to determine whether SoGH1 was likely to use acyl sugars as sugar donors. We performed in vitro enzyme assays with His-tagged SoGH1 (Extended Data Fig. 5a). As the surrogate acceptor in our assays, we used QA-TriR-FRX from our previous work on QS saponins^35^ as this was more accessible for purification in quantity than the saponarioside pathway intermediates (Extended Data Fig. 5b). Potential sugar donors of SoGH1 such as UDP-d-quinovose, UDP-4-keto-6-deoxy-d-glucose and their acyl sugar variants are not commercially available and their biosynthetic routes are unknown. Characterized GH1 TGs belonging to At/Os6 subfamily have shown the use of various acyl-glucose donors in vitro, including hydroxybenzoyl^40,42–45^, hydroxycinnamoyl^40–42,44,45^, phenolic^44,46,53^ and flavonoid glucosides^44^, as well as fatty acid-derived glucosides^53^. We, therefore, tested a variety of commercially available potential sugar donors (Extended Data Fig. 5). As expected, SoGH1 showed no activity when UDP-glucose was provided as a sugar donor. However, a prominent product peak with *m*/*z* 1,555.6810 ([M − H]^−^ of QA-TriR-FRX plus a hexose) was observed when SoGH1 was incubated with QA-TriR-FRX and benzoyl-β-d-glucoside (Extended Data Fig. 5c). Although less efficient, SoGH1 also showed glucosylation activity when 1-*O*-coumaroyl-β-d-glucoside, 1-*O*-feruloyl-β-d-glucoside and naringenin-7-*O*-β-d-glucoside were used as sugar donors (Extended Data Fig. 5c). Our data suggest that SoGH1 accepts a wide range of sugar donors, including benzoyl, hydroxycinnamoyl and flavonoid glucosides.

### The complete biosynthetic pathway to SpB

**Extended Data Fig. 6:**
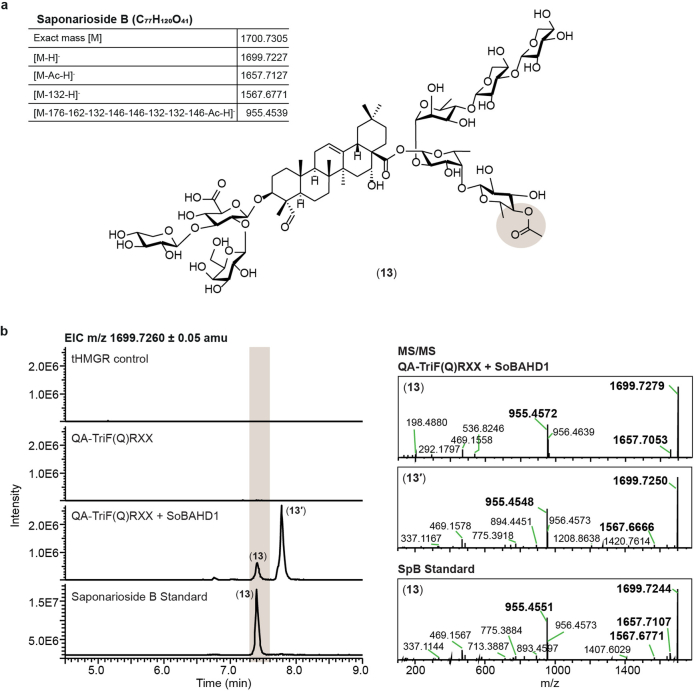
Characterization of SoBAHD1. **a**. Structure of saponarioside B (**13**), a product of SoBAHD1 when acting in combination with the *S. officinalis* enzymes required to produce **12**. Modification performed by SoBAHD1 has been highlighted and a table showing relevant calculated adducts and fragments of **13** included. **b**. *N. benthamiana* leaves transiently co-expressing various genes were extracted and analysed using HPLC-MS, representative (n = 6) extracted ion chromatograms (EIC) and MS/MS spectra are shown. EIC displayed are for *m/z* 1699.7206, the calculated mass of the [M-H]^-^ adduct of **13**. The negative controls used were extracts from *N. benthamiana* leaves co-expressing only *AstHMGR* (tHMGR control) or co-expressing the *S. officinalis* genes required to produce **12** (*tHMGR*, *SobAS1*, *CYP716A379, CYP72A984, SoCSL1, UGT73DL1, UGT73CC6, UGT74CD1, SoSDR1, UGT79T1, UGT79L3, UGT73M2* and *SoGH1*) (QA-TriF(Q)RXX). The additional activity of SoBAHD1 produced two product peaks (**13** and **13′**) which are identified as SpB and SO1699 (see Extended Data Fig. 7) by comparison to authentic standards.

**Extended Data Fig. 7:**
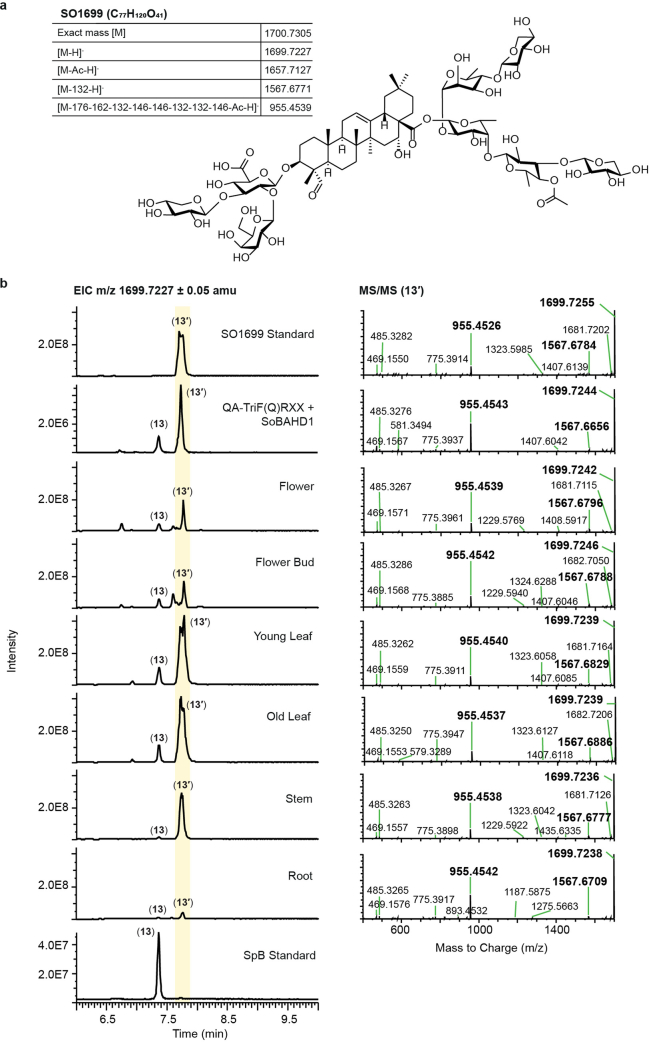
Identification of SO1699 in *N. benthamian* a leaf extracts transiently expressing *S. officinalis* genes and in extracts of different soapwort organs. **a**. Structure of 3-*O*-{β-d-xylopyranosyl-(1 → 3)-[β-d-galactopyranosyl-(1 → 2)]-β-d-glucopyranosiduronic acid}-28-*O*-{β-d-xylopyranosyl-(1 → 4)-α-l-rhamnopyranosyl-(1 → 2)-[β-d-xylopyranosyl-(1 → 3)-β-d-4-*O*-acetylquinovopyranosyl-(1 → 4)]-β-d-fucopyranosyl ester}-quillaic acid (SO1699, **13′**). A table showing relevant calculated adducts and fragments of **13′** is also shown. **b**. EIC at *m/z* 1699.7227 from various samples and respective MS/MS spectra are shown. Peak (**13’**) of same mass, RT, and MS/MS fragmentation pattern is present in *N. benthamiana* leaves samples transiently co-expressing *S. officinalis* genes required produce QA-TriF(Q)RXX (*tHMGR*, *SobAS1*, *CYP716A379, CYP72A984, SoCSL1, UGT73DL1, UGT73CC6, UGT74CD1, SoSDR1, UGT79T1, UGT79L3, UGT73M2, SoGH1*) and SoBAHD1, and as well as all soapwort samples analysed. This peak (**13′**) has different RT compared to SpB (**13**) standard and was identified as SO1699 based on comparison with authentic standard.

With the successful pathway elucidation to **12**, only an acetylation step remained to complete the biosynthetic pathway to SpB (**13**). We, therefore, revisited our short-listed candidates and screened five BAHD-ATs (B, benzyl alcohol *O*-acetyltransferase; A, anthocyanin *O*-hydroxycinnamoyltransferase; H, *N*-hydroxycinnamoyl/benzoyltransferase; D, deacetylvindoline 4-*O*-acetyltransferase) by transient expression in *N*. *benthamiana* (Extended Data Fig. 4). LC–MS analysis of the resulting leaf extracts revealed that coexpression of *SoBAHD1* in combination with the gene set to produce **12** led to the formation of two new products (**13** and **13′**) with the expected mass corresponding to SpB ([M − H]^−^ = *m*/*z* 1,699.7227) (Extended Data Fig. 6). MS/MS analysis revealed that these products had the same fragmentation pattern. The major fragment ions produced were *m*/*z* 1,657.7127 ([M − H]^−^ of **12**) and 955.4539 *m*/*z* ([M − H]^−^ of **7**), suggesting the fragmentation of an acetyl group followed by the loss of the entire C-28 sugar chain (Extended Data Fig. 6b). The two products differed in RT, with the RT of **13** corresponding to that of an authentic SpB standard. On the basis of these results, we identified **13** as SpB, produced by the acetylation of d-quinovose moiety of **12** by SoBAHD1. We noticed that **13′** was also present in our soapwort plant extracts and investigated the identity of **13′** (Extended Data Fig. 7). To confirm the identity of **13′**, we isolated and purified this compound from a commercially available source of *S*. *officinalis* leaf material. Subsequent 1D and 2D NMR resolved the structure of **13′** as 3-*O*-{β-d-xylopyranosyl-(1 → 3)-[β-d-galactopyranosyl-(1 → 2)]-β-d-glucopyranosiduronic acid}-28-*O*-{β-d-xylopyranosyl-(1 → 4)-α-l-rhamnopyranosyl-(1 → 2)-[β-d-xylopyranosyl-(1 → 3)-β-d-4-*O*-acetylquinovopyranosyl-(1 → 4)]-β-d-fucopyranosyl ester}-QA (hereafter abbreviated as SO1699; Supplementary Figs. 62–70 and Supplementary Table 9). This compound was first isolated by Moniusko-Szajw et al.^8^ and may be a direct precursor to SO1861, as SO1699 is lacking only in the terminal d-glucose moiety (Supplementary Fig. 1 and Extended Data Fig. 7a). Phylogenetic analysis of SoBAHD1 together with functionally characterized BAHD ATs from other plant species placed SoBAHD1 in BAHD clade III (Supplementary Fig. 71), a clade that contains BAHD ATs with diverse catalytic functions that are involved in the formation of alkaloids, esters, flavonoids and monoterpenes^54^.

**Fig. 6:**
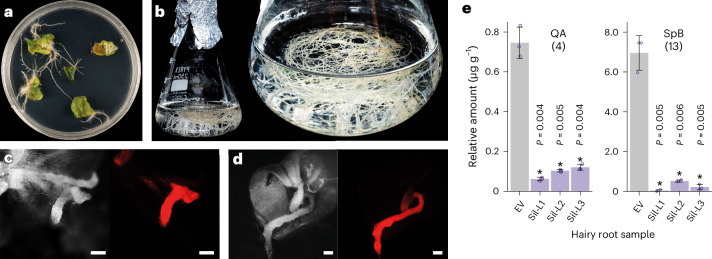
Silencing of *SobAS1* in *S*. *officinalis* hairy roots. **a**,**b**, Photographs showing hairy root induction from leaves of *S*. *officinalis* plantlets (**a**) and 4-week-old hairy roots maintained in liquid medium (**b**). **c**,**d**, Images of transformed hairy roots expressing DsRed fluorescence: empty vector (EV) control (**c**) and representative *SobAS1*-RNAi (RNA interference) line (**d**; left, monochromatic light; right, red fluorescence). Scale bars, 1,000 μm. This experiment was performed independently three times with similar results. **e**, LC–MS analysis of *S*. *officinalis* hairy root extracts from *SobAS1*-RNAi lines (Sil-L1, Sil-L2 and Sil-L3) and EV control. The bar graphs show the relative amounts of QA (**4**) and SpB (**13**) in the different lines. Compounds were identified by comparison with commercial or authentic standards. Relative abundance was calculated using the internal standard digitoxin. Each bar represents the mean of three biological samples and error bars indicate the s.e.m. A two-sided Student’s *t*-test was used to analyze significance (exact *P* values are shown). The expression levels of *SobAS1* in *SobAS1-*RNAi lines are shown in Supplementary Fig. 75. Source data

Following the discovery of the complete biosynthetic route to SpB, we next explored the in planta roles of these genes. Like many plant specialized metabolites, the production of triterpenes can be induced in response to elicitors such as methyl jasmonate (MeJa)^27^. When *S*. *officinalis* plants were treated with 50 μM MeJa, we observed increased expression of all 14 saponarioside biosynthetic genes, most notably in the roots (compared to the leaves and stem) 6 h after MeJa treatment (Supplementary Fig. 72). We also generated hairy root cultures of *S*. *officinalis* and confirmed the presence of QA (**4**) and SpB (**13**) (Fig. 6 and Supplementary Figs. 73 and 74). Silencing of *SobAS1* led to a significant decrease in the levels of both **4** and **13**, confirming the role of SobAS1 in triterpene biosynthesis in *S*. *officinalis* (Fig. 6e and Supplementary Fig. 75).

We also investigated the evolutionary relationship between the genes discovered herein and those in related Caryophyllales (as shown in Extended Data Fig. 3). Many triterpenoid saponins have been isolated from members of the Caryophyllaceae^55^ and Amaranthaceae^56^ families. However, buckwheat (*Fagopyrum esculentum*), a member of the more distant Polygonaceae family within the Caryophyllales, is known to produce nonglycosylated triterpenoids^57^. Although total saponin content has been inferred from seed extracts by ultraviolet absorbance^58^, no isolated saponins have been reported from this species so far^59^. Orthologs of the full SpB pathway were found in the genomes of *Dianthus caryophyllus* and *Gypsophila paniculata* (Caryophyllaceae) and some of the biosynthetic genes (typically those involved in the earlier pathway steps) also had orthologs in *Beta vulgaris* and *Spinacia oleracea* (Amaranthaceae) (Extended Data Table 1 and Supplementary Fig. 76). Interestingly, only orthologs of SoSDR1 and two of the CYPs were found in *F*. *esculentum* (Extended Data Table 1 and Supplementary Fig. 76). Future in-depth genomic and phylogenetic analyses, coupled with the characterization of orthologous enzymes in these species, may shed light on the evolution of saponin biosynthesis in the Caryophyllales order.

## Discussion

Here, we successfully elucidated the complete 14-step biosynthetic pathway for SpB, one of the major saponins found in *S*. *officinalis*. The pathway genes discovered here also enable the biosynthesis of SO1699, a saponin that is structurally related to the pharmaceutically important endosomal escape enhancer SO1861 (ref. ^16^). These advances now open up opportunities to design and produce suites of saponins and analogs in heterologous hosts for the evaluation of properties such as anticancer activity, endosome escape enhancement for targeted tumor therapies and potential immunostimulant activity. Our establishment of *S*. *officinalis* hairy root cultures also offers a platform for qualitative and quantitative manipulation of saponin content within the naturally producing plant.

The enzymes of the saponarioside pathway were characterized de novo in this work and pathway elucidation did not depend on searching for homologs of previously characterized saponin biosynthetic genes from QS but instead relied on the close coexpression of the *S*. *officinalis* saponarioside biosynthetic genes coupled with functional analysis. Saponarioside biosynthesis shows striking structural similarities with QS saponin biosynthesis, the two pathways proceding through essentially the same biosynthetic intermediates up to the last common pathway intermediate QA-TriFRXX (**11**) before diverging. However, with the exception of the first two pathway steps, the corresponding enzymes of the two pathways do not show close amino acid similarity (Extended Data Table 2). *S*. *officinalis* (Caryophyllales) and QS (Fabales) are phylogenetically remote from each other and it seems remarkable that both species are able to make such similar and unusual specialized metabolites. While several of the QS saponin biosynthetic genes are partially clustered in the genome in biosynthetic gene clusters^35^, the saponarioside biosynthetic genes discovered here are scattered across different chromosomes (Supplementary Fig. 77). Although it is tempting to speculate that the two pathways may have arisen by convergent evolution, caution must be exercised in making this assumption because of the challenges with interpreting the ancestral origins of cognate pathway genes given the taxonomic distance between the two species. Regardless, our work offers a distinct set of enzymes for the biosynthesis of QS-like compounds.

**Extended Data Fig. 8:**
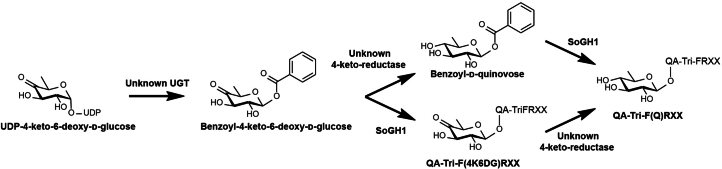
Possible route to quinovoside formation in *S. officinalis.* Formation of d-quinovose *in situ*. UDP-4-keto-6-deoxy-d-glucose exists as an intermediate UDP-l-rhamnose biosynthesis from UDP-d-glucose. UDP-4-keto-6-deoxy-d-glucose could serve as a sugar donor for formation of acyl-4-keto-6-deoxy-d-glucose, which may be the direct sugar donor for SoGH1. The 4-keto group may then be reduced following attachment to the saponin, to form the final d-quinovose in QA-TriF(Q)RXX (**11**). Alternatively, the 4-keto group may be reduced to form acyl-d-quinovose by an unknown 4-ketoreductase which then serves as the sugar donor for SoGH1. Note that the acyl donor component is depicted as benzoic acid for illustrative purposes, but this could be substituted for another suitable acyl group. 4K6DG, 4-keto-6-deoxy-d-glucose.

We further report the discovery of an unusual GH1 TG, SoGH1, which is required for the addition of d-quinovose to the glycosylated saponin scaffold during saponarioside biosynthesis and is likely to use an acyl sugar donor. Unlike other previously characterized GH1 TG enzymes involved in plant natural product glycosylation, this enzyme is localized in the cytosol rather than the vacuole. Although several triterpenoid saponins isolated from the Caryophyllaceae family are known to contain d-quinovose (for example, saponins from *Gypsophila* species^60–62^), the origin of this sugar in plants has so far been elusive. Sugar structural diversity is usually generated at the sugar nucleotide level. For example, thymidine diphosphate (TDP)-d-quinovose is produced by the reduction of TDP-4-keto-6-deoxy-d-glucose in *Streptomyces venezuelae*^63^. However, previous sugar nucleotide profiling of *N*. *benthamiana* reported UDP-rhamnose as the only detectable UDP-deoxyhexose in this plant^35,64^. Given that d-fucose and d-quinovose are C-4 epimers, we hypothesize that the biosynthesis of d-quinovose may be similar to the previous mechanism reported for d-fucose^35^, requiring UDP-4-keto-6-deoxy-d-glucose as a sugar donor before being reduced in situ by a yet unidentified SDR. This reduction could occur once 4-keto-6-deoxy-d-glucose is transferred to the relevant acyl acceptor to form acyl-d-quinovose, which is then used by SoGH1 (Extended Data Fig. 8). Alternatively, this reduction may occur as the terminal step, with acyl-4-keto-6-deoxy-d-glucose serving as the donor for SoGH1 and with reduction of 4-keto-6-deoxy-d-glucose to d-quinovose following attachment to QA-TriFRXX (**11**). Our future research will aim to further resolve quinovosylation and d-quinovose biosynthesis in plants, as well as the mechanism of SoGH1, to shed light on this currently unknown area.

Our investigations of orthologous saponarioside biosynthetic genes in other Caryophyllales species suggest that the biosynthetic pathway for saponarioside-like compounds may have evolved before the emergence of the Caryophyllaceae and is likely to be found across this family. The earlier biosynthetic steps may be common to an even wider range of species, such as in Amaranthaceae. Furthermore, the presence of SoSDR1 orthologs across different families of Caryophyllales, as well as the report of an equivalent gene from soapbark^35^, suggests that this fucosylation mechanism may be found across angiosperms more broadly. These results open up the opportunity to investigate the evolution of saponin biosynthesis across the Caryophyllales.

Collectively, our work paves the way for metabolic engineering of *S*. *officinalis* saponins in heterologous systems, opening up the potential for large-scale production and biochemical studies of these biologically active saponins in the future. The generated sequence resources for *S*. *officinalis* generated in this study could also enable and guide the discovery of biosynthetic routes to other structurally related saponins in the wider Caryophyllaceae family, enabling this reservoir of saponin diversity to be harnessed and engineered for therapeutic applications.

## Methods

### Standards

Standards were obtained from the following sources: oleanolic acid (Merck), echinocystic acid (Extrasynthese), QA (Extrasynthese) and compounds **5**-**8** (Reed et al.)^35^. Compound **9** was previously generated in house (Supplementary Figs. 78–83 and Supplementary Table 10). Internal standards coprostanol (GC–MS) and digitoxin (LC–MS) were obtained from Merck. Methods to generate standards for SpA, SpB (**13**), SO1699 (**13′**) and compound **12** are described in the Supplementary Methods. Compounds used for SoGH1 in vitro assays were obtained from the following sources: 4-nitrophenyl-β-d-glucoside (Merck), benzoyl-β-d-glucoside (Synthose), 1-*O*-coumaroyl-β-d-glucoside (Synthose), 1-*O*-feruloyl-β-d-glucoside (Synthose), hydroquinone-β-d-glucoside (Merck); phenyl-β-d-glucoside (Merck), 1-*O*-galloyl-β-d-glucoside (Synthose), naringenin-7-*O*-β-d-glucoside (A-APIN Chemicals), quercetin-3-*O*-β-d-glucoside (Extrasynthese) and UDP-β-d-glucose (Merck).

### *S*. *officinalis* sampling and maintenance

*S*. *officinalis* plants were obtained from Norfolk Herbs. These plants were maintained in pots and grown in a glasshouse at the John Innes Center. Every December, decayed aboveground organs such as leaves and stems were removed; then, the plants were separated by the rhizomes and individually repotted. Four clonal *S*. *officinalis* plants (named JIC 1, JIC 2, JIC 3 and JIC 4) were harvested in July 2019. Each plant was divided into six different organs, namely, the flowers, flower buds, young leaves, old leaves, stem and root (Supplementary Fig. 22). Harvested plant material was flash-frozen in liquid nitrogen. Frozen plant samples were ground into fine powder using a mortar and pestle with liquid nitrogen and stored at −80 °C until further use.

### Quantification of SpA and SpB in *S*. *officinalis* organs

For metabolite analysis, 1-ml aliquots of frozen ground sample were dried in a freeze-dryer for 2 days. Aliquots (10 mg) of the ground samples were extracted using 1 ml of extraction buffer (80% (v/v) methanol–H_2_O and 10 μg ml^−1^ digitoxin) and incubated at room temperature for 2 h with shaking at 1,400 r.p.m. Following centrifugation at 12,000*g* for 5 min, the supernatants were filtered using 0.2-μm Costar Spin-X microcentrifuge tube filters (Merck). Filtered samples were transferred to Teflon-sealed, screw-capped 2-ml glass vials (Agilent) with glass inserts. LC–MS analysis was performed using a ThermoFisher Q Exactive HPLC system fitted with a Hybrid Quadrupole-Orbitrap MS instrument (ThermoFisher). Samples were analyzed using a Kinetex XB-C18 100A (50 × 2.1 mm, 2.6 μM; Phenomenex) column using a 16.5-min method developed previously^35^. Data were collected with Xcalibur 4.3 and analyzed using FreeStyle 1.6. SpA and SpB were identified by comparison with authentic standards and the relative amounts were quantified using the internal standard digitoxin.

### Genome sequencing and assembly of *S*. *officinalis*

Genome sequencing and assembly was carried out by the HudsonAlpha Institute for Biotechnology. The draft genome was generated by sequencing the genomic DNA (gDNA) extracted using the Qiagen DNeasy kit on Illumina Novaseq. After using the Illumina reads to assess genome complexity and heterozygosity for the *S*. *officinalis* samples, high-molecular-weight gDNA was extracted from JIC 2 leaf samples using a modified CTAB protocol as previously described^35^. To aid in genome annotation, total RNA was extracted from six different *S*. *officinalis* organs from four clonal individuals each (as described above) using Rneasy Plant Mini kit (Qiagen) as previously described^35^. Along with RNA extraction, on-column DNase digestion was performed using RQ1 RNase-free DNase (Promega). RNA-seq and assembly were carried out by the Earlham Institute. The RNA-seq library was prepared using the NEBNext Ultra II Directional RNA-seq library preparation kit and was subsequently sequenced on two lanes of a NovaSeq 6000 SP flow cell (150 paired-end reads).

The genome was assembled with 41.61× (coverage against the haploid genome size) PacBio HiFi reads (mean length = 17,825 bp) using HiFiAsm^23^ and polished with RACON with 59× Illumina 2× 150 paired-end reads. The resulting contigs were oriented, ordered and joined into chromosomes using the JUICER pipeline with 65.5× HiC reads, which indicated no misjoins in the initial assembly. A total of 44 joins were informed from JUICER and applied to the initial assembly to form the final assembly consisting of 14 chromosomes, which contained 99.46% of the assembled sequences. Because of minor residual heterozygosity, five adjacent alternative haplotypes were identified on the joined contig set and collapsed using the longest common substring between the two haplotypes. Chromosomes were numbered largest to smallest, with the p-arm oriented to the 5′ end.

Genome annotation was aided by using Illumina RNA-seq reads using PERTRAN (JGI). PacBio Iso-Seq CCS was performed on the complementary DNA (cDNA) produced from the RNA pool of JIC 2 soapwort plant material and was used to obtain putative full-length transcripts. Gene models were predicted by homology-based predictors and AUGUSTUS^66^. The transcripts were further selected using C-score and a protein basic local alignment search tool (BLASTP) score ratio to the mutual best hit BLASTP score, as well as the protein and expressed sequence tag (EST) coverage. The filtered gene models were subjected to Pfam analysis and models with weak gene models and more than 30% transposable element domains were removed. Gene models with low homology, short single exons without protein domains and low expression were also manually filtered.

### Orthogroup and synteny analysis

Genomes of five species of the Caryophyllales were used for orthogroup and macrosynteny analysis and plotting with the assembled *S*. *officinalis* genome using OrthoFinder^67^ and GENESPACE^68^. These were *D*. *caryophyllus*^69^ (Caryophyllaceae), *G*. *paniculata*^70^ (Caryophyllaceae), *B*. *vulgaris*^71^ (Amaranthaceae), *S*. *oleracea*^72^ (Amaranthaceae) and *F*. *esculentum*^73^ (Polygonaceae). Protein sequence data of the identified orthologs across these species are provided in the Source Data for Extended Data Table 1.

### Phylogenetic analysis

Gene families were mined from target genomes using HMMER^74^ and relevant Pfam domains (OSC, PF13243 and PF13249; CYP, PF00067; BAHD, PF02458; UGT, PF00201; CSL, PF03552; GH1, PF00232) and reference sequences cited the literature where appropriate. Alignments of gene families were carried out using protein sequences in MAFFT^75^ with a maximum of 1,000 iterations. Phylogenetic trees were generated from alignments using RaXML^76^ using the PROTGAMMAAUTO model and 100 bootstraps. Bootstrap values are shown for values < 80%.

### Coexpression analysis and hierarchical clustering

All analyses were performed in R Studio 1.4. Transcript quantification from the de novo transcriptome assembly was used for coexpression. The conversion table of the de novo transcriptome identifier (ID) to genome ID is provided in the Source Data for Extended Data Fig. 4. Salmon quantification results were read in using tximport^77^. Transcripts with read counts of zero in any of the plant organs were removed and the remaining read counts were normalized using DESeq2 (ref. ^78^) by ‘median of ratios’ method. DESeq2 was used to perform log_2_ transformation on the normalized read counts with a pseudo count of one. The resulting read counts were used for coexpression analysis using *SobAS1* as the bait gene. Coexpression analysis was performed using Pearson’s correlation method. The heat map was generated using Heatmap3 (ref. ^79^) by the hierarchical clustering method.

### Gateway cloning

RNA extracted for sequencing was also used for cDNA synthesis. cDNA was generated from 0.8 μg of DNase-treated RNA using GoScriptTM Reverse Transcriptase (Promega) following the manufacturer’s instructions. The treated cDNA was then diluted 1:20 with distilled water and a cDNA pool was produced by combining equal volumes of diluted cDNA from each plant organ. The coding sequences of candidate *S*. *officinalis* genes were PCR-amplified from the cDNA pool using gene specific primers (see Supplementary Data 1), except for *CYP72A984* and *SoGH1*, which were synthesized by Twist Biosciences and Integrated DNA Technologies, respectively. The PCR products were purified using a QIAquick PCR Purification kit following the manufacturer’s protocol. Gateway technology (Invitrogen) was used to transfer the purified PCR products or synthesized gene fragments into the pDONR207 entry vector and subsequently into the pEAQ-HT-DEST1 expression vector^80^.

### Agrotransformation and transient expression in *N*. *benthamiana*

Agrotransformation was performed as previously described^81^ using *Agrobacterium* *tumefaciens* strain LBA4404. Different gene combinations were tested by combining *A*. *tumefaciens* strains carrying the gene of interest before infiltration. All agroinfiltration combinations included the truncated feedback-insensitive *HMGR* gene (*tHMGR*) cloned from *Avena strigosa* to boost triterpene yields^81^. Small-scale hand agroinfiltration, sample harvest and preparation were performed as previously described^81^. Large-scale agroinfiltration of 110 *N*. *benthamiana* plants was performed by vacuum infiltration as previously described^82^. Leaves were harvested 5 days after infiltration and lyophilized, resulting in 90.5 g of dried leaf material. Compound isolation and structural verification by NMR are described in the Supplementary Information.

### Metabolite extraction and analysis of *N*. *benthamiana* leaves

For GC–MS analysis, 10 mg of dried leaf samples were used for extraction. The weighed leaf sample was homogenized with two 3-mm tungsten beads using the Geno/Grinder (SPEX) at 1,000 r.p.m. for 1 min. Ground samples were extracted using 550 μl of ethyl acetate containing 50 μg ml^−1^ coprostanol as the internal standard by agitating intermittently for 20 min at room temperature. After centrifugation at 12,000*g* for 1 min, the supernatants were recovered and transferred into new 2-ml Eppendorf tubes. Samples were then filtered using 0.2-μm Costar Spin-X microcentrifuge tube filters (Merck) and dried using a Genevac EZ-2 evaporator (SP Scientific) before derivatization with 50 μl of 1-(trimethylsilyl)imidazole-pyridine mixture (Sigma-Aldrich). GC–MS analysis was performed on an Agilent 7890B machine fitted with a Zebron AB5-HT Inferno Column (Phenomenex) using a 20-min method program developed previously^35^. Data were collected and analyzed using MassHunter Workstation 10.0. Sample preparation for LC–MS analysis followed the same protocol for the extraction of *S*. *officinalis* plant organs but using 550 μl of extraction buffer instead. LC–MS analysis was carried out as described above for *S*. *officinalis* plant extracts.

### Subcellular localization

To make C-terminal protein fusions with mRFP, *SoGH1* was amplified from pDEST-*SoGH1* plasmids with the primers listed in Supplementary Data 1 and cloned into the pB7RWG2 construct^83^. To express free GFP, the 3xFLAG tag was cloned into the pMDC83 vector^84^. Fluorescent fusion protein constructs were verified by sequencing the full plasmids (Plasmidsaurus). The subcellular localization of fusion constructs was evaluated in *N*. *benthamiana* leaves. Agrotransformation, agroinfiltration and metabolite analysis were performed as described above. Images were taken 2 days after infiltration using a ZEISS LSM880 confocal microscope. The GFP signal was detected with excitation at 488 nm and emission at 498–552 nm, while the RFP signal was detected with excitation at 561 nm and emission at 596–650 nm.

### Preparation of recombinant *SoGH1*

*SoGH1* was expressed with a carboxy-terminal 6xHis-tag in *N*. *benthamiana* using *Agrobacterium*-infiltrated transient expression^81^. The His-tag was added by PCR using the primers listed in Supplementary Data 1 and the amplified fragment was inserted into a unique NruI site of the linearized pEAQ-HT vector^85^ by In-Fusion cloning. The protein was expressed in *N*. *benthamiana* and purified using TALON metal affinity resin as described for UGT74BX1 by Reed et al.^35^.

### In vitro sugar transfer assay

The reaction mixture was composed of 20 mM HEPES pH 7.5, 150 mM NaCl, 0.3% (v/v) 2-mercaptoethanol, 5% (v/v) DMSO, 0.1 mM QA-TriR-FRX (NMR-confirmed sugar acceptor^35^) and 0.5 mM of each sugar donor in a final volume of 50 μl. Sugar donor stock dissolved in 100% DMSO (10 mM) was diluted in the reaction buffer to give 0.5 mM sugar donor and 5% DMSO, respectively. Reactions were initiated by addition of the purified SoGH1 to the reaction mixture and incubated at 25 °C for overnight. After quenching with methanol (final 50%), the filtered reaction mixture (5 μl) was analyzed using LC–MS as described above.

### MeJa treatment

*S*. *officinalis* seeds sourced from Jelitto were germinated at VIB as described in the Supplementary Methods. Then, 2 weeks after germination, plantlets were transferred to hydroponic boxes containing nutrient solution (1/4 Murashige and Skoog medium^86^ with vitamins) and grown under long-day conditions (18 h of light) at 24 °C for 3 months before elicitation and sampling. Elicitation was performed by adding MeJa to the nutrient solution to reach a final concentration of 50 μM. Mock-treated plants were instead administered with the same amount of ethanol used for jasmonate elicitation. Leaves, stems and roots were sampled in triplicate (with each biological replicate being a pool of material derived from three individual plants) 6 and 24 h after treatment. RNA was extracted using the ReliaPrep RNA miniprep system (Promega) following the manufacturer’s instructions for fibrous tissues. RNA-seq was performed as described in the Supplementary Methods.

### Generation of *SobAS1*-silenced hairy roots of *S*. *officinalis*

Primers for gene silencing were designed from unique regions of *SobAS1* (listed in Supplementary Data 1). The resulting gene fragment was cloned into the pDONR207 entry vector and subsequently subcloned into pK7WGIGW-2R (ref. ^83^) using Gateway technology. Control hairy roots were raised using empty pK7WG2R (ref. ^83^). All constructs were transformed into *A*. *rhizogenes* ATCC15834. The leaf explant for hairy root induction was taken from in vitro raised plantlets (see Supplementary Methods for seed germination and in vitro plantlet maintenance). The transformed *A*. *rhizogenes* were grown at 28 °C under continuous shaking (200 r.p.m.) and then pelleted at 25 °C in a centrifuge. A bacterial suspension was prepared for infection, comprising 100 μM acetosyringone in Murashige and Skoog medium and 1% sucrose, attaining an optical density at 600 nm of 0.6. Explants were wounded and injected with bacterial suspension using a needle with ~5 injections per leaf explant. The agro-infected explants were kept for 4 days in cocultivation medium comprising semisolid (0.8% agar) Murashige and Skoog medium supplemented with 3% sucrose and 100 μM acetosyringone in the dark at 25 °C. They were then transferred to semisolid (0.8% agar) Murashige and Skoog medium supplemented with 3% sucrose, 500 mg l^−1^ cefotaxime and 50 mg l^−1^ kanamycin at 25 °C. The transgenic nature of the hairy roots was assessed by dsRED fluorescence after 4 weeks of coincubation using a Zeiss Axio Zoom V16 stereo microscope, with a 43HE dsRED filter (excitation wavelength, 537–562 nm; emission wavelength, 570–640 nm). The positive hairy roots were maintained in liquid B5 (with vitamins and sucrose) in the dark at 25 °C with shaking at 100 r.p.m. and 4-week-old cultures were used for metabolite analysis. Details of metabolite extraction and analysis of hairy root cultures are described in the Supplementary Methods.

### Reporting summary

Further information on research design is available in the Nature Portfolio Reporting Summary linked to this article.

## Online content

Any methods, additional references, Nature Portfolio reporting summaries, source data, extended data, supplementary information, acknowledgements, peer review information; details of author contributions and competing interests; and statements of data and code availability are available at 10.1038/s41589-024-01681-7.

## Supplementary information

Supplementary Information Supplementary Methods, Figs. 1–83, Tables 1–10 and References.

Reporting Summary

Supplementary Data 1 List of primers used in this study, additional details for Supplementary Fig. 1b and source data for Supplementary Fig. 75 and Supplementary Table 4.

## Source data

Source Data Fig. 1 Source data for Fig. 1b.

Source Data Fig. 4 Source data for Fig. 4d.

Source Data Fig. 5 Source data for Fig. 5a.

Source Data Fig. 6 Source data and statistical analysis for Fig. 6e.

Source Data Extended Data Table 1 Source data for Extended Data Table 1.

Source Data Extended Data Fig. 4 Source data for Extended Data Fig. 4.

Source Data Extended Data Fig. 5 Full gel image in Extended Data Fig. 5a.

## Figures and Tables

**Extended Data Table 1:**
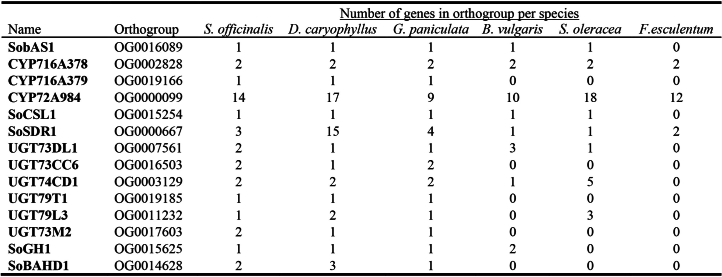
Number of orthologues of saponarioside biosynthetic genes found across Caryophyllales species

Caryophyllales species studied include Saponaria officinalis (S. officinalis), Dianthus caryophyllus (D. caryophyllus), Gypsophila paniculata (G. paniculata), Beta vulgaris (B. vulgaris), Spinacia oleracea (S. oleracea), Fagopyrum esculentum (F. esculentum).

Source data

**Extended Data Table 2:**
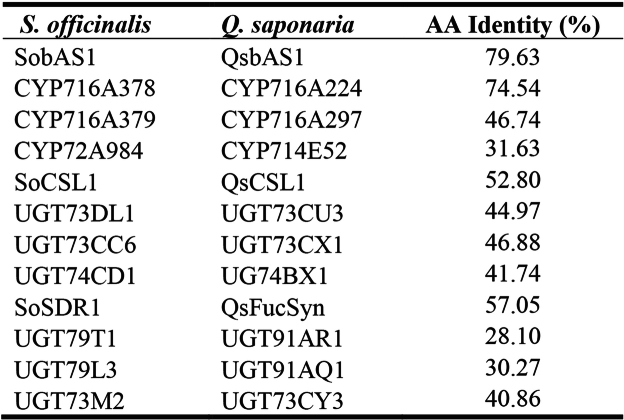
Amino acid (AA) sequence identity between *S. officinalis* and *Q. saponaria* enzymes sharing same biochemical activities

Q. saponaria enzymes were previously identified in^35^.

## Data Availability

The fully assembled and annotated *S*. *officinalis* genome sequence was deposited under BioProject ID PRJNA1018723. The RNA-seq reads were deposited under BioProject IDs PRJNA1008697 and PRJNA1035542. The sequences of the genes characterized in this study can also be found in GenBank as follows: *SobAS1* (OR426407), *CYP716A378* (OR426395), *CYP716A379* (OR426402), *CYP72A984* (OR426401), *SoCSL1* (OR426404), *UGT73DL1* (OR426405), *UGT73CC6* (OR426403), *SoSDR1* (OR426396), *UGT74CD1* (OR426399), *UGT79T1* (OR426408), *UGT79L3* (OR426397), *UGT73M2* (OR426400), *SoGH1* (OR426398) and *SoBAHD1* (OR426406). The InterPro-85.0 (https://www.ebi.ac.uk/interpro/) and Pfam-33.1 (http://pfam.xfam.org/) databases were also consulted. The data that support the findings of this study are available within the main text and the Supplementary Information. Data are also available from the corresponding author upon request. Source data are provided with this paper.
