# Supplementary material for: Unlocking saponin biosynthesis in soapwort
Source: Nat Chem Biol. 2024 Jul 23;21(2):215–26. doi: 10.1038/s41589-024-01681-7 (PMC11782082; doi:10.1038/s41589-024-01681-7)
Supplement: Supplementary file 1 — Supplementary Methods, Figs. 1–83, Tables 1–10 and References. [file 41589_2024_1681_MOESM1_ESM.pdf]

nature chemical biology

Supplementary information

https://doi.org/10.1038/s41589-024-01681-7

Unlocking saponin biosynthesis in soapwort

In the format provided by the authors and unedited

Contents

Supplementary Methods 8

Methyl jasmonate elicitation and RNAseq........ 8

S. officinalis hairy root cultures........ 8

qRT-PCR of *SobAS*-RNAi hairy roots 9

Compound extraction and purification........ 9

Supplementary Figures 13

Supplementary Fig. 1. Complex saponins found in *S. officinalis*. 13

Supplementary Fig. 2. QS-21 isolated from *Quillaja saponaria*. 14

Supplementary Fig. 3. Key HMBC and coupled HSQC coupling constants ($^1J_{H-C}$) recorded for saponarioside A purified from *S. officinalis* leaves. 15

Supplementary Fig. 4. ^{1}H-NMR spectrum of saponarioside A recorded in MeOH-d_4, 600 MHz. 16

Supplementary Fig. 5. ^{1}H-^{1}H-COSY spectrum of saponarioside A recorded in MeOH-d_4, 600 MHz. 17

Supplementary Fig. 6. ^{1}H-^{13}C HSQC spectrum of saponarioside A recorded in MeOH-d_4, 600/150 MHz. 18

Supplementary Fig. 7. ^{1}H-^{13}C coupled HSQC spectrum of saponarioside A recorded in MeOH-d_4, 600/150 MHz. 19

Supplementary Fig. 8. ^{1}H-^{13}C HSQC-TOCSY spectrum of saponarioside A recorded in MeOH-d_4, 600/150 MHz. 20

Supplementary Fig. 9. ^{1}H-^{13}C HMBC spectrum of saponarioside A recorded in MeOH-d_4, 600/150 MHz. 21

Supplementary Fig. 10. DEPTQ-135 spectrum of saponarioside A recorded in MeOH-d_4, 150 MHz. 22

Supplementary Fig. 11. HR-LC-MS (EIC) of saponarioside A. 23

Supplementary Fig. 12. Key HMBC and coupled HSQC coupling constants ($^1J_{H-C}$) recorded for saponarioside B (13) purified from *S. officinalis* leaves. 24

Supplementary Fig. 13. ^{1}H-NMR spectrum of saponarioside B (13) recorded in MeOH-d_4, 600 MHz. 25

Supplementary Fig. 14. ^{1}H-^{1}H COSY spectrum of saponarioside B (13) recorded in MeOH-d_4, 600 MHz. 26

Supplementary Fig. 15. ^{1}H-^{13}C HSQC spectrum of saponarioside B (13) recorded in MeOH-d_4, 600/150 MHz. 27

Supplementary Fig. 16. ^{1}H-^{13}C Coupled HSQC spectrum of saponarioside B (13) recorded in MeOH-d_4, 600/150 MHz. 28

Supplementary Fig. 17. Expanded 1H-13C Coupled HSQC spectrum for (C28-Rhamonsyl motif) of saponarioside B (13) recorded in MeOH-d4, 600/150 MHz. 29

Supplementary Fig. 18. ^{1}H-^{13}C HSQC-TOCSY spectrum of saponarioside B (13) recorded in MeOH-d_4, 600/150 MHz. 30

Suppelmentary Fig. 19. ^{1}H-^{13}C HMBC spectrum of saponarioside B (13) recorded in MeOH-d_4, 600/150 MHz. 31

Supplementary Fig. 20. DEPTQ-135 spectrum of saponarioside B (13) recorded in MeOH-d_4, 150 MHz. 32

Supplementary Fig. 21. HR-LC-ESI-MS (TIC) of saponarioside B (13). 33

Supplementary Fig. 22. Representative image of a soapwort plant harvested in July 2019. 34

Supplementary Fig. 23. Activity of CYP716A378, CYP716A369, CYP72A984 and SoCSL1 35

Supplementary Fig. 24. Production of echinocystic acid by activity of CYP716A379. 36

Supplementary Fig. 25. Production of quillaic acid by activity of CYP72A984. 37

Supplementary Fig. 26. New product peak produced by activity of CYP72A984. 38

Supplementary Fig. 27. Phylogenetic tree of CYP716, CYP714 and CYP72 candidates in soapwort. 39

Supplementary Fig. 28. Phylogenetic tree of CSL candidates in soapwort. 40

Supplementary Fig. 29. Characterization of SoCSL1. 41

Supplementary Fig. 30. New product peak produced by activity of SoCSL. 42

Supplementary Fig. 31. Characterization of UGT73DL1. 43

Supplementary Fig. 32. Characterization of UGT73CC6. 44

Supplementary Fig. 33. Phylogenetic analysis of UGT candidates found in soapwort. 45

Supplementary Fig. 34. Characterization of UGT74CD1 and SoSDR 46

Supplementary Fig. 35. Characterization of UGT79T1 48

Supplementary Fig. 36. Characterization of UGT79L3. 49

Supplementary Fig. 37. Characterization of UGT73M2. 50

Supplementary Fig. 38 Characterization of SoGH1 51

Supplementary Fig. 39. Key HMBC and coupled HSQC coupling constants ($^{1}J_{H\text{-}C}$) recorded for QA-TriF(Q)RXX (12) purified from soapwort flowers. 53

Supplementary Fig. 40. ^{1}HNMR spectrum of QA-TriF(Q)RXX (12) standard material purified from soapwort flowers, recorded in MeOH-d_4, 600 MHz. 54

Supplementary Fig. 41. ^{1}H-^{1}H COSY spectrum of QA-TriF(Q)RXX (12) standard material purified from soapwort flowers, recorded in MeOH-d_4, 600 MHz. 55

Supplementary Fig. 42. ^{1}H-^{13}C HSQC spectrum of QA-TriF(Q)RXX (12) standard material purified from soapwort flowers, recorded in MeOH-d_4, 600/150 MHz. 56

Supplementary Fig. 43. ^{1}H-^{13}C HMBC spectrum of QA-TriF(Q)RXX (12) standard material purified from soapwort flowers, recorded in MeOH-d_4, 600/150 MHz. 57

Supplementary Fig. 44. ^{1}H-^{1}H ROESY spectrum of QA-TriF(Q)RXX (12) standard material purified from soapwort flowers, recorded in MeOH-d_4, 600 MHz. 58

Supplementary Fig. 45. DEPTQ-135 spectrum of QA-TriF(Q)RXX (12) standard material purified from soapwort flowers recorded in MeOH-d_4, 150 MHz. 59

Supplementary Fig. 46. LC-MS overlay between QA-TriF(Q)RXX (12) standard material and one generated in *N. benthamiana*. 60

Supplementary Fig. 47. Parameters for (^{1}H, HSQC and COSY) NMR experiments for compound QA-TriF(Q)RXX (12). 61

Supplementary Fig. 48. Parameters for (coupled HSQC, HMBC and ROESY) NMR experiments for compound QA-TriF(Q)RXX (12). 62

Supplementary Fig. 49. Parameters for (DEPTQ-135) NMR experiments for compound QA-TriF(Q)RXX (12). 63

Supplementary Fig. 50. Extracts of *N. benthamiana* leaves producing compound 12 spiked with authentic QA-TriF(Q)RXX (12) standard. 64

Supplementary Fig. 51. Key HMBC, chemical shifts and coupled HSQC coupling constants ($^{1}J_{H\text{-}C}$) recorded for QA-TriF(Q)RXX (12) produced in *N. benthamiana*. Blue arrows represent H→C. 65

Supplementary Fig. 52. ^{1}H-NMR spectrum of QA-TriF(Q)RXX (12) produced in *N. benthamiana*, recorded in Pyridine-d_5, 600 MHz. 66

Supplementary Fig. 53. Expanded ^{1}H-NMR spectrum (4.7-6.50 ppm) of QA-TriF(Q)RXX (12) produced in *N. benthamiana*, recorded in Pyridine-d_5, 600 MHz. 67

Supplementary Fig. 54. ^{1}H-^{1}H COSY spectrum of QA-TriF(Q)RXX (12) produced in *N. benthamiana*, recorded in Pyridine-d_5, 600 MHz. 68

Supplementary Fig. 55. ^{1}H-^{13}C HSQC spectrum of QA-TriF(Q)RXX (12) produced in *N. benthamiana*, recorded in Pyridine-d_5, 600/150 MHz. 69

Supplementary Fig. 56. ^{1}H-^{13}C coupled HSQC spectrum of QA-TriF(Q)RXX (12) produced in *N. benthamiana*, recorded in Pyridine-d_5 (600/150 MHz). 70

Supplementary Fig. 57. ^{1}H-^{13}C HMBC spectrum of QA-TriF(Q)RXX (12) produced in *N. benthamiana*, recorded in Pyridine-d_5, 600/150 MHz. 71

Supplementary Fig. 58. DEPTQ-135 spectrum of QA-TriF(Q)RXX (12) produced in *N. benthamiana*, recorded in Pyridine-d_5, 150 MHz. 72

Supplementary Fig. 59. HR-LC-ESI-MS (EIC) of QA-TriF(Q)RXX (12) produced in *N. benthamiana*. 73

Supplementary Fig. 60. HR-LC-ESI-MS with predicted molecular formula of QA-TriF(Q)RXX (12) produced in *N. benthamiana*. 74

Supplementary Fig. 61. HR-LC-ESI-MS/MS2 for molecular ion 1657.7106 of QA-TriF(Q)RXX (12) produced in *N. benthamiana*. 75

Supplementary Fig. 62. Key HMBC and coupled HSQC coupling constants ($^{1}J_{H\text{-}C}$) recorded for SO1699 (13′) purified from *S. officinalis* leaves. Red arrows represent H→C. 76

Supplementary Fig. 63. ^{1}H-NMR spectrum of SO1699 (13´) recorded in MeOH-d_4, 600 MHz...77

Supplementary Fig. 64. ^{1}H-^{1}H COSY spectrum of SO1699 (13´) recorded in MeOH-d_4, 600 MHz...78

Supplementary Fig. 65. ^{1}H-^{13}C HSQC spectrum of SO1699 (13´) recorded in MeOH-d_4, 600/150 MHz...79

Supplementary Fig. 66. ^{1}H-^{13}C coupled HSQC spectrum of SO1699 (13´) recorded in MeOH-d_4, 600/150 MHz...80

Supplementary Fig. 67. ^{1}H-^{13}C HSQC-TOCSY spectrum of SO1699 (13´) recorded in MeOH-d_4, 600/150 MHz...81

Supplementary Fig. 68. ^{1}H-^{13}C HMBC spectrum of SO1699 (13´) recorded in MeOH-d_4, 600/150 MHz...82

Supplementary Fig. 69. DEPTQ-135 spectrum of SO1699 (13´) recorded in MeOH-d4, 150 MHz...83

Supplementary Fig. 70. HR-LC-MS (EIC) of SO1699 (13´) with calculated chemical formula $C_{77}H_{119}O_{47}$ [M-H]. 84

Supplementary Fig. 71. Phylogenetic analysis of BAHD acyltranferase candidates found in *S. officinalis*...85

Supplementary Fig. 72. Expression profiles of saponarioside biosynthetic genes induced by methyl jasmonate treatment 86

Supplementary Fig. 73. Detection of quillaic acid in hairy root cultures of *S. officinalis*...87

Supplementary Fig. 74. Detection of saponarioside B in hairy root cultures of *S. officinalis*. 88

Supplementary Fig. 75. Relative expression levels of *SobAS1* in *SobAS1*-RNAi hairy root lines (Sil-1, Sil-2, Sil-3). 89

Supplementary Fig. 76. Orthologues of saponarioside biosynthetic genes found across Caryophyllales species. 90

Supplementary Fig. 77. *S. officinalis* chromosome map showing the physical location of identified saponarioside biosynthetic genes. 91

Supplementary Fig. 78. Key HMBC recorded for QA-Trix-F (8) purified from *Quillaja saponaria* bark extract. Red arrows represent H→C. 92

Supplementary Fig. 79. ^{1}H-NMR spectrum of QA-TriF (8) recorded in MeOH-*d*4 (600 MHz). 93

Supplementary Fig. 80. ^{1}H-^{1}H COSY spectrum of QA-TriF (8) recorded in MeOH-*d*4 (600 MHz). 94

Supplementary Fig. 81. ^{1}H-^{13}C HSQC spectrum of QA-TriF (8) recorded in MeOH-*d*4 (600/150 MHz). 95

Supplementary Fig. 82. ^{1}H-^{13}C HMBC spectrum of QA-TriF (8) recorded in MeOH-*d*4 (600/150 MHz). 96

Supplementary Fig. 83. DEPTQ-135 spectrum of QA-TriF (8) recorded in MeOH-*d*4 (150 MHz). 97

Supplementary Tables 98

Supplementary Table 1. ^{1}H, ^{13}C NMR spectroscopic data recorded for saponarioside A, MeOH-d_4 (600/150 MHz). 98

Supplementary Table 2. ^{1}H, ^{13}C NMR spectroscopic data recorded for saponarioside B (13), MeOH-d_4 (600/150 MHz). 99

Supplementary Table 3. Summary statistics of *S. officinalis* genome assembly. 100

Supplementary Table 4. Summary statistics of the assembled soapwort pseudochromosome. 101

Supplementary Table 5. Transcript read counts of candidate OSCs in different soapwort organs. 102

Supplementary Table 6. Oxidosqualene cylcase sequences and species used for Fig 2A. 103

Supplementary Table 7. ^{1}H, ^{13}C NMR spectroscopic data recorded for QA-TriF(Q)RXX (12) standard material purified from soapwort flowers, MeOH-d_4 (600/150 MHz)..........104

Supplementary Table 8. NMR spectroscopic data [(^{1}H, ^{13}C), anomeric protons] for QA-TriF(Q)RXX (12) produced in *N. benthamiana* compared to literature, both recorded in pyridine-d_5. 105

Supplementary Table 9. ^{1}H, ^{13}C NMR spectroscopic data recorded for SO1699 (13´), MeOH-d_4 (600/150 MHz) 106

Supplementary Table 10. ^{1}H, ^{13}C NMR spectroscopic data recorded for QA-Trix-F (8), MeOH-d_4 (600/150 MHz). 107

Supplementary References 108

Supplementary Methods

Methyl jasmonate elicitation and RNAseq

Seed germination

Seeds were surface sterilized and placed in Magenta boxes with germination medium containing: 2.2 gr L^{-1} MS[1] with vitamins (Duchefa), 1.5 gr L^{-1} sucrose, 0.5 gr L^{-1} MES, 4 gr L^{-1} Phytagel, pH was adjusted with 1 M KOH to 5.8. Boxes were sealed and kept in the dark for 1 week at 4 degrees prior transferring to long day for germination. Seeds were germinated in long day (18 hours of light) under cold fluorescent lamps at 24 °C.

RNA sequencing for expression profiling

RNA concentration and purity were determined spectrophotometrically using the Nanodrop ND-8000 (Nanodrop Technologies) and RNA integrity was assessed using a Bioanalyzer 2100 (Agilent). Per sample, an amount of 500 ng of total RNA was used as input. Using the Illumina TruSeq® Stranded mRNA Sample Prep Kit (protocol version: # 1000000040498 v00 October 2017) poly-A containing mRNA molecules were purified from the total RNA input using poly-T oligo-attached magnetic beads. In a reverse transcription reaction using random primers, RNA was converted into first strand cDNA and subsequently converted into double-stranded cDNA in a second strand cDNA synthesis reaction using DNA Polymerase I and RNAse H. The cDNA fragments were extended with a single 'A' base to the 3' ends of the blunt-ended cDNA fragments after which multiple indexing adapters were ligated introducing different barcodes for each sample. Finally, enrichment PCR was carried out to enrich those DNA fragments that have adapter molecules on both ends and to amplify the amount of DNA in the library. Sequence-libraries of each sample were equimolarly pooled and sequenced on Illumina NovaSeq 6000 (Single Reads, 100 cycles, 1% PhiX) at the VIB Nucleomics Core (www.nucleomics.be).

S. officinalis hairy root cultures

Seed germination and plantlet generation

After washing with sterile water, seeds were kept in sterile water for 3-4 h and surface sterilized in sodium hypochlorite (5% w/v) for 30 min, followed by three times washing with sterile water. In addition, seeds were washed for 1 min in 70% ethanol (v/v), followed by three times washing with sterile water. The seeds were germinated on MS medium (pH 5.88), with 3% sucrose and 0.8% agar. Sub-culturing of plantlets was done after 4 weeks and was

maintained in MS medium (pH 5.88), with 3% sucrose and 0.8% agar at 25 °C with 16 h light photoperiod.

Metabolite extraction and analysis

Harvested hairy roots were freeze dried and 10 mg was each sample was used for metabolite analysis. Samples were ground into fine powder with two 3 mm tungsten beads using GenoGrinder at 1000 rpm for 1 min. Ground samples were extracted using 1 mL of extraction buffer (80% (v/v) MeOH/H_2O, 10 µg/mL digitoxin), shaking at 1200 rpm for 2 h at 25 °C. After, samples were centrifuged and 900 µL of supernatant were recovered which was defatted with 500 µL of hexane. Samples were vortexed and centrifuged to recover 800 µL of bottom aqueous layer and were filtered using 0.2 µm Costar® Spin-X® microcentrifuge tube filters. 600 uL of filtered samples were recovered and evaporated under N_2 gas and samples were resuspended in 100 µL of MeOH which were transferred to 2 mL glass vials with inserts. HPLC-MS analysis followed the sample protocol for analyzing *S. officinalis* plant extracts.

qRT-PCR of *SobAS*-RNAi hairy roots

Primers for qRT-PCR were designed from unique regions using Primer3 software[2] (Supplementary Data 1). The qRT-PCR analysis was performed on a CFX96 Touch Real-Time PCR instrument (Bio-Rad) in the following conditions: initial step in the thermal cycler for 3 min at 95 °C, followed by PCR amplification for 45 cycles of 10 s at 95 °C, 20 s at 60 °C and 20 s at 7 2°C, and finally dissociation analysis to confirm the specificity of PCR products with 0.5 °C ramping from 65 °C to 95 °C. Each 20 µL reaction was comprised of 10 µL Light Cycler 480 SYBR Green I Master mix (Roche Life Science), 2 µL cDNA template (50 ng/µL), 7 µL DNase/RNase free H_2O and 1 µL primer mix (0.5 µM each primer). The $2^{-\Delta\Delta Ct}$ method[3] using was adopted to calculate relative gene expression housekeeping gene actin (*Saoffv11038905m*) as a reference.

Compound extraction and purification

General considerations

Organic solvents used for extraction and flash chromatography were reagent grade and used directly without further distillation. Extraction was performed using a combination of MeOH/H_2O (90/10, and 80/20) under refluxing at 95 °C for two days. HPLC mobile phases

were prepared using HPLC grade solvents. LC-MS spectral data were recorded on either SHIMADZU-2020, single quad, or Q-Exactive orbitrap mass spectrometer using Kinetex-XB-C_{18} (50 × 10 mm i.d.; 2.6 µm), (JIC, UK). 1D and 2D NMR spectra were recorded on Bruker Avance 600 MHz spectrometer equipped with a BBFO Plus Smart probe and a triple resonance TCI cryoprobe, respectively (JIC, UK). The chemical shifts are relative to the residual signal solvent [(MeOH-d_4: δ_H 3.31; δ_C 49.15); (pyridine-d_5 : δ_H 8.74, 7.58, 7.22; δ_C 150.35, 135.91, 123.87)]. Preparative HPLC experiments were performed on Agilent, preparative/semi-preparative HPLC using Luna C_{18} columns (250 x 21.2 and 250 × 10 mm i.d.; 5 µm; USA). All the cure NMR data were collected and processed using MestReNova version 6.0.2-5475 software.

Saponariosides A and B purified from S. officinalis leaves

Commercial dried leaves of *S. officinalis* were purchased from Joannas Garden (Germany, https://joannasgarden.com). For extraction, 17 g of leaf material were extracted under reflux using a combination of methanol/water [80/20, v/v] at 110 °C. The aqueous methanolic extracted was collected and dried under reduced pressure, then re-suspended in the least amount of methanol and complete to 1 L by distilled water. Then it was partitioned against hexane, ethyl acetate, and *n*-butanol. The butanolic layer was collected and completely dried under reduced pressure, then dissolved in the last amount of methanol and immediately saturated with cold acetone, where the crude saponins fraction was precipitated and collected by filtration. A part of this enriched-saponins crude was subjected to purification by preparative HPLC [Luna C_{18} columns (250 x 21.2, i.d.; 5 µm; USA] using a linear gradient of water/acetonitrile [(90/10→10/90), 25 mL/min, over 32 min] acidified with 0.1 formic acid. Promising fractions were collected, dried, and further subjected to reparative semi-preparative HPLC (250 × 10 mm i.d.; 5 µm; USA) using water/acetonitrile [(70/30→20/80, 4 mL/min, over 24 min] acidified with 0.1 formic acid to afford **1.1** and **2.2 mg** of **saponariosides A** (**Purity 60 % based on NMR calculations**) **and B** respectively as white powder material. The identity of the isolated compounds was resolved based on a combination of extensive 1 and 2D NMR spectral data interpretations together with comparison with the literature.

*QA-TriF(Q)RXX (**12**) purified from S. officinalis flowers*

Flowers of *S. officinalis* plants maintained in JIC glasshouse were harvested and freeze-dried. For extraction, 102 g of freeze-dried *S. officinalis* flowers were exhaustively extracted using a combination of MeOH/water (80/20, V/V) (5 x 1L) under refluxing. The aqueous methanolic

crude extract was collected and evaporated under reduced pressure. Then, it was re-dissolved in a combination of MeOH/Water (10/90, total volume 1 L) and directly partitioned against *n*-BuOH (2.5 L). The *n*-butanolic layer was collected and evaporated under educed pressure to afford a greenish viscus material, which was directly resolved over a RP-C18 (120 g) column using a linear gradient of water/acetonitrile [100/0→20/80] (2400 mL), acidified with 0.1 formic acid. 12 sub-fractions (200 mL each) were collected and monitored by LC-MS. Promising fraction 7, was further purified by semi-preparative HPLC (250 × 10 mm i.d.; 5 µm; USA) using a short gradient of water/acetonitrile acidified with 0.1 formic acid [70/30→20/80, 4 mL/min] 4 mL/min over 20 min. Target molecule was collected and lyophilized to afford **6.2 mg** of **QA-TriF(Q)RXX** (**12**) as a white powder. The identity of the purified compound was resolved based on a combination of extensive 1 and 2D NMR spectral data interpretations together with comparison with the literature.

*QA-TriF(Q)RXX (**12**) produced in N. benthamiana*

Large-scale agroinfiltration of 110 *N. benthamiana* plants agroinfiltrated with relevant gene combinations (*AstHMGR*, *SobAS1*, *CYP716A379*, *CYP72A984*, *SoCSL1*, *UGT73DL1*, *UGT73CC6*, *UGT74CD1*, *SoSDR1*, *UGT79T1*, *UGT79L3*, *UGT73M2* and *SoGH1*) was performed by vacuum infiltration as previously described[4]. Leaves were harvested 5 days after infiltration and were lyophilised, resulting in 90.5 g of dried leaf material. Metabolite extraction and purification was performed as previously described[5]. The resulting 90.5 g of dried leaf material was extracted with methanol/water (80/20) and saponins were partitioned from the aqueous methanolic extract using *n*-butanol. Flash column chromatography (FCC) using RP C-18 column (120 g) was performed using a linear gradient of water/acetonitrile acidified with 0.1 formic acid (2400 mL) [100/0→30/70]. A total 12 subfractions (200 mL each) were collected and monitored by HR-LC-ESI-MS, where promising fractions containing peak (**12**) were combined and introduced for further reparative purifications using preparative HPLC via a linear gradient of water/acetonitrile acidified with 0.1 formic acid [100/0→30/70].

*SO1699 (**13'**) purified from S. officinalis leaves*

250 g of *S. officinalis* leaves (purchased from Joannas Garden) were extracted under reflux using a combination of methanol/water [90/10, v/v] at 110 °C. The aqueous methanolic extracted was collected and dried under reduced pressure. A part of this enriched-saponins crude extract was subjected over a Rp-C18 (120 g) flash chromatography using a linear gradient of water/acetonitrile acidified with 0.1 FA [95/5 up to 0/100]. 12 sub-fractions were

collected and monitored by QE-LS-MS. Promising fraction F6 was further purified by preparative HPLC [Luna C18 columns (250 x 21.2, i.d.; 5 µm; USA] using a linear gradient of water/acetonitrile [(70/30→0/100), 25 mL/min, over 30 min] acidified 0.1 formic acid. Promising peak was collected, dried and further subjected to reparative semi-preparative HPLC (250 × 10 mm i.d.; 5 µm; USA) using water/acetonitrile [(70/30→20/80, 4 mL/min, over 24 min] acidified with 0.1 formic acid to afford **1.2 mg** as white powder material. The identity of the isolated compound was resolved based on a combination of extensive 1 and 2D NMR spectral data interpretations together with comparison with the literature.

*QA-TriF (**8**) purified from bark of Quillaja saponaria*

1 g of commercially available Sigma-Aldrich *Quillaja saponaria* bark was solubilized in methanol/water [80/20, V/V] and directly subjected to a Biotage C18-60 g reversed phase flash column chromatography using a linear gradient of [H_2O/ACN + 0.1 % formic acid, (90/10 → 30/70), along 60 minutes, 50 mL/min]. Twelve major sub-fractions **F1-F12** were collected and were monitored by LC-MS. Promising sub-fraction **F8** was introduced for further repetitive purifications using an Agilent semi-preparative HPLC [an isocratic mode, H_2O/ACN + 0.1 % formic acid, (55/45), along 30 minutes, 4 mL/min, (Luna 5 µ C18 (2), 250 x 10 mm)]. The target molecule was collected dried (**2.1 mg**). Based on extensive 1 and 2D-NMR along with HRESI-MS data interpretation, the compound was identified as quillaic acid 3-*O*-{α-L-rhamnopyranosyl-(1→3)-[β-D-galactopyranosyl-(1→2)]-β-D-glucopyranosiduronic acid}-28-*O*-[β-D-fucopyranosyl], abbreviated as QA-TriF (**8**).

Supplementary Figures

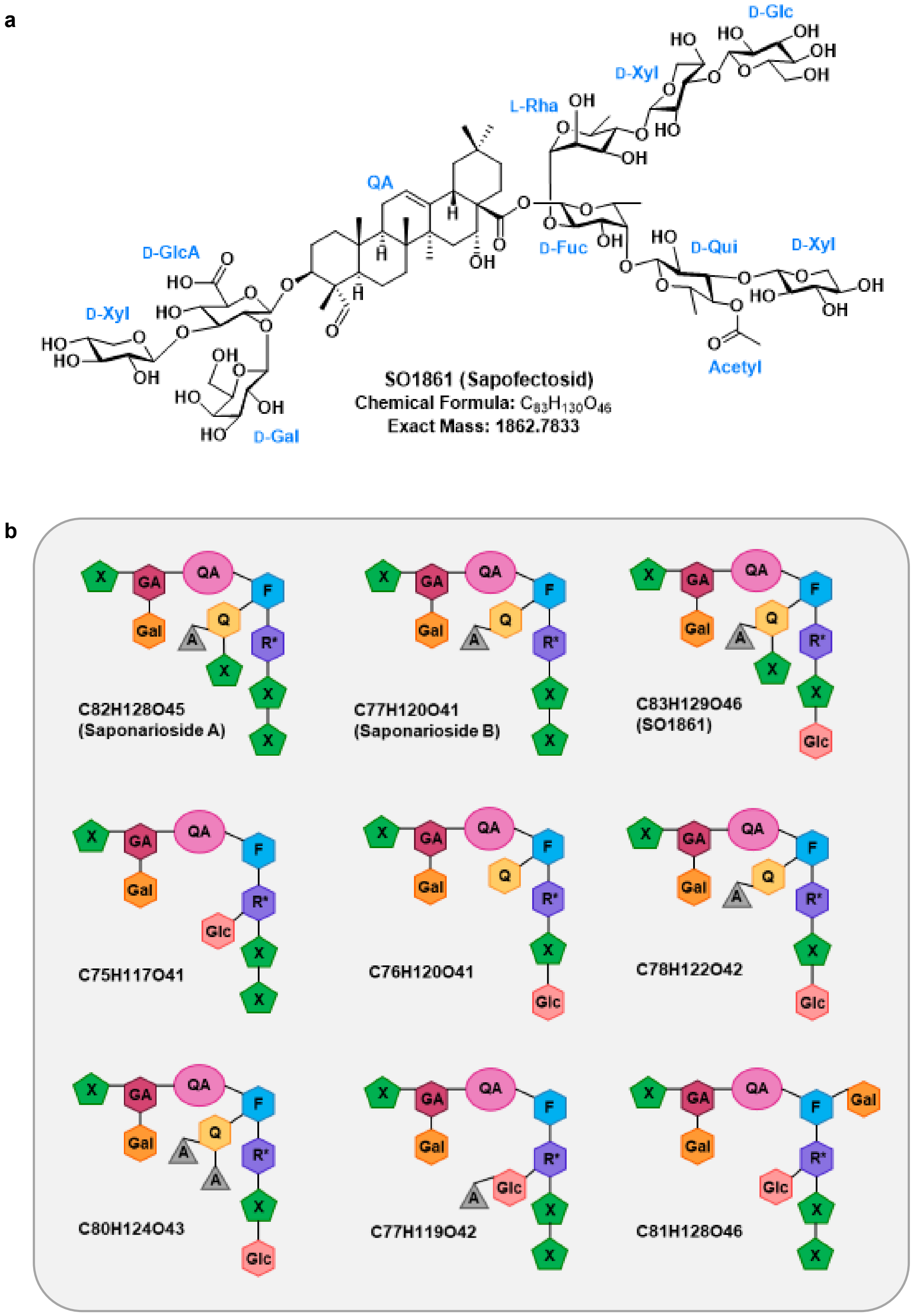

Supplementary Fig. 1. Complex saponins found in *S. officinalis*. a. Chemical structure of SO1861 (sapofectosid). **b.** Schematic drawings of soapwort saponins with similar chemical structures. Chemical details of each saponin can be found in Supplementary Data 1. QA, quillaic acid; D-GlcA (GA), β-D-glucuronic acid; D-Gal (Gal), β-D-galactose; D-Xyl (X), β-D-xylose; D-Fuc (F), β-D-fucose; D-Qui (Q), β-D-quinovose; L-Rha (R*), α-L-rhamnose; D-Glc (Glc), β-D-glucose.

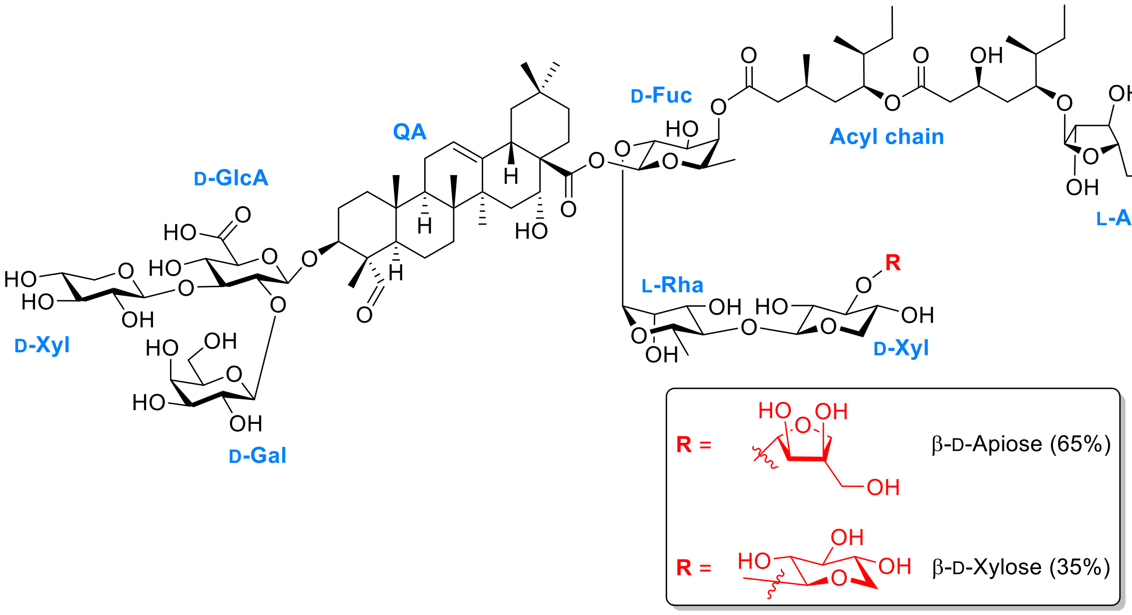

Supplementary Fig. 2. QS-21 isolated from *Quillaja saponaria*. Variants of QS-21 with L-Rha instead of D-Xyl at C-3 also exist. QA, quillaic acid; D-GlcA, β-D-glucuronic acid; D-Gal, β-D-galactose; D-Xyl, β-D-xylose; D-Fuc, β-D-fucose; L-Rha, α-L-rhamnose; L-Ara, α-L-arabinofuranose.

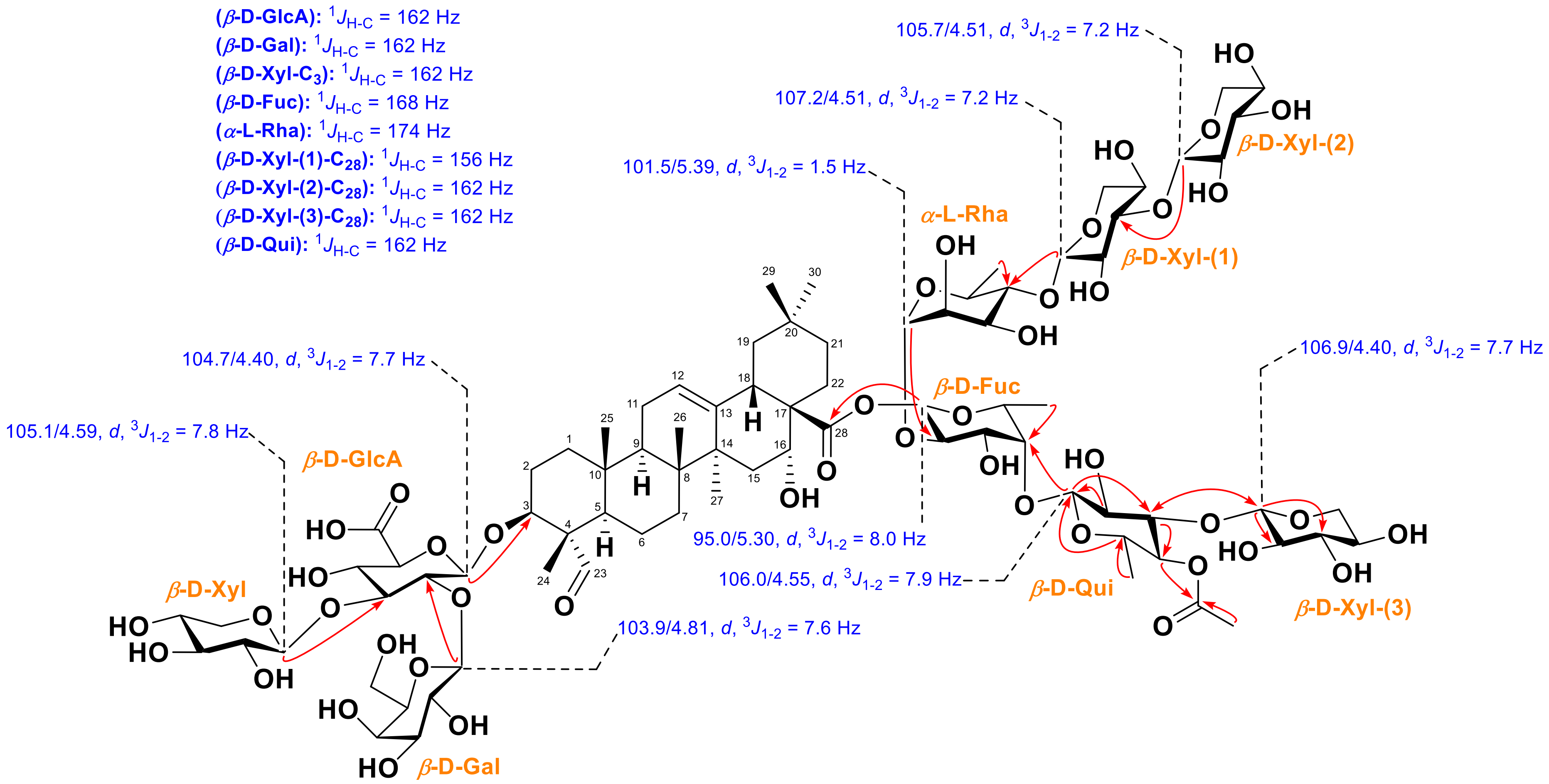

Supplementary Fig. 3. Key HMBC and coupled HSQC coupling constants ($^{1}J_{H\text{-}C}$) recorded for saponarioside A purified from *S. officinalis* leaves. Red arrows represent H→C.

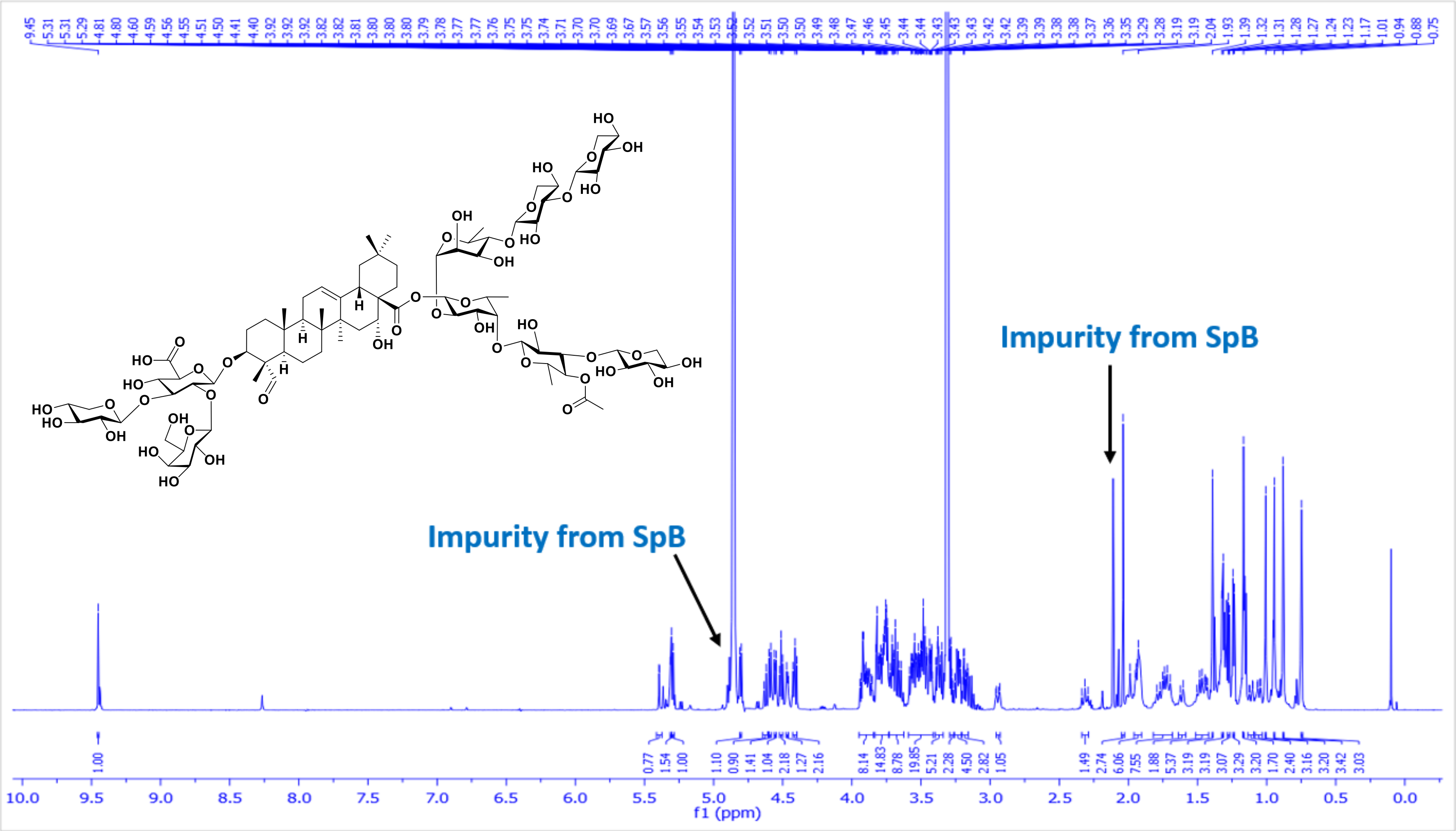

Supplementary Fig. 4. **^{1}H-NMR spectrum of saponarioside A recorded in MeOH-*d*$_4$, 600 MHz.**

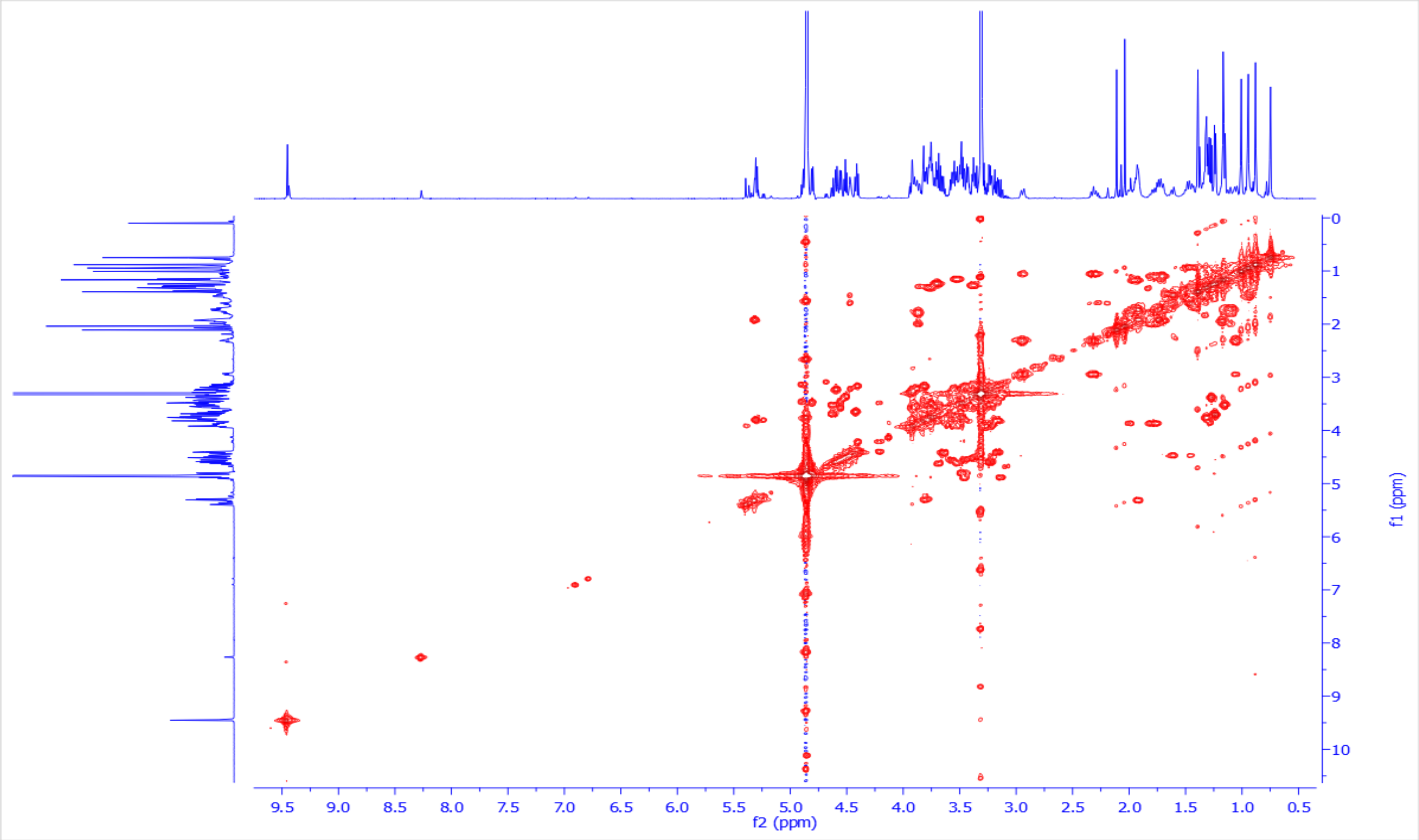

Supplementary Fig. 5. **^{1}H-^{1}H-COSY spectrum of saponarioside A recorded in MeOH-*d*₄, 600 MHz.**

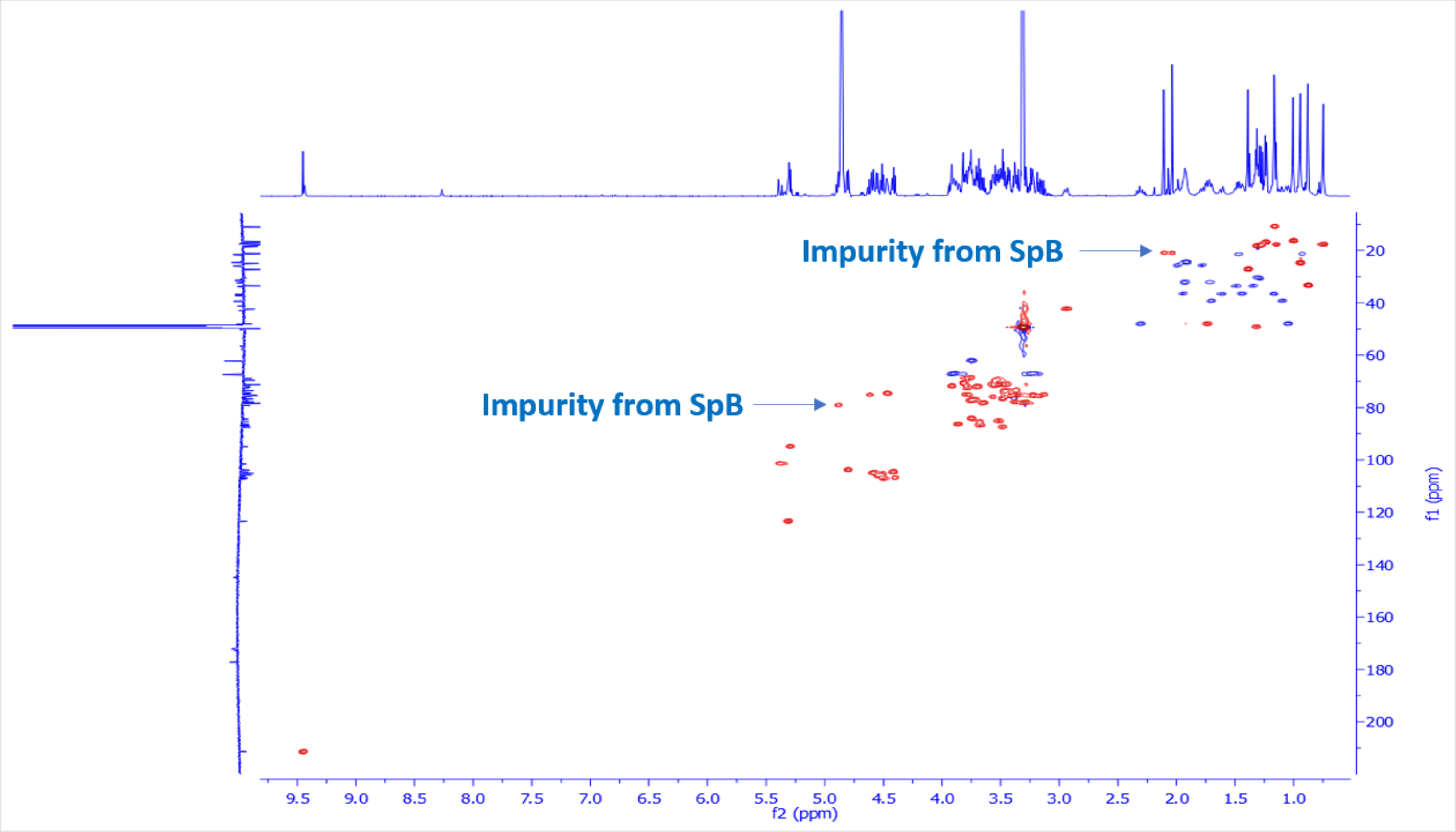

Supplementary Fig. 6. ^{1}H-^{13}C **HSQC spectrum of saponarioside A recorded in MeOH-*d*4, 600/150 MHz.**

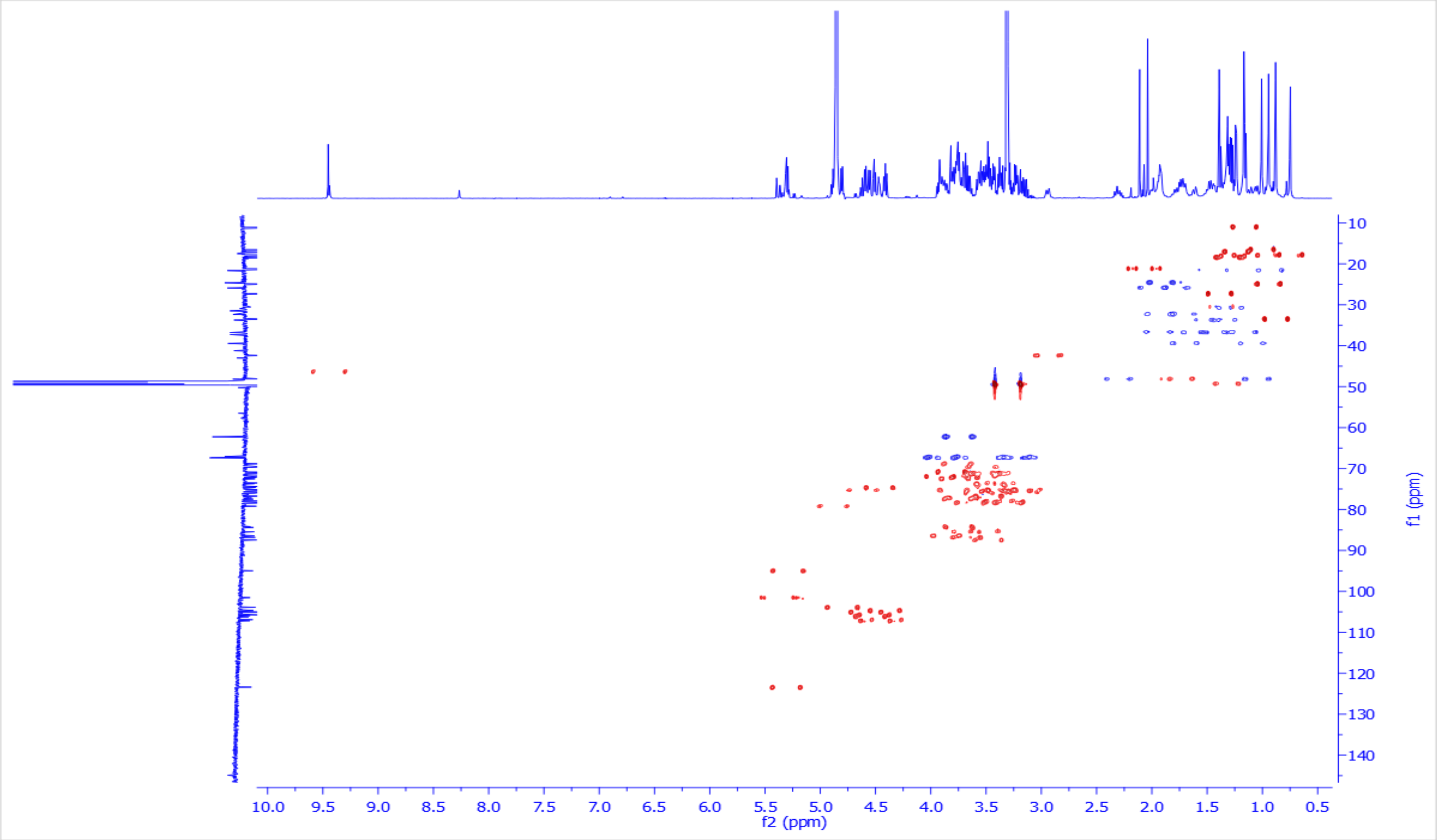

Supplementary Fig. 7. ^{1}H-^{13}C coupled HSQC spectrum of saponarioside A recorded in MeOH-*d*4, 600/150 MHz.

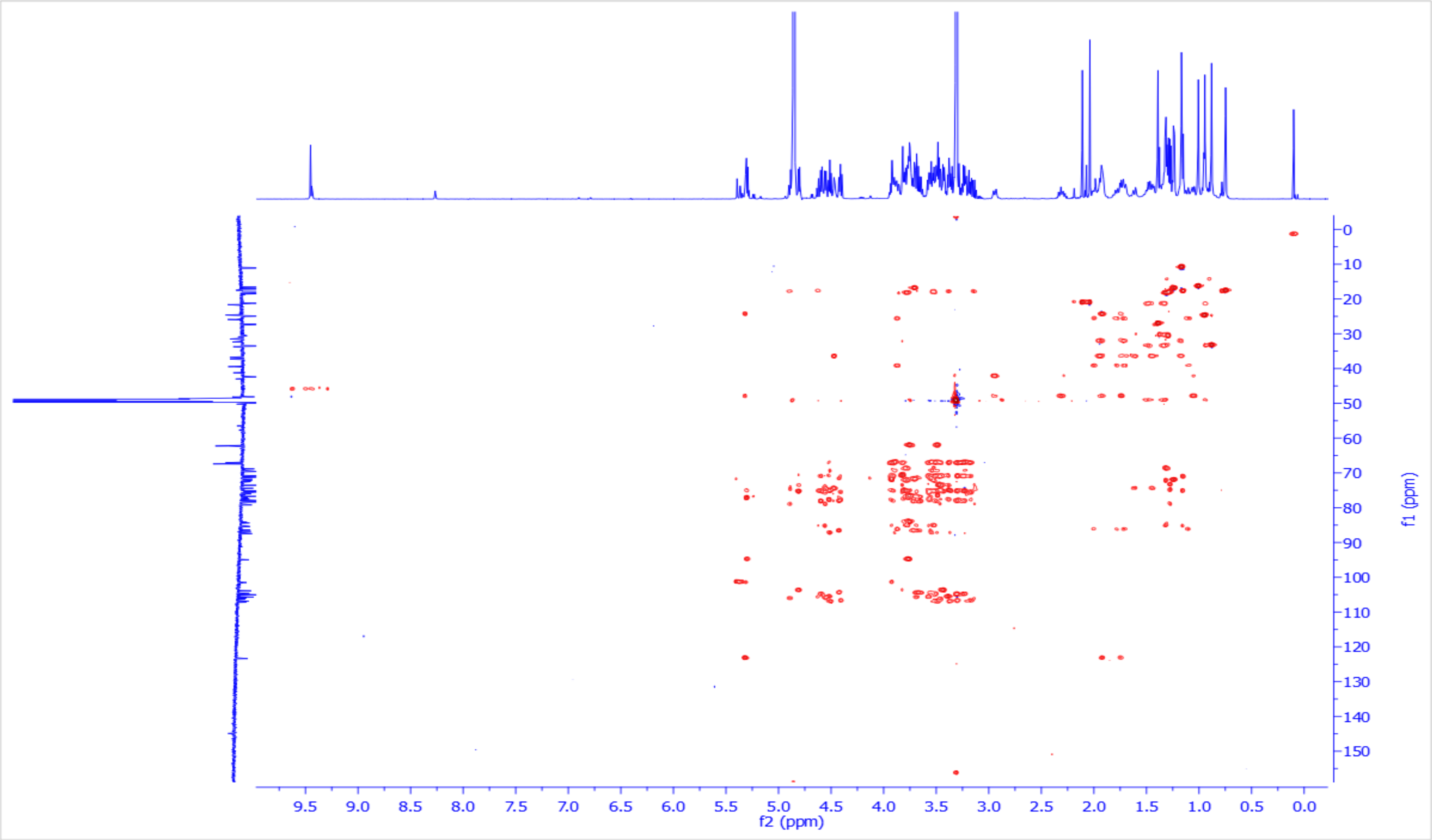

Supplementary Fig. 8. ^{1}**H-**^{13}**C HSQC-TOCSY spectrum of saponarioside A recorded in MeOH-*d*$_4$, 600/150 MHz.**

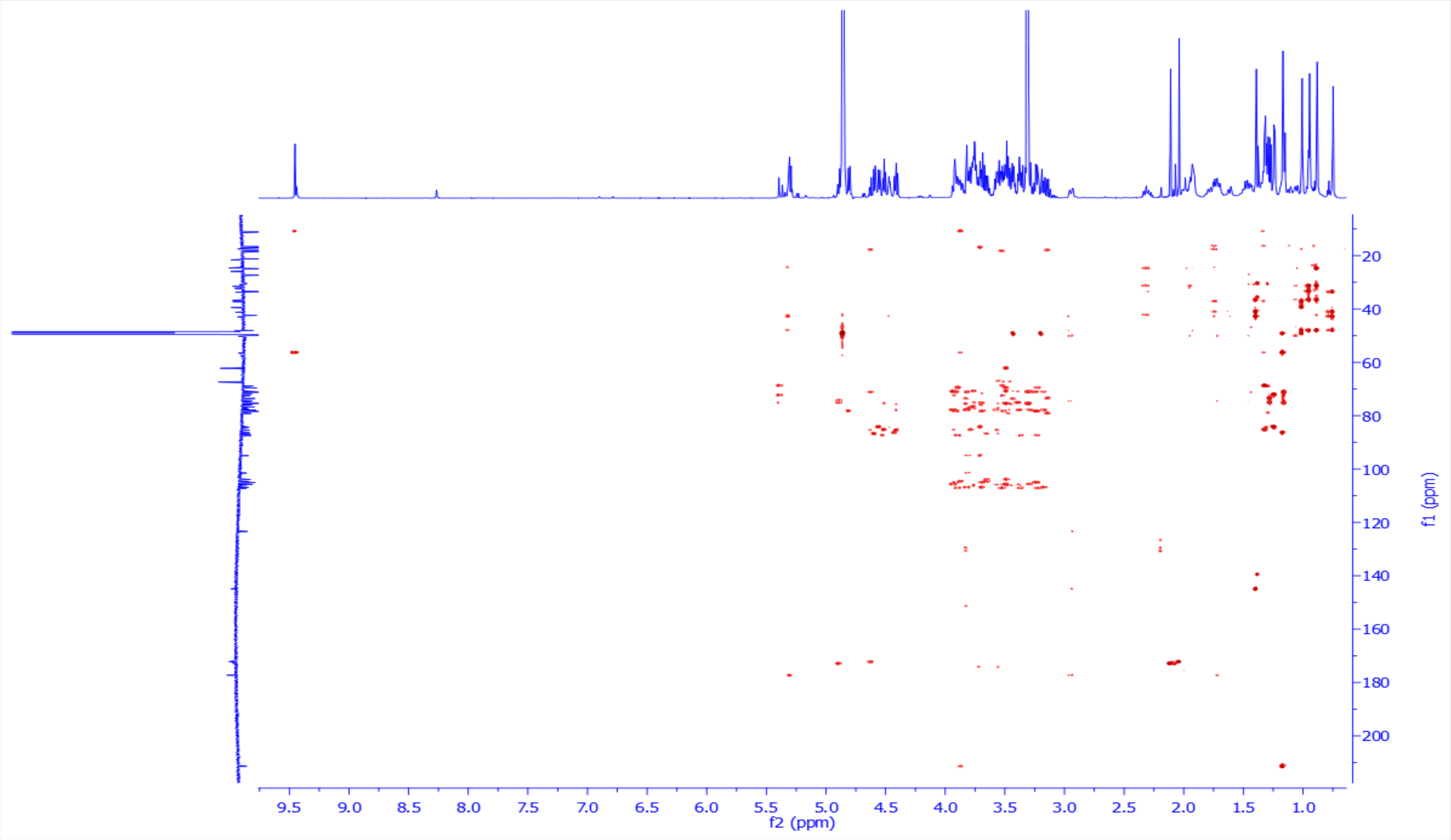

Supplementary Fig. 9. **^{1}H-^{13}C HMBC spectrum of saponarioside A recorded in MeOH-*d*$_4$, 600/150 MHz.**

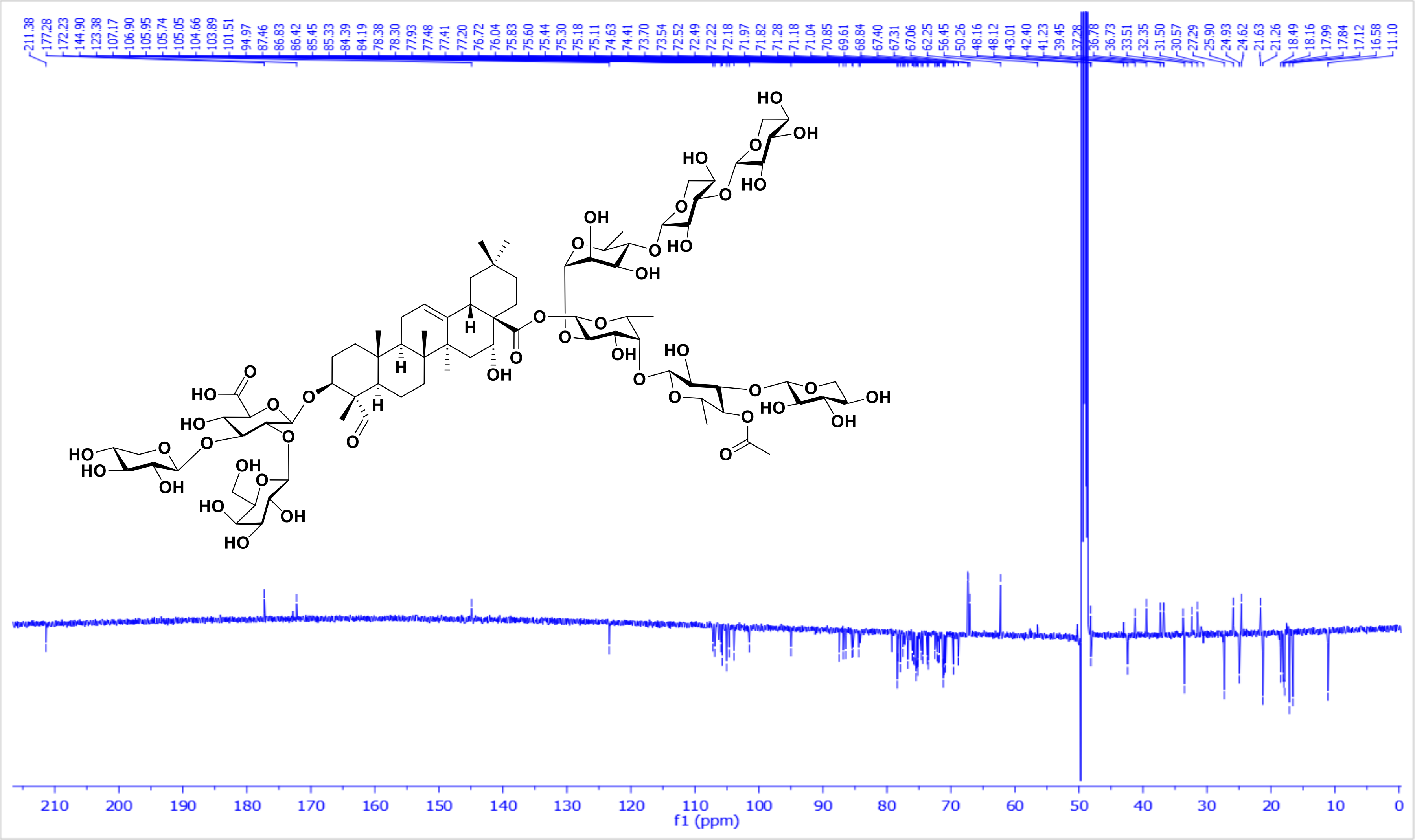

Supplementary Fig. 10. DEPTQ-135 spectrum of saponarioside A recorded in MeOH-d_4, 150 MHz.

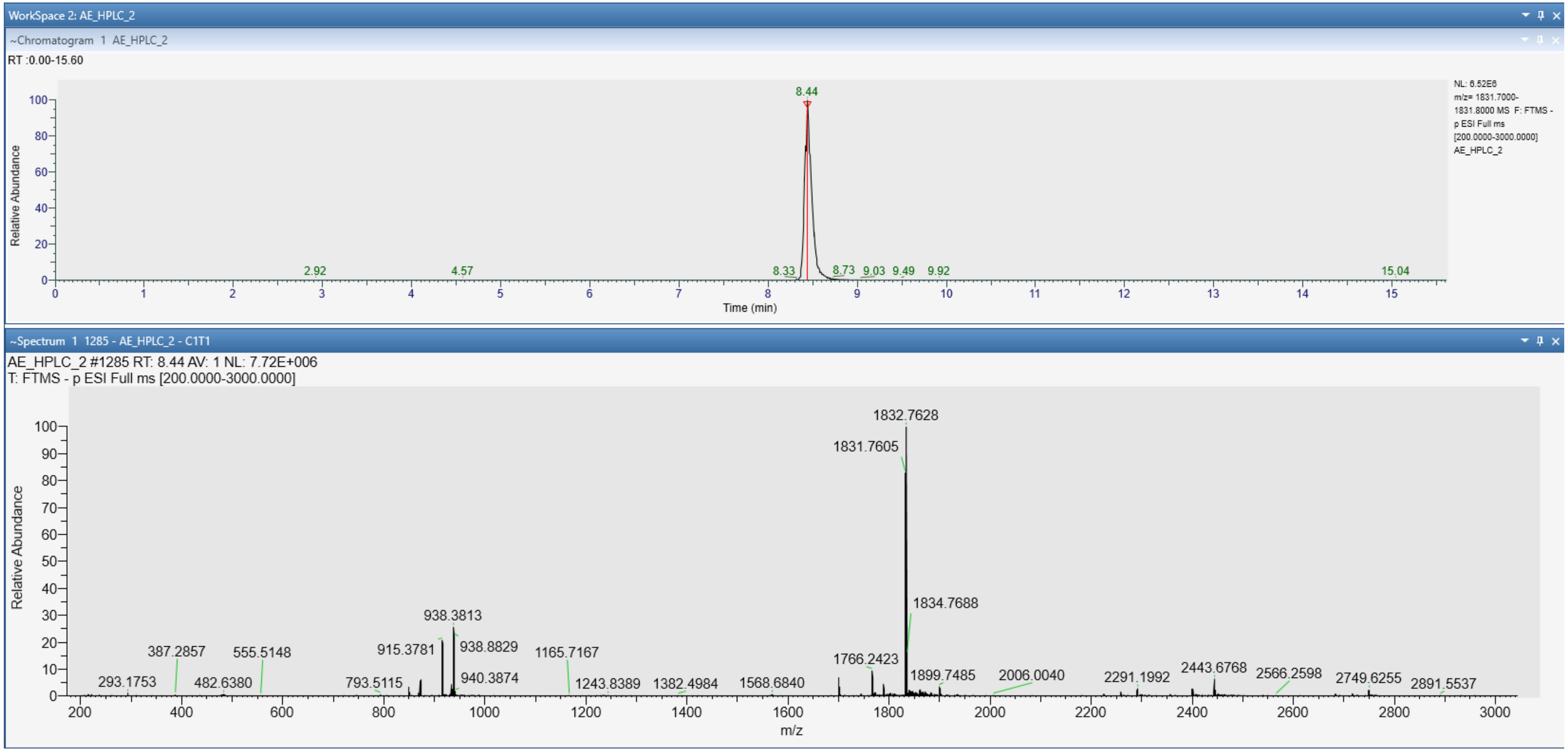

Supplementary Fig. 11. HR-LC-MS (EIC) of saponarioside A.

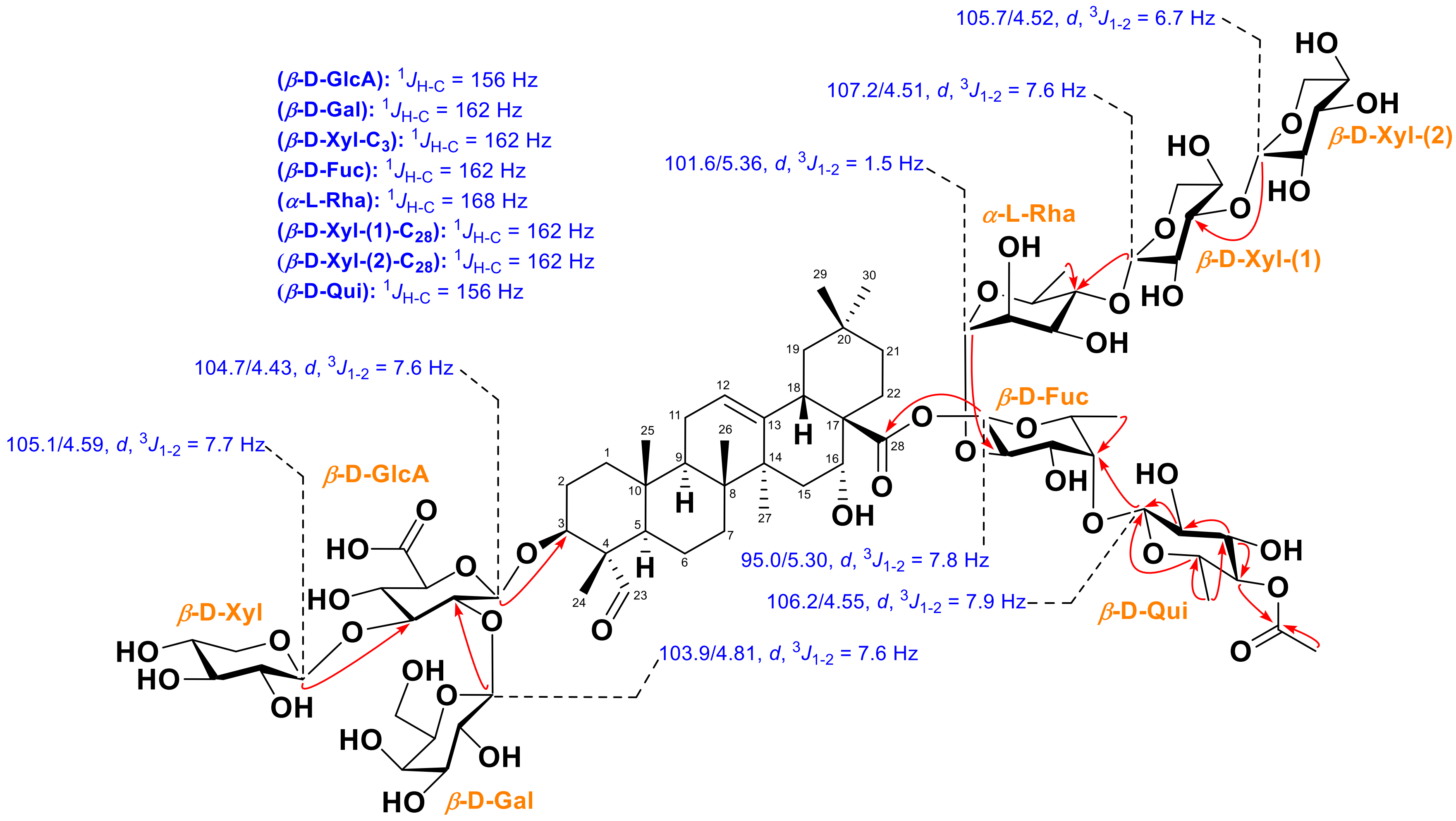

Supplementary Fig. 12. Key HMBC and coupled HSQC coupling constants ($^{1}J_{H\text{-}C}$) recorded for saponarioside B (13) purified from *S. officinalis* leaves. Red arrows represent H→C.

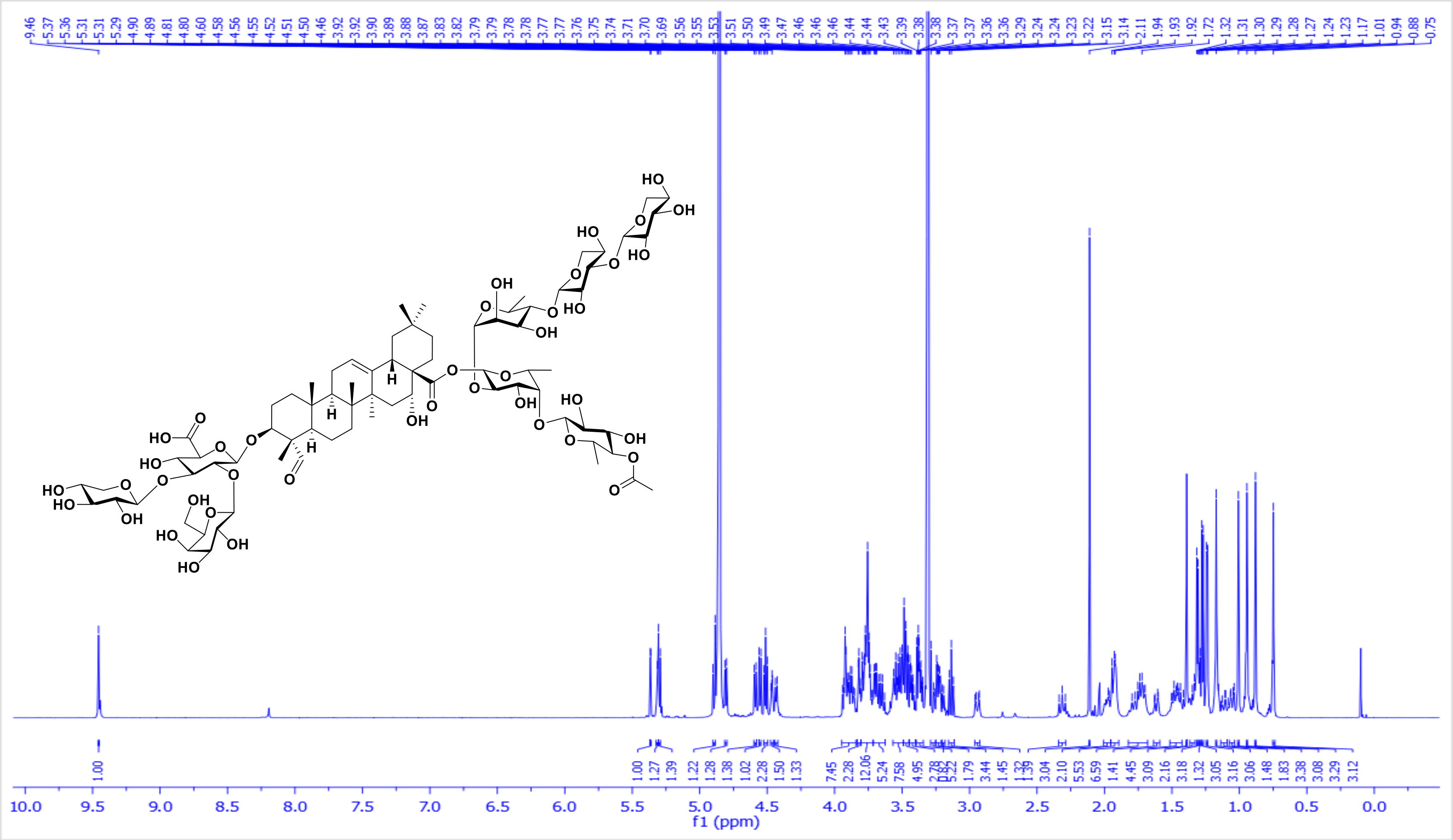

Supplementary Fig. 13. **^{1}H-NMR spectrum of saponarioside B (13) recorded in MeOH-*d*$_4$, 600 MHz.**

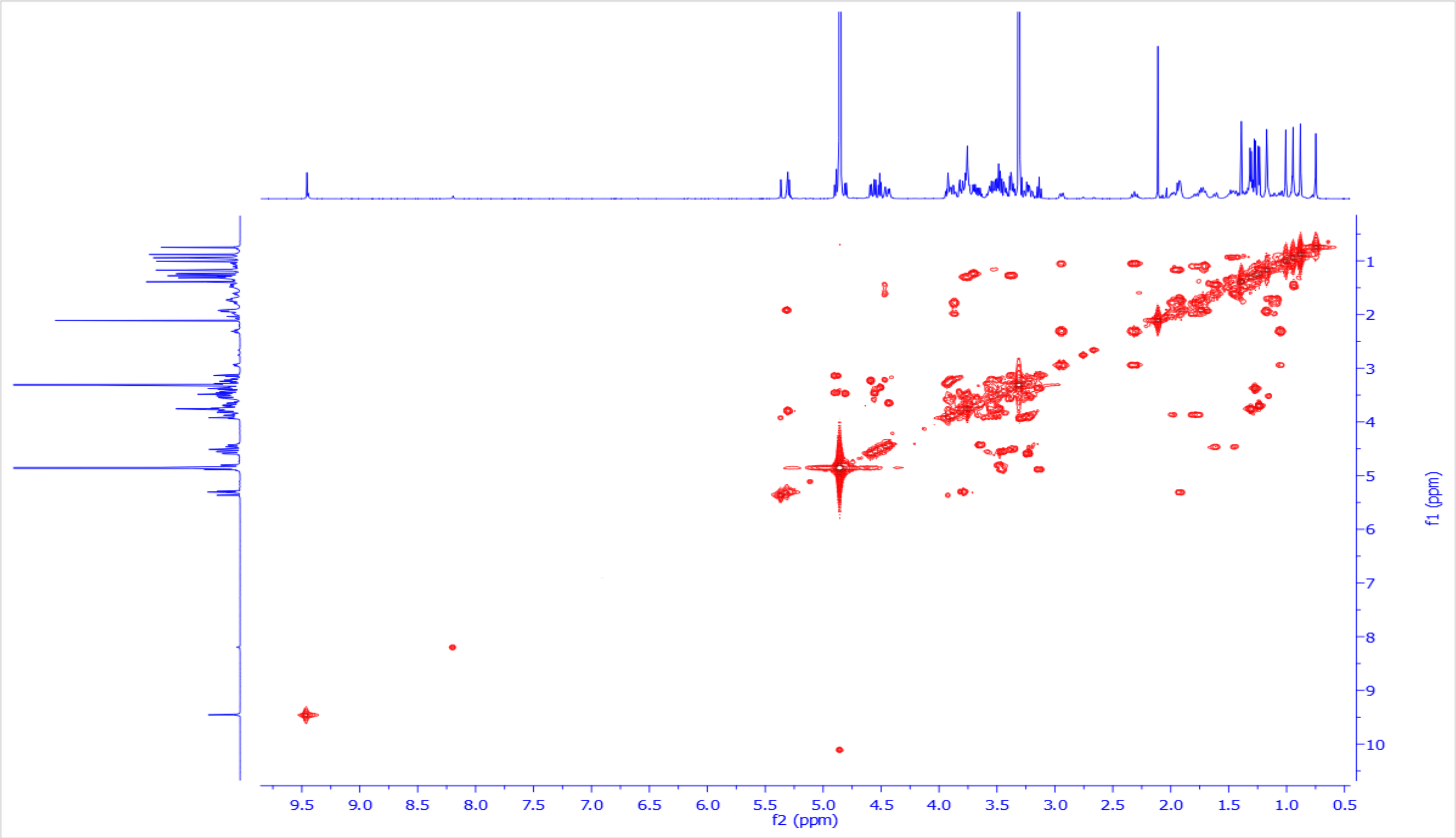

Supplementary Fig. 14. ^{1}H-^{1}H **COSY spectrum of saponarioside B (13) recorded in MeOH-*d*$_4$, 600 MHz.**

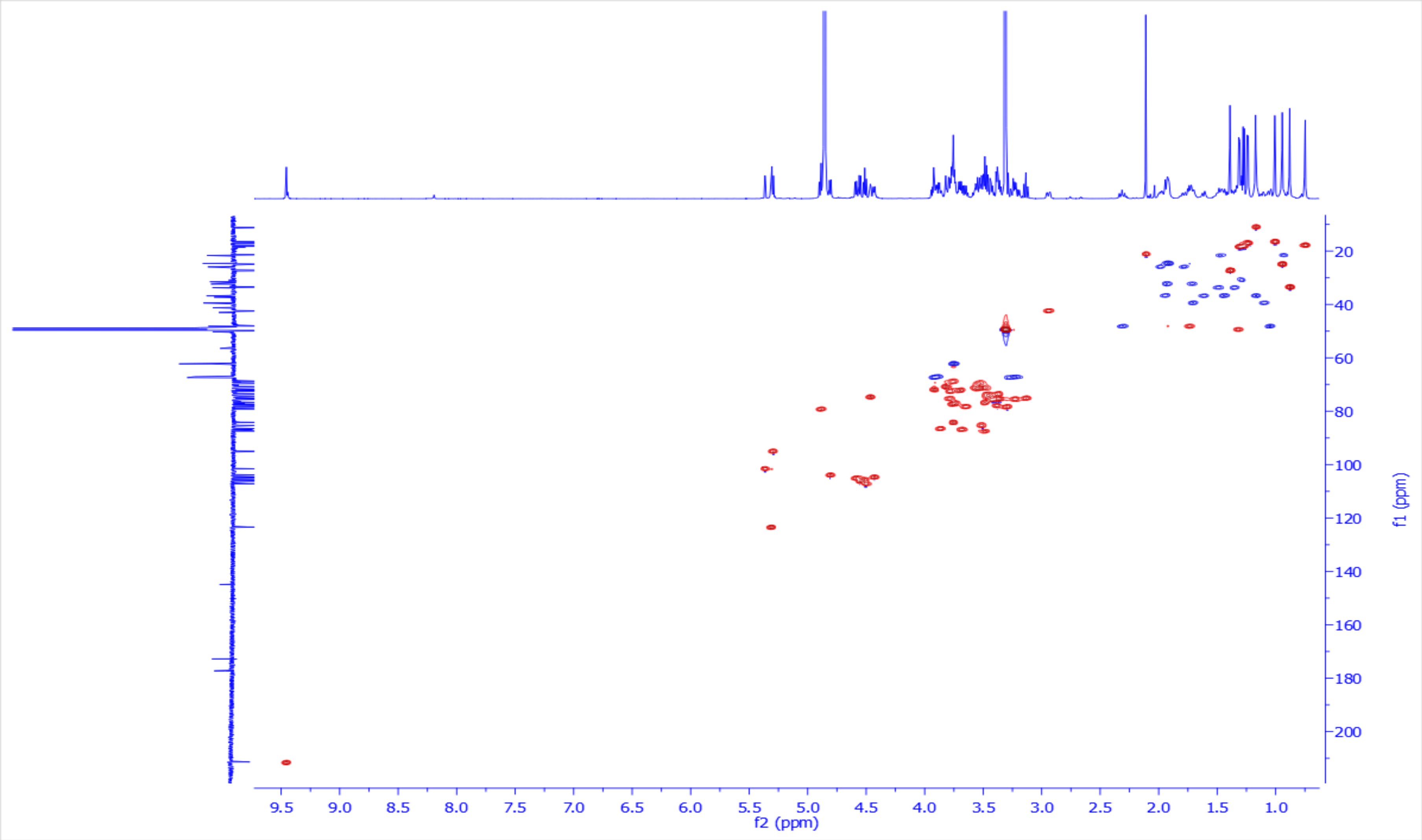

Supplementary Fig. 15. **^{1}H-^{13}C HSQC spectrum of saponarioside B (13) recorded in MeOH-*d*$_4$, 600/150 MHz.**

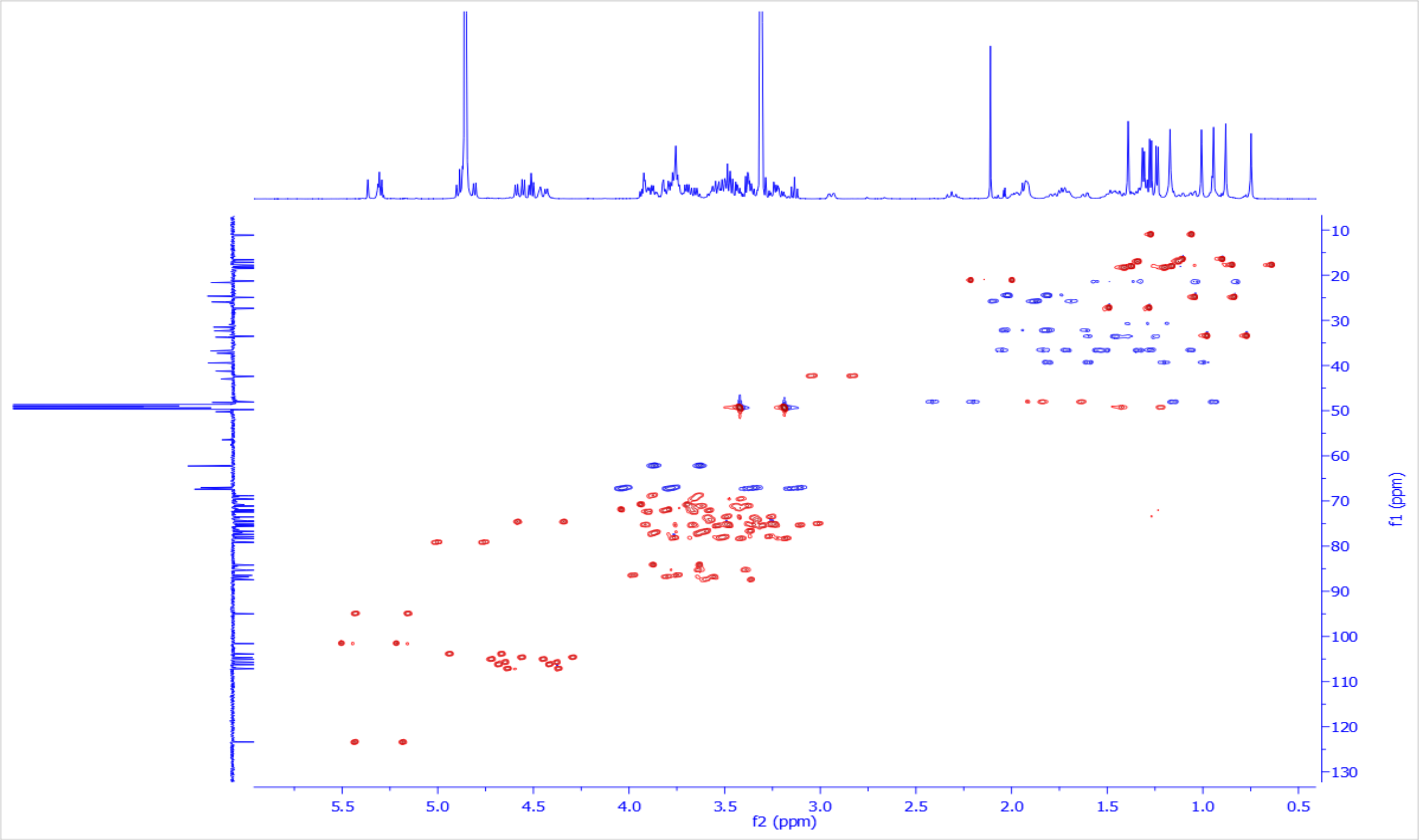

Supplementary Fig. 16. ^{1}H-^{13}C Coupled HSQC spectrum of saponarioside B (13) recorded in MeOH-*d*$_4$, 600/150 MHz.

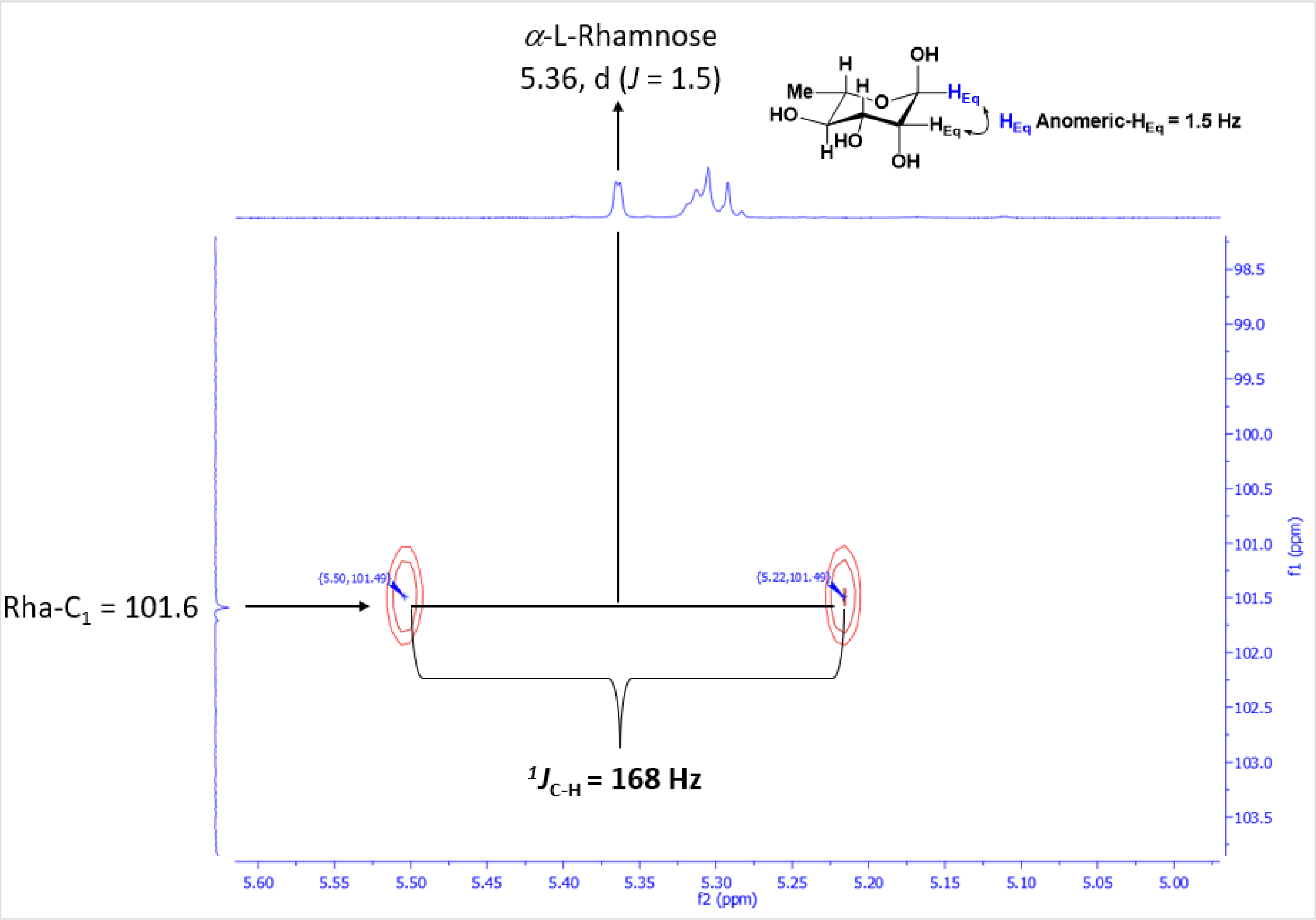

Supplementary Fig. 17. Expanded 1H-13C Coupled HSQC spectrum for (C28-Rhamonsyl motif) of saponarioside B (13) recorded in MeOH-d4, 600/150 MHz.[6,7]

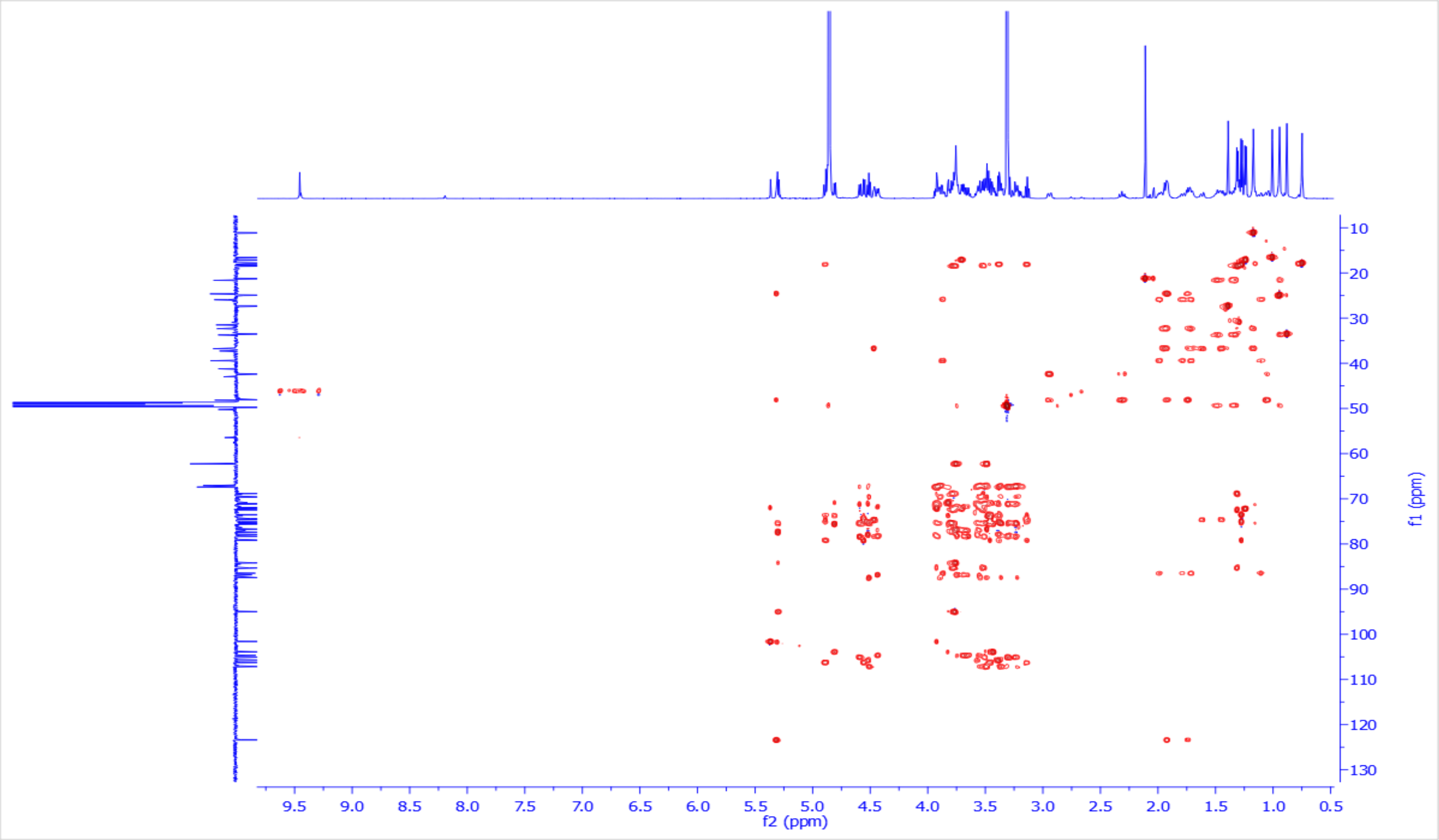

Supplementary Fig. 18. **^{1}H-^{13}C HSQC-TOCSY spectrum of saponarioside B (13) recorded in MeOH-d_4, 600/150 MHz.**

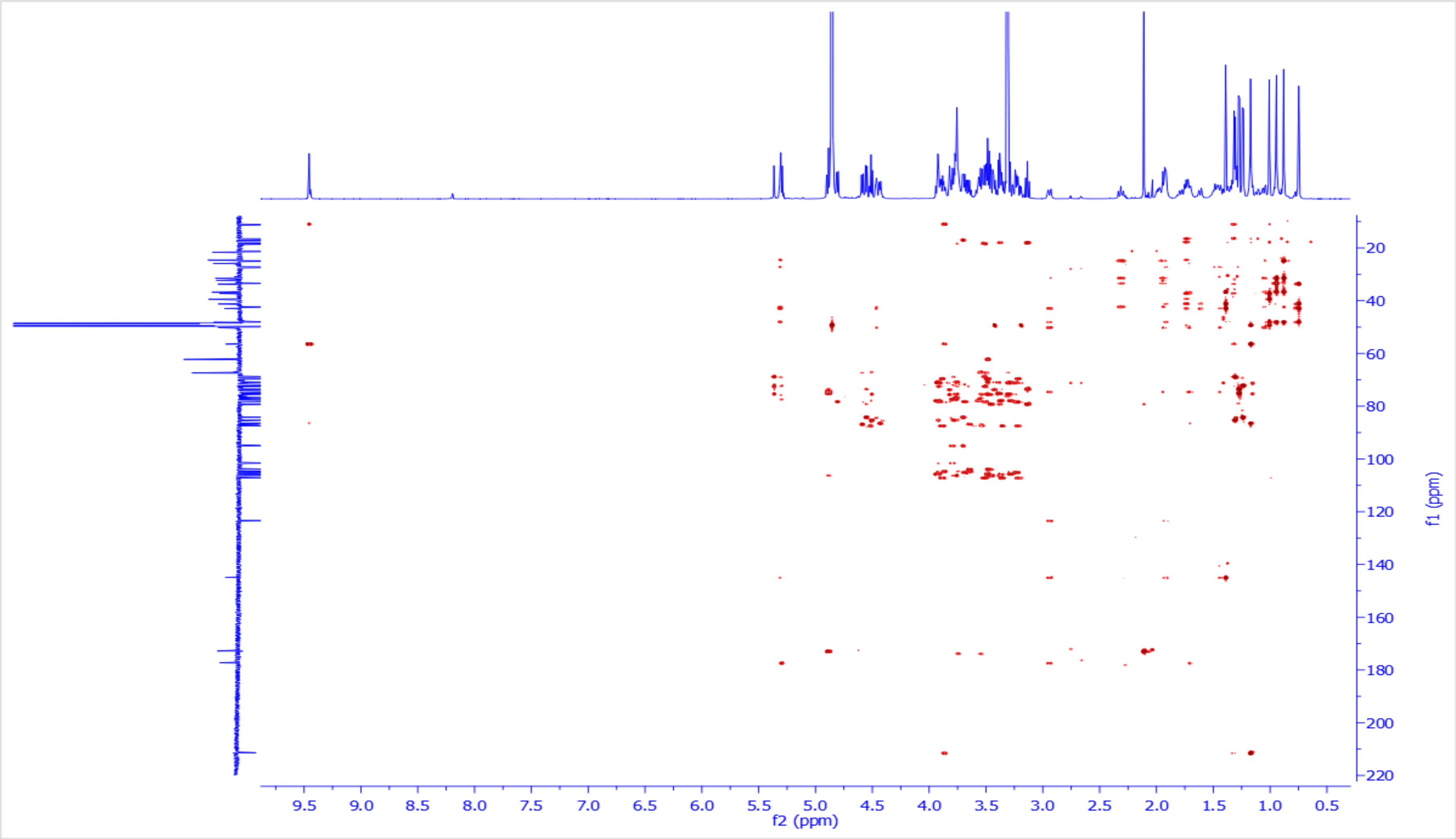

Suppelmentary Fig. 19. ^{1}H-^{13}C HMBC spectrum of saponarioside B (13) recorded in MeOH-d_4, 600/150 MHz.

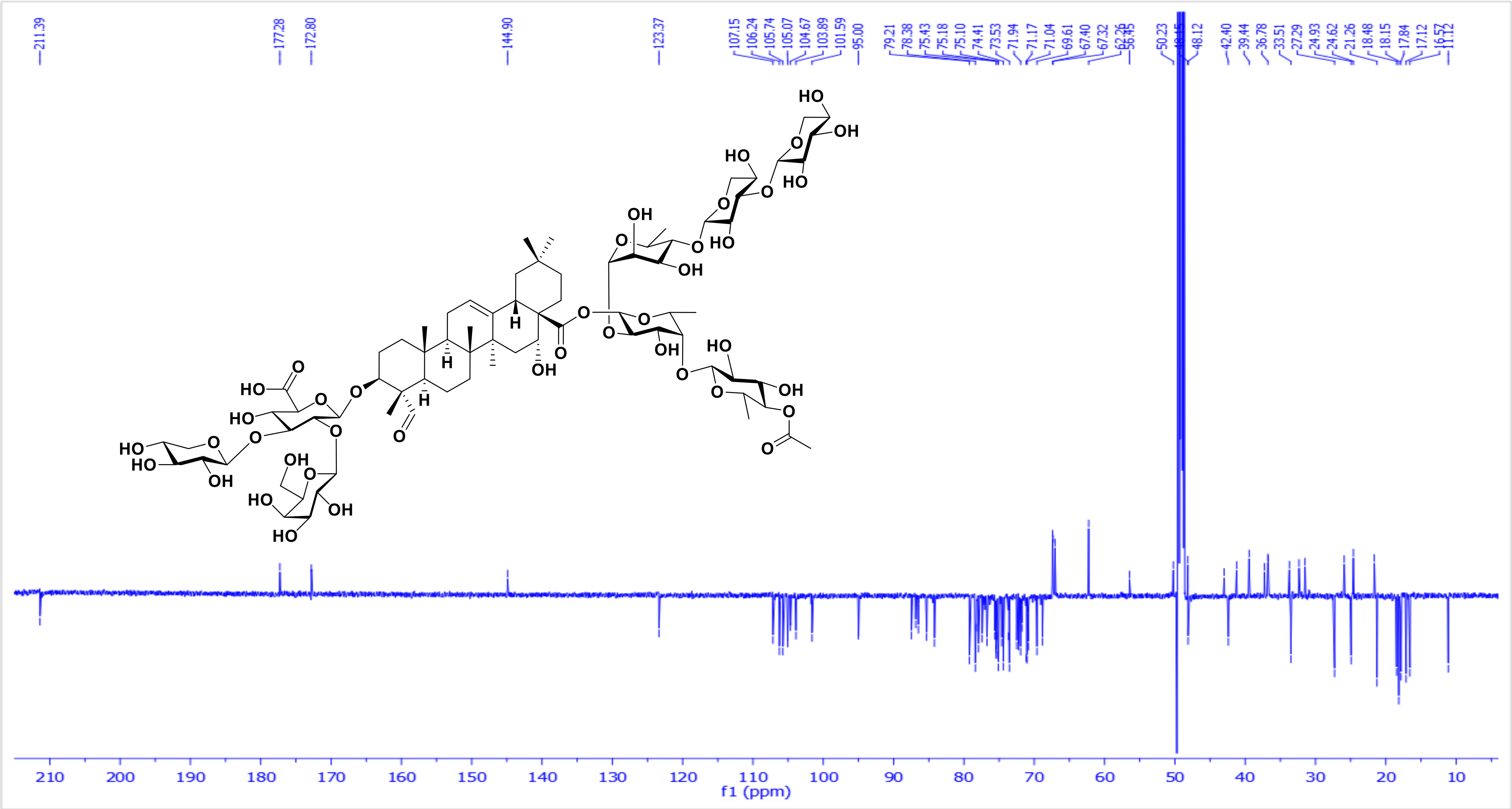

Supplementary Fig. 20. DEPTQ-135 spectrum of saponarioside B (13) recorded in MeOH-d_4, 150 MHz.

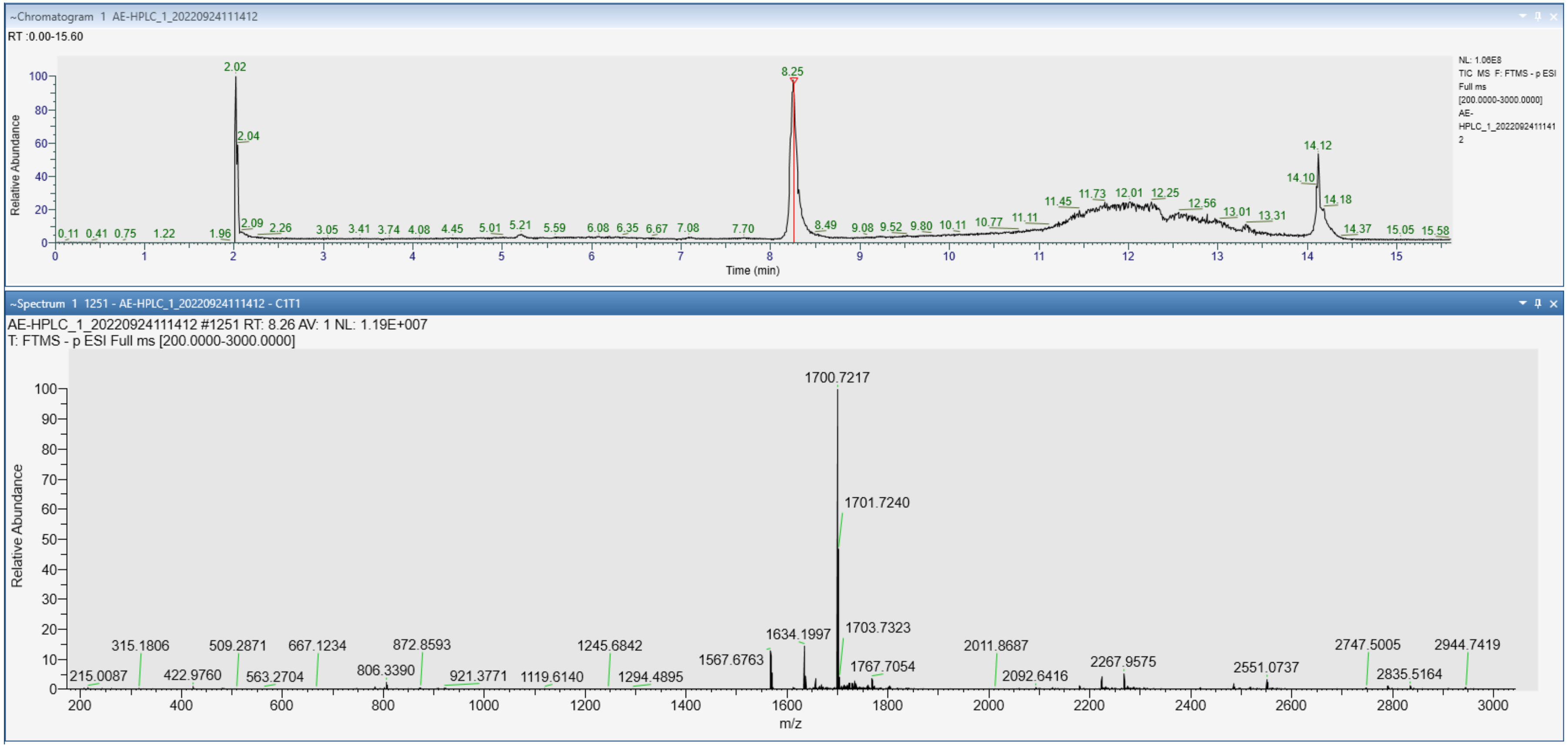

Supplementary Fig. 21. HR-LC-ESI-MS (TIC) of saponarioside B (13).

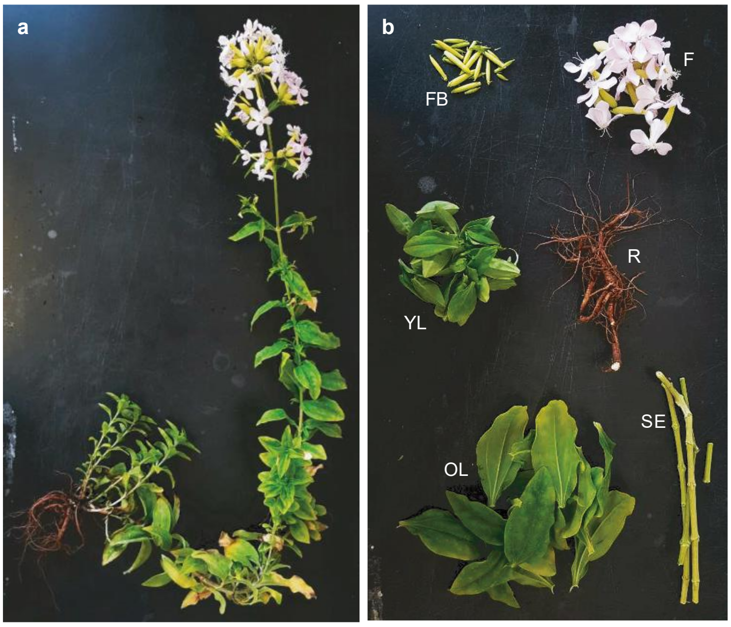

Supplementary Fig. 22. Representative image of a soapwort plant harvested in July 2019. a. Whole plant. **b.** The different organs harvested: flower bud (fb), flower (f), young leaf (yL), old leaf (oL), root (r), stem (se).

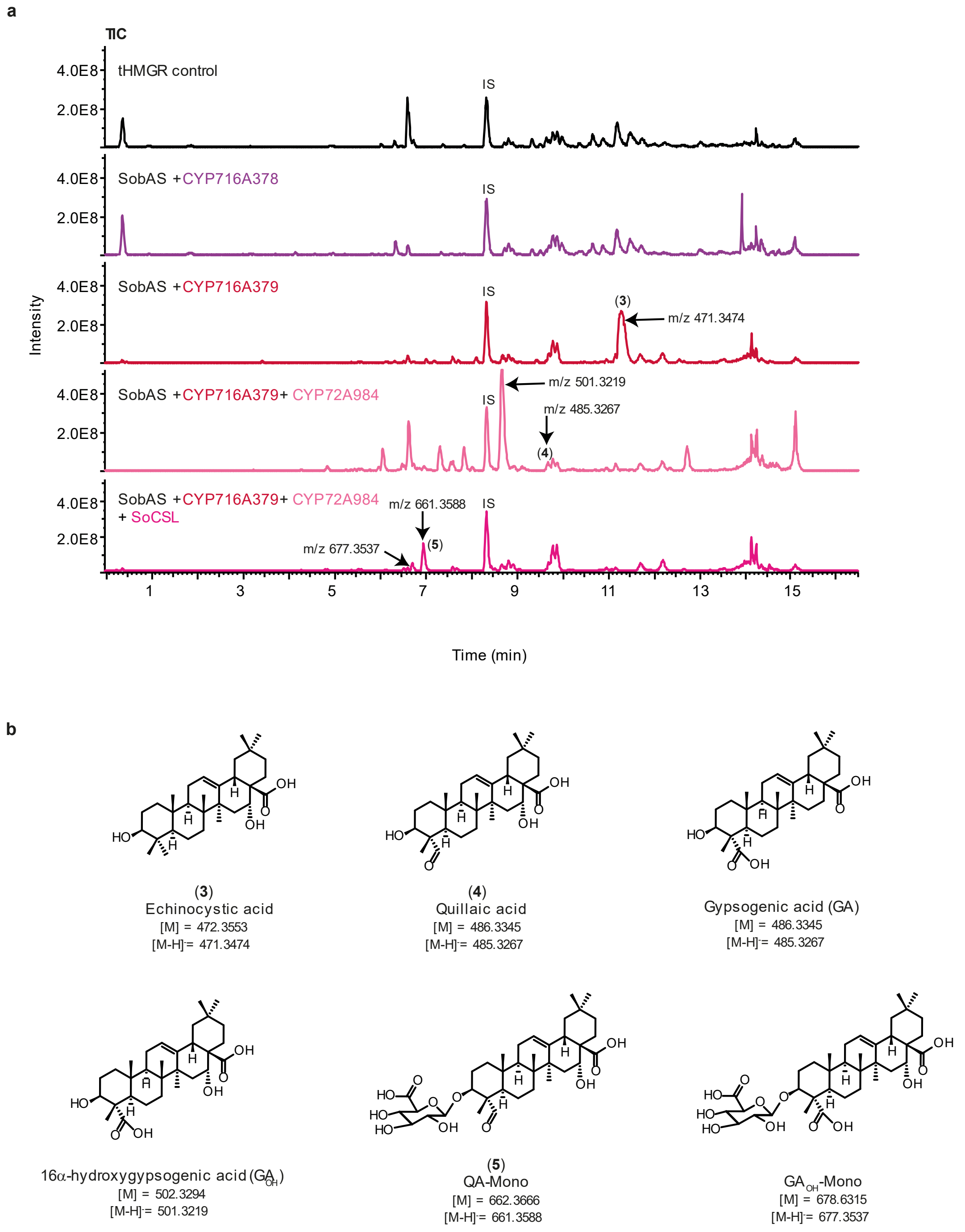

Supplementary Fig. 23. Activity of CYP716A378, CYP716A369, CYP72A984 and SoCSL1. **a.** Total ion chromatograms (TIC) of *N. benthamiana* leaves transiently expressing different gene combinations together with *AstHMGR* and *SobAS1*. Leaf extracts were analysed using HPLC-MS in negative ionization mode. The extract from *N. benthamiana* leaves only expressing *AstHMGR* was used as a negative control (tHMGR control). New product peaks of interest are labelled, and the extracted ion chromatograms (EIC) are shown in Supplementary Figs. 24-26 and 30. IS, internal standard digitoxin. **b.** Predicted enzyme products. Identity of compounds **3**, **4** and **5** are confirmed by authentic standards, and identities of GA, GA_{OH}, GA_{OH}-Mono are putatively identified based on the expected $[M-H]^-$. GA_{OH}-Mono, 3-*O*-{β-D-glucopyranosiduronic acid}-16α-hydroxygypsogenic acid.

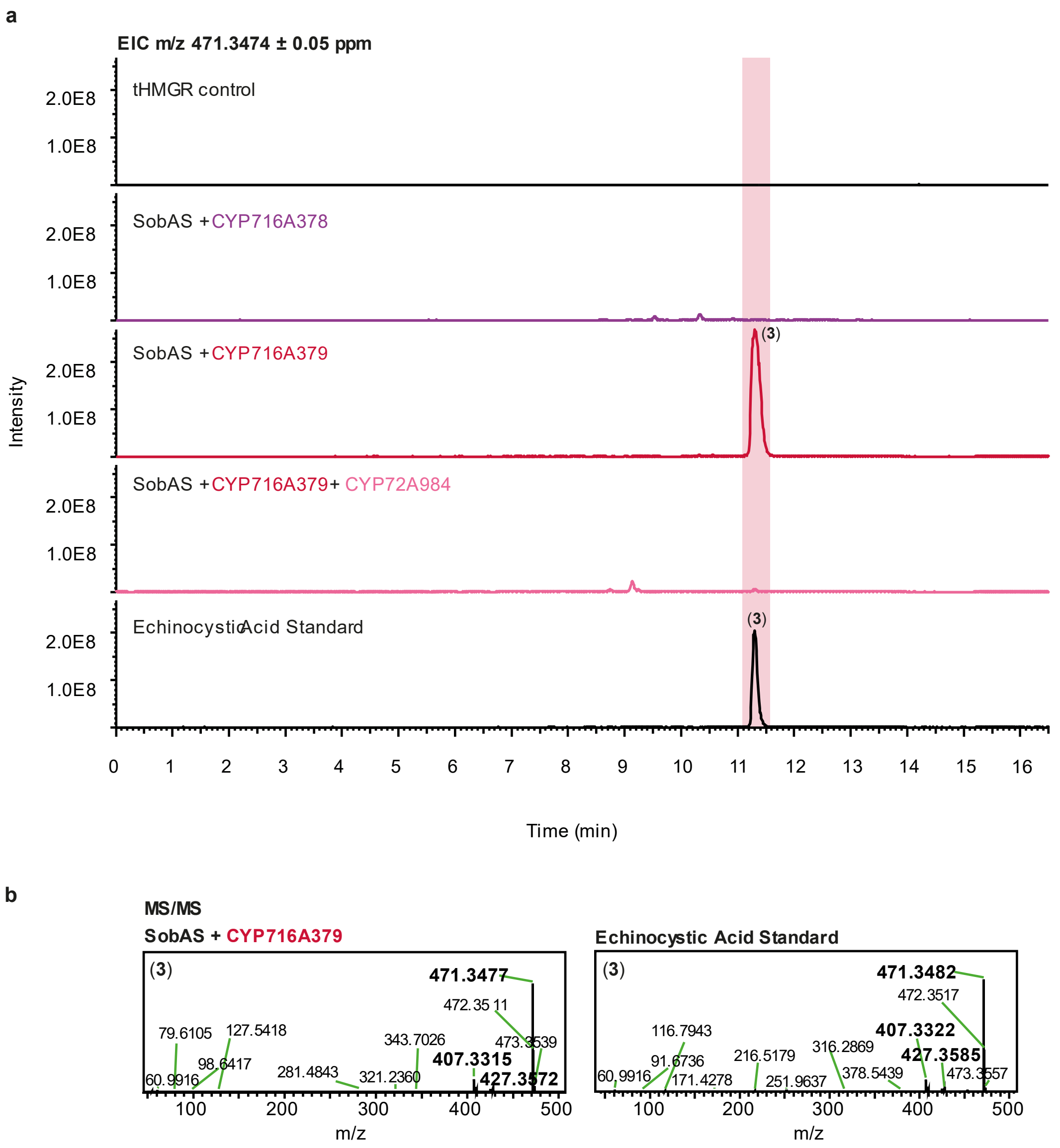

Supplementary Fig. 24. Production of echinocystic acid by activity of CYP716A379. a. HPLC-MS EIC at *m/z* 471.3474 from extracts of *N. benthamiana* leaves co-expressing different gene combinations together with *AstHMGR* and *SobAS1*. **b.** MS/MS fragmentation of peak (**3**) compared to echinocystic acid standard. The extract from *N. benthamiana* leaves only expressing *AstHMGR* was used as a negative control (tHMGR control). Identity of (**3**) is confirmed by comparison with commercial echinocystic acid standard.

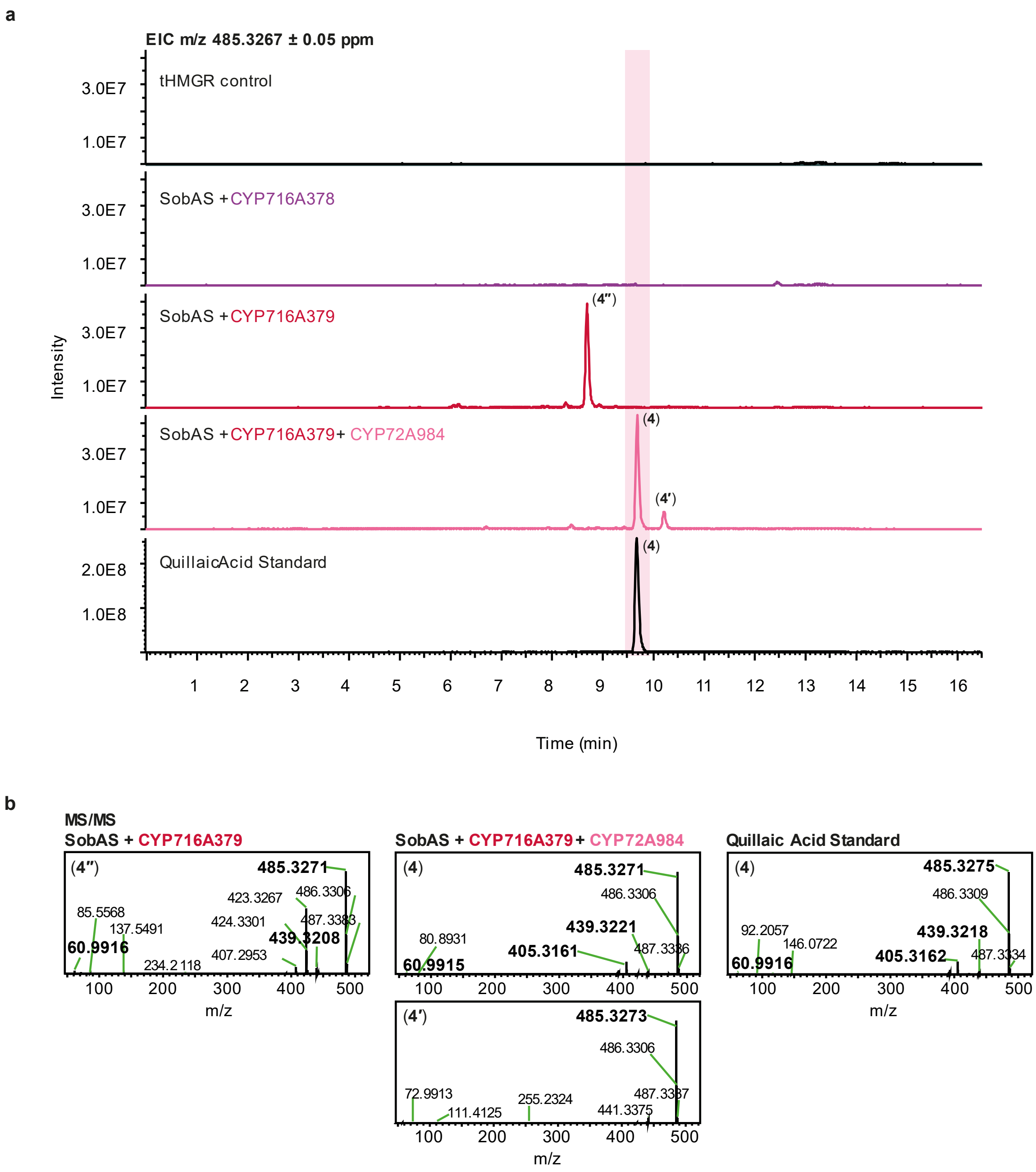

Supplementary Fig. 25. Production of quillaic acid by activity of CYP72A984. a. HPLC-MS EIC at *m/z* 485.3267 from extracts of *N. benthamiana* leaves co-expressing different gene combinations together with *AstHMGR* and *SobAS1*. **b.** MS/MS spectra of detected peaks. *N. benthamiana* leaves only expressing *AstHMGR* was used as a negative control (tHMGR control). The activity of CYP72A984 produced a major peak corresponding to (**4**), identified by comparison with a commercial quillaic acid standard, and a minor peak (**4′**) predicted to be gypsogenic acid based on expected [M-H]$^-$ of gypsogenic acid. The activity of CYP716A379 in combination with SobAS1 also produced a peak (**4″**) with *m/z* 485.3267 but with a different retention time to quillaic acid. However, compared to **4″**, compound **3** accumulates in much higher levels and is likely to be the main product produced by CYP716A379 while **4″** is likely a product of off-target enzyme activity in *N. benthamiana*.

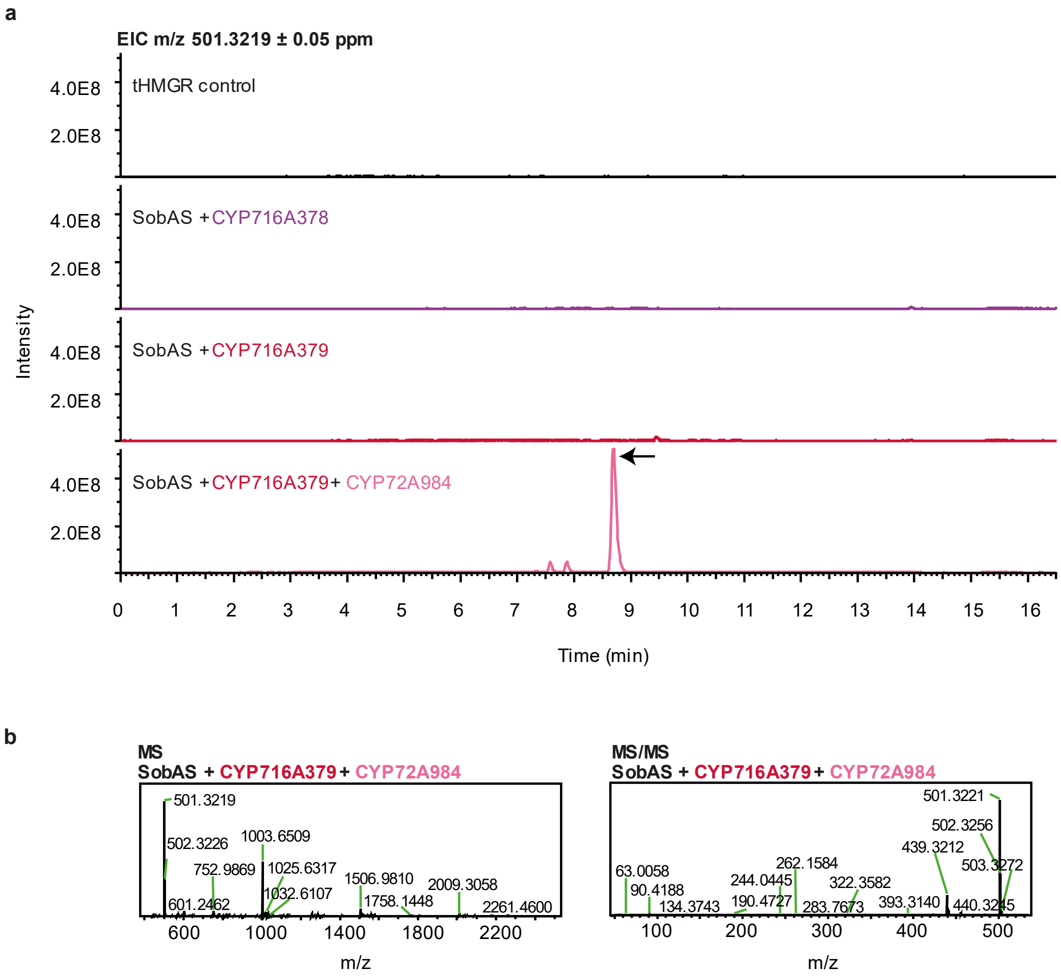

Supplementary Fig. 26. New product peak produced by activity of CYP72A984. a. HPLC-MS EIC at *m/z* 501.3219 from extracts of *N. benthamiana* leaves co-expressing gene combinations together with *AstHMGR* and *SobAS1*. **b.** MS and MS/MS spectra of the product peak. The *m/z* of this product peak corresponds to the expected $[M-H]^-$ of 16α-hydroxygypsogenic acid. MS/MS fragmentation reveals a major fragment ion of *m/z* 439.3212, suggesting a loss of $[COOH + OH]^-$. Based on *m/z* and fragmentation pattern, this new peak is putatively identified as 16α-hydroxygypsogenic acid.

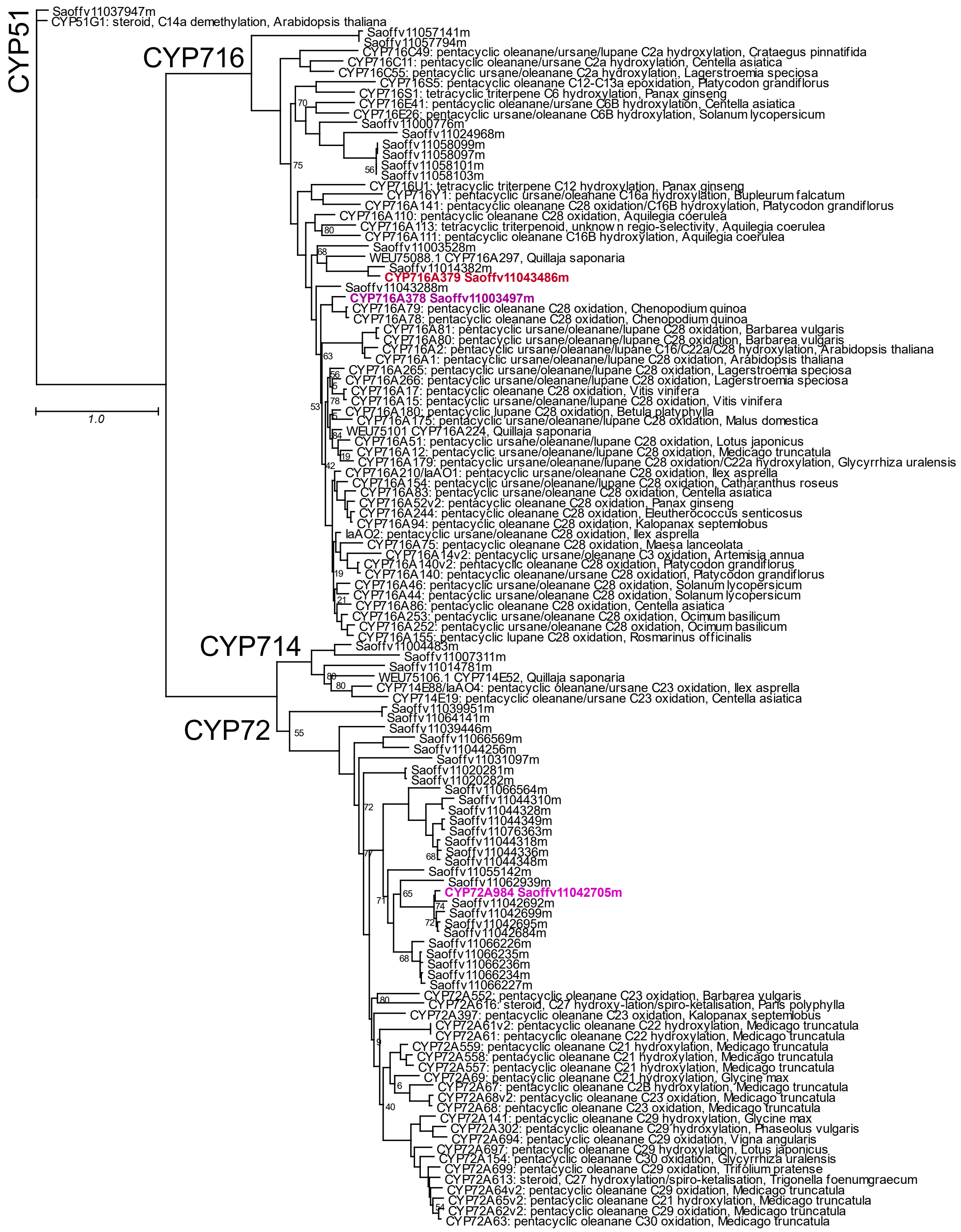

Supplementary Fig. 27. Phylogenetic tree of CYP716, CYP714 and CYP72 candidates in soapwort. Genome mining, alignment of protein sequences and generation of maximum likelihood phylogeny was carried out as descried in methods. Reference sequences were included from [8] and [9], with full CYP name and brief description of activity shown on the tree. Functional *S. officinalis* genes required for synthesis of quillaic acid are in bold and coloured (C-28 oxidase in red, dual C-28/C16 oxidase in purple, C-23 oxidase in pink). Bootstrap values less than 80% are shown beside each node. The scale bar indicates the number of amino acid substitutions per site.

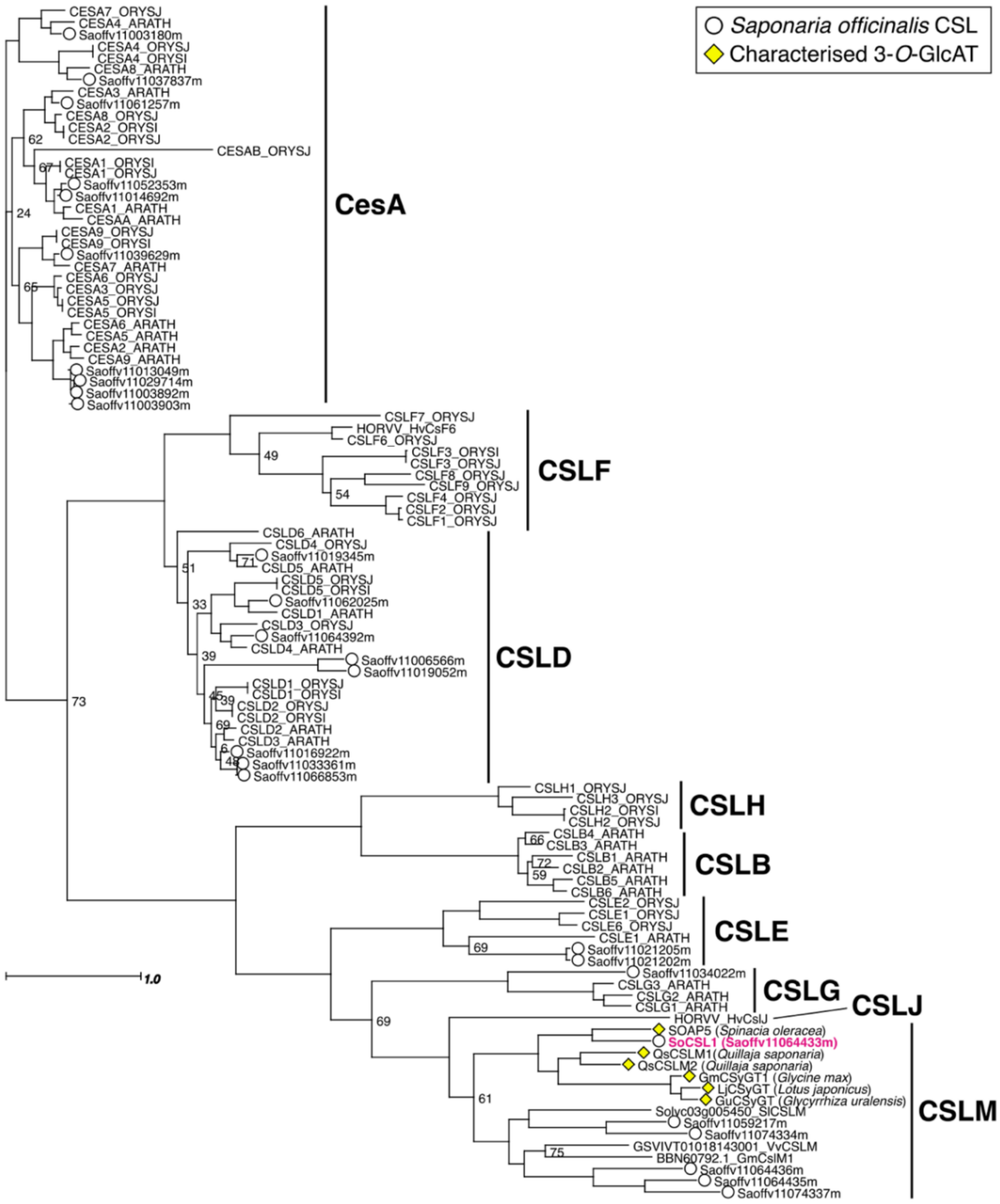

Supplementary Fig. 28. Phylogenetic tree of CSL candidates in soapwort. Genome mining, alignment of protein sequences and generation of maximum likelihood phylogeny was carried out as descried in methods. Sequences from *S. officinalis* are labelled with white circles. Yellow diamonds indicate characterised GlcA transferase activity. All UniProt sequences of this gene family were included from *Arabidopsis thaliana*, *Oryza sativa* Japonica and *Oryza sativa* Indica (UniProt names displayed on tree). GenBank IDs for additional sequences included in the alignment and phylogeny in are from *Vitis vinifera*: GSVIVT01018143001_VvCSLM (CBI26389.3); *Hordeum vulgare*: HORVV_HvCsF6 (XP_044960146.1), HORVV_HvCsLJ (XP_044974896.1); *Solanum lycopersicum*: Solyc03g005450_SlCSLM (XP_004234035.1); *Glycine max*: BBN60792.1_GmCslM1 (XP_003536256.1), GmCSyGT1 (XP_006582441.1); *Glycyrrhiza uralensis*: GuCSyGT (BBN60794.1); *Lotus japonicus*: LjCSyGT (BBN60795.1); *Spinacia oleracea*: SOAP5 (XP_021842158.1) and *Quillaja saponaria*: QsCSLM1 (WEU75093.1), QsCSLM2 (WEU75105.1). Bootstrap values less than 80% are shown beside each node. The scale bar indicates the number of amino acid substitutions per site.

a

QA-Mono (C36H54O11)

Exact mass [M]	662.3666
[M-H]⁻	661.3588
[M-176-H]⁻	485.3267

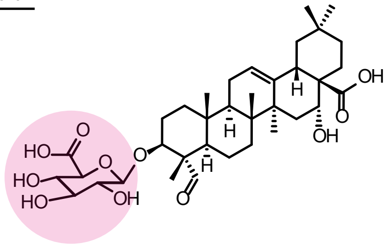

(**5**)

b

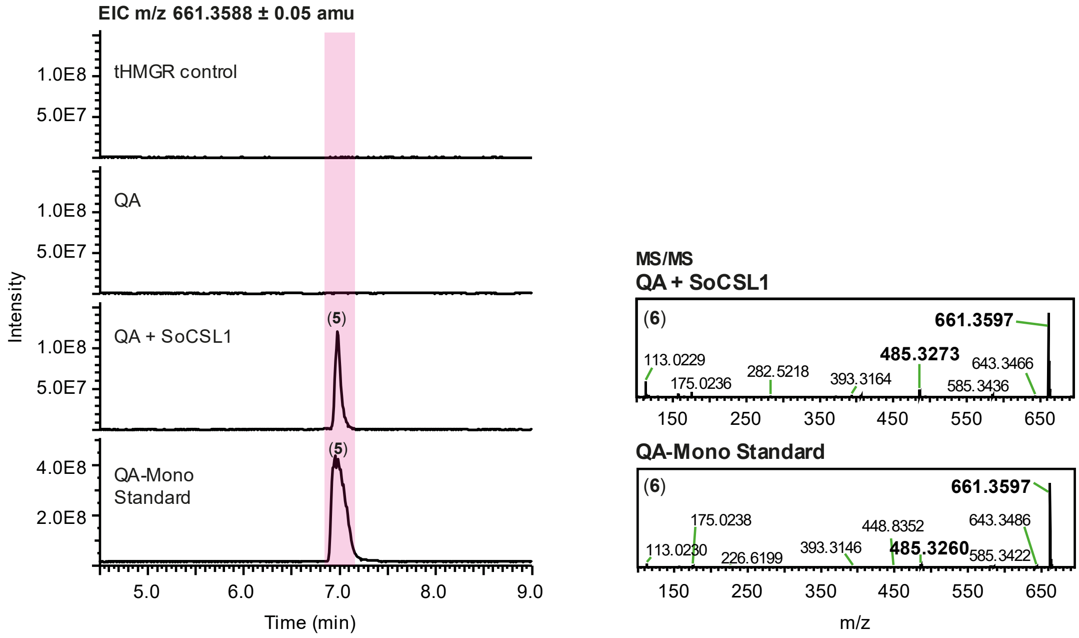

Supplementary Fig. 29. Characterization of SoCSL1. a. Structure of 3-*O*-{β-D-glucopyranosiduronic acid}-quillaic acid (QA-Mono, **5**), the product of SoCSL1 when acting in combination with *S. officinalis* enzymes required for production of quillaic acid (QA, **4**). Modification performed by SoCSL1 has been highlighted and a table showing relevant calculated adducts and fragments of **5** included. **b.** *N. benthamiana* leaves transiently co-expressing various genes were extracted and analysed using HPLC-MS, representative extracted ion chromatograms (EIC) and MS/MS spectra are shown. EIC displayed are for *m/z* 661.3588, the calculated mass of the [M-H]⁻ adduct of **5**. The negative controls used were extracts from *N. benthamiana* leaves co-expressing only *AstHMGR* (tHMGR control) or co-expressing the *S. officinalis* genes required to produce **4** (*tHMGR*, *SobAS1*, *CYP716A379 and CYP72A984*) (QA). The additional activity of SoCSL1 produced a peak corresponding to **5**, identified as QA-Mono by comparison with an authentic standard.

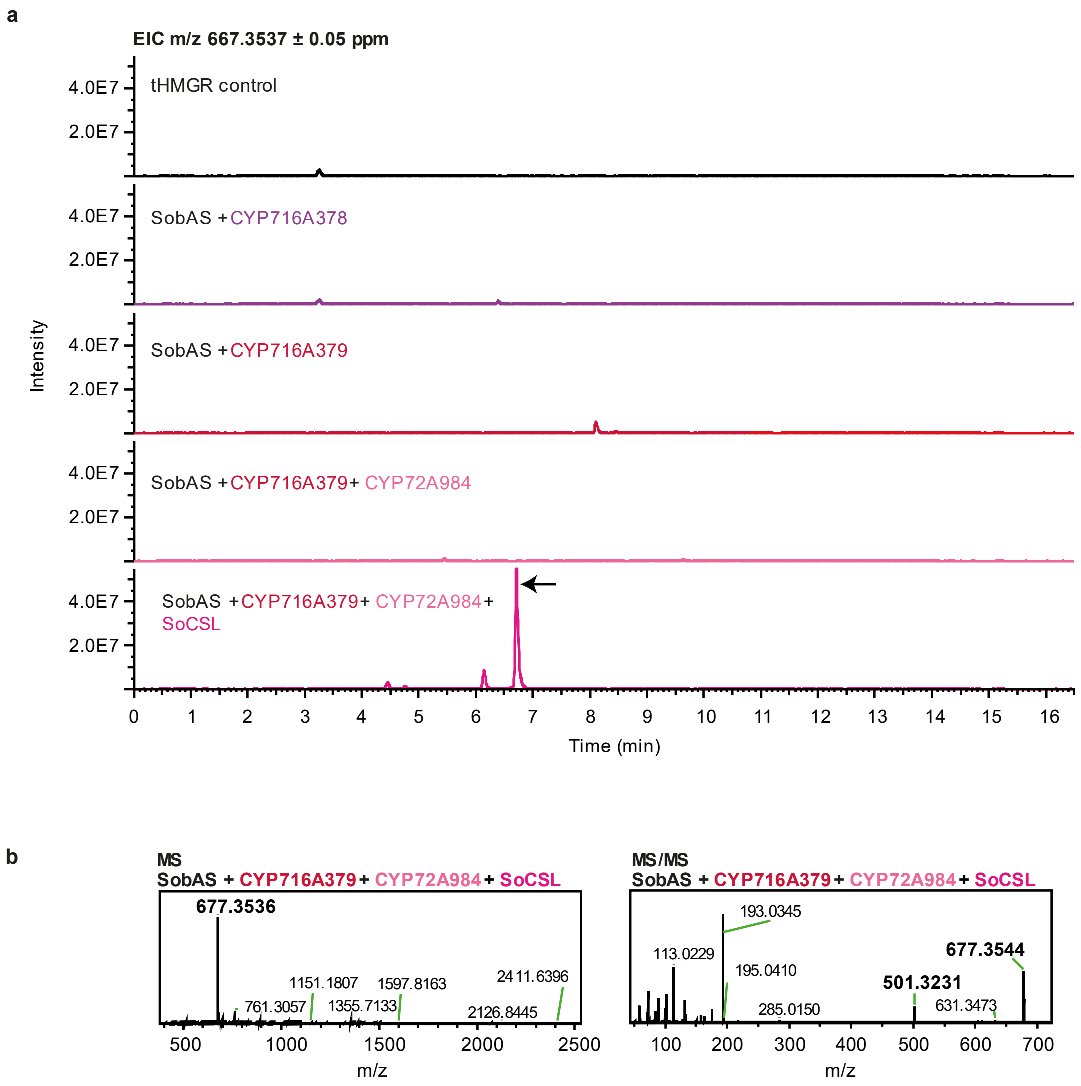

Supplementary Fig. 30. New product peak produced by activity of SoCSL. a. HPLC-MS EIC at *m/z* 667.3537 from extracts of *N. benthamiana* leaves co-expressing different gene combinations together with *AstHMGR* and *SobAS1* **b.** MS and MS/MS spectra of the emphasized product peak. The *m/z* of this product peak corresponds to the expected $[M\text{-}H]^-$ of 16α-hydroxygypsogenic acid with a glucuronic acid. MS/MS fragmentation pattern reveals a major fragment ion of *m/z* 501.3231, suggesting loss of glucuronic acid $[M\text{-}176\text{-}H]^-$. Based on *m/z* and fragmentation pattern, this new peak is putatively identified as 3-*O*-{β-D-glucopyranosiduronic acid}-16α-hydroxygypsogenic acid.

a

QA-Di (C42H64O16)

Exact mass [M]	824.4194
[M-H]⁻	823.4 116
[M-162-H]⁻	661.3588
[M-176-162-H]⁻	485.3267

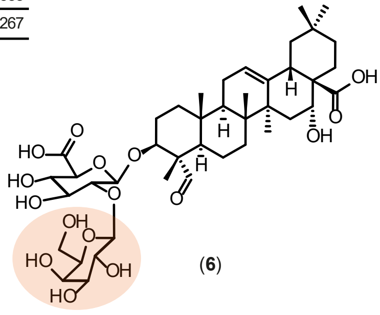

(**6**)

b

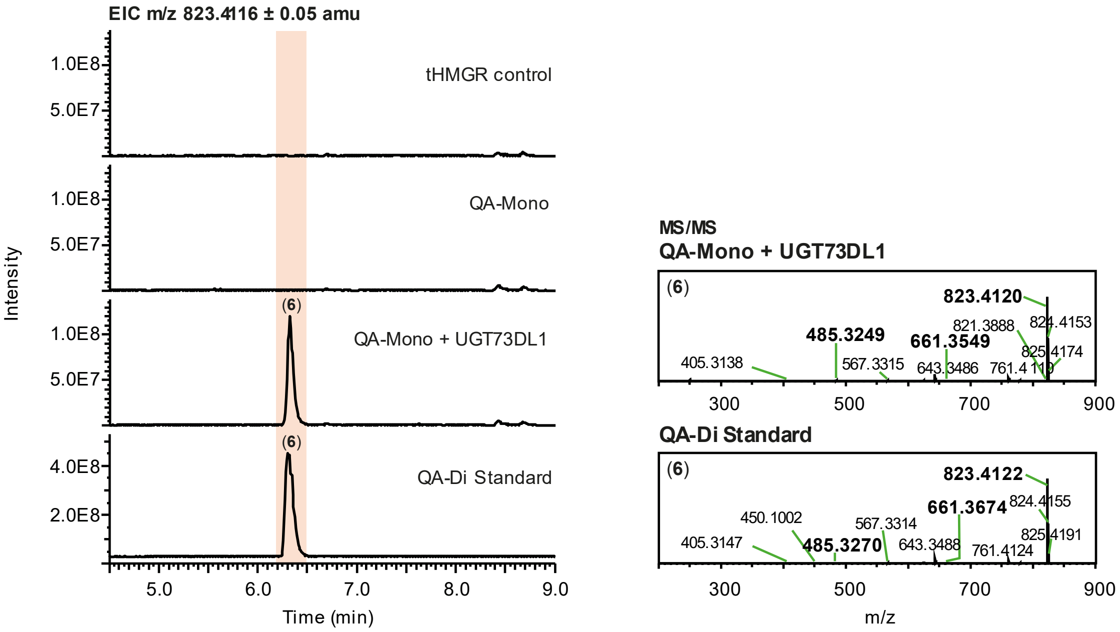

Supplementary Fig. 31. Characterization of UGT73DL1. a. Structure of 3-*O*-{[β-D-galactopyranosyl-(1→2)]-β-D-glucopyranosiduronic acid}-quillaic acid (QA-Di, **6**), product of UGT73DL1 when acting in combination with the *S. officinalis* enzymes required for production of **5**. Modification performed by UGT73DL1 has been highlighted and a table showing relevant calculated adducts and fragments of **6** is included. **b.** *N. benthamiana* leaves transiently co-expressing various genes were extracted and analysed using HPLC-MS, representative (n=6) extracted ion chromatograms (EIC) and MS/MS spectra are shown. EIC displayed are for *m/z* 823.4116, the calculated mass of the [M-H]⁻ adduct of **6**. The negative controls used were extracts from *N. benthamiana* leaves co-expressing only *AstHMGR* (tHMGR control) or co-expressing the *S. officinalis* genes required to produce **5** (*tHMGR*, *SobAS1*, *CYP716A379*, *CYP72A984* and *SoCSL1*) (QA-Mono). The additional activity of *SoUGT73DL1* produced a peak corresponding to **6**, identified by comparison with an authentic QA-Di standard.

a

QA-Tri (C47H72O20)

Exact mass [M]	956.4617
[M-H]⁻	955.4539
[M-132-H]⁻	823.4116
[M-176-162-132-H]⁻	485.3267

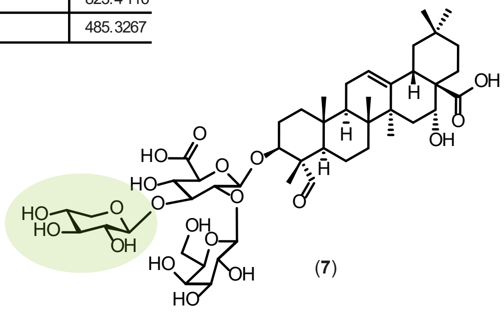

b

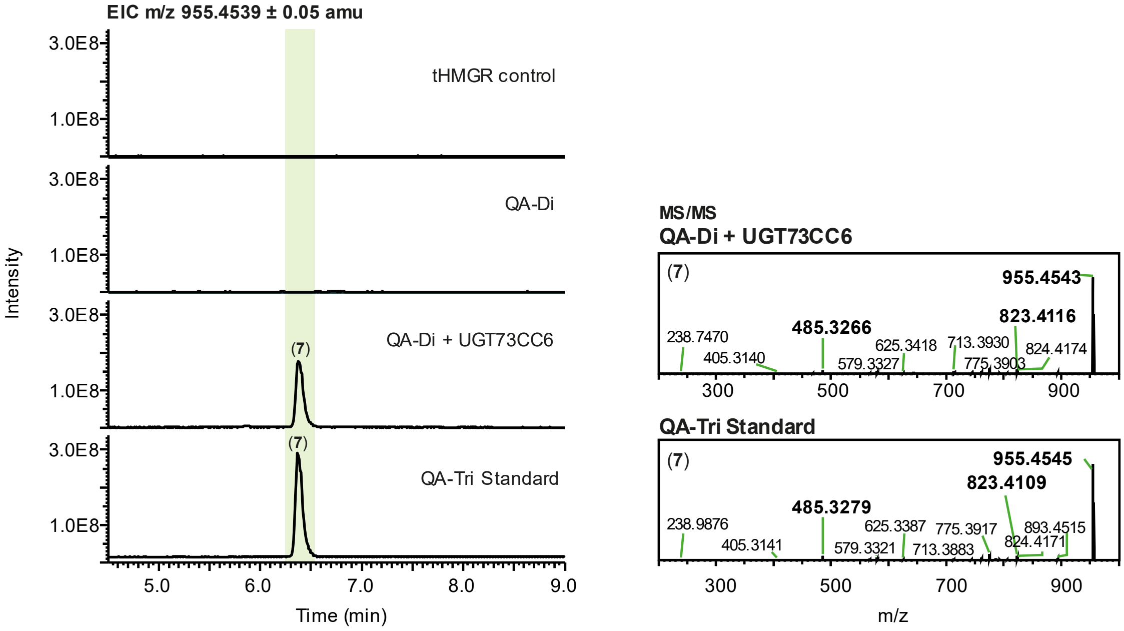

Supplementary Fig. 32. Characterization of UGT73CC6. a. Structure of 3-*O*-{β-D-xylopyranosyl-(1→3)-[β-D-galactopyranosyl-(1→2)]-β-D-glucopyranosiduronic acid}-quillaic acid (QA-Tri, **7**), product of UGT73CC6 when acting in combination with the *S. officinalis* enzymes required for production of **6**. Modification performed by UGT73CC6 has been highlighted and a table showing relevant calculated adducts and fragments of **7** included. **b.** *N. benthamiana* leaves transiently co-expressing various genes were extracted and analysed using HPLC-MS, representative (n=6) extracted ion chromatograms (EIC) and MS/MS spectra are shown. EIC displayed are for *m/z* 955.4539, the calculated mass of the [M-H]⁻ adduct of **7**. The negative controls used were extracts from *N. benthamiana* leaves co-expressing only *AstHMGR* (tHMGR control) or co-expressing the *S. officinalis* genes required to produce (**6**) (*tHMGR, SobAS1*, *CYP716A379, CYP72A984*, *SoCSL1* and *UGT73DL1*) (QA-Di). The additional activity of *UGT73CC6* produced a peak corresponding to **7**, identified by comparison with an authentic QA-Tri standard.

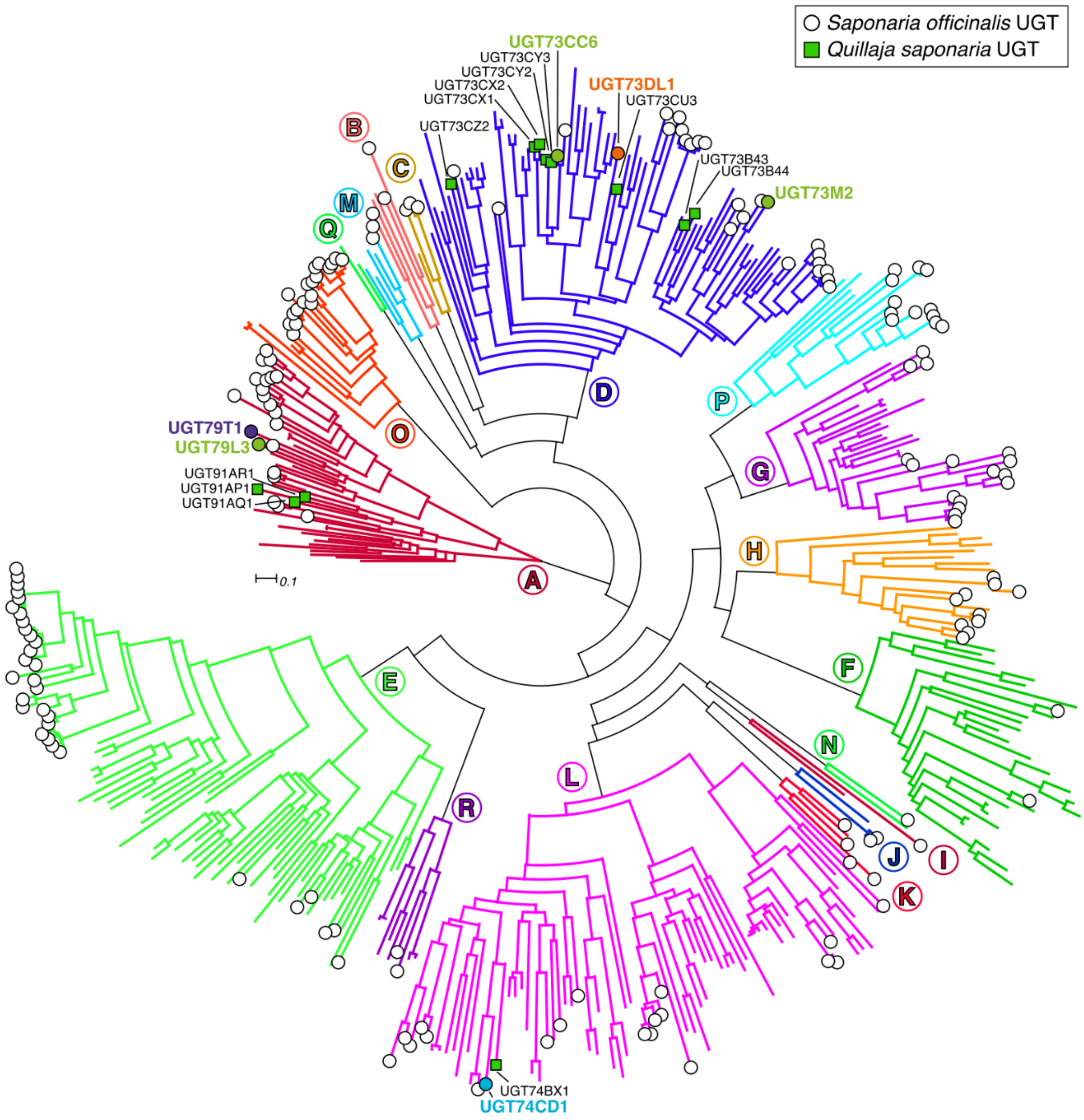

Supplementary Fig. 33. Phylogenetic analysis of UGT candidates found in soapwort. Genome mining, alignment of protein sequences and generation of maximum likelihood phylogeny was carried out as descried in methods. Sequences from *S. officinalis* are indicated with white circles and functional saponin UGTs from *Q. saponaria*[9] are labelled with green squares. UGTs indicated with bold and coloured names are functional saponin biosynthetic genes as discussed in the main text. Other genes included in the alignment and phylogeny are from [10], and to classify the mined UGTs into the labelled clades are from [11]. Different UGT clades are labelled by circled letters. The scale bar indicates the number of amino acid substitutions per site.

a

QA-TriF (C53H82O24)

Exact mass [M]	1102.5196
[M-H]⁻	1101.5118
[M-146-H]⁻	955.4539
[M-146-132-H]⁻	823.4116
[M-176-162-132-146-H]⁻	485.3267

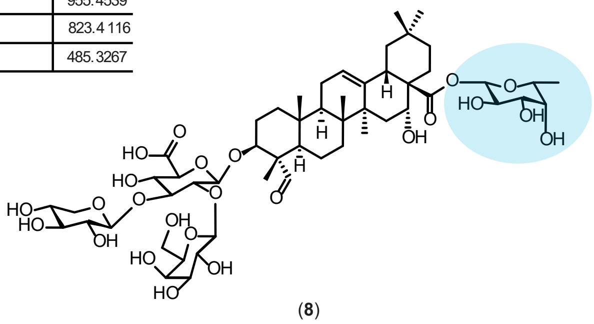

(**8**)

b

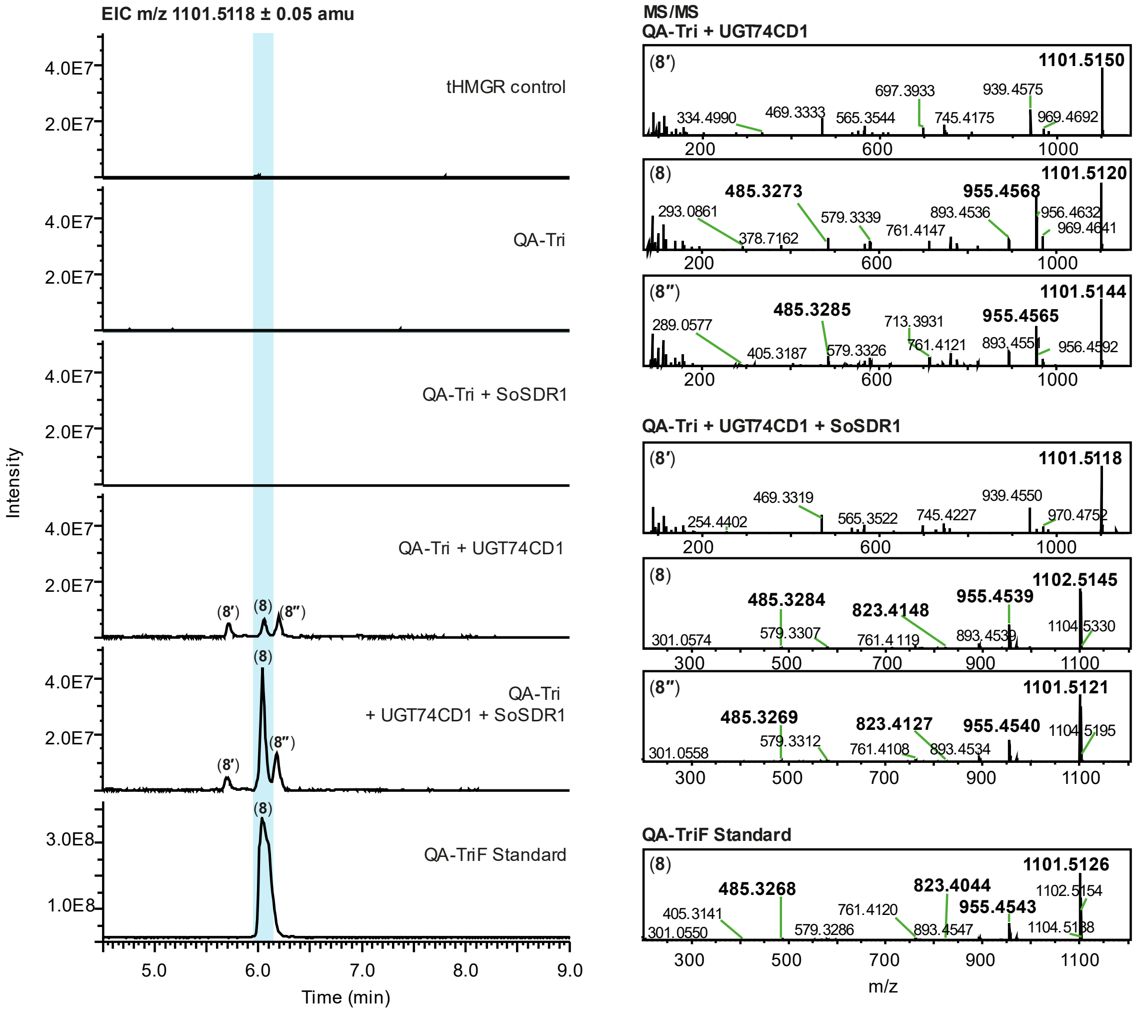

Supplementary Fig. 34. Characterization of UGT74CD1 and SoSDR. a. Structure of 3-*O*-{β-D-xylopyranosyl-(1→3)-[β-D-galactopyranosyl-(1→2)]-β-D-glucopyranosiduronic acid}-28-*O*-{β-D-fucopyranosyl ester}-quillaic acid (QA-TriF, **8**), product of UGT74CD1 when acting in combination with *S. officinalis* enzymes required for production of **7**. Modification performed by UGT74CD1 has been highlighted and a table showing relevant calculated adducts and fragments of **8** is included. **b.** *N. benthamiana* leaves transiently co-expressing various genes were extracted and analysed using HPLC-MS, representative (n=6) extracted

ion chromatograms (EIC) and MS/MS spectra are shown. EIC displayed are for *m/z* 1101.5118, the calculated mass of the $[M\text{-}H]^-$ adduct of **8**. The negative controls used were extracts from *N. benthamiana* leaves co-expressing only *AstHMGR* (tHMGR control) or co-expressing the *S. officinalis* genes required to produce **7** (*tHMGR*, *SobAS1*, *CYP716A379, CYP72A984*, *SoCSL1*, *UGT73DL1* and *UGT73CC6*) (QA-Tri). The additional activity of UGT74CD1 produced a peak corresponding to **8**, identified by comparison with an authentic standard. Addition of SoSDR1 in combination with UGT74CD1 increased yields of **8** significantly but was not capable of producing **8** in the absence of UGT74CD1.

a

QA-TriFR (C59H92O28)

Exact mass [M]	1248.5775
$[M-H]^-$	1247.5679
$[M-132-H]^-$	1115.5274
$[M-146-146-H]^-$	955.4539
$[M-146-146-132-H]^-$	823.4116
$[M-176-162-132-146-146-H]^-$	485.3267

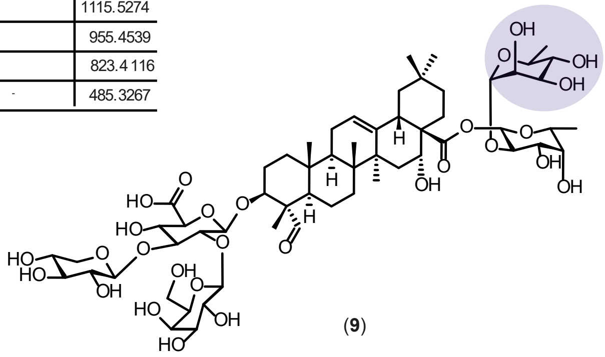

(**9**)

b

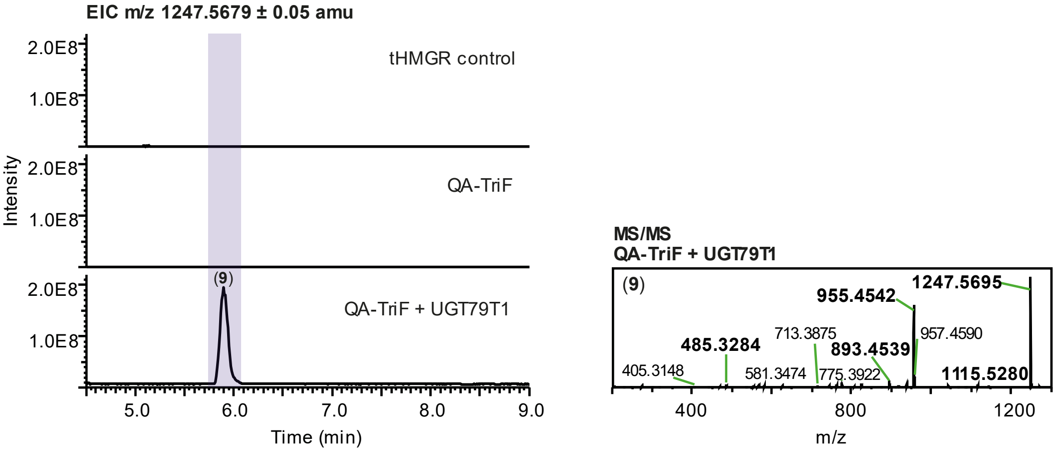

Supplementary Fig. 35. Characterization of UGT79T1. **a.** Structure of 3-*O*-{β-D-xylopyranosyl-(1→3)-[β-D-galactopyranosyl-(1→2)]-β-D-glucopyranosiduronic acid}-28-*O*-{α-L-rhamnopyranosyl-(1→2)-β-D-fucopyranosyl ester}-quillaic acid (QA-TriFR, **9**), the anticipated product of UGT79T1 when acting in combination with the *S. officinalis* enzymes required for production of **8**). Expected modification performed by UGT79T1 has been highlighted and a table showing relevant calculated adducts and fragments of **9** included. **b.** *N. benthamiana* leaves transiently co-expressing various genes were extracted and analysed using HPLC-MS, representative (n=6) extracted ion chromatograms (EIC) and MS/MS spectra are shown. EIC displayed are for *m/z* 1247.5679, the calculated mass of the $[M-H]^-$ adduct of **9**. The negative controls used were extracts from *N. benthamiana* leaves co-expressing only *AstHMGR* (tHMGR control) or co-expressing the *S. officinalis* genes required to produce **8** (*tHMGR*, *SobAS1*, *CYP716A379*, *CYP72A984*, *SoCSL1*, *UGT73DL1*, *UGT73CC6*, *UGT74CD1* and *SoSDR1*) (QA-TriF). The additional activity of *SoUGT79T1* produced a peak corresponding to **9** based on MS/MS spectra, and later confirmed by comparison of downstream products to authentic standards.

a

QA-TriFRX (C64H100O32)	
Exact mass [M]	1380.6198
[M-H]-	1379.6 119
[M-132-H] -	1247.5694
[M-146-146-132-H] -	955.4539
[M-132-146-146-132-H] -	823.4 116
[M-176-162-132-146-146-132-H] -	485.3267

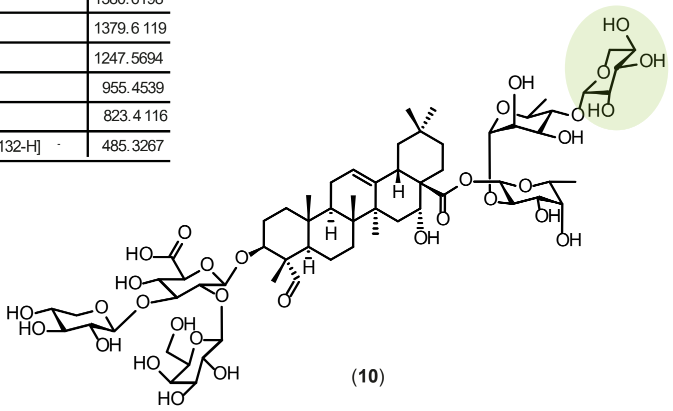

(**10**)

b

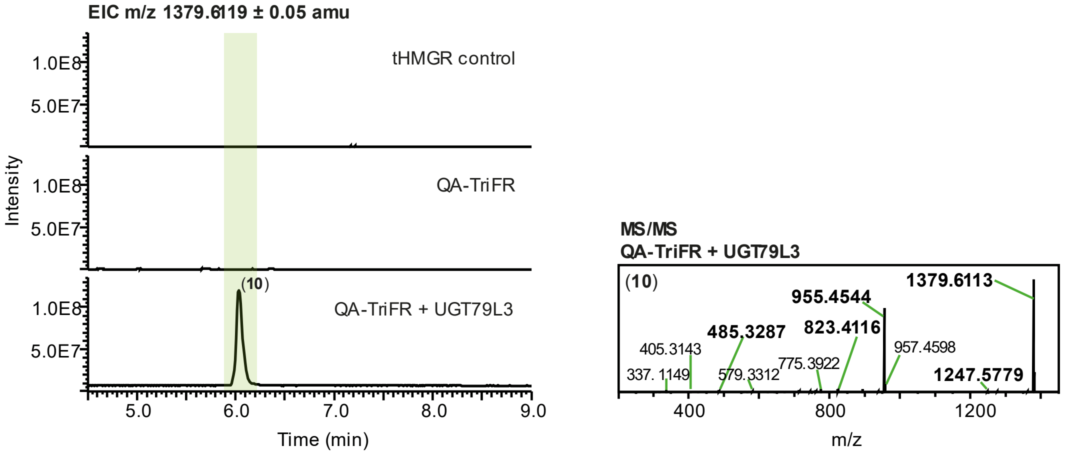

Supplementary Fig. 36. Characterization of UGT79L3. a. Structure of 3-*O*-{β-D-xylopyranosyl-(1→3)-[β-D-galactopyranosyl-(1→2)]-β-D-glucopyranosiduronic acid}-28-*O*-{β-D-xylopyranosyl-(1→4)-α-L-rhamnopyranosyl-(1→2)-β-D-fucopyranosyl ester}-quillaic acid (QA-TriFRX, **10**), the anticipated product of UGT79L3 when acting in combination with the *S. officinalis* enzymes putatively required for production of **9**. Predicted modification performed by UGT79L3 has been highlighted and a table showing relevant calculated adducts and fragments of **10** included. **b.** *N. benthamiana* leaves transiently co-expressing various genes were extracted and analysed using HPLC-MS, representative (n=6) extracted ion chromatograms (EIC) and MS/MS spectra are shown. EIC displayed are for *m/z* 1379.6119, the calculated mass of the [M-H]$^-$ adduct of **10**. The negative controls used were extracts from *N. benthamiana* leaves co-expressing only *AstHMGR* (tHMGR control) or co-expressing the *S. officinalis* genes predicted to produce **9** (*tHMGR*, *SobAS1*, *CYP716A379, CYP72A984, SoCSL1*, *UGT73DL1*, *UGT73CC6*, *UGT74CD1*, *SoSDR1* and *UGT79T1*) (QA-TriFR). The additional activity of UGT79L3 produced a peak putatively identified as **10** based on MS/MS spectra, and later confirmed by comparison of downstream products to authentic standards.

a

QA-TriFRXX (C69H108O36)

Exact mass [M]	1512.6620
[M-H]-	1511.6542
[M-132-H]-	1379.6 119
[M-176-162-132-146-146-132-132-H]-	955.4539

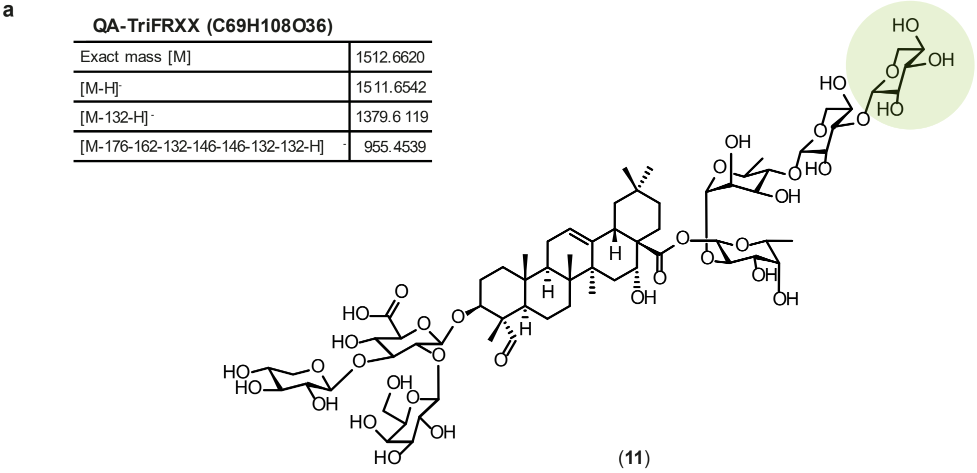

(11)

b

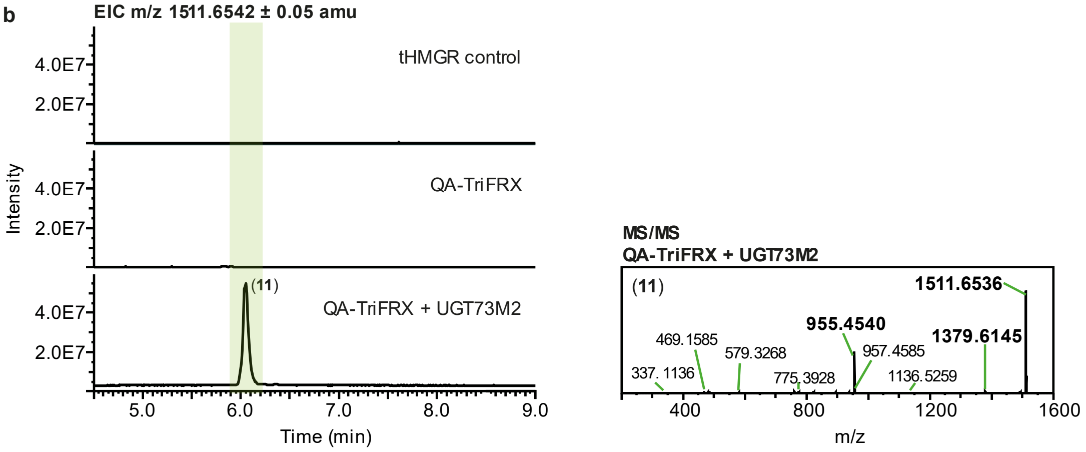

Supplementary Fig. 37. Characterization of UGT73M2. a. Structure of 3-*O*-{β-D-xylopyranosyl-(1→3)-[β-D-galactopyranosyl-(1→2)]-β-D-glucopyranosiduronic acid}-28-*O*-{β-D-xylopyranosyl-(1→3)-β-D-xylopyranosyl-(1→4)-α-L-rhamnopyranosyl-(1→2)-β-D-fucopyranosyl ester}-quillaic acid (QA-TriFRXX, **11**), the anticipated product of UGT73M2 when acting in combination with the *S. officinalis* enzymes putatively required for production of **10**. Expected modification performed by UGT73M2 has been highlighted and a table showing relevant calculated adducts and fragments of **11** included. **b.** *N. benthamiana* leaves transiently co-expressing various genes were extracted and analysed using HPLC-MS, representative (n=6) extracted ion chromatograms (EIC) and MS/MS spectra are shown. EIC displayed are for *m/z* 1511.6542, the calculated mass of the [M-H]- adduct of **11**. The negative controls used were extracts from *N. benthamiana* leaves co-expressing only *AstHMGR* (tHMGR control) or co-expressing the *S. officinalis* genes predicted to produce **10** (*tHMGR*, *SobAS1*, *CYP716A379*, *CYP72A984*, *SoCSL1*, *UGT73DL1*, *UGT73CC6*, *UGT74CD1*, *SoSDR1*, *UGT79T1* and *UGT79L3*) (QA-TriFRX). The additional activity of UGT73M2 produced a peak putatively identified as **11** based on MS/MS spectra, and later confirmed by comparison of downstream products to authentic standards.

a

QA-TriF(Q)RXX ($C_{75}H_{118}O_{40}$)	
Exact mass [M]	1658.7199
$[M-H]^-$	1657.7121
$[M-132-H]^-$	1525.6699
$[M-176-162-132-146-146-132-132-146-H]^-$	955.4539

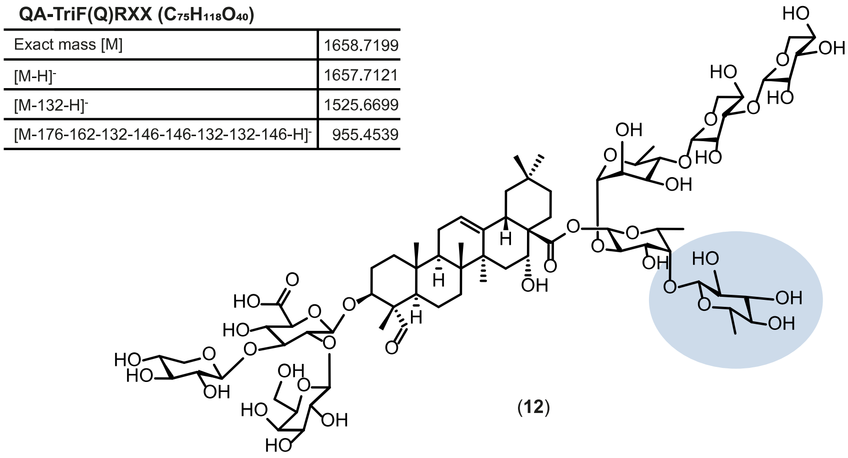

(**12**)

b

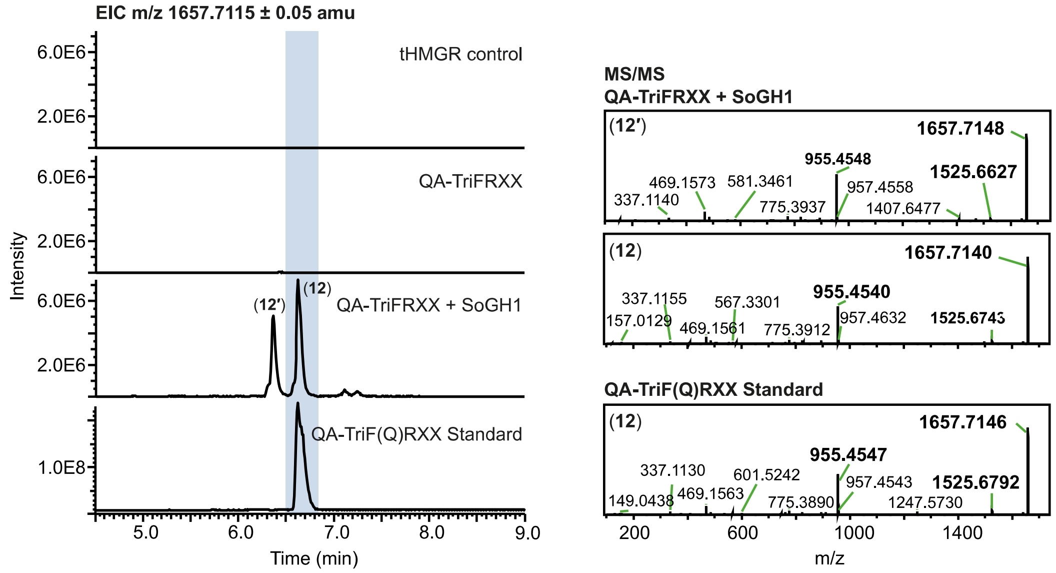

c

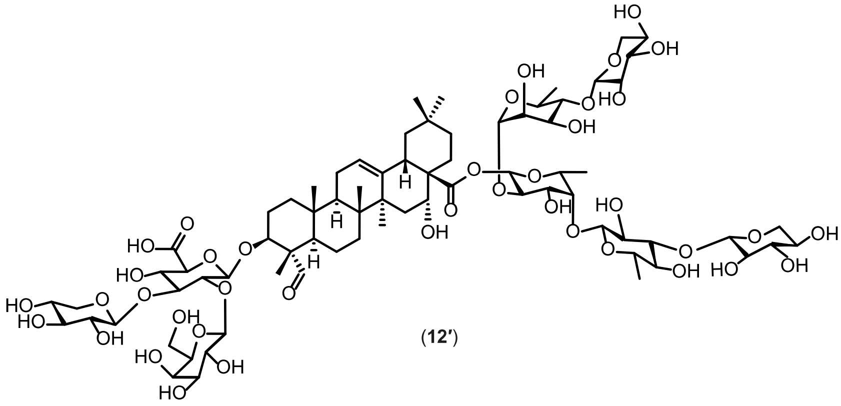

(**12′**)

Supplementary Fig. 38 Characterization of SoGH1. a. Structure of 3-*O*-{β-D-xylopyranosyl-(1→3)-[β-D-galactopyranosyl-(1→2)]-β-D-glucopyranosiduronic acid}-28-*O*-{β-D-xylopyranosyl-(1→3)-β-D-xylopyranosyl-(1→4)-α-L-rhamnopyranosyl-(1→2)-[β-D-quinovopyranosyl-(1→4)]-β-D-fucopyranosyl ester}-quillaic acid (QA-TriF(Q)RXX, **12**), a product of SoGH1 when acting in combination with the *S. officinalis* enzymes expected to be

required for production of **11**. Expected modification performed by SoGH1 has been highlighted and a table showing relevant calculated adducts and fragments of **12** included. **b.** *N. benthamiana* leaves transiently co-expressing various genes were extracted and analysed using HPLC-MS, representative (n=6) extracted ion chromatograms (EIC) and MS/MS spectra are shown. EIC displayed are for *m/z* 1657.7115, the calculated mass of the $[M-H]^-$ adduct of **12**. The negative controls used were extracts from *N. benthamiana* leaves co-expressing only *AstHMGR* (tHMGR control) or co-expressing the *S. officinalis* genes proposed to produce **11** (*tHMGR*, *SobAS1*, *CYP716A379*, *CYP72A984*, *SoCSL1*, *UGT73DL1*, *UGT73CC6*, *UGT74CD1*, *SoSDR1*, *UGT79T1*, *UGT79L3* and *UGT73M2*) (QA-TriFRXX). The additional activity of SoGH1 produced a peak corresponding to **12** confirmed by comparison to authentic standard. Further evidence is provided in Supplementary Fig. 50. **c.** Structure of 3-*O*-{β-D-xylopyranosyl-(1→3)-[β-D-galactopyranosyl-(1→2)]-β-D-glucopyranosiduronic acid}-28-*O*-{β-D-xylopyranosyl-(1→4)-α-L-rhamnopyranosyl-(1→2)-[β-D-xylopyranosyl-(1→3)-β-D-quinovopyranosyl-(1→4)]-β-D-fucopyranosyl ester}-quillaic acid, the predicted structure of **12′**. Based on the matching MS/MS fragmentation pattern to **12**, **12′** may be a positional isomer of **12**.

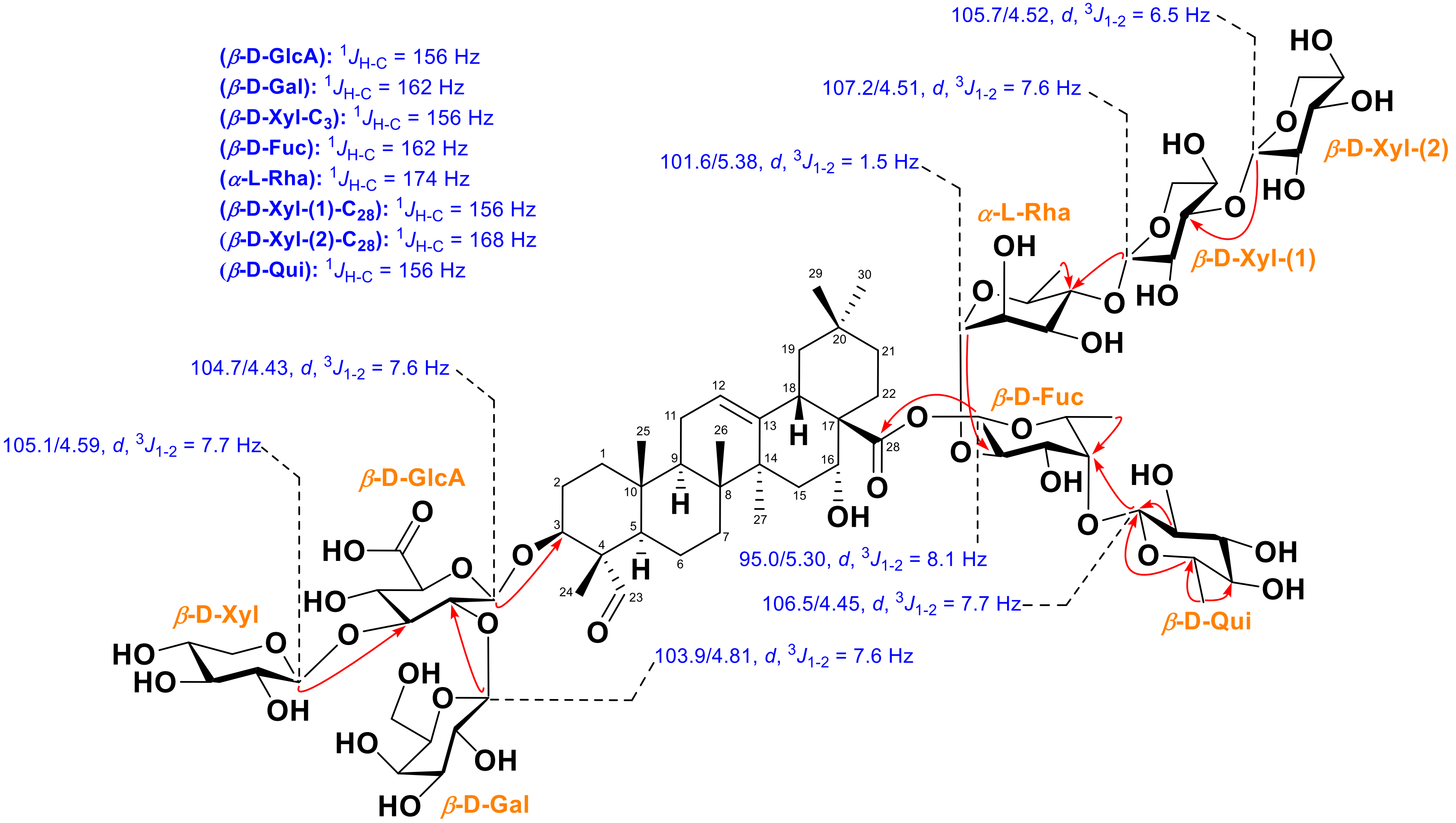

Supplementary Fig. 39. Key HMBC and coupled HSQC coupling constants ($^{1}J_{H\text{-}C}$) recorded for QA-TriF(Q)RXX (12) purified from soapwort flowers. Red arrows represent H→C.

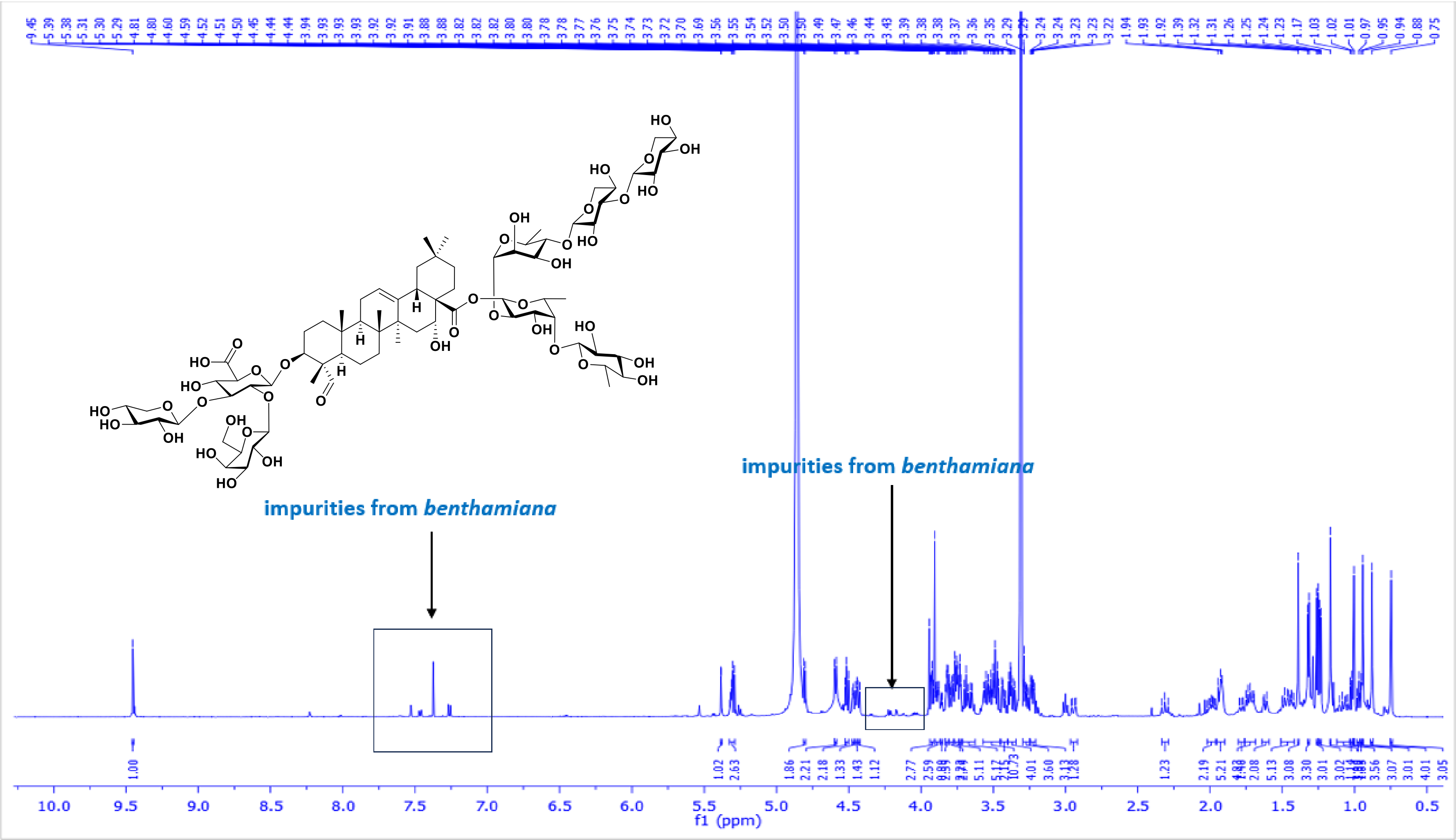

Supplementary Fig. 40. **^{1}HNMR spectrum of QA-TriF(Q)RXX (12) standard material purified from soapwort flowers, recorded in MeOH-*d*4, 600 MHz.**

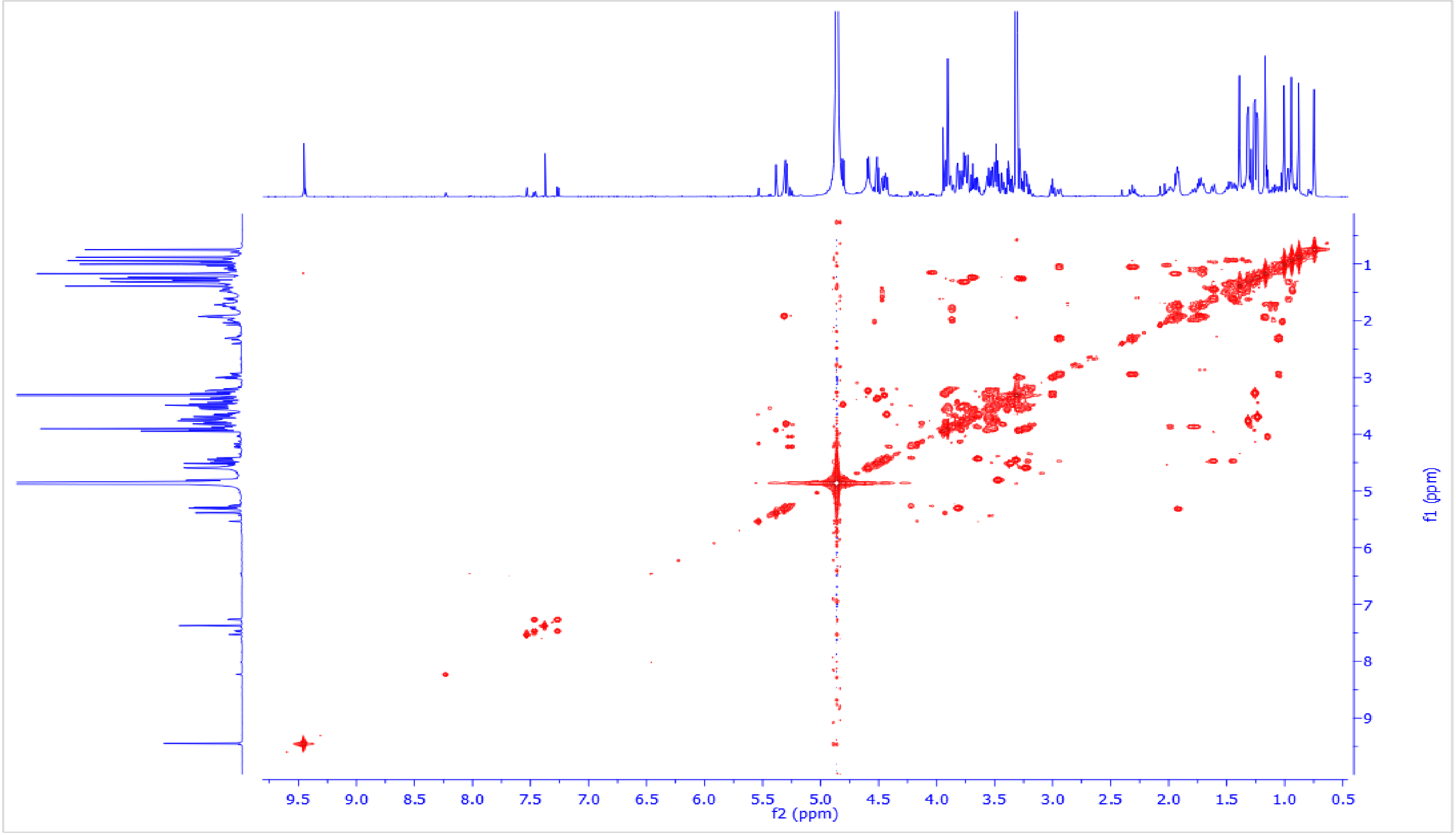

Supplementary Fig. 41. **^{1}H-^{1}H COSY spectrum of QA-TriF(Q)RXX (12) standard material purified from soapwort flowers, recorded in MeOH-*d*$_{4}$, 600 MHz.**

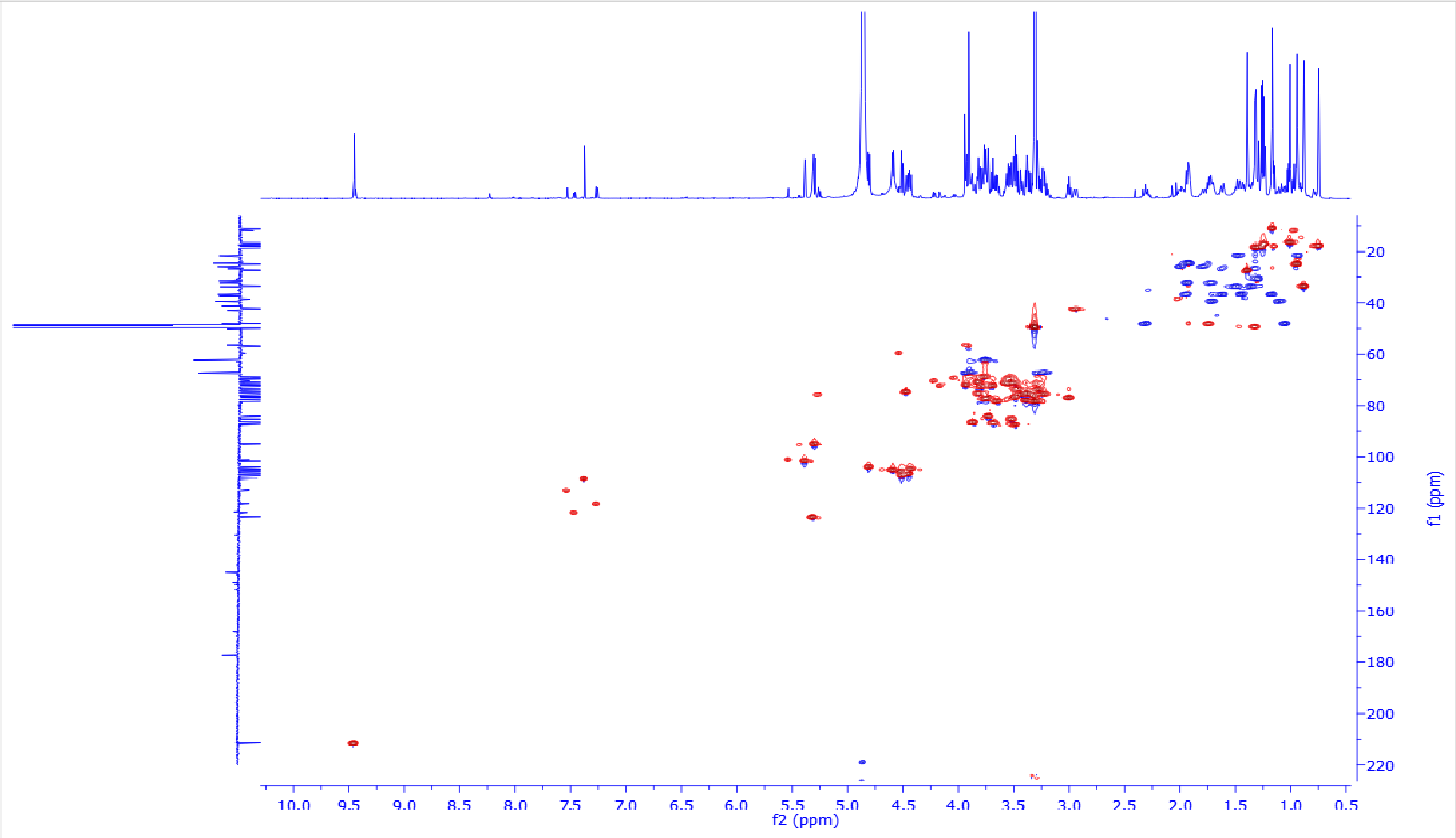

Supplementary Fig. 42. ^{1}H-^{13}C **HSQC spectrum of QA-TriF(Q)RXX (12) standard material purified from soapwort flowers, recorded in MeOH-*d*$_4$, 600/150 MHz.**

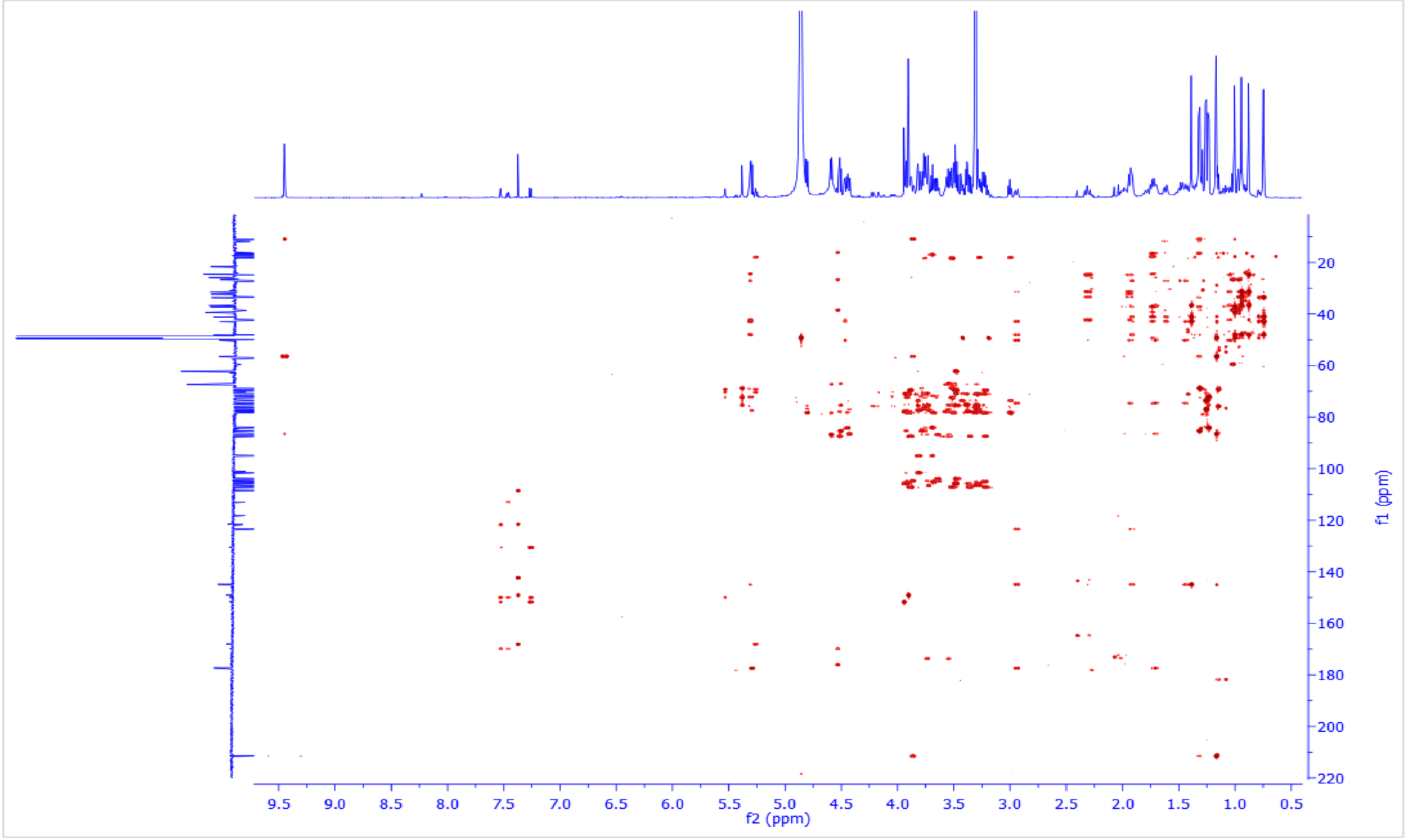

Supplementary Fig. 43. ^{1}H-^{13}C HMBC spectrum of QA-TriF(Q)RXX (12) standard material purified from soapwort flowers, recorded in MeOH-d_4, 600/150 MHz.

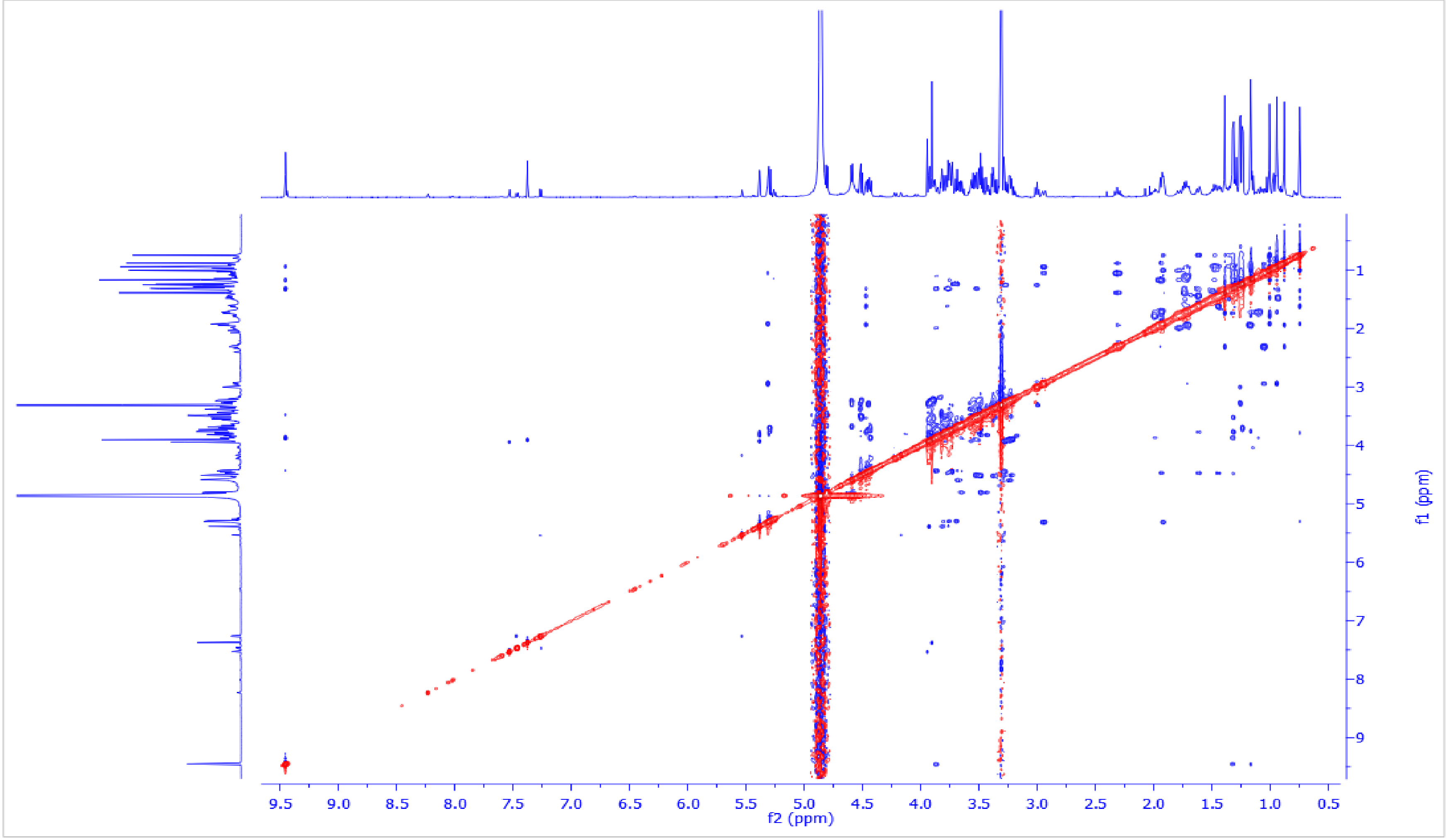

Supplementary Fig. 44. ^{1}**H-**^{1}**H ROESY spectrum of QA-TriF(Q)RXX (12) standard material purified from soapwort flowers, recorded in MeOH-*d*4, 600 MHz.**

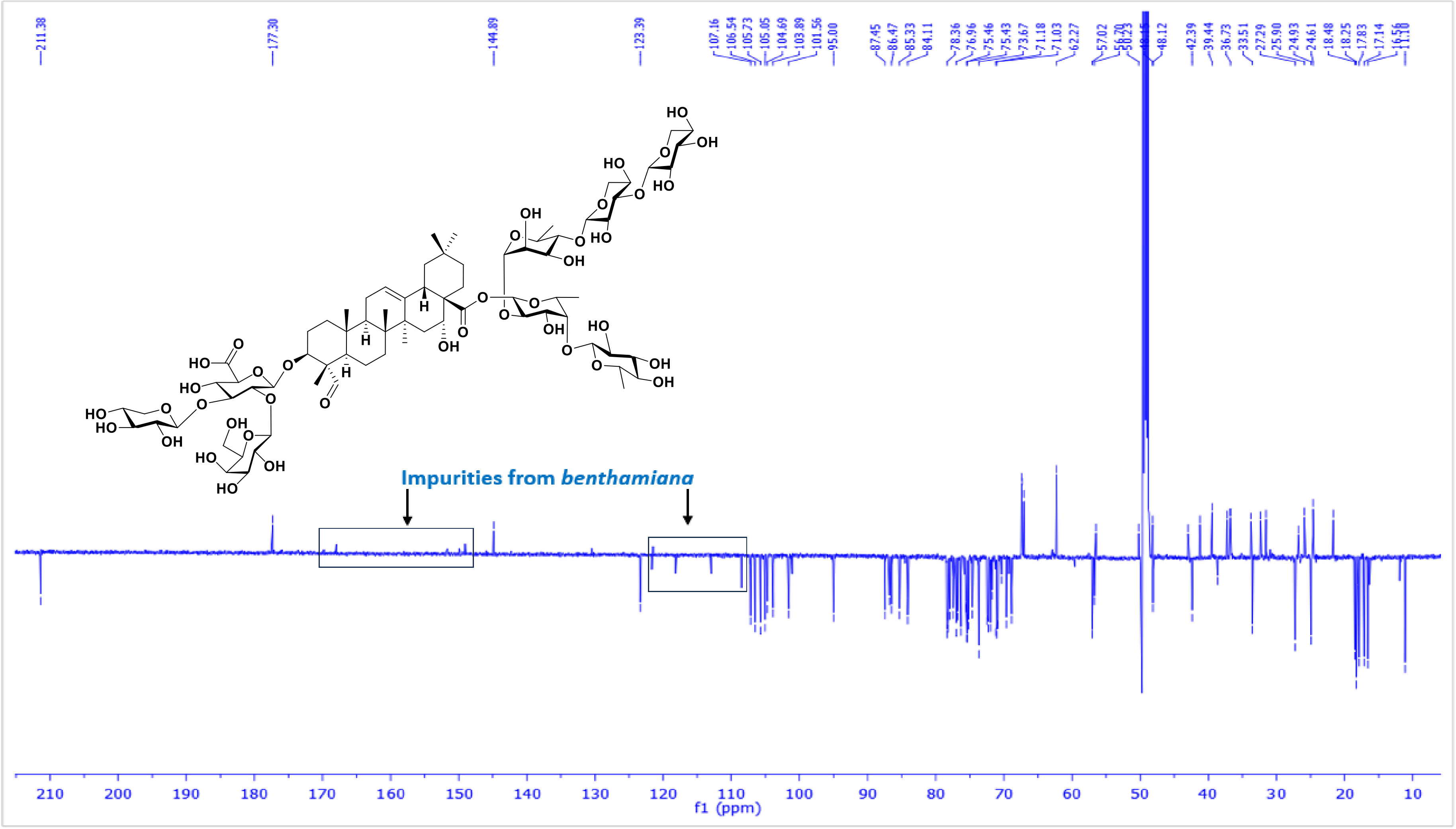

Supplementary Fig. 45. DEPTQ-135 spectrum of QA-TriF(Q)RXX (12) standard material purified from soapwort flowers recorded in MeOH-*d*4, 150 MHz.

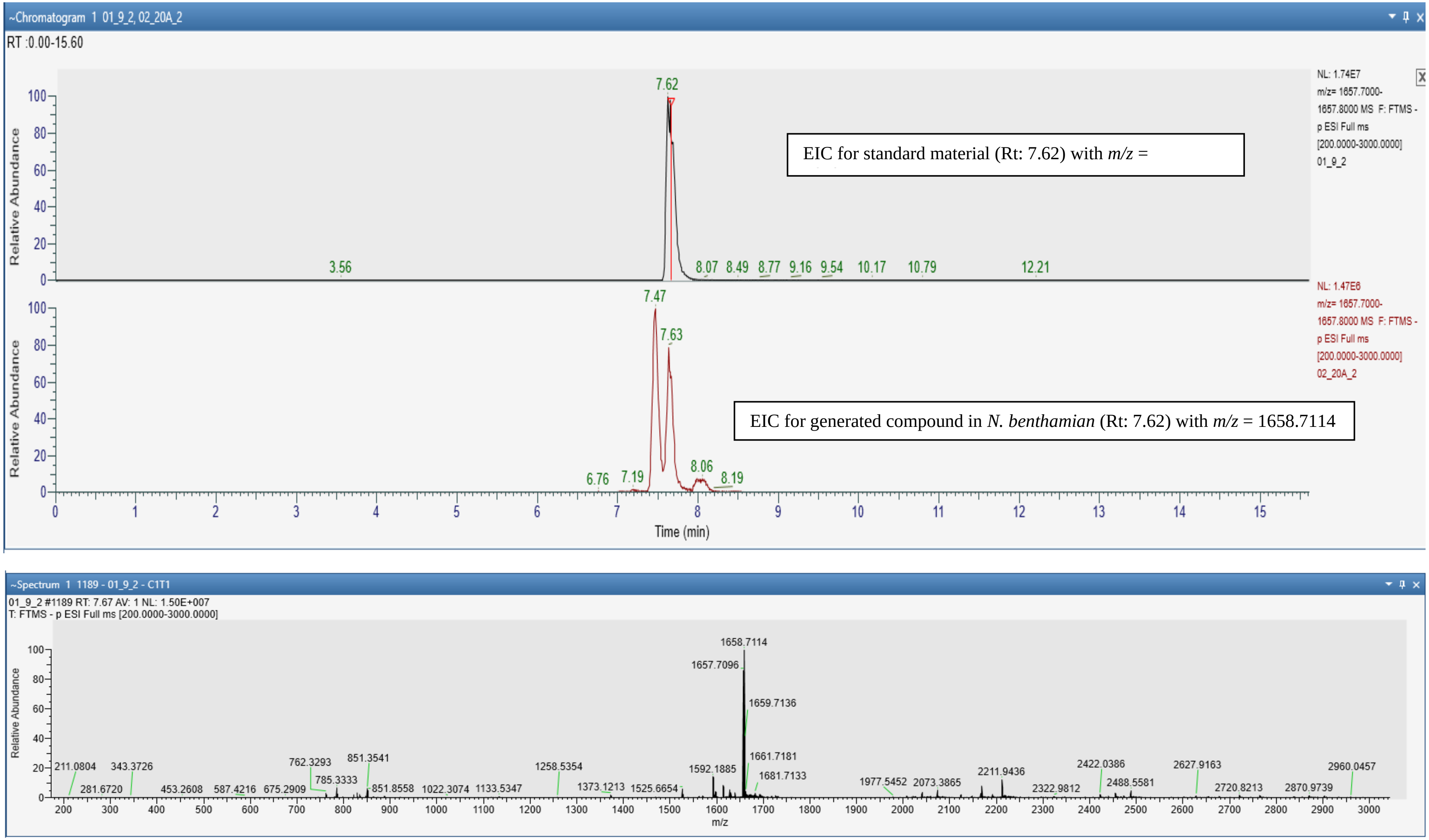

Supplementary Fig. 46. LC-MS overlay between QA-TriF(Q)RXX (12) standard material and one generated in *N. benthamiana*.

^{1}H-NMR

	Parameter	Value
1	Data File Name	/ / Ji-cfs2/ shared/ Research-Groups/ Anne-Osbourn/ NMR600/ data/ eldemer/ nmr/ 20220915-AE-JJ-1657-F9/ 11/ pdata/ 1/ 1r
2	Title	20220915-AE-JJ-1657-F9
3	Comment	
4	Origin	Bruker BioSpin GmbH
5	Owner	nmrsu
6	Site	
7	Spectrometer	Avance Neo
8	Author	
9	Solvent	MeOD
10	Temperature	298.0
11	Pulse Sequence	zg30
12	Number of Scans	256
13	Receiver Gain	101
14	Relaxation Delay	0.1000
15	Pulse Width	8.0000
16	Acquisition Time	3.6700
17	Acquisition Date	2022-09-15T09:13:10
18	Modification Date	2022-09-15T09:11:57
19	Spectrometer Frequency	600.18
20	Spectral Width	17857.1
21	Lowest Frequency	-5233.6
22	Nucleus	1H
23	Acquired Size	65536
24	Spectral Size	131072

^{1}H-^{13}C HSQC

	Parameter	Value (f2, f1)
1	Data File Name	/ / Ji-cfs2/ shared/ Research-Groups/ Anne-Osbourn/ NMR600/ data/ eldemer/ nmr/ 20220915-AE-JJ-1657-F9/ 13/ pdata/ 1/ 2rr
2	Title	20220915-AE-JJ-1657-F9
3	Comment	
4	Origin	Bruker BioSpin GmbH
5	Owner	nmrsu
6	Site	
7	Spectrometer	Avance Neo
8	Author	
9	Solvent	MeOD
10	Temperature	298.0
11	Pulse Sequence	hsqcedetgpsisp2.3
12	Number of Scans	25
13	Receiver Gain	101
14	Relaxation Delay	1.5000
15	Pulse Width	8.0000
16	Acquisition Time	0.0532
17	Acquisition Date	2022-09-15T13:37:48
18	Modification Date	2022-09-15T13:36:37
19	Spectrometer Frequency	(600.18, 150.92)
20	Spectral Width	(9615.4, 36222.4)
21	Lowest Frequency	(-608.2, -1237.8)
22	Nucleus	(1H, 13C)
23	Acquired Size	(512, 256)
24	Spectral Size	(1024, 1024)

^{1}H-^{1}H COSY

	Parameter	Value (f2, f1)
1	Data File Name	/ / Ji-cfs2/ shared/ Research-Groups/ Anne-Osbourn/ NMR600/ data/ eldemer/ nmr/ 20220915-AE-JJ-1657-F9/ 12/ pdata/ 1/ 2rr
2	Title	20220915-AE-JJ-1657-F9
3	Comment	
4	Origin	Bruker BioSpin GmbH
5	Owner	nmrsu
6	Site	
7	Spectrometer	Avance Neo
8	Author	
9	Solvent	MeOD
10	Temperature	298.0
11	Pulse Sequence	cosygpppqf.ptype
12	Number of Scans	20
13	Receiver Gain	101
14	Relaxation Delay	0.8874
15	Pulse Width	8.0000
16	Acquisition Time	0.1556
17	Acquisition Date	2022-09-15T10:47:43
18	Modification Date	2022-09-15T10:46:31
19	Spectrometer Frequency	(600.18, 600.18)
20	Spectral Width	(6578.9, 6602.0)
21	Lowest Frequency	(-476.3, -487.4)
22	Nucleus	(1H, 1H)
23	Acquired Size	(1024, 256)
24	Spectral Size	(1024, 1024)

Supplementary Fig. 47. Parameters for (^{1}H, HSQC and COSY) NMR experiments for compound QA-TriF(Q)RXX (12).

Coupled ^1H-^{13}C HSQC

	Parameter	Value (f2, f1)
1	Data File Name	/ / Ji-cfs2/ shared/ Research-Groups/ Anne-Osbourn/ NMR600/ data/ eldemer/ nmr/ 20220915-AE-JJ-1657-F9/ 17/ pdata/ 1/ 2rr
2	Title	20220915-AE-JJ-1657-F9
3	Comment	Cpopled HSQC (plw12=0), NS=40
4	Origin	Bruker BioSpin GmbH
5	Owner	nmrsu
6	Site	
7	Spectrometer	Avance Neo
8	Author	
9	Solvent	MeOD
10	Temperature	298.0
11	Pulse Sequence	hsqcedetgpsisp2.3
12	Number of Scans	40
13	Receiver Gain	101
14	Relaxation Delay	1.0000
15	Pulse Width	8.0000
16	Acquisition Time	0.1434
17	Acquisition Date	2022-09-16T15:23:35
18	Modification Date	2022-09-16T15:22:23
19	Spectrometer Frequency	(600.18, 150.92)
20	Spectral Width	(7142.9, 24902.3)
21	Lowest Frequency	(-751.2, -886.4)
22	Nucleus	(1H, 13C)
23	Acquired Size	(1024, 256)
24	Spectral Size	(2048, 1024)

^1H-^{13}C HMBC

	Parameter	Value (f2, f1)
1	Data File Name	/ / Ji-cfs2/ shared/ Research-Groups/ Anne-Osbourn/ NMR600/ data/ eldemer/ nmr/ 20220915-AE-JJ-1657-F9/ 14/ pdata/ 1/ 2rr
2	Title	20220915-AE-JJ-1657-F9
3	Comment	
4	Origin	Bruker BioSpin GmbH
5	Owner	nmrsu
6	Site	
7	Spectrometer	Avance Neo
8	Author	
9	Solvent	MeOD
10	Temperature	298.0
11	Pulse Sequence	hmbcetgpl2nd.2
12	Number of Scans	30
13	Receiver Gain	101
14	Relaxation Delay	1.5000
15	Pulse Width	8.0000
16	Acquisition Time	0.2621
17	Acquisition Date	2022-09-15T21:30:16
18	Modification Date	2022-09-15T21:29:06
19	Spectrometer Frequency	(600.18, 150.92)
20	Spectral Width	(7812.5, 36223.3)
21	Lowest Frequency	(-917.9, -1259.6)
22	Nucleus	(1H, 13C)
23	Acquired Size	(2048, 512)
24	Spectral Size	(4096, 1024)

^1H-^1H ROESY

	Parameter	Value (f2, f1)
1	Data File Name	/ / Ji-cfs2/ shared/ Research-Groups/ Anne-Osbourn/ NMR600/ data/ eldemer/ nmr/ 20220915-AE-JJ-1657-F9/ 16/ pdata/ 1/ 2rr
2	Title	20220915-AE-JJ-1657-F9
3	Comment	
4	Origin	Bruker BioSpin GmbH
5	Owner	nmrsu
6	Site	
7	Spectrometer	Avance Neo
8	Author	
9	Solvent	MeOD
10	Temperature	298.0
11	Pulse Sequence	roesyadjsphpp.ptg
12	Number of Scans	15
13	Receiver Gain	101
14	Relaxation Delay	1.4962
15	Pulse Width	8.0000
16	Acquisition Time	0.1556
17	Acquisition Date	2022-09-16T12:02:01
18	Modification Date	2022-09-16T12:00:47
19	Spectrometer Frequency	(600.18, 600.18)
20	Spectral Width	(6578.9, 6602.0)
21	Lowest Frequency	(-622.4, -631.6)
22	Nucleus	(1H, 1H)
23	Acquired Size	(1024, 256)
24	Spectral Size	(2048, 1024)

Supplementary Fig. 48. Parameters for (coupled HSQC, HMBC and ROESY) NMR experiments for compound QA-TriF(Q)RXX (12).

DEPTq-135

	Parameter	Value
1	Data File Name	/ / Ji-cfs2/ shared/ Research-Groups/ Anne-Osbourn/ NMR600/ data/ eldemer/ nmr/ 20220915-AE-JJ-1657-F9/ 15/ pdata/ 1/ 1r
2	Title	20220915-AE-JJ-1657-F9
3	Comment	
4	Origin	Bruker BioSpin GmbH
5	Owner	nmrsu
6	Site	
7	Spectrometer	Avance Neo
8	Author	
9	Solvent	MeOD
10	Temperature	298.0
11	Pulse Sequence	deptqgpsp
12	Number of Scans	15000
13	Receiver Gain	101
14	Relaxation Delay	2.0000
15	Pulse Width	12.0000
16	Acquisition Time	0.9175
17	Acquisition Date	2022-09-16T09:52:44
18	Modification Date	2022-09-16T09:51:23
19	Spectrometer Frequency	150.92
20	Spectral Width	35714.3
21	Lowest Frequency	-2530.5
22	Nucleus	13C
23	Acquired Size	32768
24	Spectral Size	32768

Supplementary Fig. 49. Parameters for (DEPTQ-135) NMR experiments for compound QA-TriF(Q)RXX (12).

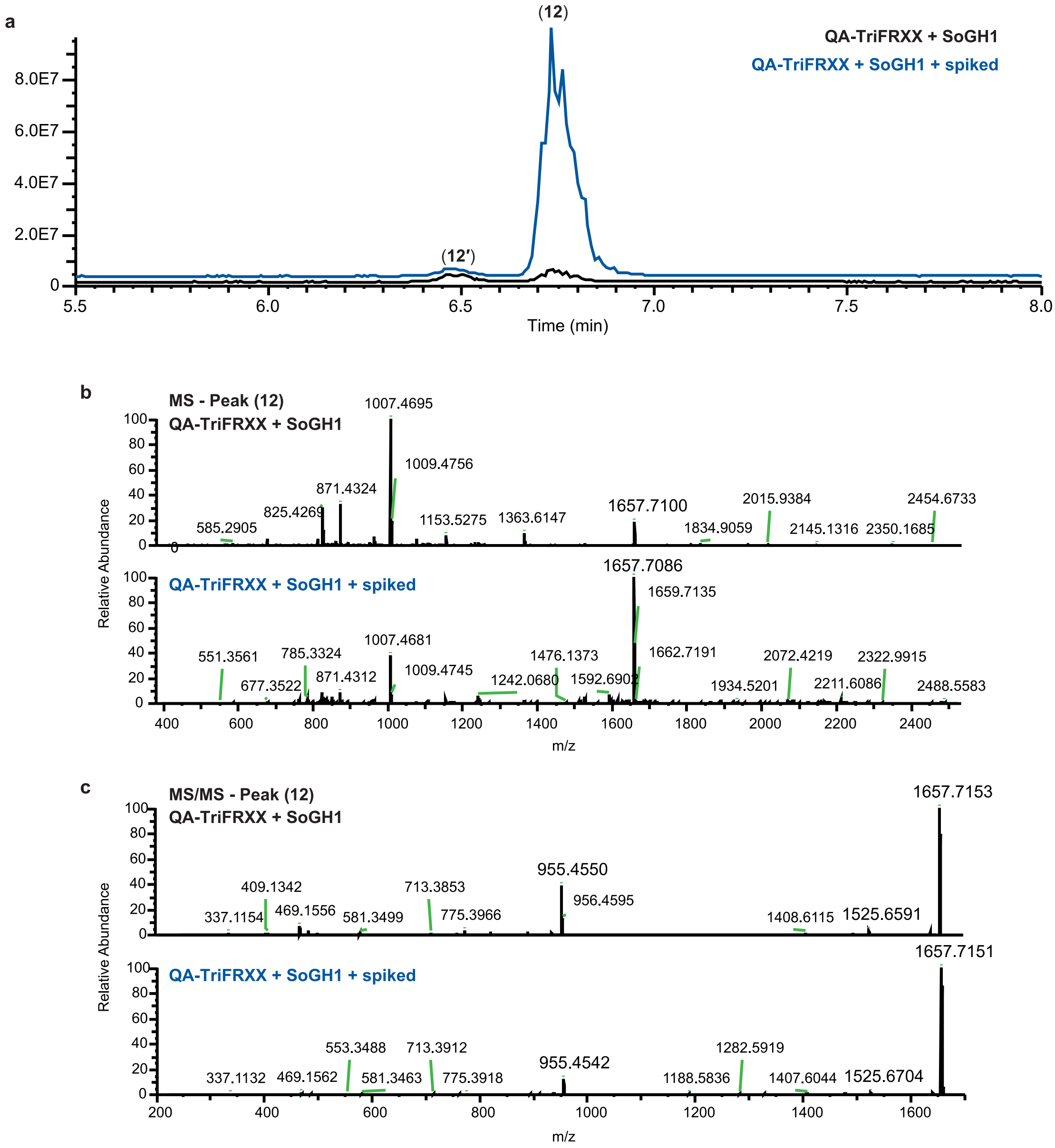

Supplementary Fig. 50. Extracts of *N. benthamiana* leaves producing compound 12 spiked with authentic QA-TriF(Q)RXX (12) standard. a. Extracted ion chromatograms (EIC) of extracts of *N. benthamiana* leaves transiently co-expressing genes proposed to produce **11** (*tHMGR, SobAS1, CYP716A379, CYP72A984, SoCSL1, UGT73DL1, UGT73CC6, UGT74CD1, SoSDR1, UGT79T1, UGT79L3* and *UGT73M2*) (QA-TriFRXX) together with SoGH1, compared with EIC of the same leaf extract spiked with authentic QA-TriF(Q)RXX standard. EIC displayed are for *m/z* 1657.7115, the calculated mass of the [M-H]$^-$ adduct of **12**. **b.** Mass spectra of peak **12**. **c.** MS/MS spectra of peak **12** showing fragmentation pattern.

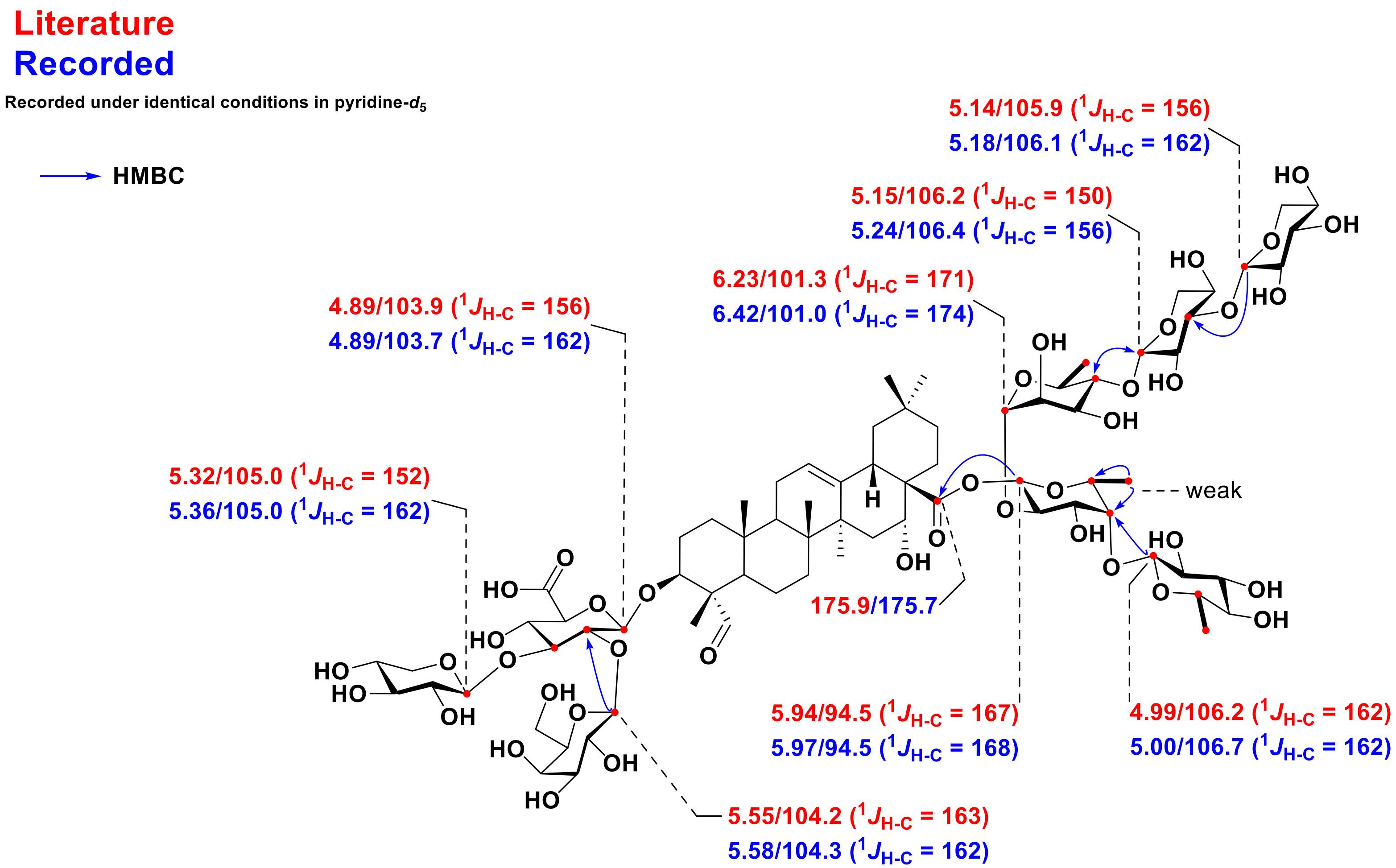

Supplementary Fig. 51. Key HMBC, chemical shifts and coupled HSQC coupling constants ($^1J_{H\text{-}C}$) recorded for QA-TriF(Q)RXX (12) produced in *N. benthamiana*. Blue arrows represent H→C.

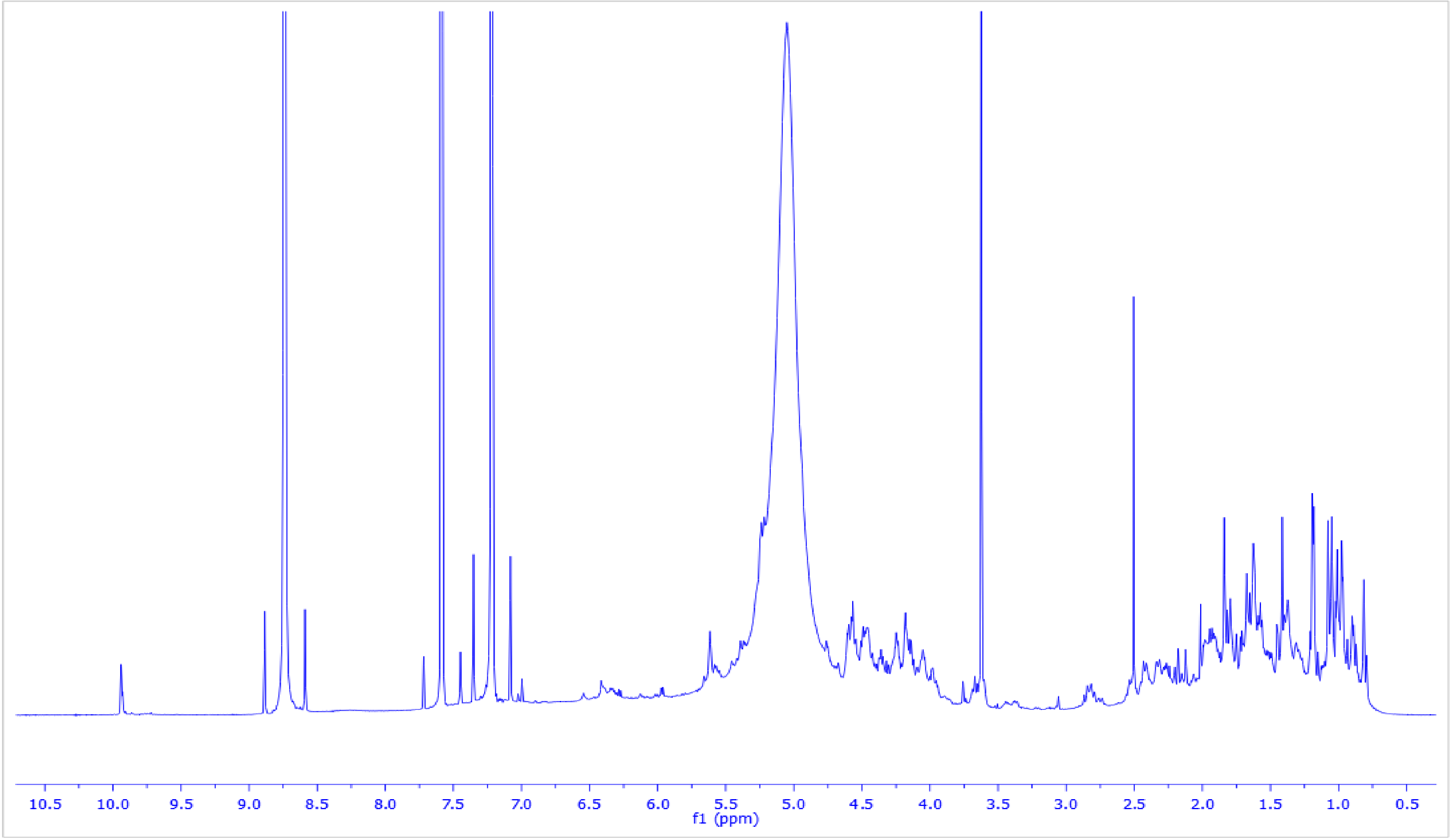

Supplementary Fig. 52. ^{1}H-NMR spectrum of QA-TriF(Q)RXX (12) produced in *N. benthamiana*, recorded in Pyridine-d_5, 600 MHz.

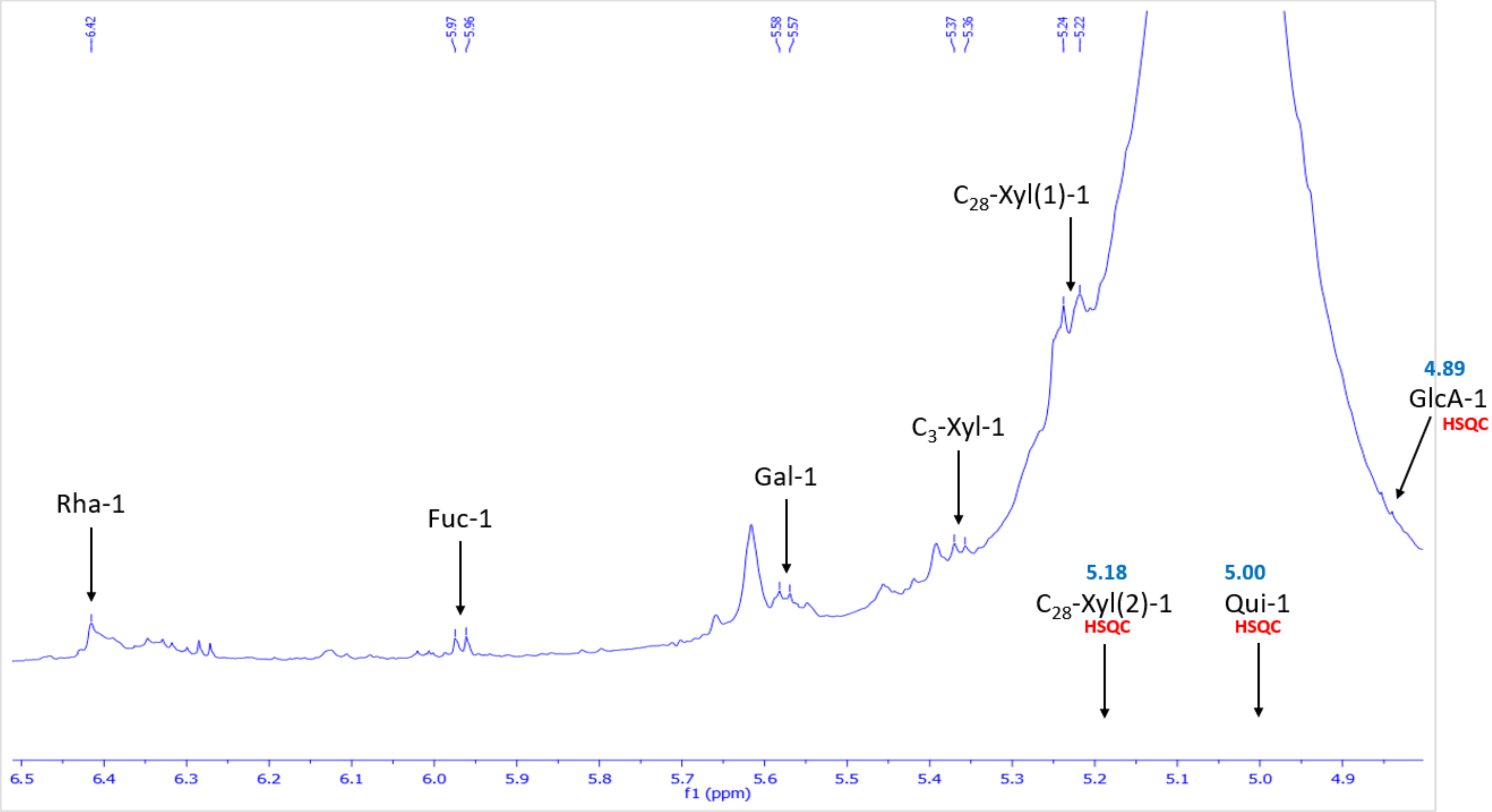

Supplementary Fig. 53. Expanded ^{1}H-NMR spectrum (4.7-6.50 ppm) of QA-TriF(Q)RXX (12) produced in *N. benthamiana*, recorded in Pyridine-*d*5, 600 MHz.

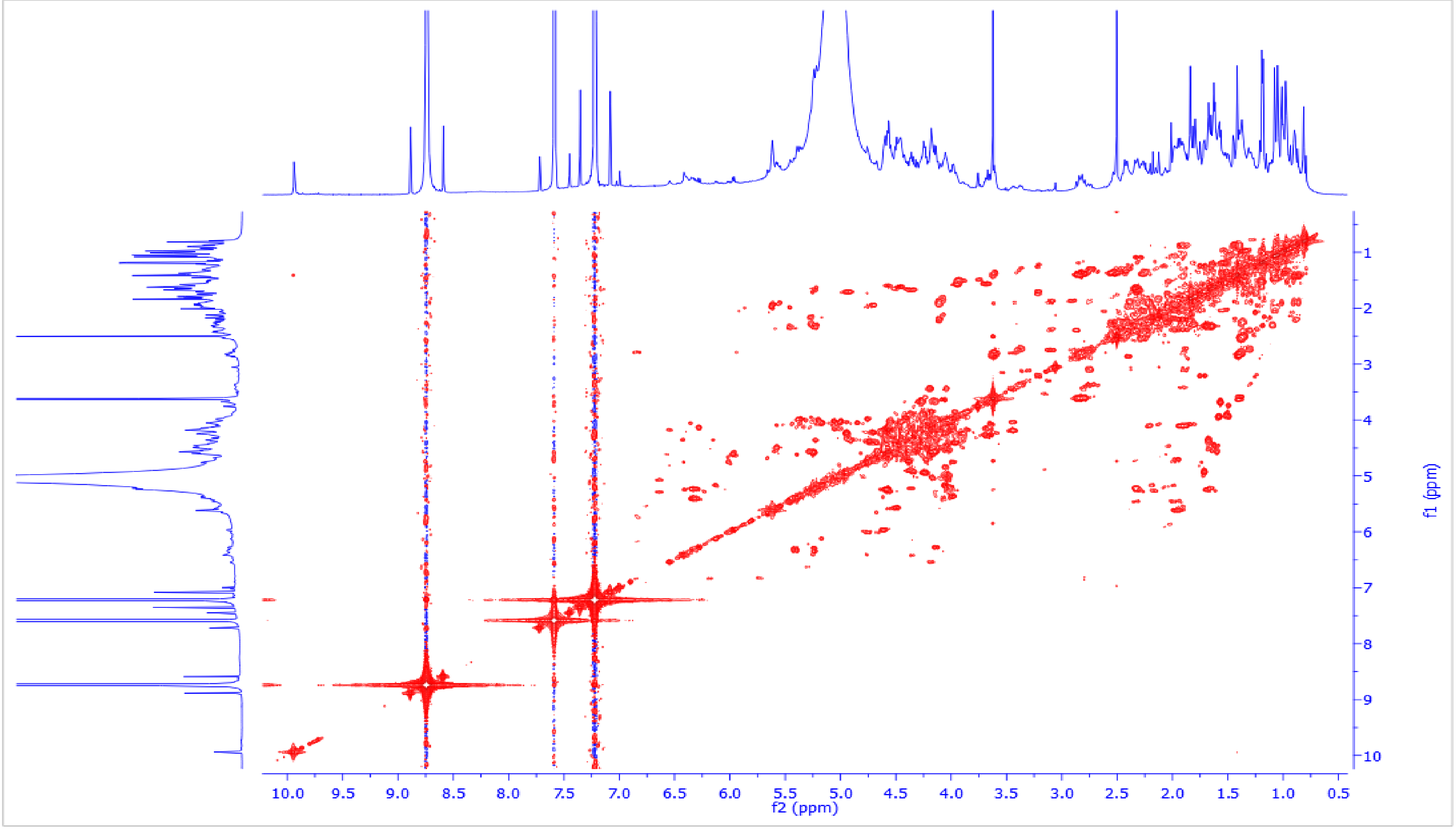

Supplementary Fig. 54. **^{1}H-^{1}H COSY spectrum of QA-TriF(Q)RXX (12) produced in *N. benthamiana*, recorded in Pyridine-d_5, 600 MHz.**

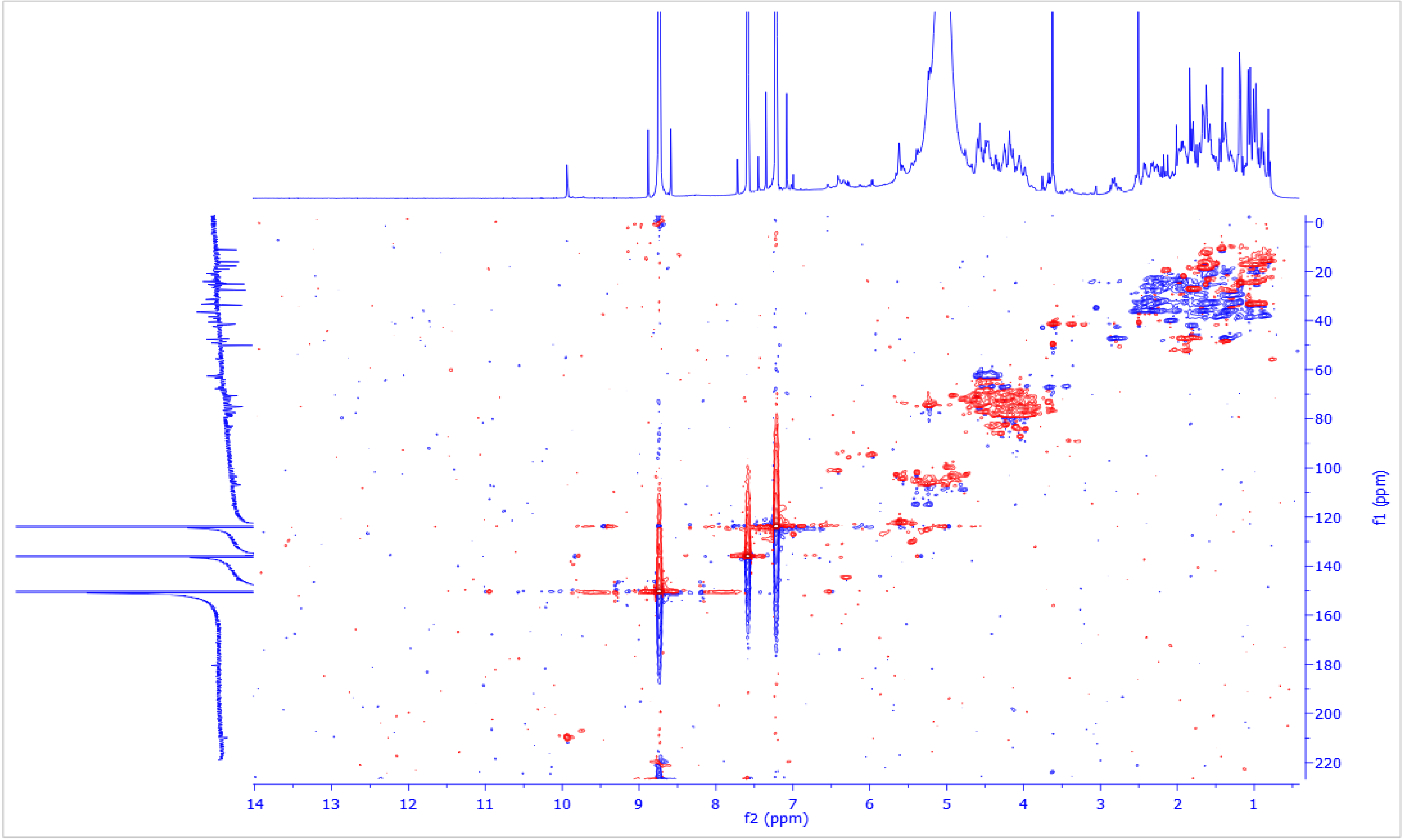

Supplementary Fig. 55. ^{1}H-^{13}C HSQC spectrum of QA-TriF(Q)RXX (12) produced in *N. benthamiana*, recorded in Pyridine-*d*$_5$, 600/150 MHz.

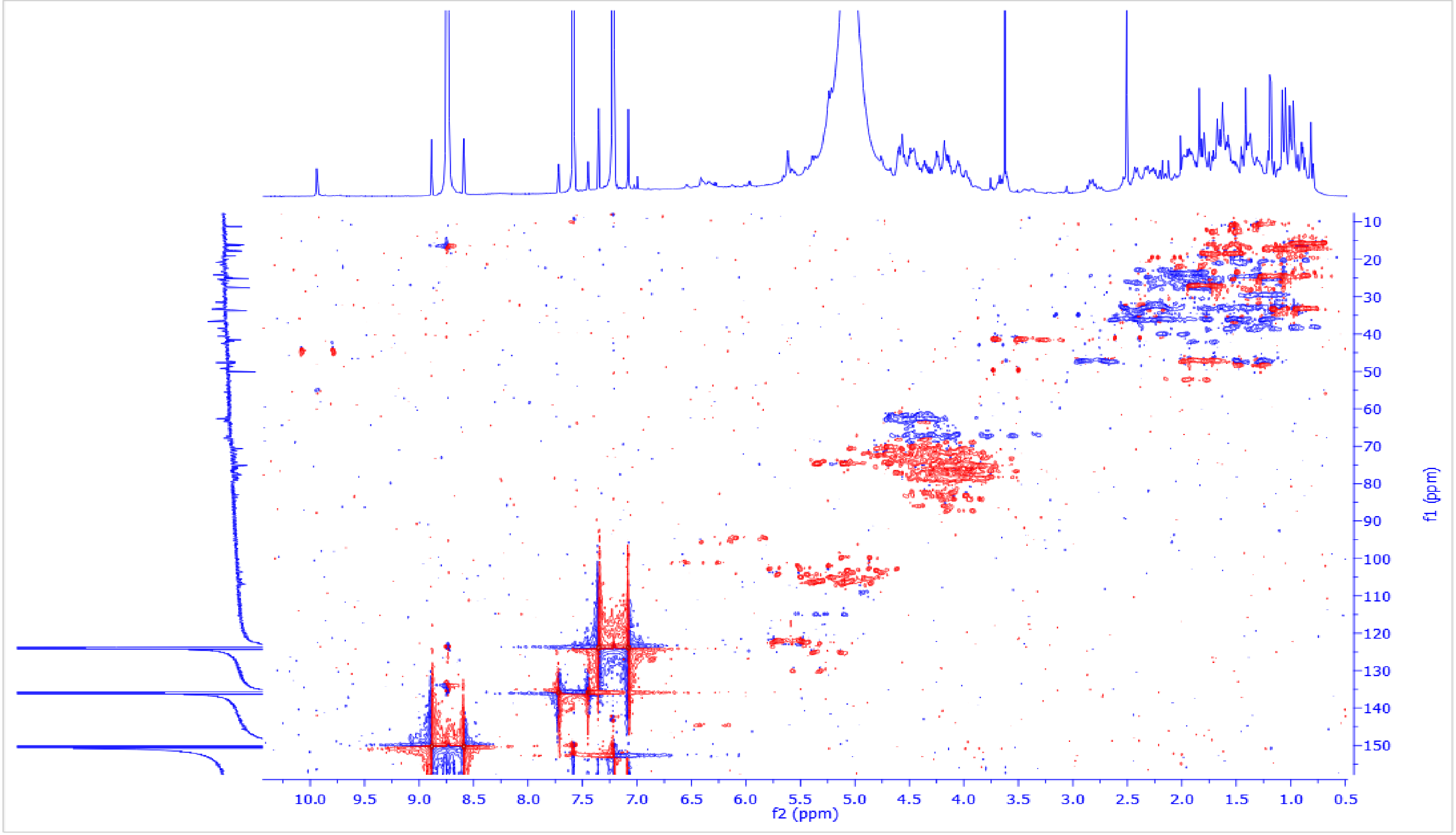

Supplementary Fig. 56. ^{1}H-^{13}C **coupled HSQC spectrum of QA-TriF(Q)RXX (12) produced in** ***N. benthamiana,*** **recorded in Pyridine-*d*5 (600/150 MHz).**

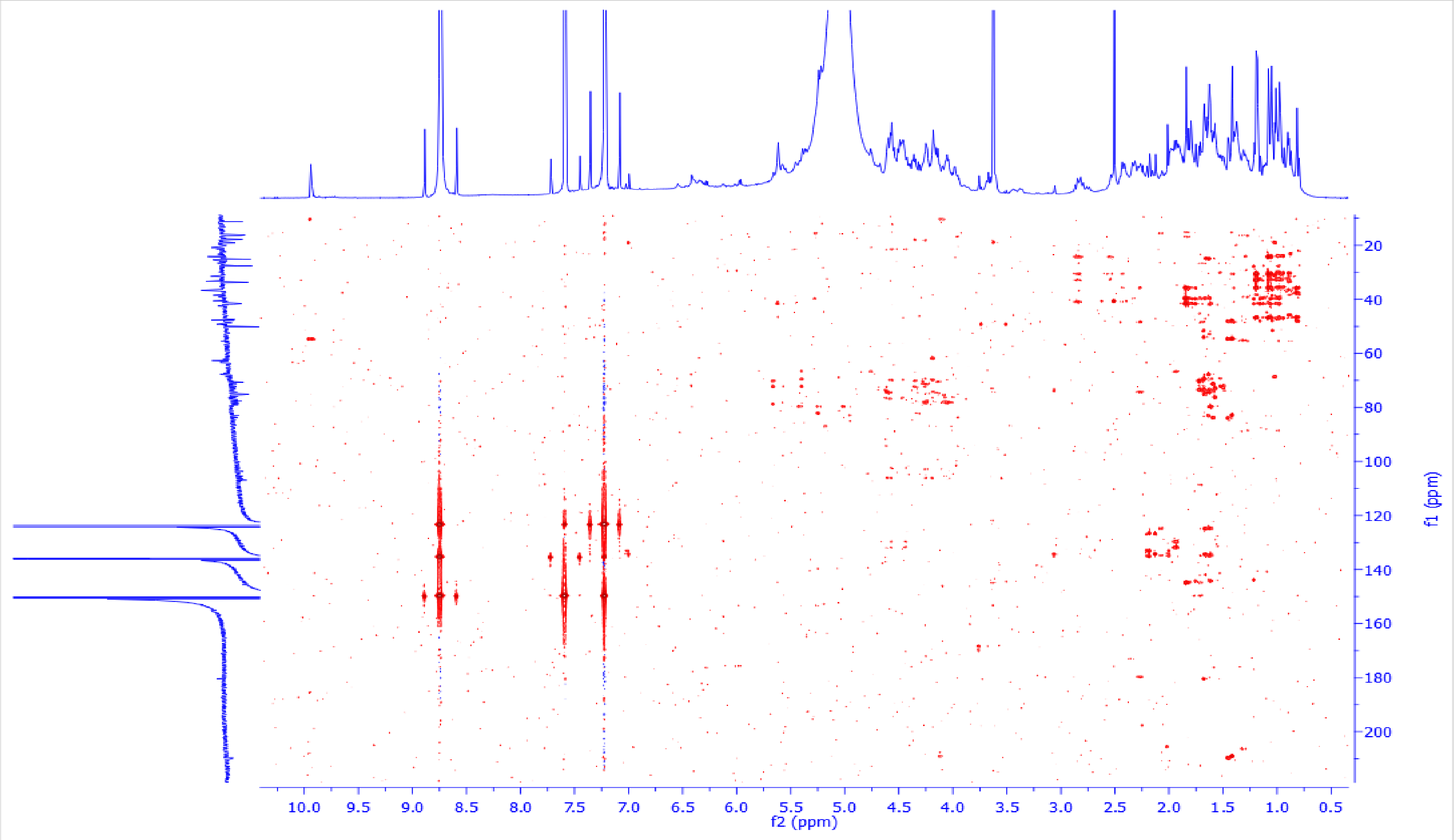

Supplementary Fig. 57. ^{1}H-^{13}C HMBC spectrum of QA-TriF(Q)RXX (12) produced in *N. benthamiana*, recorded in Pyridine-*d*5, 600/150 MHz.

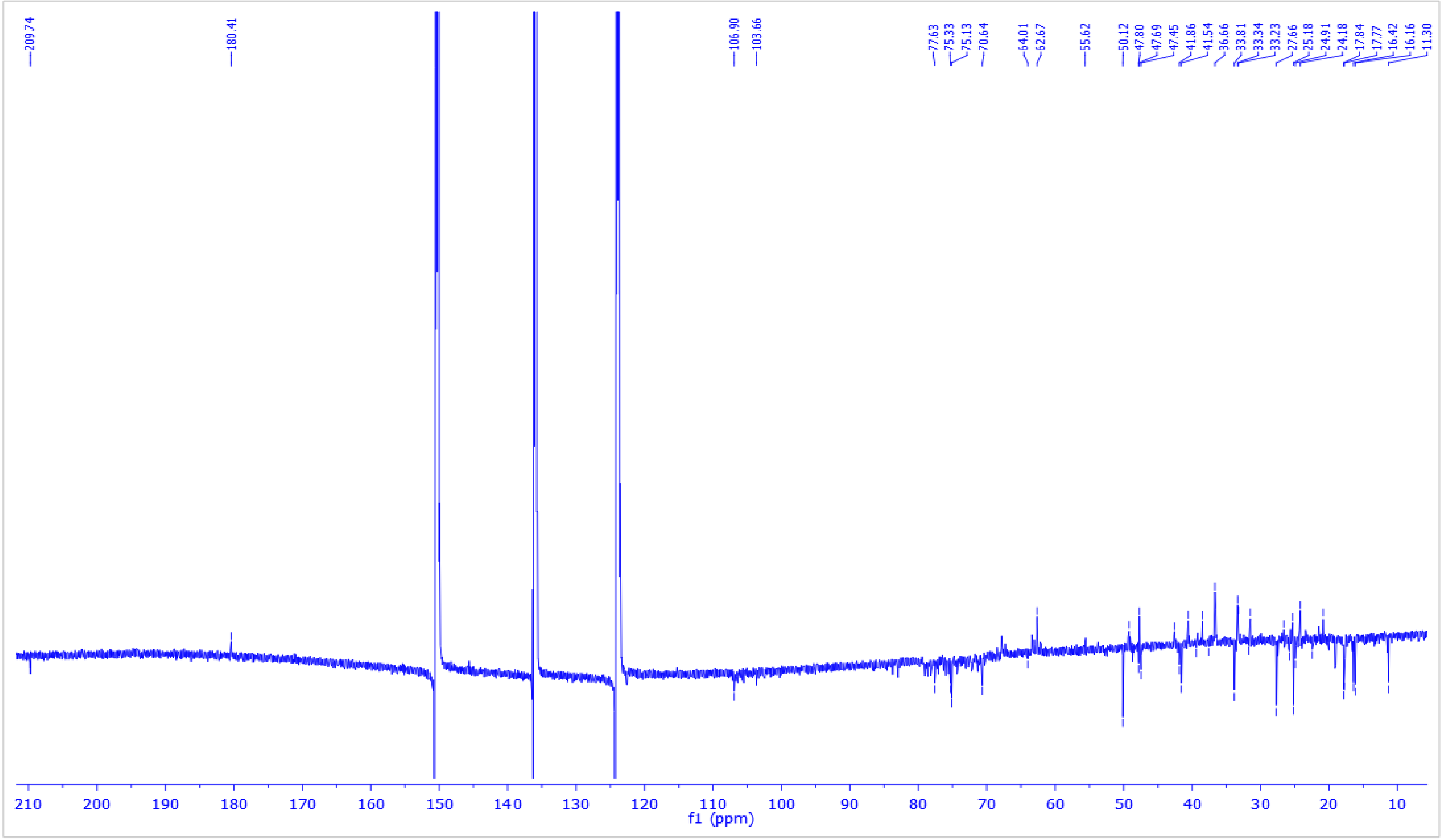

Supplementary Fig. 58. DEPTQ-135 spectrum of QA-TriF(Q)RXX (12) produced in *N. benthamiana*, recorded in Pyridine-d_5, 150 MHz.

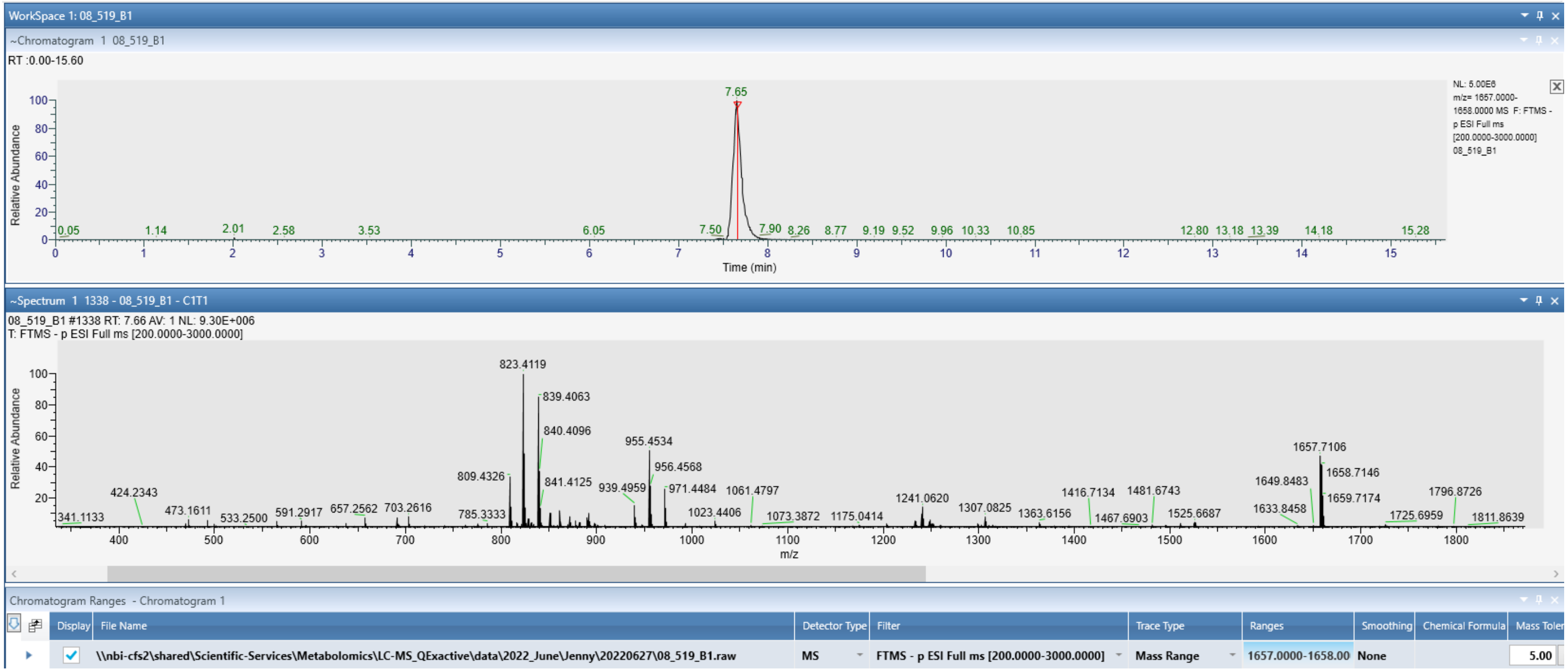

Supplementary Fig. 59. HR-LC-ESI-MS (EIC) of QA-TriF(Q)RXX (12) produced in *N. benthamiana.*

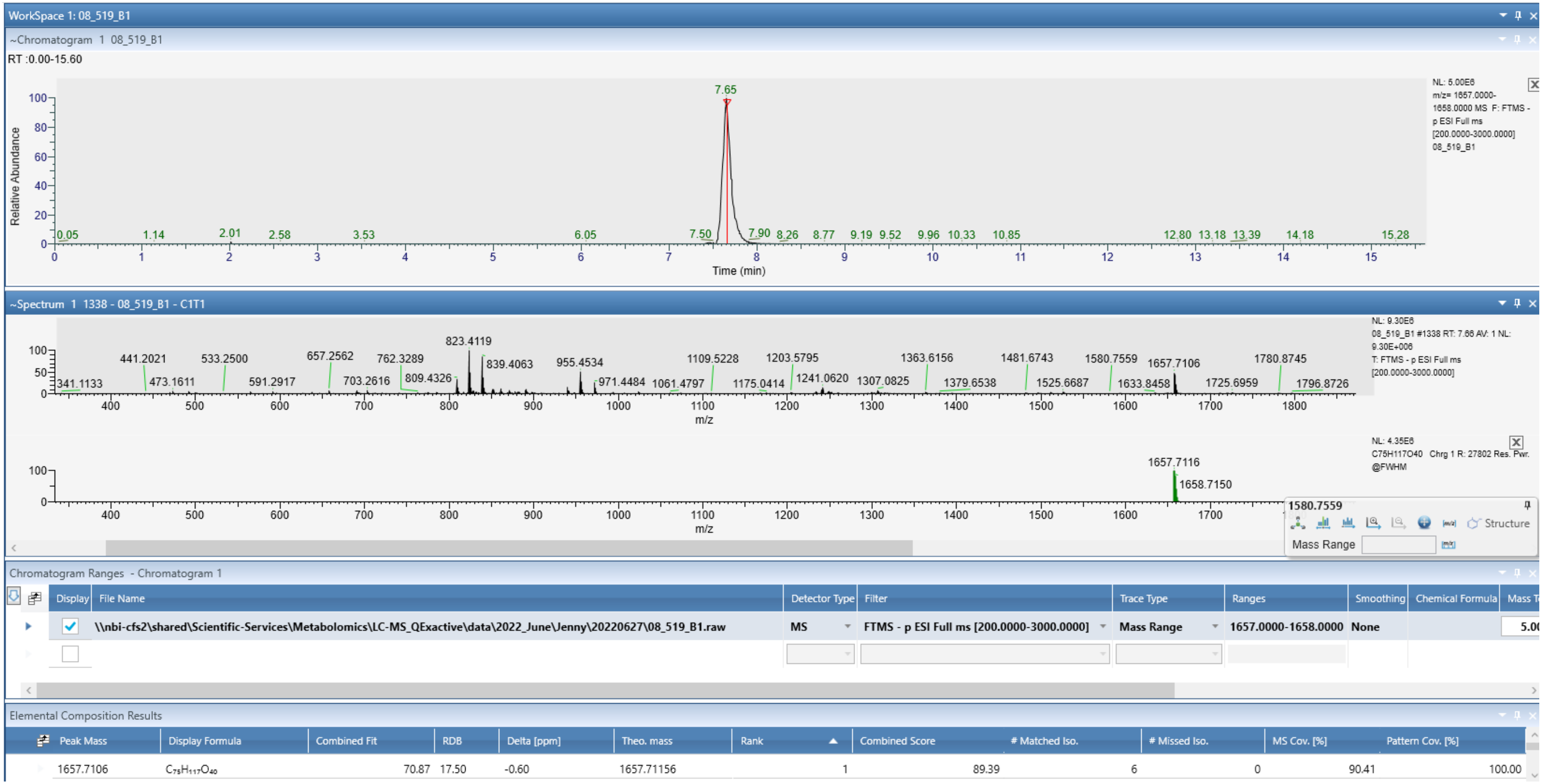

Supplementary Fig. 60. HR-LC-ESI-MS with predicted molecular formula of QA-TriF(Q)RXX (12) produced in *N. benthamiana.*

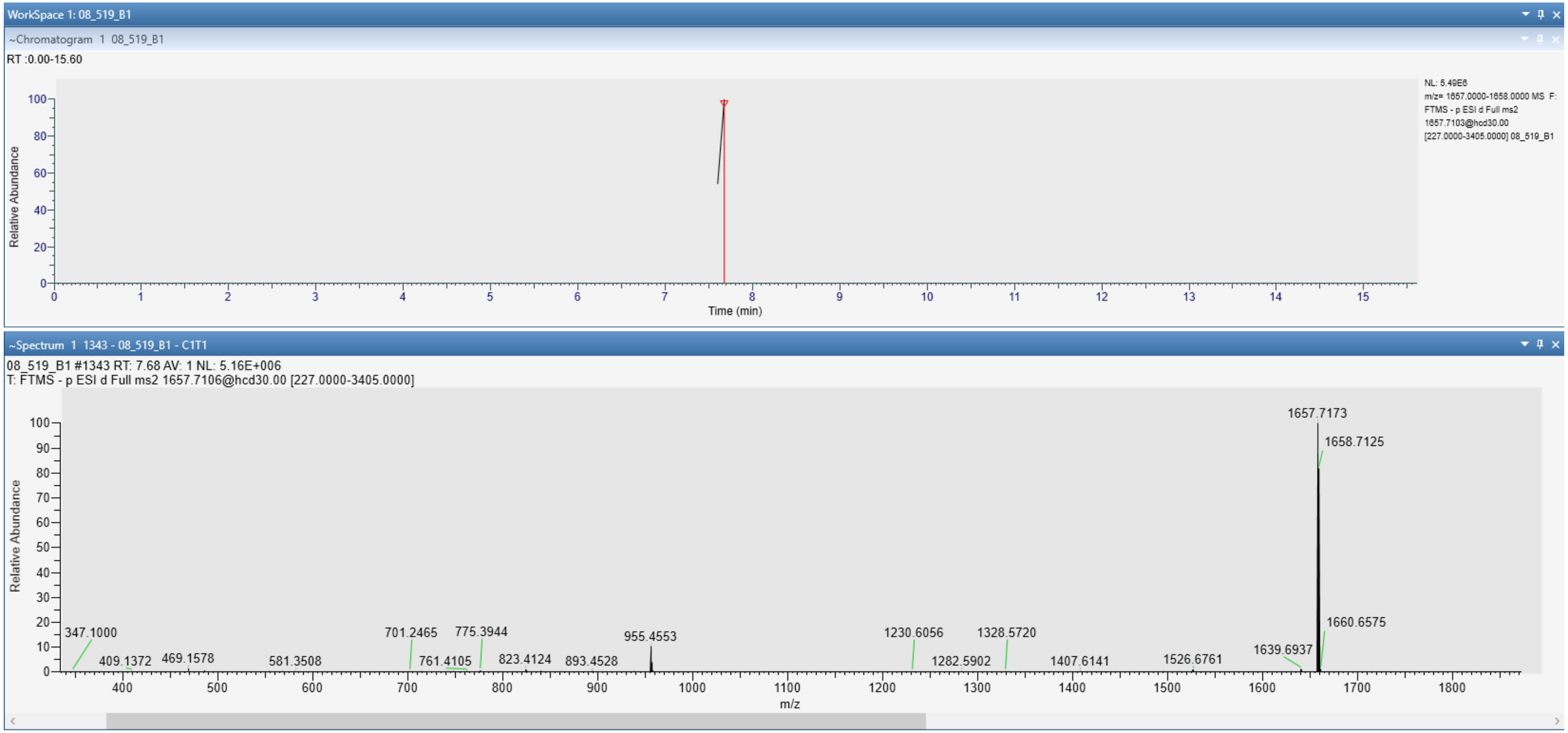

Supplementary Fig. 61. HR-LC-ESI-MS/MS2 for molecular ion 1657.7106 of QA-TriF(Q)RXX (12) produced in *N. benthamiana.*

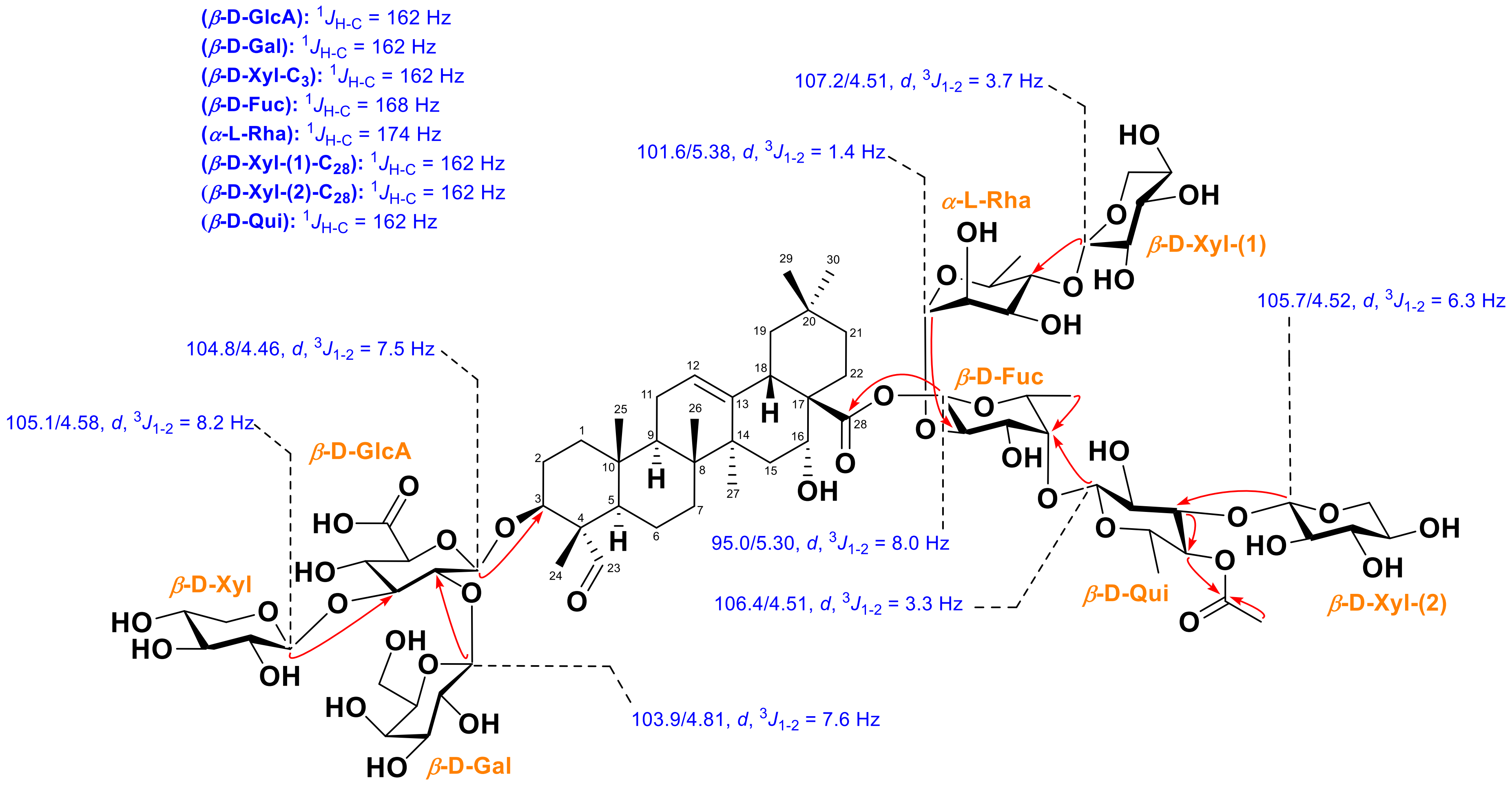

Supplementary Fig. 62. Key HMBC and coupled HSQC coupling constants ($^{1}J_{H\text{-}C}$) recorded for SO1699 (13′) purified from *S. officinalis* leaves. Red arrows represent H→C.

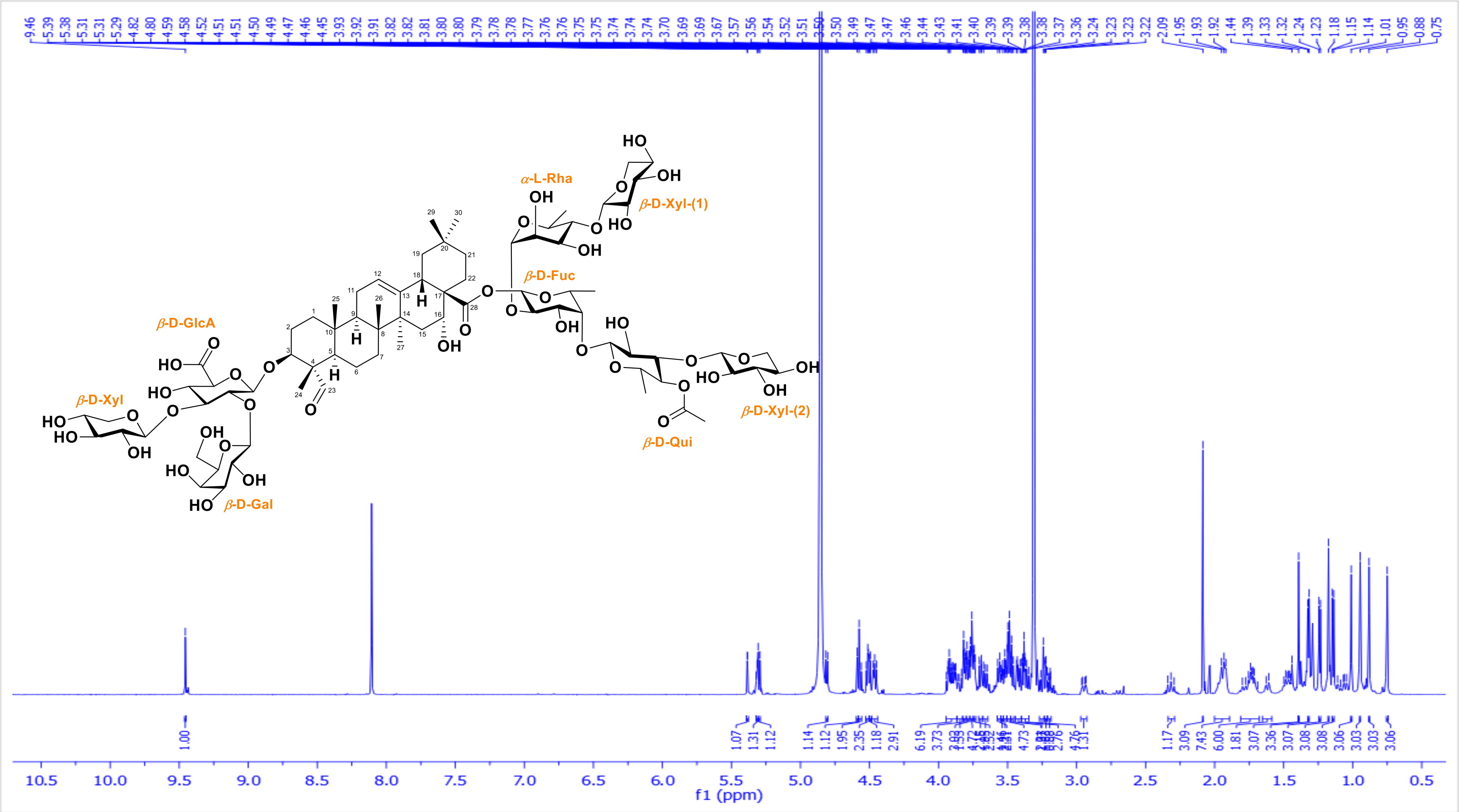

Supplementary Fig. 63. **^{1}H-NMR spectrum of SO1699 (13′) recorded in MeOH-*d*$_4$, 600 MHz.**

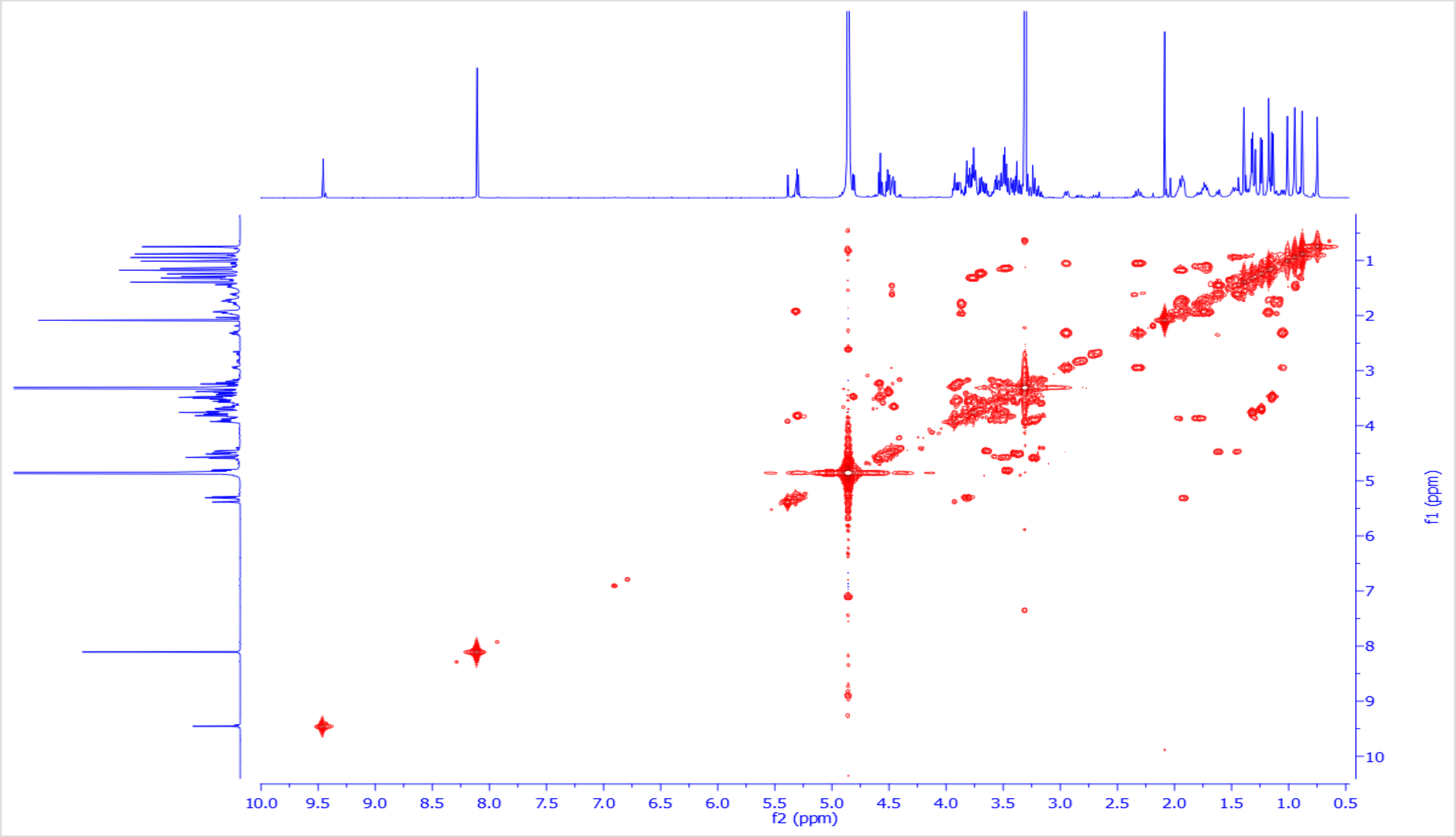

Supplementary Fig. 64. ^{1}H-^{1}H COSY spectrum of SO1699 (13′) recorded in MeOH-*d*$_4$, 600 MHz.

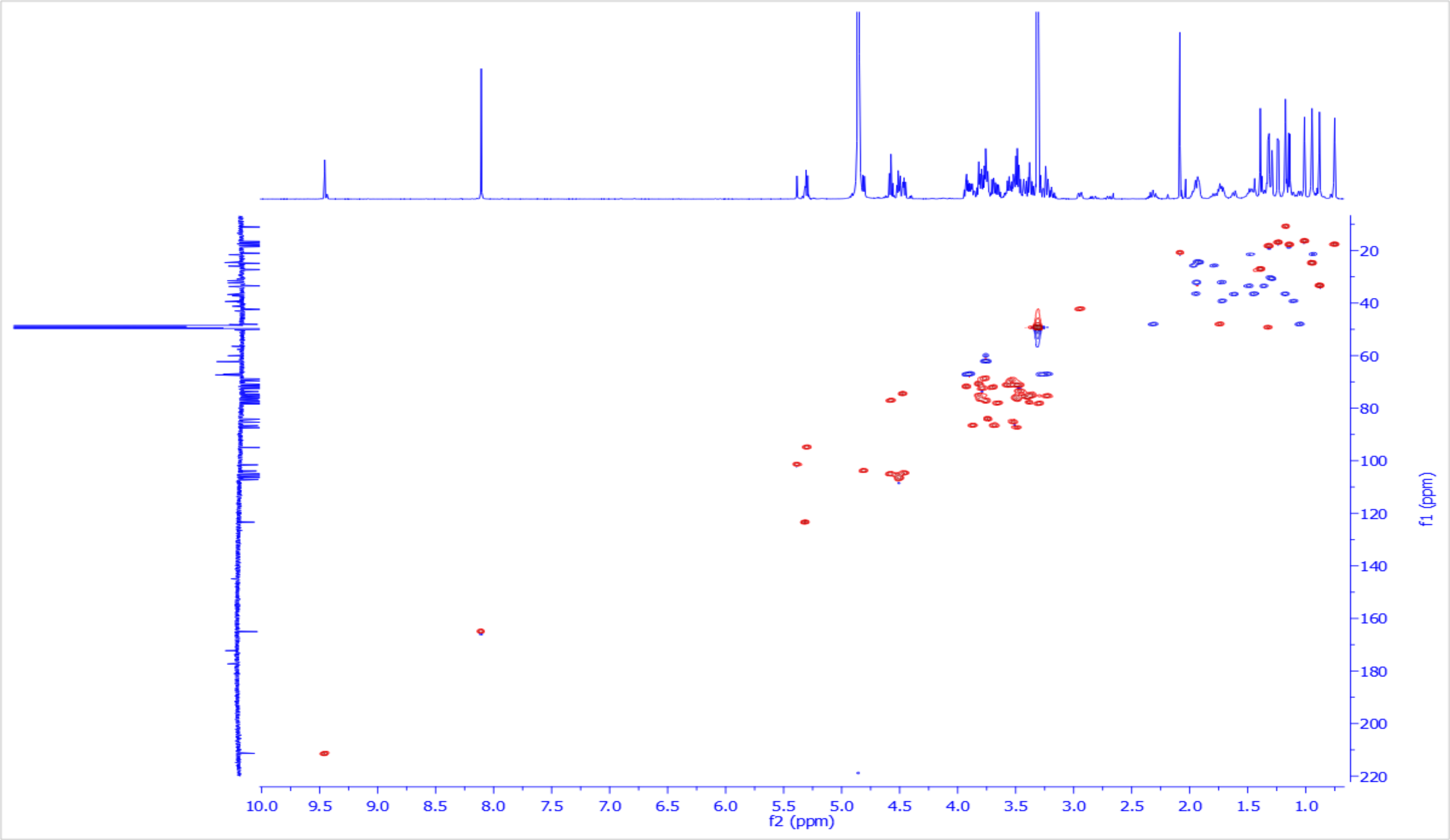

Supplementary Fig. 65. ^{1}H-^{13}C **HSQC spectrum of SO1699 (13′) recorded in MeOH-d_4, 600/150 MHz.**

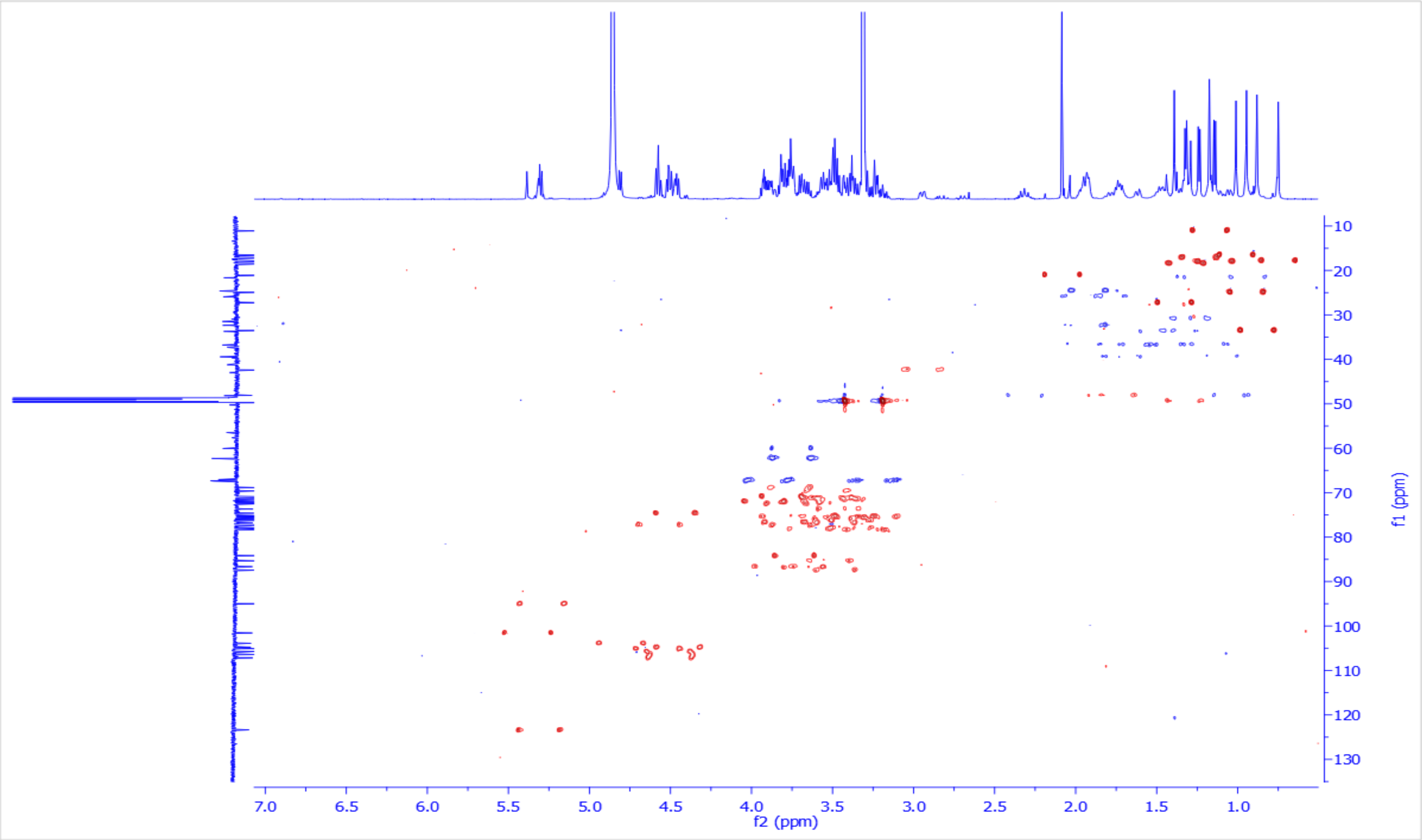

Supplementary Fig. 66. ^{1}H-^{13}C coupled HSQC spectrum of SO1699 (13′) recorded in MeOH-d_4, 600/150 MHz.

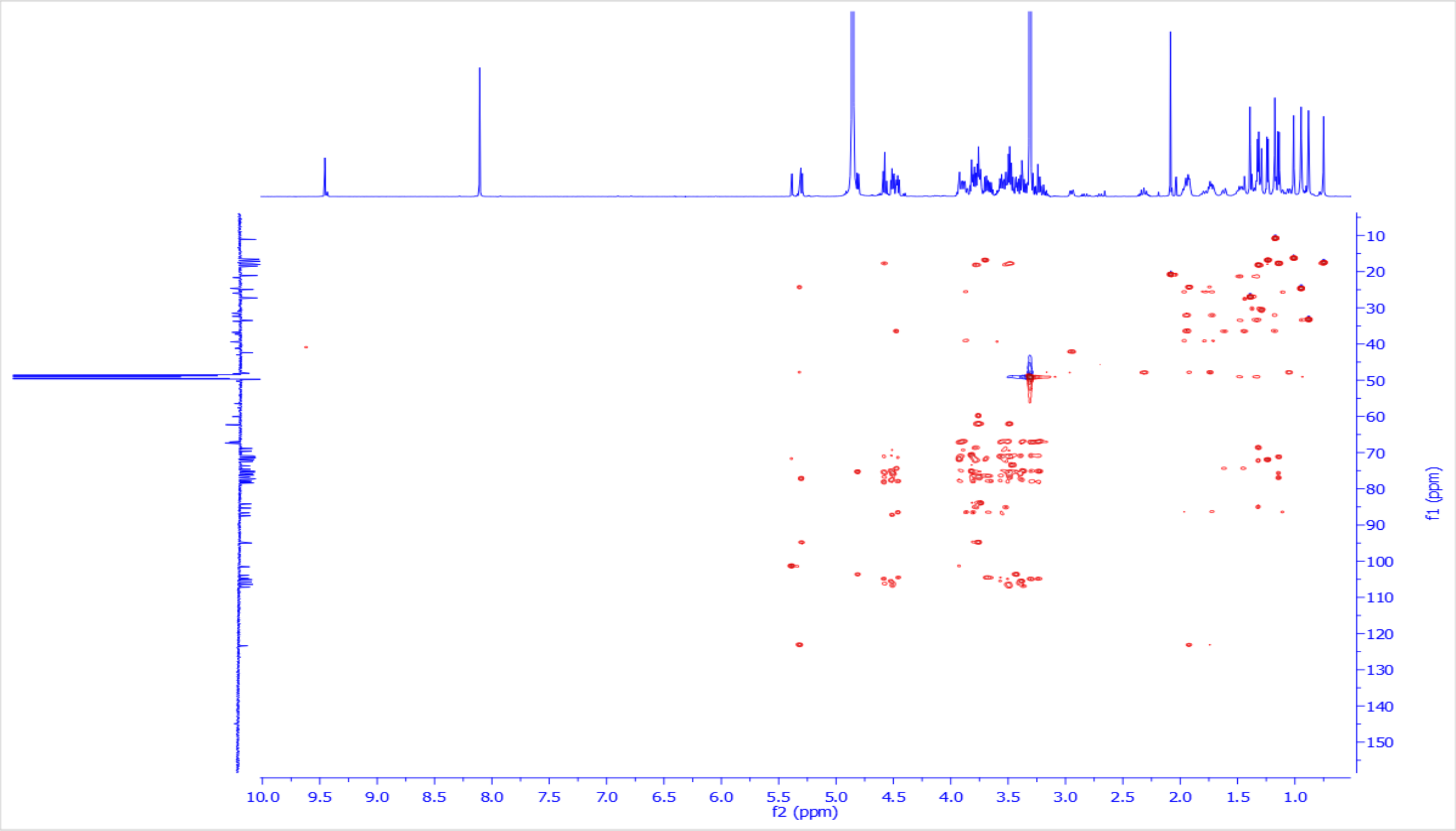

Supplementary Fig. 67. ^{1}**H-**^{13}**C HSQC-TOCSY spectrum of SO1699 (13′) recorded in MeOH-*d*$_4$, 600/150 MHz.**

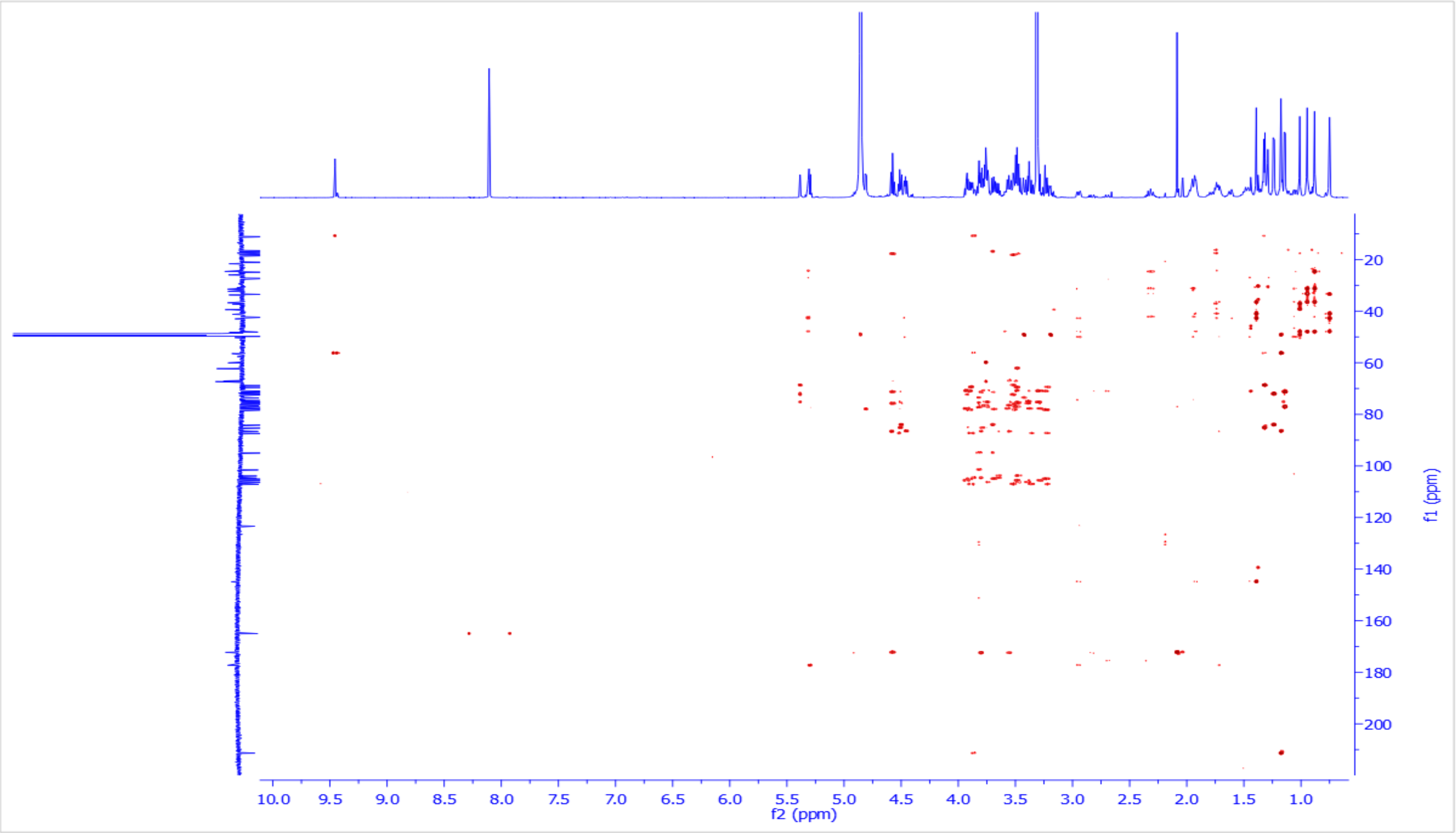

Supplementary Fig. 68. **^{1}H-^{13}C HMBC spectrum of SO1699 (13′) recorded in MeOH-*d*$_4$, 600/150 MHz.**

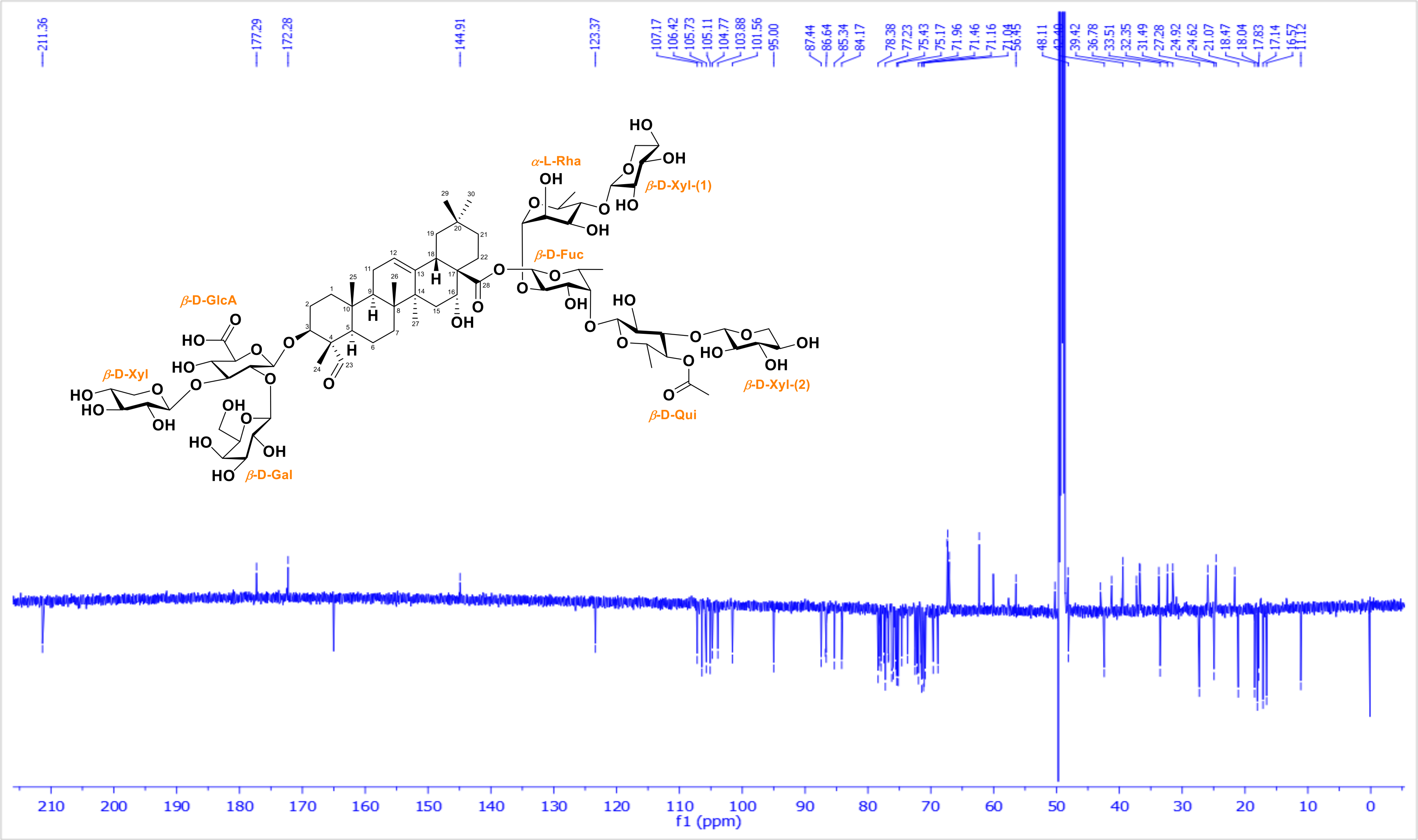

Supplementary Fig. 69. DEPTQ-135 spectrum of SO1699 (13′) recorded in MeOH-d4, 150 MHz.

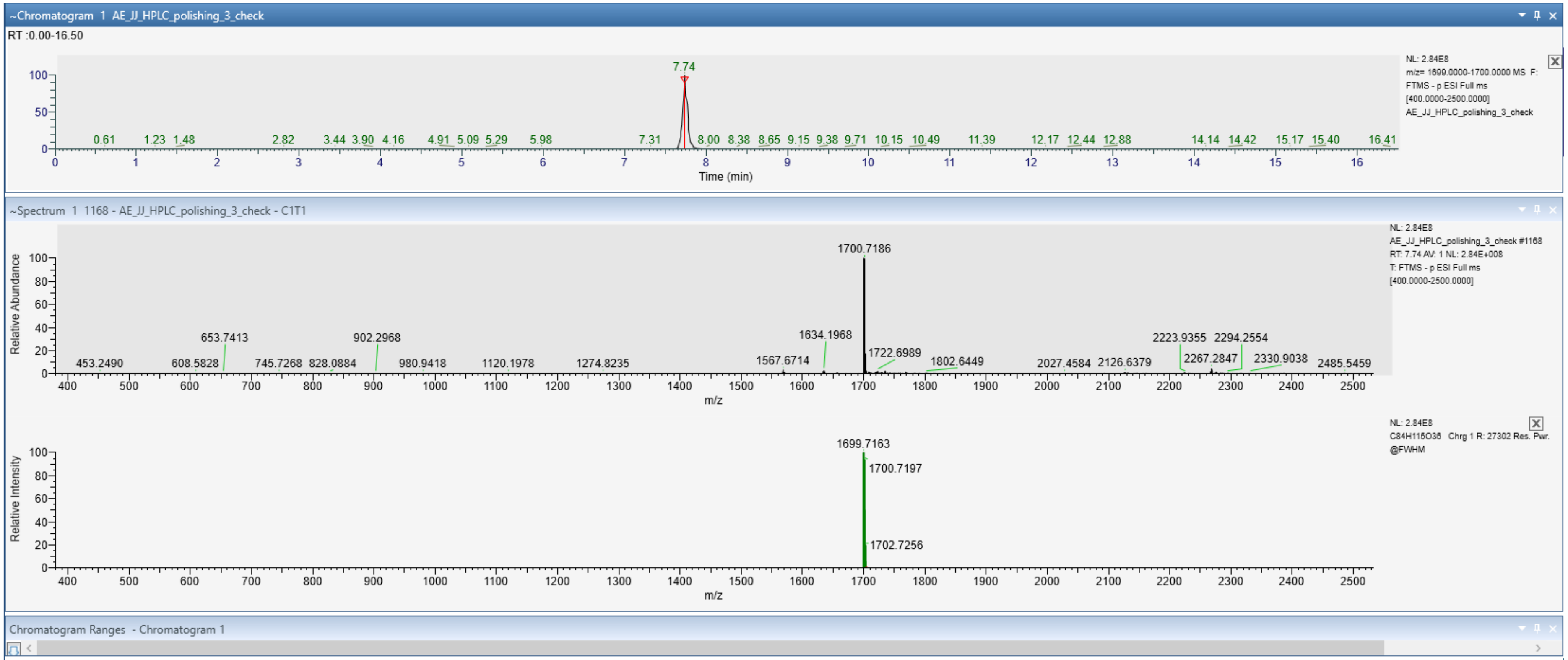

Elemental Composition Results

Peak Mass	Display Formula	Combined Fit	RDB	Delta [ppm]	Theo. mass	Rank	Combined Score	# Matched Iso.	# Missed Iso.	MS Cov. [%]	Pattern Cov. [%]
1699.7159	$C_{84}H_{115}O_{36}$	49.10	27.50	-0.18	1699.71626	1	76.79	6	1	78.33	99.86
1699.7159	$C_{77}H_{119}O_{41}$	24.39	18.50	-3.64	1699.72213	2	75.08	5	2	77.89	99.29

Supplementary Fig. 70. HR-LC-MS (EIC) of SO1699 (13′) with calculated chemical formula $C_{77}H_{119}O_{47}$ [M-H].

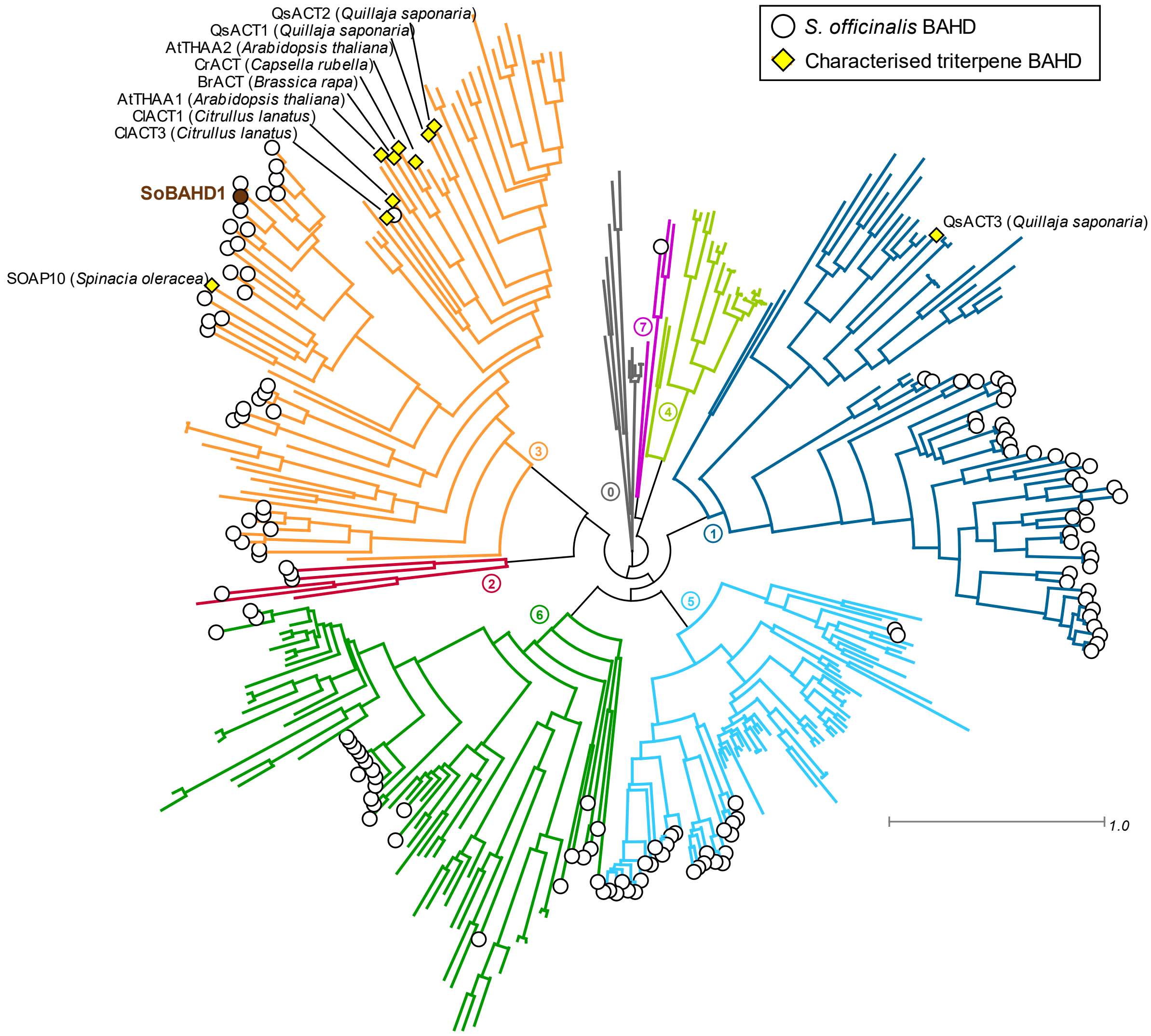

Supplementary Fig. 71. Phylogenetic analysis of BAHD acyltranferase candidates found in *S. officinalis*. Genome mining, alignment of protein sequences and generation of maximum likelihood phylogeny was carried out as descried in methods. Sequences from *S. officinalis* are indicated with white circles. Sequences from [12] were used as reference for clade assignment. The following additional characterised BAHDs were included and labelled with yellow diamonds: AtTHAA1 (At5g47980, *Arabidopsis thaliana*), AtTHAA2 (AT5G47950, *A. thaliana*), BrACT (XP_009117330.1, *Brassica rapa*), CrACT (Carubv10017289m, *Capsella rubella*), ClACT1 (Cla007081, *Citrullus lanatus*), ClACT3 (Cla022713, *C. lanatus*), SOAP10 (KNA12459.1, *Spinacia oleracea*) and three BAHDs from *Quillaja saponaria*: QsACT1 (OQ107258), QsACT2 (OQ241420) and QsACT3 (OQ241426). Labelled clade numbers corresponding to those described in [12]. The characterised sequence SoBAHD1 is labelled in bold. The scale bar indicates the number of amino acid substitutions per site.

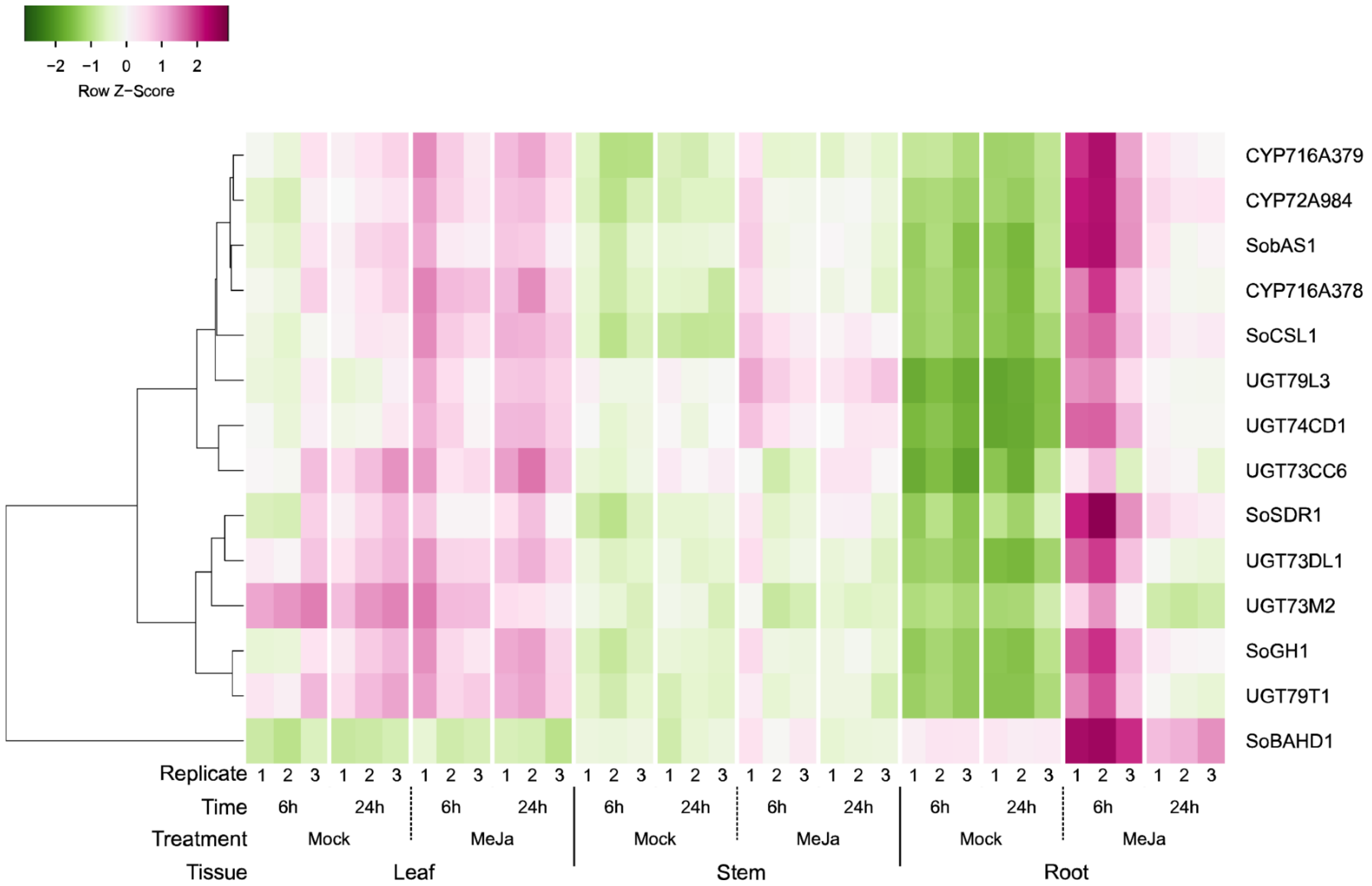

Supplementary Fig. 72. Expression profiles of saponarioside biosynthetic genes induced by methyl jasmonate treatment. Hydroponically grown *S. officinalis* plants were treated with 50 µM methyl jasmonate (MeJa) or ethanol (Mock) and were sampled in triplicates at 6 and 24 h after treatment.

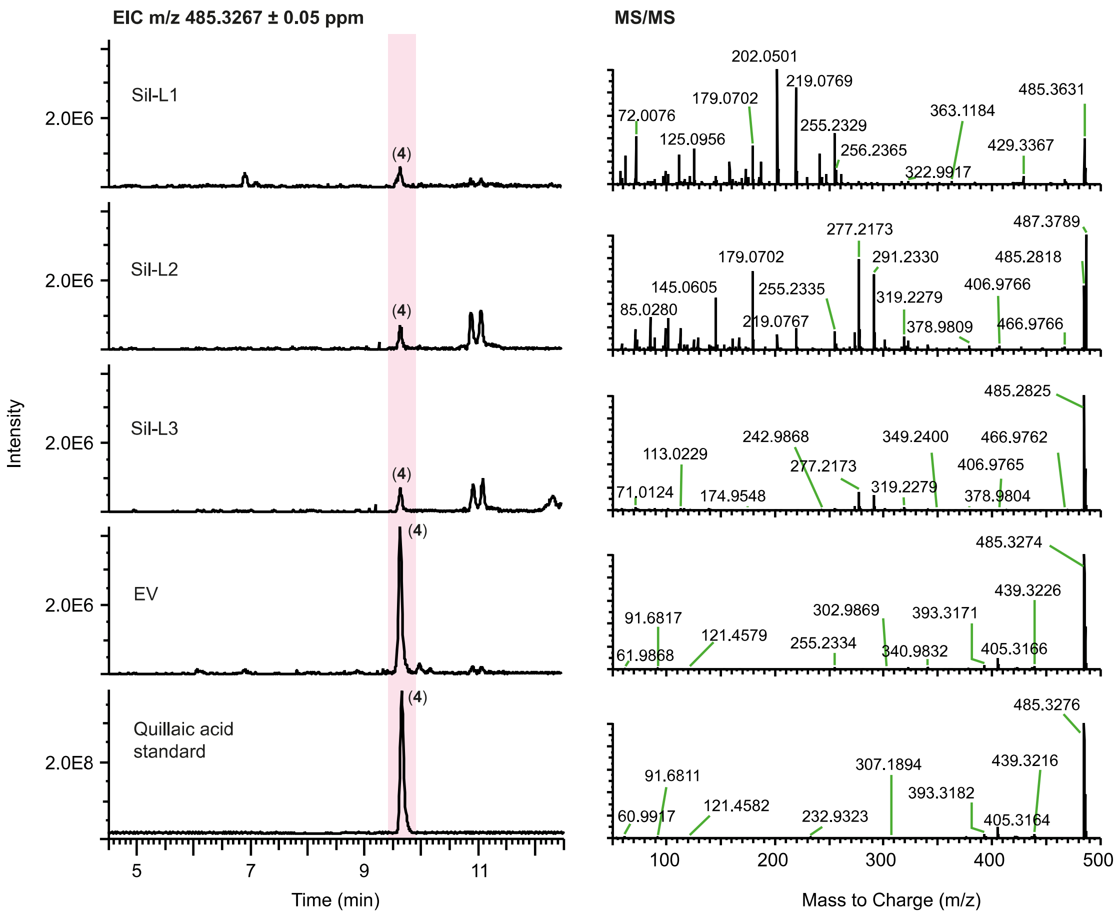

Supplementary Fig. 73. Detection of quillaic acid in hairy root cultures of *S. officinalis*. EIC at *m/z* 485.3267 ([M-H]$^-$ of quillaic acid) from *SobAS1* silenced hairy root lines (Sil-L1, L2, L3) and empty vector control (EV) are shown. Corresponding MS/MS spectra are also displayed. Quillaic acid (**4**) is identified by comparison to commercial standard.

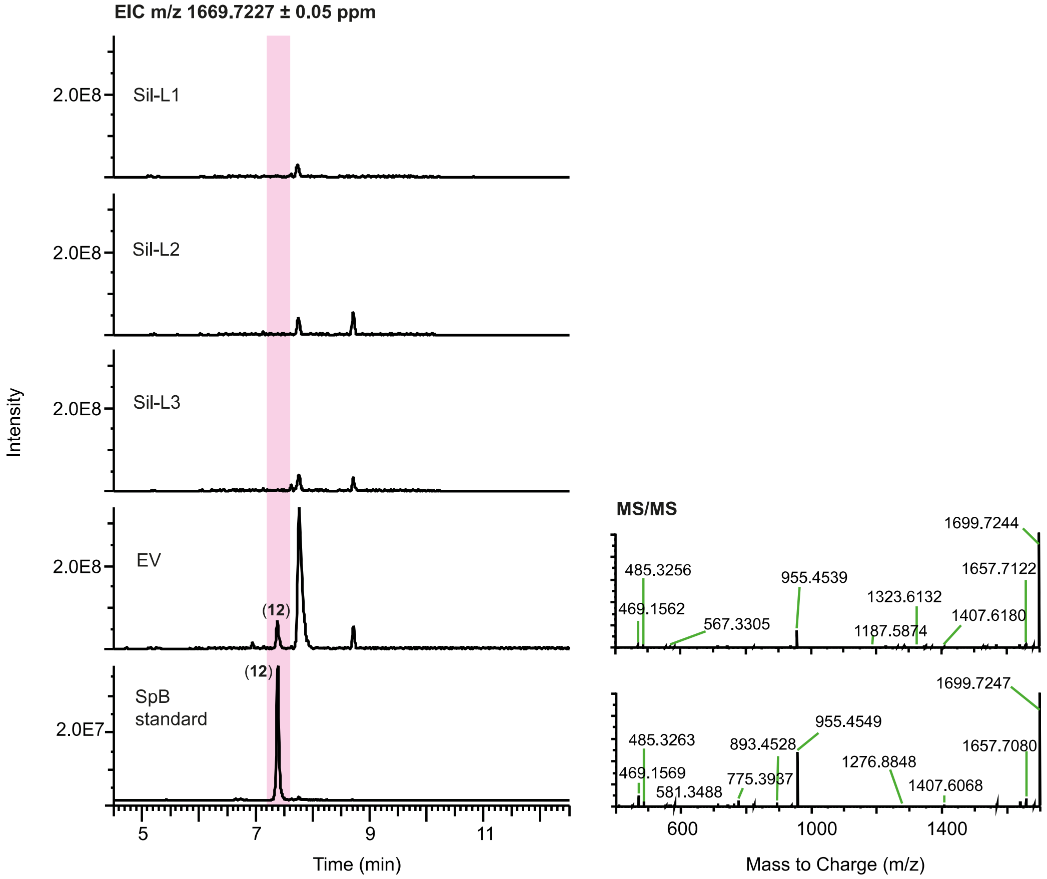

Supplementary Fig. 74. Detection of saponarioside B in hairy root cultures of *S. officinalis.* EIC at *m/z* 1669.7227 ([M-H]$^-$ of SpB) from *SobAS1* silenced hairy root lines (Sil-L1, L2, L3) and empty vector control (EV) are shown. Corresponding MS/MS spectra are also displayed. Saponarioside B (**13**) is identified by comparison to authentic standard.

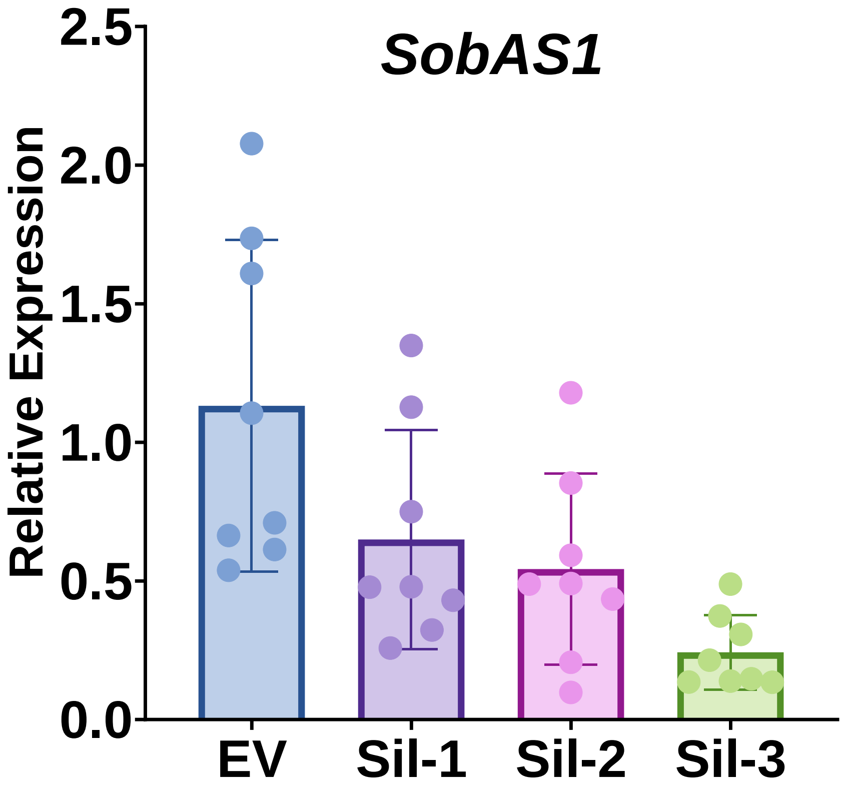

Supplementary Fig. 75. Relative expression levels of *SobAS1* in *SobAS1*-RNAi hairy root lines (Sil-1, Sil-2, Sil-3). EV, empty vector control. Each bar represents the mean of eight biological replicates, and error bars indicate standard deviation. Source data is available in Supplementary Data 1.

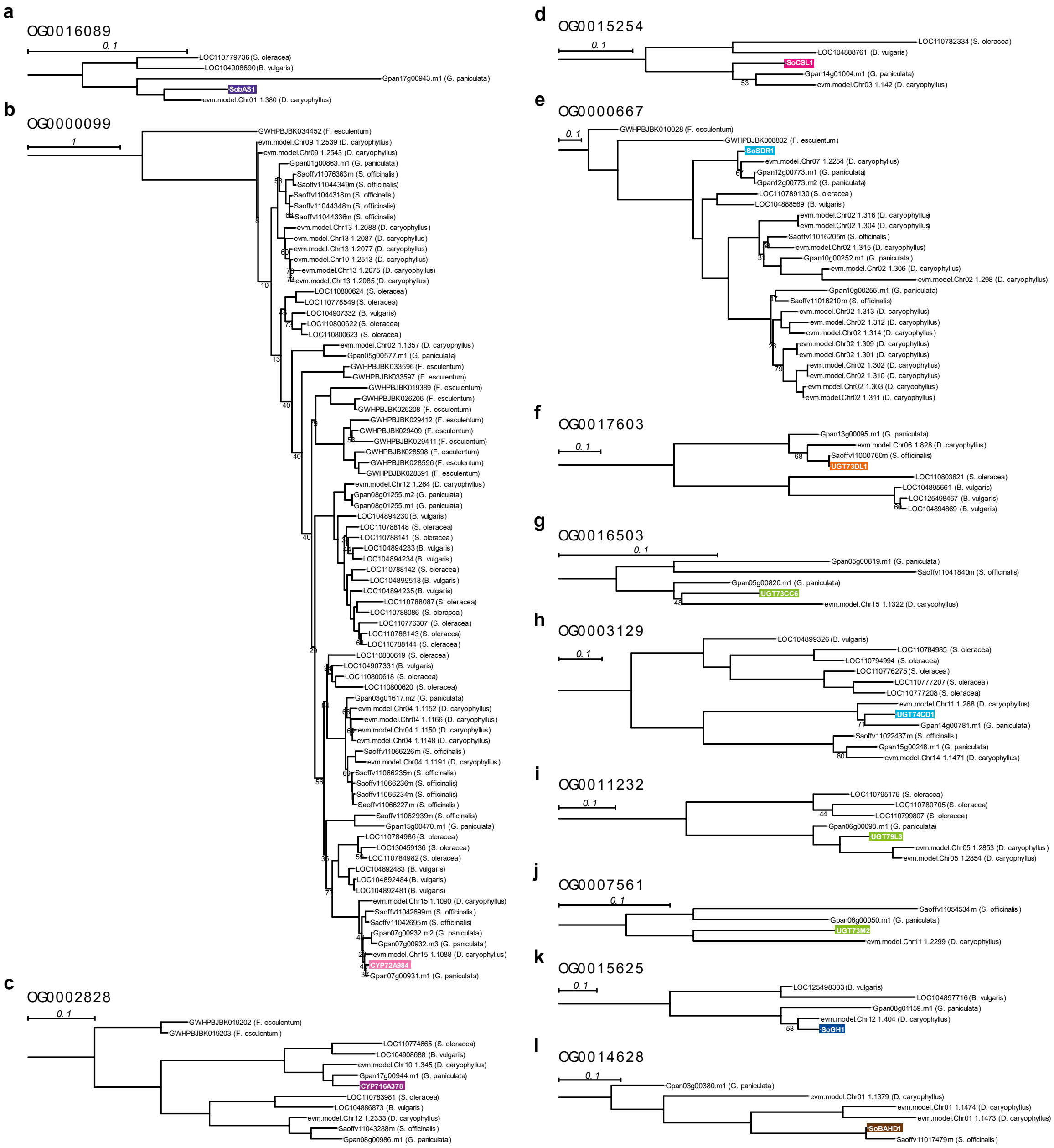

Supplementary Fig. 76. Orthologues of saponarioside biosynthetic genes found across Caryophyllales species. Orthologues found in each orthogroup (as defined by OrthoFinder[13] for the five Caryophyllales species studied (as in Extended Data Fig. 3). **a-l.** Maximum likelihood phylogenies for the identified orthogroups which contained greater than three members. Alignment of protein sequences and generation of maximum likelihood phylogeny was carried out as descried in methods. Bootstrap values less than 80% are shown beside each node. The scale bar indicates the number of amino acid substitutions per site.

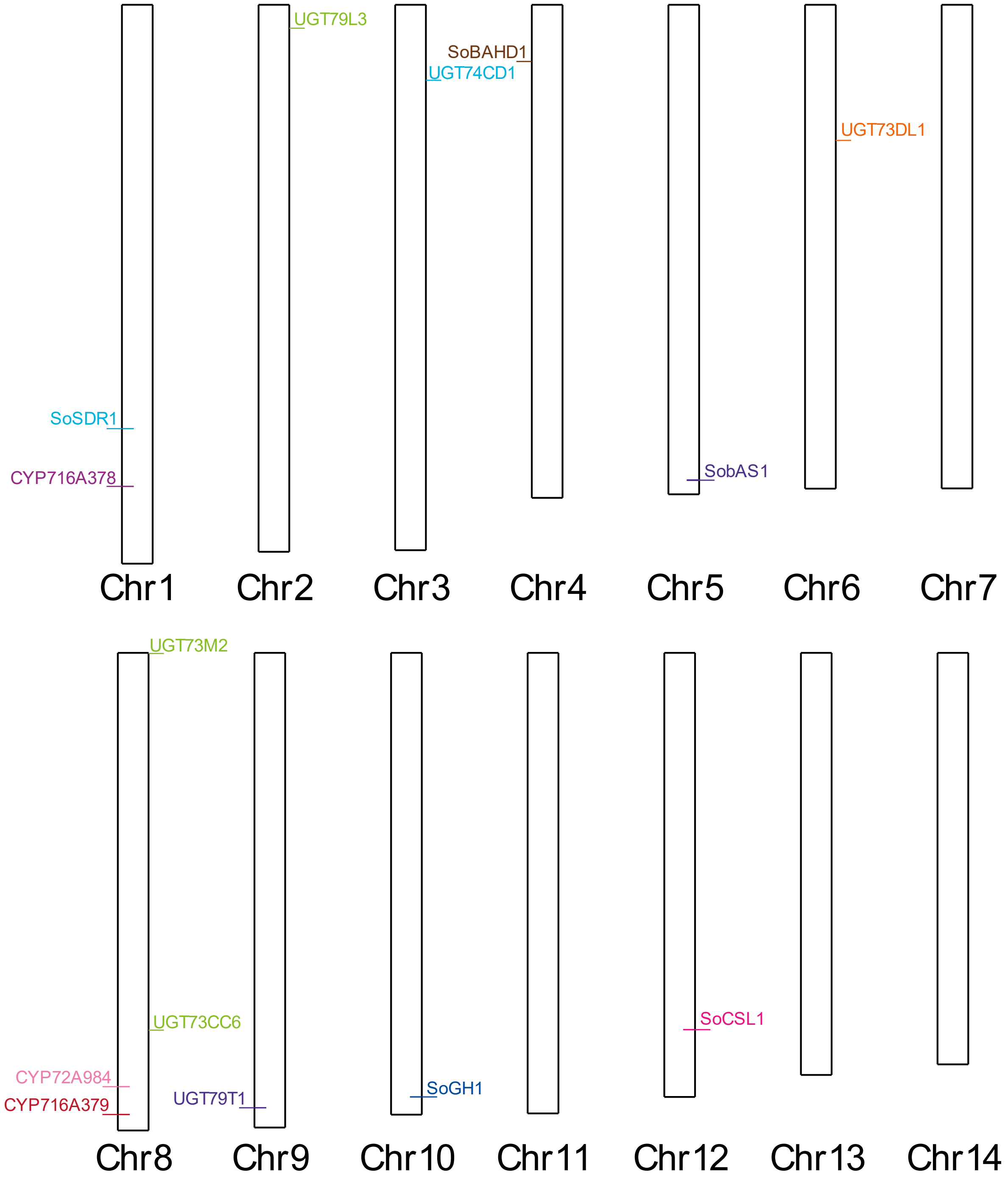

Supplementary Fig. 77. ***S. officinalis*** **chromosome map showing the physical location of identified saponarioside biosynthetic genes.**

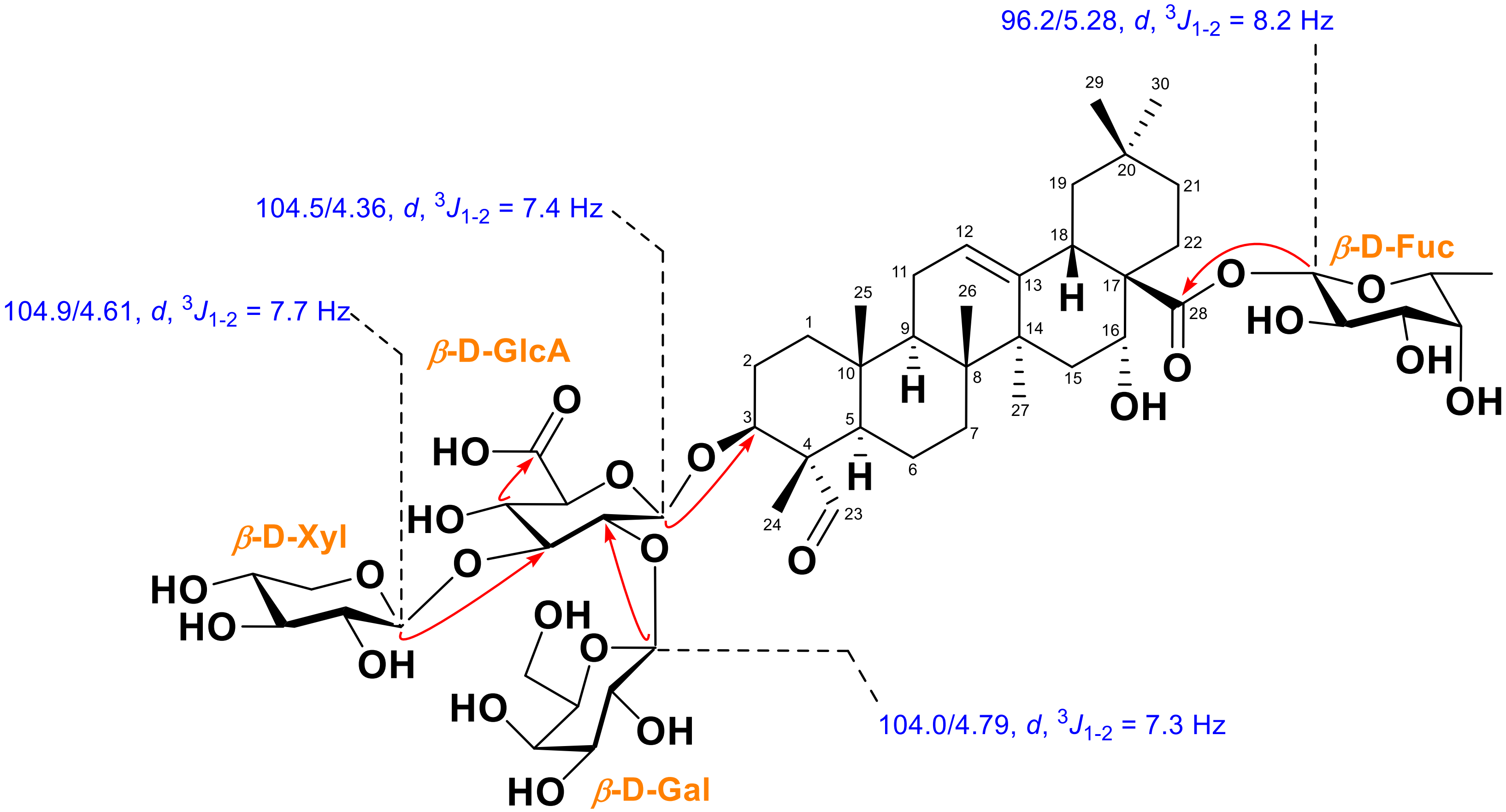

Supplementary Fig. 78. Key HMBC recorded for QA-Trix-F (8) purified from *Quillaja saponaria* bark extract. Red arrows represent H→C.

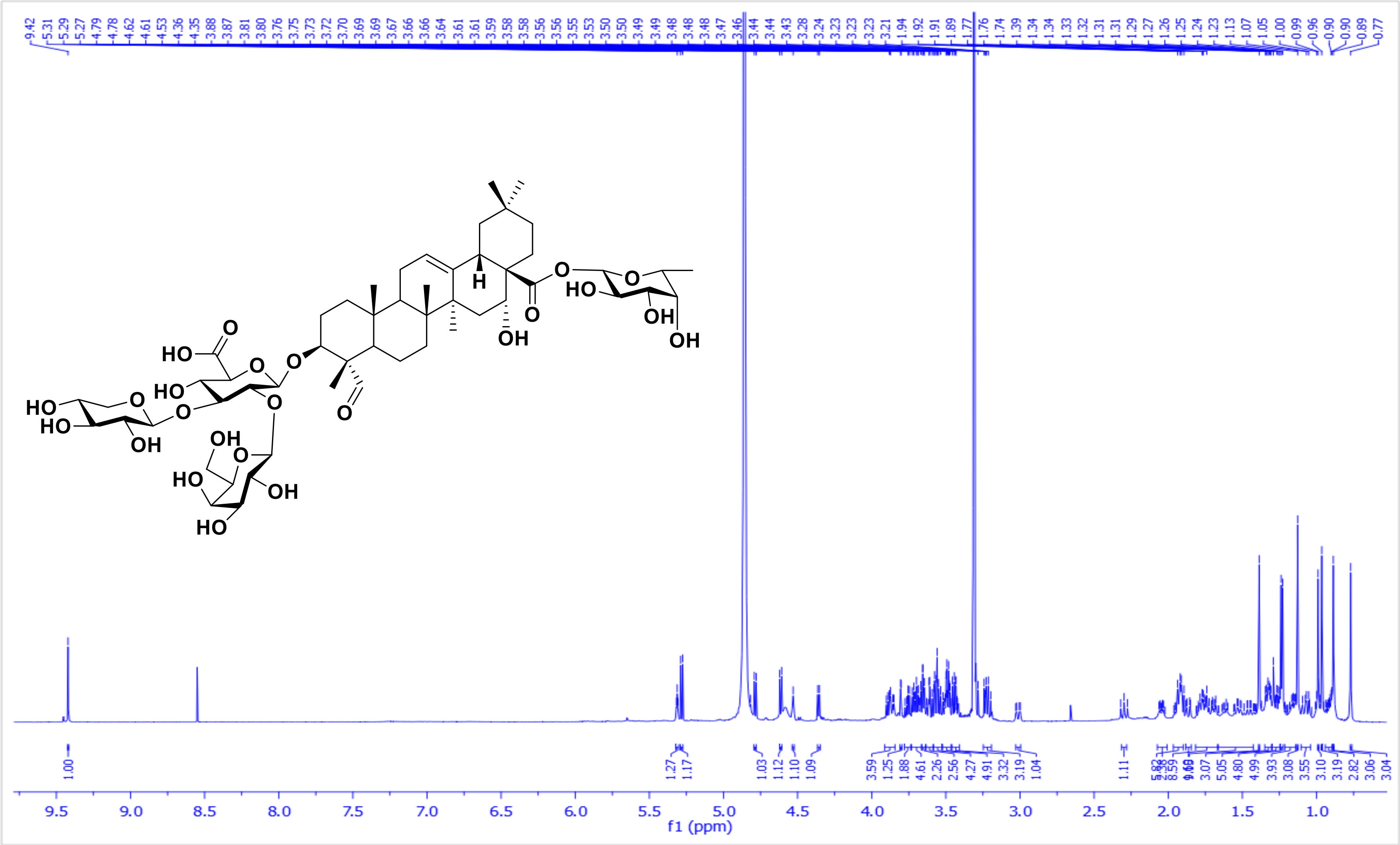

Supplementary Fig. 79. ¹H-NMR spectrum of QA-TriF (8) recorded in MeOH-*d4* (600 MHz).

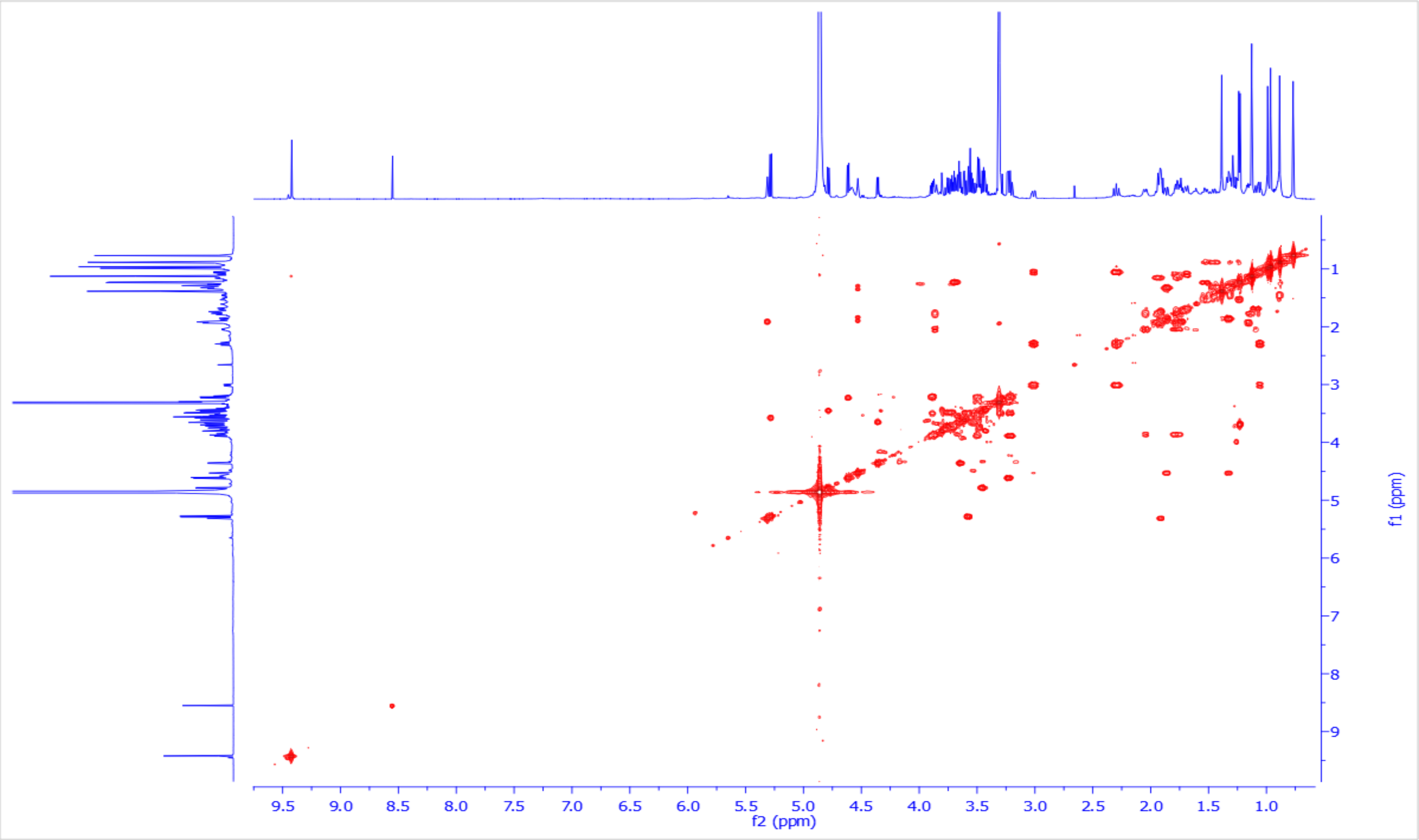

Supplementary Fig. 80. ^{1}H-^{1}H **COSY spectrum of QA-TriF (8) recorded in MeOH-*d4* (600 MHz).**

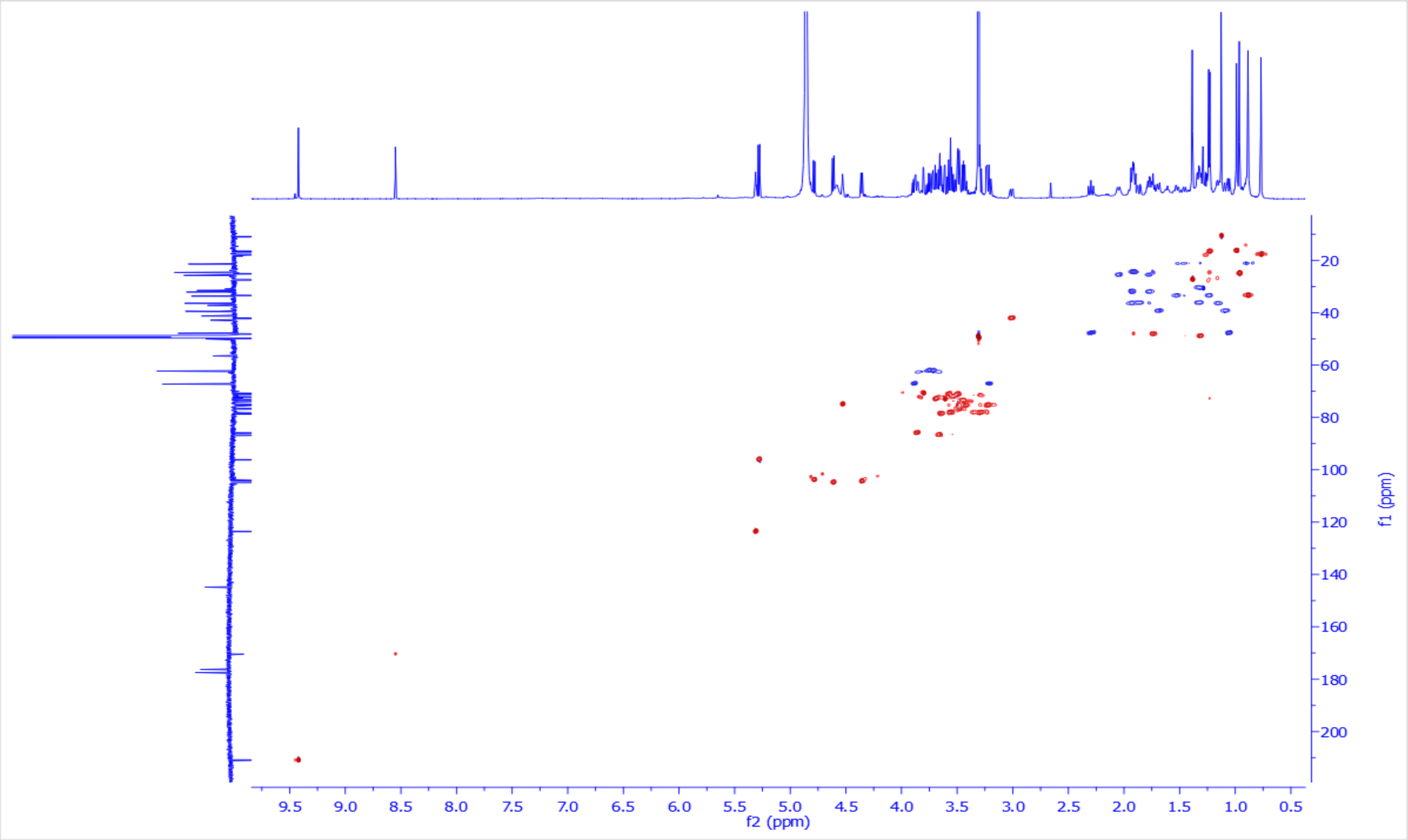

Supplementary Fig. 81. **^{1}H-^{13}C HSQC spectrum of QA-TriF (8) recorded in MeOH-*d*4 (600/150 MHz).**

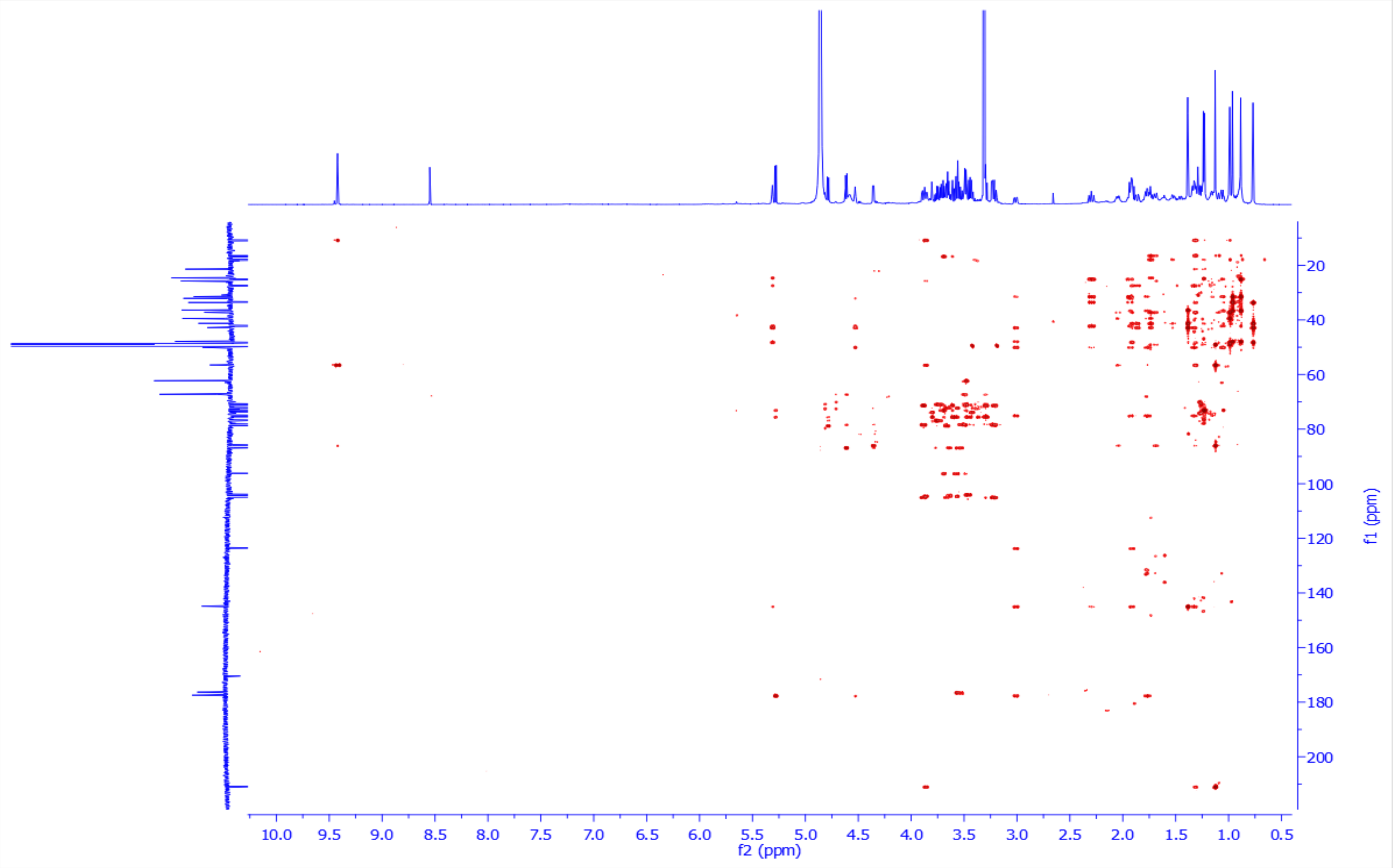

Supplementary Fig. 82. ^{1}H-^{13}C HMBC spectrum of QA-TriF (8) recorded in MeOH-*d4* (600/150 MHz).

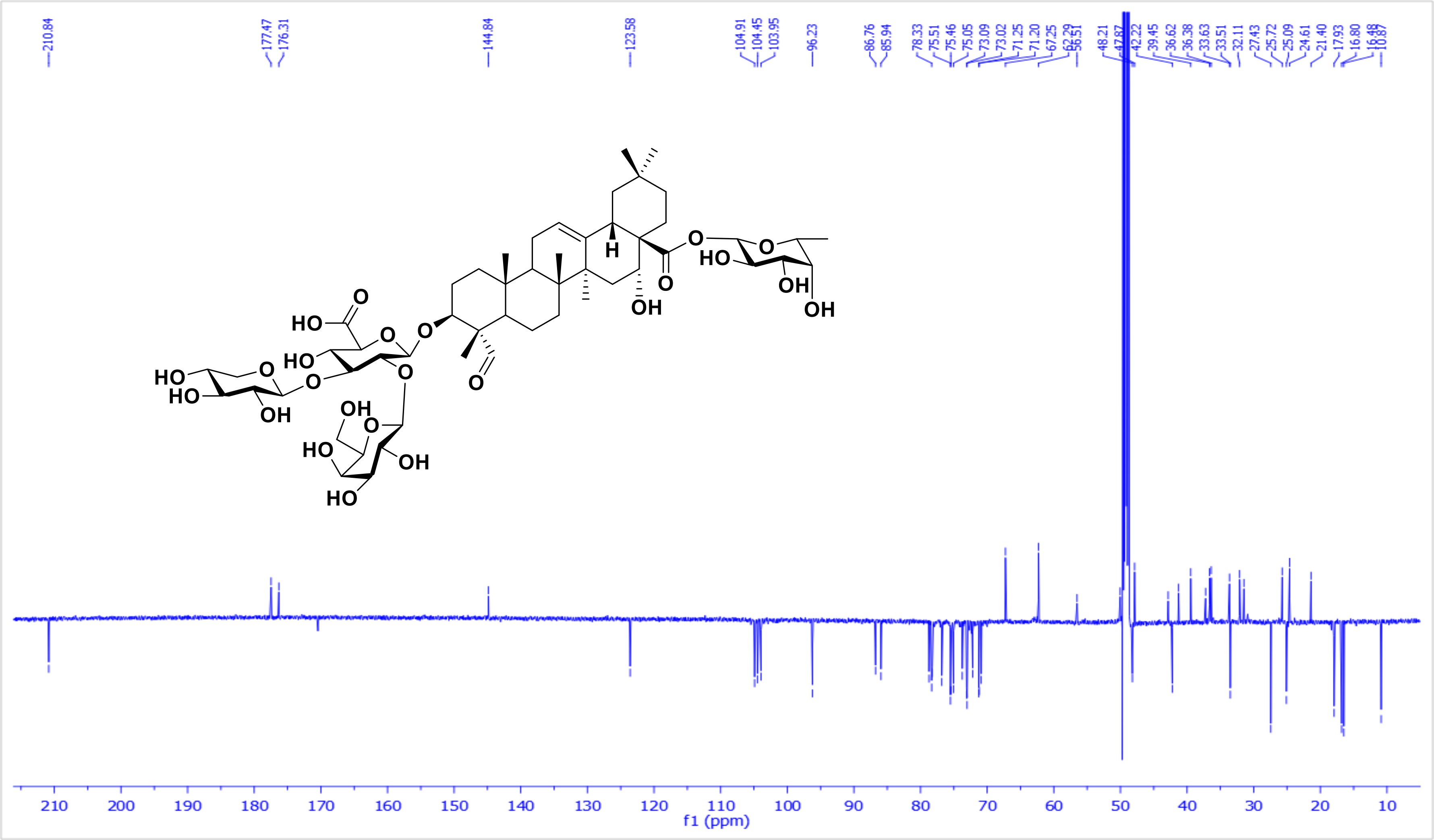

Supplementary Fig. 83. DEPTQ-135 spectrum of QA-TriF (8) recorded in MeOH-*d*4 (150 MHz).

Supplementary Tables

Supplementary Table 1. ^{1}H, ^{13}C NMR spectroscopic data recorded for saponarioside A, MeOH-d_4 (600/150 MHz).

No.	δ_C, Type	δ_H mult, (J in Hz)	No.	δ_C, Type	δ_H mult, (J in Hz)
**1**	39.5, CH_2	1.71/1.09, m	**C_3-Xyl-1**	105.1, CH	4.59, d (7.8)
**2**	25.9, CH_2	1.98/1.79, m	**C_3-Xyl-2**	75.5, CH	3.22, m
**3**	86.4, CH	3.86, m	**C_3-Xyl-3**	78.4, CH	3.29, overlapped
**4**	56.5, Cq	-	**C_3-Xyl-4**	71.2, CH	3.51, m
**5**	49.3, CH, overlapped	1.31, m	**C_3-Xyl-5**	67.3, CH_2	3.91/3.24, m
**6**	21.6, CH_2	1.47/0.93, m	**Fuc-1**	95.0, CH	5.30, d (8)
**7**	33.7, CH_2	1.49/1.35, m	**Fuc-2**	75.2, CH	3.79, m
**8**	41.2, Cq	-	**Fuc-3**	77.4, CH	3.75, m
**9**	48.1, CH	1.73, m	**Fuc-4**	84.4, CH	3.76, m
**10**	37.3, Cq	-	**Fuc-5**	72.2, CH	3.70, m
**11**	24.6, CH_2	1.92/1.92, m	**Fuc-6**	17.1, CH_3	1.24, d (6.3)
**12**	123.4, CH	5.30, m	**Rha-1**	101.5, CH	5.39, d (1.5)
**13**	144.9, Cq	-	**Rha-2**	71.9, CH	3.92, m
**14**	43.0, Cq	-	**Rha-3**	72.5, CH	3.78, m
**15**	36.7, CH_2	1.94/1.44, m	**Rha-4**	85.3, CH	3.51, m
**16**	74.6, CH	4.47, m	**Rha-5**	68.8, CH	3.76, m
**17**	50.2, Cq	-	**Rha-6**	18.5, CH_3	1.31, d (6.2)
**18**	42.4, CH	2.94, dd (14.0, 3.8)	**C_{28}-Xyl (1)-1**	107.2, CH	4.51, d (7.2)
**19**	48.2, CH_2	2.31, t (13.4)/1.05, m	**C_{28}-Xyl (1)-2**	75.3, CH	3.37, m
**20**	31.5, Cq	-	**C_{28}-Xyl (1)-3**	87.5, CH	3.48, m
**21**	36.8, CH_2	1.94/1.17, m	**C_{28}-Xyl (1)-4**	69.6, CH	3.54, m
**22**	32.3, CH_2	1.92/1.71, m	**C_{28}-Xyl (1)-5**	67.1, CH_2	3.88/3.22, m
**23**	211.4, CH	9.45, s	**C_{28}-Xyl (2)-1**	105.7, CH	4.51, d (7.2)
**24**	11.1, CH_3	1.17, s	**C_{28}-Xyl (2)-2**	75.3, CH	3.37, m
**25**	16.6, CH_3	1.01, s	**C_{28}-Xyl (2)-3**	77.9, CH	3.38, m
**26**	17.8, CH_3	0.75, s	**C_{28}-Xyl (2)-4**	71.2, CH	3.57, m
**27**	27.3, CH_3	1.39, s	**C_{28}-Xyl (2)-5**	67.4, CH_2	3.94/3.29, m
**28**	177.3, Cq	-	**Qui-1**	106.0, CH	4.45, d (7.9)
**29**	33.5, CH_3	0.88, s	**Qui-2**	73.7, CH	3.46, m
**30**	24.9, CH_3	0.94, s	**Qui-3**	85.5, CH	3.68, m
**GlcA-1**	104.7, CH	4.40, d (7.7)	**Qui-4**	75.1, CH	4.63, d (9.5)
**GlcA-2**	78.3, CH	3.64, m	**Qui-5**	75.2, CH	3.38, m
**GlcA-3**	86.8, CH	3.68, m	**Qui-6**	18.2, CH_3	1.27, d (6.2)
**GlcA-4**	71.5, CH	3.56, m	**4-OAc**	21.3, CH_3/172.2, Cq	2.04, s/-
**GlcA-5**	75.2, CH	3.79, m	**C_{28}-Xyl (3)-1**	106.9, CH	4.40, d (7.7)
**GlcA-6**	ND	-	**C_{28}-Xyl (3)-2**	75.8, CH	3.17, m
**Gal-1**	103.9, CH	4.81, d (7.6)	**C_{28}-Xyl (3)-3**	77.9, CH	3.38, m
**Gal-2**	73.6, CH	3.46, m	**C_{28}-Xyl (3)-4**	71.0, CH	3.51, m
**Gal-3**	75.3, CH	3.36, m	**C_{28}-Xyl (3)-5**	67.1, CH_2	3.88/3.22, m
**Gal-4**	70.9, CH	3.81, m			
**Gal-5**	76.7, CH	3.48, m			
**Gal-6**	62.3, CH_2	3.74/3.74, m			

Supplementary Table 2. ^{1}H, ^{13}C NMR spectroscopic data recorded for saponarioside B (13), MeOH-d_4 (600/150 MHz).

No.	δ_C, Type	δ_H mult, (J in Hz)	No.	δ_C, Type	δ_H mult, (J in Hz)
**1**	39.4, CH_2	1.71/1.10, m	**Gal-3**	75.4, CH	3.37, m
**2**	25.9, CH_2	1.98/1.78, m	**Gal-4**	70.9, CH	3.82, m
**3**	86.5, CH	3.87, dd (12.3, 4.8)	**Gal-5**	76.7, CH	3.49, m
**4**	56.5, Cq	-	**Gal-6**	62.3, CH_2	3.75/3.75, m
**5**	49.4, CH, overlapped	1.32, m	**C_3-Xyl-1**	105.1, CH	4.59, d (7.7)
**6**	21.6, CH_2	1.48/0.93, m	**C_3-Xyl-2**	75.5, CH	3.23, m
**7**	33.7, CH_2	1.49/1.35, m	**C_3-Xyl-3**	78.4, CH	3.30, overlapped
**8**	41.2, Cq	-	**C_3-Xyl-4**	71.2, CH	3.51, m
**9**	48.2, CH	1.73, m	**C_3-Xyl-5**	67.3, CH_2	3.91/3.24, m
**10**	37.3, Cq	-	**Fuc-1**	95.0, CH	5.30, d (7.8)
**11**	24.6, CH_2	1.92/1.92, m	**Fuc-2**	75.5, CH	3.80, m
**12**	123.4, CH	5.30, m	**Fuc-3**	77.4, CH	3.75, m
**13**	144.9, Cq	-	**Fuc-4**	84.2, CH	3.76, m
**14**	43.0, Cq	-	**Fuc-5**	72.2, CH	3.71, m
**15**	36.7, CH_2	1.94/1.44, m	**Fuc-6**	17.1, CH_3	1.24, d (6.4)
**16**	74.6, CH	4.47, m	**Rha-1**	101.6, CH	5.36, d (1.5)
**17**	50.2, Cq	-	**Rha-2**	71.9, CH	3.92, m
**18**	42.4, CH	2.94, dd (14.3, 3.9)	**Rha-3**	72.5, CH	3.79, m
**19**	48.1, CH_2	2.31, t (13.6)/1.05, m	**Rha-4**	85.3, CH	3.51, m
**20**	31.5, Cq	-	**Rha-5**	68.8, CH	3.77, m
**21**	36.8, CH_2	1.94/1.17, m	**Rha-6**	18.5, CH_3	1.31, d (6.2)
**22**	32.3, CH_2	1.93/1.73, m	**C_{28}-Xyl (1)-1**	107.2, CH	4.51, d (7.6)
**23**	211.4, CH	9.46, s	**C_{28}-Xyl (1)-2**	75.3, CH	3.37, m
**24**	11.1, CH_3	1.17, s	**C_{28}-Xyl (1)-3**	87.5, CH	3.48, m
**25**	16.6, CH_3	1.01, s	**C_{28}-Xyl (1)-4**	69.6, CH	3.54, m
**26**	17.8, CH_3	0.75, s	**C_{28}-Xyl (1)-5**	67.1, CH_2	3.88/3.22, m
**27**	27.3, CH_3	1.39, s	**C_{28}-Xyl (2)-1**	105.7, CH	4.52, d (6.7)
**28**	177.3, Cq	-	**C_{28}-Xyl (2)-2**	75.3, CH	3.37, m
**29**	33.5, CH_3	0.88, s	**C_{28}-Xyl (2)-3**	77.9, CH	3.38, m
**30**	24.9, CH_3	0.94, s	**C_{28}-Xyl (2)-4**	71.2, CH	3.58, m
**GlcA-1**	104.7, CH	4.43, d (7.6)	**C_{28}-Xyl (2)-5**	67.4, CH_2	3.93/3.28, m
**GlcA-2**	78.3, CH	3.65, m	**Qui-1**	106.2, CH	4.55, d (7.9)
**GlcA-3**	86.8, CH	3.68, m	**Qui-2**	73.5, CH	3.46, m
**GlcA-4**	71.8, CH	3.56, m	**Qui-3**	75.2, CH	3.13, m
**GlcA-5**	75.5, CH	3.79, m	**Qui-4**	79.2, CH	4.89, d (9.4)
**GlcA-6**	ND	-	**Qui-5**	75.2, CH	3.38, m
**Gal-1**	103.9, CH	4.81, d (7.6)	**Qui-6**	18.3, CH_3	1.27, d (6.1)
**Gal-2**	73.7, CH	3.47, m	**4-*O*-Ac**	20.9/172.8 CH_3/Cq	2.11, s/-

Supplementary Table 3. Summary statistics of *S. officinalis* genome assembly.

Pseudochromosome (1n)	14
Total length	2089.5 Mbp
Number of scaffolds	129
Scaffold L/N50	7/148.8 Mbp
Number of contigs	168
Contig L/N50	9/91.6 Mbp
Protein coding genes	37604
BUSCO Score	95.2 %

Supplementary Table 4. Summary statistics of the assembled soapwort pseudochromosome. The GC content of the remaining 115 contigs are available in Supplementary Data 1.

Scaffold	Contigs	Scaffold Size (bp)	GC (%)
Chr 01	3	171,998,431	39.41
Chr 02	8	168,346,886	39.37
Chr 03	6	167,862,214	39.00
Chr 04	3	151,746,589	39.07
Chr 05	2	150,658,900	38.97
Chr 06	3	148,914,749	39.13
Chr 07	4	148,844,233	39.40
Chr 08	1	146,996,705	39.28
Chr 09	6	146,090,521	39.10
Chr 10	1	142,121,670	39.17
Chr 11	2	141,745,259	39.30
Chr 12	6	136,675,473	39.22
Chr 13	5	129,907,096	39.30
Chr 14	3	126,650,553	38.99
Remaining	115	11,376,882	

Supplementary Table 5. Transcript read counts of candidate OSCs in different soapwort organs.

Gene ID	Flower	Flower Bud	Young Leaf	Old Leaf	Stem	Root
Saoffv11027757	12836.96	9677.26	4230.58	1270.73	1177.65	1349.48
Saoffv11008135	3870.93	2440.89	2536.82	2633.58	1891.88	1669.22
Saoffv11003490	233.09	266.29	406.24	397.76	259.66	181.83
Saoffv11043295	169.40	6.62	4.37	8.48	17.47	2.06

Supplementary Table 6. Oxidosqualene cylcase sequences and species used for Fig 2A.

OSC name	GenBank ID	Species
AsCS1	CAC84559.1	*Avena strigosa*
BpBPW	BAB83087.1	*Betula platyphylla*
BpBPX1	BAB83085.1	*Betula platyphylla*
BpBPX2	BAB83086.1	*Betula platyphylla*
BpBPY	BAB83088.1	*Betula platyphylla*
GgbAS1	BAA89815.1	*Glycyrrhiza glabra*
GgCAS1	BAA76902.1	*Glycyrrhiza glabra*
GgLUS1	BAD08587.1	*Glycyrrhiza glabra*
GmBAS3	AHI17180.1	*Glycine max*
LjAMY2	AAO33580.1	*Lotus japonicus*
LjLAS	BAE95410.1	*Lotus japonicus*
LjOSC1	BAE53429.1	*Lotus japonicus*
LjOSC3	BAE53430.1	*Lotus japonicus*
LjOSC5	BAE53431.1	*Lotus japonicus*
LjOSC9	BBK68801.1	*Lotus japonicus*
MtBAS1	CAD23247.1	*Medicago truncatula*
PgDS	ACZ71036.1	*Panax ginseng*
PgPNA	BAF33291.1	*Panax ginseng*
PgPNX	BAA33460.1	*Panax ginseng*
PgPNY1	BAA33461.1	*Panax ginseng*
PgPNY2	BAA33722.1	*Panax ginseng*
PgPNZ1	BAA33462.1	*Panax ginseng*
PsPSM	BAA97559.1	*Pisum sativum*
PsPSX	BAA23533.1	*Pisum sativum*
PsPSY	BAA97558.1	*Pisum sativum*
PtBS	ABL07607.1	*Polygala tenuifolia*
PtCAS1	ABX75046.1	*Polygala tenuifolia*
PtCAS2	ABX75048.2	*Polygala tenuifolia*
QsbAS1	WEU75096.1	*Quillaja saponaria*
VhBS	ABK76265.1	*Vaccaria hispanica*

Supplementary Table 7. ^{1}H, ^{13}C NMR spectroscopic data recorded for QA-TriF(Q)RXX (12) standard material purified from soapwort flowers, MeOH-d_4 (600/150 MHz).

No.	δ_C, Type	δ_H mult, (*J* in Hz)	No.	δ_C, Type	δ_H mult, (*J* in Hz)
**1**	39.4, CH_2	1.71/1.10, m	**Gal-3**	75.4, CH	3.37, m
**2**	25.9, CH_2	1.99/1.79, m	**Gal-4**	70.9, CH	3.82, m
**3**	86.5, CH	3.88, m	**Gal-5**	76.7, CH	3.49, m
**4**	56.5, Cq	-	**Gal-6**	62.3, CH_2	3.76/3.76, m
**5**	49.3, CH, overlapped	1.33, m	**C_3-Xyl-1**	105.1, CH	4.59, d (7.7)
**6**	21.6, CH_2	1.48/0.93, m	**C_3-Xyl-2**	75.5, CH	3.24, m
**7**	33.7, CH_2	1.49/1.36, m	**C_3-Xyl-3**	78.3, CH	3.31, overlapped
**8**	41.2, Cq	-	**C_3-Xyl-4**	71.2, CH	3.51, m
**9**	48.1, CH	1.75, m	**C_3-Xyl-5**	67.3, CH_2	3.91/3.24, m
**10**	37.3, Cq	-	**Fuc-1**	95.0, CH	5.30, d (8.1)
**11**	24.6, CH_2	1.92/1.92, m	**Fuc-2**	75.4, CH	3.81, m
**12**	123.4, CH	5.32, m	**Fuc-3**	77.4, CH	3.75, m
**13**	144.9, Cq	-	**Fuc-4**	84.1, CH	3.72, m
**14**	43.0, Cq	-	**Fuc-5**	72.0, CH	3.70, m
**15**	36.7, CH_2	1.94/1.45, m	**Fuc-6**	17.1, CH_3	1.24, d (6.4)
**16**	74.6, CH	4.47, m	**Rha-1**	101.6, CH	5.38, d (1.5)
**17**	50.2, Cq	-	**Rha-2**	72.0, CH	3.94, m
**18**	42.4, CH	2.94, dd (14.3, 4.0)	**Rha-3**	72.5, CH	3.80, m
**19**	48.2, CH_2	2.31, t (13.6)/1.05, m	**Rha-4**	85.3, CH	3.53, m
**20**	31.5, Cq	-	**Rha-5**	68.9, CH	3.77, m
**21**	36.8, CH_2	1.95/1.17, m	**Rha-6**	18.5, CH_3	1.32, d (6.2)
**22**	32.3, CH_2	1.94/1.73, m	**C_{28}-Xyl (1)-1**	107.2, CH	4.51, d (7.6)
**23**	211.4, CH	9.45, s	**C_{28}-Xyl (1)-2**	75.3, CH	3.38, m
**24**	11.1, CH_3	1.17, s	**C_{28}-Xyl (1)-3**	87.5, CH	3.49, m
**25**	16.6, CH_3	1.01, s	**C_{28}-Xyl (1)-4**	69.6, CH	3.55, m
**26**	17.8, CH_3	0.75, s	**C_{28}-Xyl (1)-5**	67.1, CH_2	3.88/3.22, m
**27**	27.3, CH_3	1.39, s	**C_{28}-Xyl (2)-1**	105.7, CH	4.52, d (6.5)
**28**	177.3, Cq	-	**C_{28}-Xyl (2)-2**	75.3, CH	3.38, m
**29**	33.5, CH_3	0.88, s	**C_{28}-Xyl (2)-3**	77.9, CH	3.39, m
**30**	24.9, CH_3	0.94, s	**C_{28}-Xyl (2)-4**	71.1, CH	3.59, m
**GlcA-1**	104.7, CH	4.43, d (7.6)	**C_{28}-Xyl (2)-5**	67.4, CH_2	3.93/3.29, m
**GlcA-2**	78.3, CH	3.65, m	**Qui-1**	106.5, CH	4.45, d (7.7)
**GlcA-3**	86.8, CH	3.69, m	**Qui-2**	78.2, CH	3.31, m
**GlcA-4**	71.5, CH	3.56, m	**Qui-3**	71.2, CH	3.56, m
**GlcA-5**	75.5, CH	3.82, m	**Qui-4**	76.7, CH	3.49, m
**GlcA-6**	ND	-	**Qui-5**	73.7, CH	3.30, m
**Gal-1**	103.9, CH	4.81, d (7.6)	**Qui-6**	18.3, CH_3	1.26, d (6.1)
**Gal-2**	73.7, CH	3.47, m			

Supplementary Table 8. NMR spectroscopic data [(^{1}H, ^{13}C), anomeric protons] for QA-TriF(Q)RXX (12) produced in *N. benthamiana* compared to literature, both recorded in pyridine-*d*5.

Sugar	Recorded			Literature[14]		
	^{1}H-NMR	^{13}C-NMR	Coupled HSQC ($^1J_{H\text{-}C}$) Hz	^{1}H-NMR	^{13}C-NMR	Coupled HSQC ($^1J_{H\text{-}C}$) Hz
GlcA-1	4.89	103.7	162	4.89	103.9	156
Gal-1	5.58	104.3	162	5.55	104.2	163
Xyl(C-3)-1	5.36	105.0	162	5.32	105.0	152
Fuc-1	5.97	94.5	168	5.94	94.5	167
Rha-1	6.42	101.0	174	6.23	101.3	171
Xyl'(C-28)-1	5.24	106.4	156	5.15	106.2	150
Xyl''(C-28)-1	5.18	106.1	162	5.14	105.9	156
Qui-1	5.00	106.7	162	4.99	106.2	162

Supplementary Table 9. ^{1}H, ^{13}C NMR spectroscopic data recorded for SO1699 (13′), MeOH-d_4 (600/150 MHz)

No.	δ_C, Type	δ_H mult, (J in Hz)	No.	δ_C, Type	δ_H mult, (J in Hz)
**1**	39.4, CH_2	1.72/1.10, m	**Gal-3**	75.4, CH	3.37, m
**2**	25.9, CH_2	1.96/1.78, m	**Gal-4**	70.9, CH	3.82, m
**3**	86.6, CH	3.87, m	**Gal-5**	76.2, CH	3.49, m
**4**	56.5, Cq	-	**Gal-6**	62.3, CH_2	3.75/3.75, m
**5**	49.4, CH, overlapped	1.32, m	**C_3-Xyl-1**	105.1, CH	4.58, d (8.2)
**6**	21.6, CH_2	1.48/0.94, m	**C_3-Xyl-2**	75.5, CH	3.23, m
**7**	33.7, CH_2	1.49/1.36, m	**C_3-Xyl-3**	78.4, CH	3.30, overlapped
**8**	41.2, Cq	-	**C_3-Xyl-4**	71.2, CH	3.50, m
**9**	48.1, CH	1.74, m	**C_3-Xyl-5**	67.3, CH_2	3.91/3.24, m
**10**	37.3, Cq	-	**Fuc-1**	95.0, CH	5.30, d (8.1)
**11**	24.6, CH_2	1.92/1.92, m	**Fuc-2**	75.5, CH	3.80, m
**12**	123.4, CH	5.31, m	**Fuc-3**	77.4, CH	3.75, m
**13**	144.9, Cq	-	**Fuc-4**	84.2, CH	3.74, m
**14**	43.0, Cq	-	**Fuc-5**	72.2, CH	3.70, m
**15**	36.7, CH_2	1.94/1.44, m	**Fuc-6**	17.1, CH_3	1.24, d (6.4)
**16**	74.6, CH	4.48, m	**Rha-1**	101.6, CH	5.38, d (1.4)
**17**	50.2, Cq	-	**Rha-2**	71.9, CH	3.92, m
**18**	42.4, CH	2.94, dd (14.3, 4.1)	**Rha-3**	72.5, CH	3.79, m
**19**	48.2, CH_2	2.31/1.05, m	**Rha-4**	85.3, CH	3.52, m
**20**	31.5, Cq	-	**Rha-5**	68.8, CH	3.77, m
**21**	36.8, CH_2	1.94/1.17, m	**Rha-6**	18.5, CH_3	1.32, d (6.2)
**22**	32.4, CH_2	1.93/1.72, m	**C_{28}-Xyl (1)-1**	107.2, CH	4.51, d (3.7)
**23**	211.4, CH	9.46, s	**C_{28}-Xyl (1)-2**	75.4, CH	3.36, m
**24**	11.1, CH_3	1.18, s	**C_{28}-Xyl (1)-3**	77.9, CH	3.38, m*
**25**	16.6, CH_3	1.01, s	**C_{28}-Xyl (1)-4**	69.6, CH	3.54, m
**26**	17.8, CH_3	0.75, s	**C_{28}-Xyl (1)-5**	67.1, CH_2	3.89/3.22, m*
**27**	27.3, CH_3	1.39, s	**C_{28}-Xyl (2)-1**	105.7, CH	4.52, d (6.3)
**28**	177.3, Cq	-	**C_{28}-Xyl (2)-2**	75.6, CH	3.37, m
**29**	33.5, CH_3	0.88, s	**C_{28}-Xyl (2)-3**	77.9, CH	3.38, m
**30**	24.9, CH_3	0.94, s	**C_{28}-Xyl (2)-4**	71.0, CH	3.50, m
**GlcA-1**	104.8, CH	4.46, d (7.5)	**C_{28}-Xyl (2)-5**	67.4, CH_2	3.92/3.28, m
**GlcA-2**	78.2, CH	3.65, m	**Qui-1**	106.4, CH	4.51, d (3.3)
**GlcA-3**	86.8, CH	3.68, m	**Qui-2**	75.5, CH	3.37, m
**GlcA-4**	71.2, CH	3.56, m	**Qui-3**	87.4, CH	3.49, m
**GlcA-5**	75.5, CH	3.79, m	**Qui-4**	77.2, CH	4.57, d (9.7)
**GlcA-6**	ND	-	**Qui-5**	71.4, CH	3.48, m
**Gal-1**	103.9, CH	4.81, d (7.6)	**Qui-6**	17.8, CH_3	1.14, d (6.2)
**Gal-2**	73.7, CH	3.47, m	**4-*O*-Ac**	21.1/172.3 CH_3/Cq	2.09, s/-

*Chemical shift might be changeable

Supplementary Table 10. ^{1}H, ^{13}C NMR spectroscopic data recorded for QA-Trix-F (8), MeOH-d_4 (600/150 MHz).

No.	δ_C, Type	δ_H mult, (J in Hz)	No.	δ_C, Type	δ_H mult, (J in Hz)
**1**	39.5, CH_2	1.69/1.09, m	**28**	177.5, Cq	-
**2**	25.7, CH_2	2.05/1.77, m	**29**	33.5, CH_3	0.90, s
**3**	85.9, CH	3.86, m	**30**	25.1, CH_3	0.96, s
**4**	56.5, Cq	-	**GlcA-1**	104.5, CH	4.36, d (7.4)
**5**	48.8, CH, overlapped	1.31, m	**GlcA-2**	78.3, CH	3.65, m
**6**	21.4, CH_2	1.48/0.90, m	**GlcA-3**	86.8, CH	3.66, m
**7**	33.6, CH_2	1.53/1.24, m	**GlcA-4**	71.3, CH	3.56, m
**8**	41.3, Cq	-	**GlcA-5**	76.8, CH	3.48, m
**9**	48.2, CH	1.74, m	**GlcA-6**	176.3, Cq	-
**10**	37.2, Cq	-	**Gal-1**	104.0, CH	4.79, d (7.3)
**11**	24.6, CH_2	1.92/1.92, m	**Gal-2**	73.7, CH	3.46, m
**12**	123.6, CH	5.31, br t (3.5)	**Gal-3**	75.5, CH	3.43, m
**13**	144.8, Cq	-	**Gal-4**	70.9, CH	3.80, m
**14**	42.8, Cq	-	**Gal-5**	76.7, CH	3.48, m
**15**	36.4, CH_2	1.86/1.43, m	**Gal-6**	62.3, CH_2	3.75/3.72, m
**16**	75.1, CH	4.53, m	**Xyl-1**	104.9, CH	4.61, d (7.7)
**17**	50.0, Cq	-	**Xyl-2**	75.5, CH	3.24, m
**18**	42.2, CH	3.01, dd (14.5, 4.1)	**Xyl-3**	78.3, CH	3.29, overlapped
**19**	47.9, CH_2	2.30/1.05, m	**Xyl-4**	70.9, CH	3.49, m
**20**	31.5, Cq	-	**Xyl-5**	67.3, CH_2	3.88/3.21, m
**21**	36.6, CH_2	1.93/1.16, m	**Fuc-1**	96.2, CH	5.28, d (8.2)
**22**	32.1, CH_2	1.93/1.77, m	**Fuc-2**	78.3, CH	3.57, m
**23**	210.8, CH	9.42, s	**Fuc-3**	75.5, CH	3.49, m
**24**	10.9, CH_3	1.13, s	**Fuc-4**	75.5, CH*	3.50, m*
**25**	16.5, CH_3	0.99, s	**Fuc-5**	73.1, CH	3.71, m
**26**	17.9, CH_3	0.77, s	**Fuc-6**	16.8, CH_3	1.23, d (6.5)
**27**	27.4, CH_3	1.39, s			

*Chemical shift may be changeable

Supplementary References

1 Murashige, T. & Skoog, F. A revised medium for rapid growth and bio assays with tobacco tissue cultures. *Physiologia Plantarum* **15**, 473-497 (1962).

2 Untergasser, A., Cutcutache, I., Koressaar, T., Ye, J., Faircloth, B.C., Remm, M. & Rozen, S.G. Primer3—new capabilities and interfaces. *Nucleic Acids Research* **40**, e115-e115 (2012).

3 Livak, K. J. & Schmittgen, T. D. Analysis of relative gene expression data using real-time quantitative PCR and the $2^{-\Delta\Delta CT}$ method. *Methods* **25**, 402-408 (2001).

4 Stephenson, M. J., Reed, J., Brouwer, B. & Osbourn, A. Transient expression in *Nicotiana benthamiana* leaves for triterpene production at a preparative scale. *JoVE (Journal of Visualized Experiments)* **138**, e58169 (2018).

5 Reed, J., Orme, A., El-Demerdash, A., Owen, C., Martin, L.B., Misra, R.C., Kikuchi, S., Rejzek, M., Martin, A.C., Harkess, A., Leebens-Mack, J., Louveau, T., Stephenson, M. J. & Osbourn, A. Elucidation of the pathway for biosynthesis of saponin adjuvants from the soapbark tree. *Science* **379**, 1252-1264 (2023).

6 Perlin, A. S., and B. Casu. "Carbon-13 and proton magnetic resonance spectra of D-glucose-^{13}C." *Tetrahedron Letters* **10**, 2921-2924 (1969).

7 Mazzola, E.P., Parkinson, A., Kennelly, E.J., Coxon, B., Einbond, L.S. and Freedberg, D.I. "Utility of coupled-HSQC experiments in the intact structural elucidation of three complex saponins from Blighia sapida." *Carbohydrate research* **346**, 759-768 (2011).

8 Malhotra, K. & Franke, J. Cytochrome P450 monooxygenase-mediated tailoring of triterpenoids and steroids in plants. *Beilstein Journal of Organic Chemistry* **18**, 1289-1310 (2022).

9 Reed, J., Orme, A., El-Demerdash, A., Owen, C., Martin, L.B., Misra, R.C., Kikuchi, S., Rejzek, M., Martin, A.C., Harkess, A., Leebens-Mack, J., Louveau, T., Stephenson, M. J. & Osbourn, A. Elucidation of the pathway for biosynthesis of saponin adjuvants from the soapbark tree. *Science* **379**, 1252-1264 (2023).

10 Louveau, T. & Osbourn, A. The sweet side of plant-specialized metabolism. *Cold Spring Harbor Perspectives in Biology* **11**, a034744 (2019).

11 Wilson, A. E. & Tian, L. Phylogenomic analysis of UDP-dependent glycosyltransferases provides insights into the evolutionary landscape of glycosylation in plant metabolism. *The Plant Journal* **100**, 1273-1288 (2019).

12 Kruse, L. H., Weigle, A.T., Irfan, M., Martínez-Gómez, J., Chobirko, J.D., Schaffer, J.E., Bennett, A.A., Specht, C.D., Jez, J.M., Shukla, D. & Moghe, G.D. Orthology-based analysis helps map evolutionary diversification and predict substrate class use of BAHD acyltransferases. *The Plant Journal* **111**, 1453-1468 (2022).

13 Emms, D. M. & Kelly, S. OrthoFinder: phylogenetic orthology inference for comparative genomics. *Genome Biology* **20**, 238 (2019).

14 Jia, Z. H., Koike, K. & Nikaido, T. Major triterpenoid saponins from *Saponaria officinalis*. *Journal of Natural Products* **61**, 1368-1373 (1998).
