# Supplementary material for: Unlocking saponin biosynthesis in soapwort
Source: Nat Chem Biol. 2024 Jul 23;21(2):215–26. doi: 10.1038/s41589-024-01681-7 (PMC11782082; doi:10.1038/s41589-024-01681-7)
Supplement: Supplementary file 2 — Reporting Summary [file 41589_2024_1681_MOESM2_ESM.pdf]

nature portfolio

Corresponding author(s): Anne Osbourn

Last updated by author(s): Jun 4, 2024

Reporting Summary

Nature Portfolio wishes to improve the reproducibility of the work that we publish. This form provides structure for consistency and transparency in reporting. For further information on Nature Portfolio policies, see our Editorial Policies and the Editorial Policy Checklist.

Statistics

For all statistical analyses, confirm that the following items are present in the figure legend, table legend, main text, or Methods section.

n/a	Confirmed	
☐	☒	The exact sample size (*n*) for each experimental group/condition, given as a discrete number and unit of measurement
☐	☒	A statement on whether measurements were taken from distinct samples or whether the same sample was measured repeatedly
☐	☒	The statistical test(s) used AND whether they are one- or two-sided<br>*Only common tests should be described solely by name; describe more complex techniques in the Methods section.*
☒	☐	A description of all covariates tested
☒	☐	A description of any assumptions or corrections, such as tests of normality and adjustment for multiple comparisons
☐	☒	A full description of the statistical parameters including central tendency (e.g. means) or other basic estimates (e.g. regression coefficient) AND variation (e.g. standard deviation) or associated estimates of uncertainty (e.g. confidence intervals)
☐	☒	For null hypothesis testing, the test statistic (e.g. *F*, *t*, *r*) with confidence intervals, effect sizes, degrees of freedom and *P* value noted<br>*Give P values as exact values whenever suitable.*
☒	☐	For Bayesian analysis, information on the choice of priors and Markov chain Monte Carlo settings
☒	☐	For hierarchical and complex designs, identification of the appropriate level for tests and full reporting of outcomes
☐	☒	Estimates of effect sizes (e.g. Cohen's *d*, Pearson's *r*), indicating how they were calculated

Our web collection on statistics for biologists contains articles on many of the points above.

Software and code

Policy information about availability of computer code

Data collection	MassHunter Workstation 10.0; Xcalibur 4.3; Hifiasm 0.16.1; RACON 1.4.3; JUICER 1.6; PERTRAN 1.0; MestReNova version 6.0.2-5475
Data analysis	InterProScan 5.22; MAFFT 7.127; RaxML 2.1.9; FreeStyle 1.6; HMMER-3.1b2; R Studio 1.4.1103; AUGUSTUS 3.4.0; BLAST+ 2.8.1; OrthoFinder 2.5.4; GENESPACE 1.3.1; DEseq2 1.6.3; MestReNova version 6.0.2-5475

For manuscripts utilizing custom algorithms or software that are central to the research but not yet described in published literature, software must be made available to editors and reviewers. We strongly encourage code deposition in a community repository (e.g. GitHub). See the Nature Portfolio guidelines for submitting code & software for further information.

Data

Policy information about availability of data

All manuscripts must include a data availability statement. This statement should provide the following information, where applicable:

- Accession codes, unique identifiers, or web links for publicly available datasets
- A description of any restrictions on data availability
- For clinical datasets or third party data, please ensure that the statement adheres to our policy

The fully assembled and annotated S. officinalis genome sequence has been deposited under BioProject ID PRJNA1018723. The RNA-seq reads are deposited under BioProject ID PRJNA1008697 and PRJNA1035542. The sequences of the genes characterized in this study can also be found in GenBank as follows: SobAS1 (OR426407), CYP716A378 (OR426395), CYP716A379 (OR426402), CYP72A984 (OR426401), SoCSL1 (OR426404), UGT73DL1 (OR426405), UGT73CC6 (OR426403),

SoSDR1 (OR426396), UGT74CD1 (OR426399), UGT79T1 (OR426408), UGT79L3 (OR426397), UGT73M2 (OR426400), SoGH1 (OR426398), SoBAHD1 (OR426406). InterPro-85.0 (https://www.ebi.ac.uk/interpro/) and Pfam-33.1 (http://pfam.xfam.org/). The data that support the findings of this study is available within the main text and its Supplementary Information file. Source data are provided as Source Data files. Data is also available from the corresponding author upon request.

Human research participants

Policy information about studies involving human research participants and Sex and Gender in Research.

Reporting on sex and gender	n/a
Population characteristics	n/a
Recruitment	n/a
Ethics oversight	n/a

Note that full information on the approval of the study protocol must also be provided in the manuscript.

Field-specific reporting

Please select the one below that is the best fit for your research. If you are not sure, read the appropriate sections before making your selection.

☒ Life sciences ☐ Behavioural & social sciences ☐ Ecological, evolutionary & environmental sciences

For a reference copy of the document with all sections, see nature.com/documents/nr-reporting-summary-flat.pdf

Life sciences study design

All studies must disclose on these points even when the disclosure is negative.

Sample size	Sample sizes for all experiments were performed at least in triplicates to allow for statistical analysis when required.
Data exclusions	No data were excluded from the analyses
Replication	Details of all biological and technical replicates are provided in respective figure legends, methods and supplementary information.
Randomization	Not relevant as samples were all under same conditions
Blinding	No treatments were involved in this study thus blinding was unnecessary. All data was collected from samples and analyzed for specific target compounds.

Reporting for specific materials, systems and methods

We require information from authors about some types of materials, experimental systems and methods used in many studies. Here, indicate whether each material, system or method listed is relevant to your study. If you are not sure if a list item applies to your research, read the appropriate section before selecting a response.

Materials & experimental systems

n/a	Involved in the study
☒	☐ Antibodies
☒	☐ Eukaryotic cell lines
☒	☐ Palaeontology and archaeology
☒	☐ Animals and other organisms
☒	☐ Clinical data
☒	☐ Dual use research of concern

Methods

n/a	Involved in the study
☒	☐ ChIP-seq
☒	☐ Flow cytometry
☒	☐ MRI-based neuroimaging
