# Supplementary figures and images for: Unlocking saponin biosynthesis in soapwort
Source: Nat Chem Biol. 2024 Jul 23;21(2):215–26. doi: 10.1038/s41589-024-01681-7 (PMC11782082; doi:10.1038/s41589-024-01681-7)

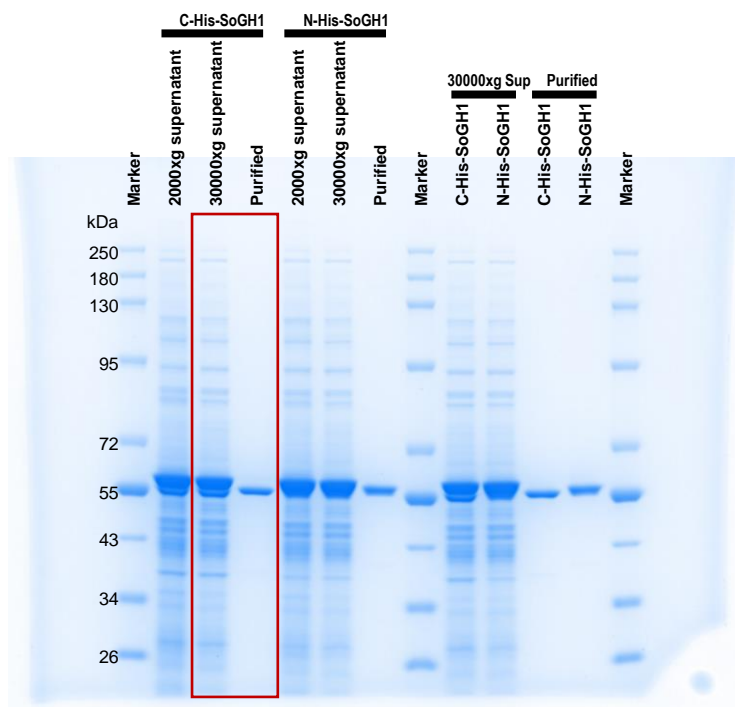

4-12% gradient gel

Supplement: Supplementary file 10 — Full gel image in Extended Data Fig. 5a. [file 41589_2024_1681_MOESM10_ESM.pdf]
